# Synthesis and Preliminary Evaluation of Biological Activity of Glycoconjugates Analogues of Acyclic Uridine Derivatives

**DOI:** 10.3390/molecules23082017

**Published:** 2018-08-13

**Authors:** Roman Komor, Gabriela Pastuch-Gawolek, Ewelina Krol, Wieslaw Szeja

**Affiliations:** 1 Department of Organic Chemistry, Bioorganic Chemistry and Biotechnology, Faculty of Chemistry, Silesian University of Technology, Krzywoustego 4, 44-100 Gliwice, Poland; wieslaw.szeja@adres.pl; 2 Biotechnology Center, Silesian University of Technology, Krzywoustego 8, 44-100 Gliwice, Poland; 3 Department of Recombinant Vaccines, Intercollegiate Faculty of Biotechnology, University of Gdansk and Medical University of Gdansk, Abrahama 58, 80-307 Gdansk, Poland; ewelina@biotech.ug.gda.pl

**Keywords:** glycosyltransferases, glycosyltransferase inhibitors, glycoconjugates, acyclic uridine derivatives, thioglycosides

## Abstract

Herein we present the methodology for obtaining glycosyltransferase inhibitors, analogues of natural enzyme substrates of donor-type: UDP-glucose and UDP-galactose. The synthesis concerned glycoconjugates, nucleoside analogues containing an acyclic ribose mimetic linked to a uracil moiety in their structure. The biological activity of the synthesised compounds was determined on the basis of their ability to inhibit the model enzyme action of β-1,4-galactosyltransferase from bovine milk. The obtained results allowed to expand and supplement the existing library of synthetic compounds that are able to regulate the biological activity of enzymes from the GT class.

## 1. Introduction

Glycosyltransferases (GTs), a superfamily of enzymes, are involved in synthesising the carbohydrate moieties of glycoproteins, glycolipids, and glycosaminoglycans, which are involved in many important biological functions. These glycoconjugates have various specific roles in cell growth and cell–cell interactions [1], cell adhesion including fertilisation [2,3], modulation of growth factor receptors [4], immune defence [5,6], inflammation [7], host–pathogen interactions [8,9,10], and both viral and parasitic infections [9]. The structures of oligosaccharides are significantly changed during such processes as cellular development, differentiation, and tumorigenesis [11,12,13], and in many disease states [14,15]. Specific glycosyltransferases synthesize oligosaccharides by the sequential transfer of the monosaccharide moiety of an activated sugar donor to an acceptor molecule. One of the most extensively studied and characterised glycosyltransferase is β-1,4-galactosyltransferase I (β4GalT). This enzyme transfers d-galactose moiety from a donor molecule to a hydroxyl group of a specific acceptor molecule which can be an oligosaccharide, a protein or a lipid [16,17]. All of these enzymes use uridine 5′-diphospho-α-d-galactose (UDP-Gal) as the donor, and many of these enzymes require a metal ion cofactor, generally a Mn^2+^ ion, for activity [18,19]. The catalytic domain of the enzyme has two flexible loops: a small loop and a long loop. The long flexible loop contains the primary metal binding site at its terminal hinge region. Both flexible loops undergo a notable conformational change from an open to a closed conformation upon binding of sugar–nucleotide and metal ion. The enzyme active site is created by an ordered binding of sugar–nucleotide and metal ion, followed by a conformational change that creates the acceptor-binding site. The reaction catalysed by GTs follows a kinetic mechanism in which the sugar nucleotide and metal ion bind to the enzyme active site first, followed by the acceptor [16].

The enzyme activity can be regulated by sugar donor analogues in which a nonionic linker that replaces the pyrophosphate moiety is capable of coordinating a metal ion present in the active site [7]. Small molecule compounds acting as selective glycosyltransferase inhibitors may provide potent drugs by blocking or preventing the synthesis of complex glycoconjugates and pathologies they cause [20].

UDP-sugar analogues (sugar donor analogues) design is based on modifications of one of the three building blocks of these molecules: the nucleoside part, the carbohydrate moiety, or the diphosphate connection [21,22,23,24,25]. The pyrophosphate bridge of the sugar donor molecule binds to a bivalent metal ion (Mn^2+^ or Mg^2+^) present in the active site of the enzyme therefore several analogues of UDP-sugar substrates with modifications of the diphosphate moiety have been researched and described [26,27]. However, due to anionic character of such compounds, their ability to efficiently penetrate the cells phospholipid bilayer is limited [12]. Numerous attempts to solve this problem have included synthesis of neutral glycosyltransferase inhibitors modified by monosaccharide moieties [28,29], tartaric or malonic acid derivatives [30], pyridine and/or triazole [31], amino acids [32,33], pyridine connected to succinic acid via amide bond [34], or thiophosphoester as a surrogate of the pyrophosphate part of the sugar donor [35,36].

Taking into account that the acceptor binding site is created by a conformational change after ordered binding of the sugar nucleotide and metal ion in the active site of the enzyme, in our approach to design inhibitors, we focus on the synthesis of the nucleoside part of the inhibitor strongly bonded by a flexible loop and weakly bonded with a metal ion, so as the creation of the catalytic enzyme part will be difficult.

**Figure 1:**
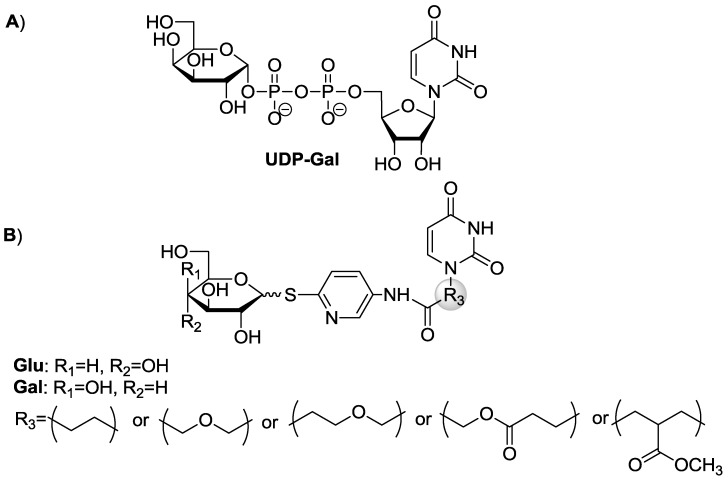
(**A**) Natural GTs substrate; (**B**) structures of synthesised GTs inhibitors.

Accepting this assumption, a series of UDP-Gal donor analogues was designed. The designed inhibitors contain components of natural glycosyltransferase donors in their structure: monosaccharide linked through a linker to the acyclic derivative of uridine (Figure 1).

## 2. Results and Discussion

**Scheme 1:**
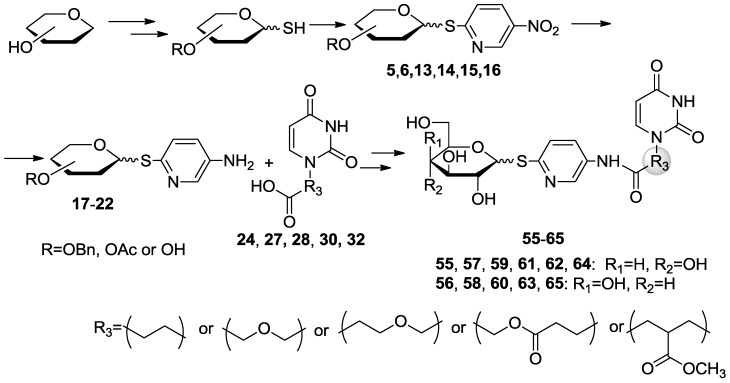
The general idea of GT inhibitors synthesis.

The enzyme β-(1,4)-galactosyltransferase (β4GalT) from bovine milk was selected as the subject of the study, which in the acceptor reaction with the glycosyl donor of α-configuration (uridine diphosphate galactose, UDP-Gal), carries the sugar molecule to form a β-(1,4)-glycosidic bond [20]. Based on literature studies, the following assumptions were made to design the inhibitor structures shown in Figure 1: the sugar unit should contain an amino terminal aglycone that will allow the amide linkage to acyclic nucleobase derivatives containing the terminal carboxyl group. Relevant uracil as a nucleic base is a key element of the inhibitor because of its strong affinity for the active enzyme site. The designed inhibitors are *S*-glycoside derivatives of such monosaccharides as d-glucose and d-galactose, in which the glycosidic bond has an α-configuration as in natural GTs substrates. *S*-Glycosides were selected because the *S*-glycosidic bond is well-tolerated by most biological systems and is more stable in hydrolysis reactions catalysed by acids or glycosylhydrolases [37,38,39] compared to *O*-glycosides. The design of a multistep synthesis of inhibitors is shown in Scheme 1.

The first step leading to obtaining the assumed glycoconjugate structures is the synthesis of the 1-thiosugar with the α-configuration, which is converted into the 1-thioglycoside by a reaction with the corresponding nitropyridine derivative, followed by a reduction of the nitro group to the amino group. The next stage is the synthesis of acyclic derivatives of uridine containing a carboxyl group in the terminal position. The final glycoconjugates are obtained by condensing the intermediates to form an amide bond and removing protecting groups.

**Scheme 2:**
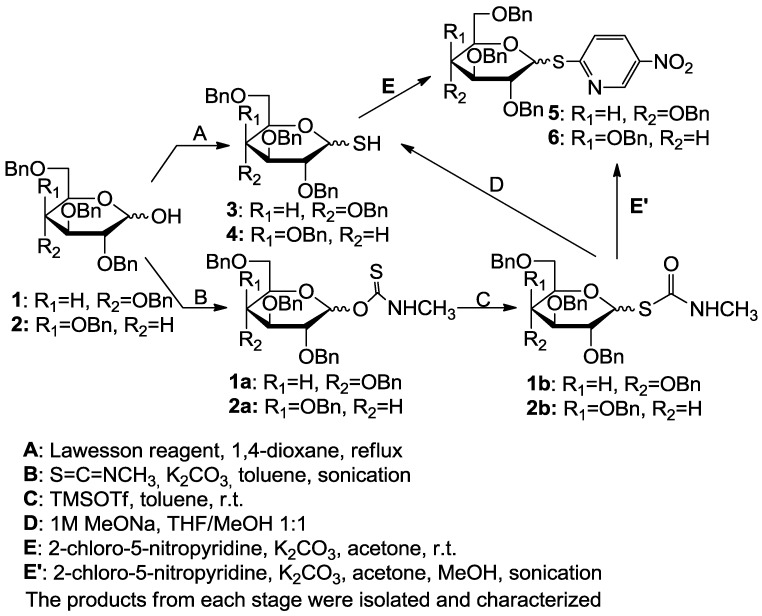
Synthesis of 1-thiosugars and the corresponding 1-thioglycosides.

While the synthesis of 1-β thiosugars is relatively simple to carry out and leads to the desired compounds at a high yield, the synthesis of analogues of the α-configuration poses many difficulties. However numerous reports for the α-glycosylthiol derivatives synthesis have appeared in the literature [40]. Although most of the reaction conditions described suffer from several limitations, including formation of mixture of isomers, multiple reaction and/or purification steps, long reaction time, low yield, use of hazardous gases (e.g., H_2_S), use of expensive reagents (e.g., (TMS)_2_S), necessity of use of appropriately functionalised substrates, etc. On the basis of literature studies, a method for transformation of alcohols to thiols using the Lawesson’s reagent was selected [41]. According to the procedure described by Bernardes et al., tetra-*O*-benzyl protected d-glucose or d-galactose was reacted with a 1.2 molar equivalent of the Lawesson reagent in 1,4-dioxane under argon (Scheme 2, procedure A). [42]. However, due to the low yields of the obtained 1-thioglucose **3** (46%) and 1-thiogalactose **4** (26%), as well as a low stereoselectivity of the reaction and difficulties in the separation of anomers, further attempts to obtain 1-thiosugars with α-configuration were made.

In this research the method using *N*-methyl thiolcarbamates for the synthesis of glycosyl thiols was applied (Scheme 2) [43]. Corresponding glycosylthiols are easy to obtain by alcoholysis and can be used for the preparation of heteroaryl 1-thioglycosides. Among the numerous methods of synthesising 1-thioglycosides described in the literature [40], for the synthesis of (5-nitro-2-pyridyl) 1-thioglycosides, the procedure of aromatic substitution of nucleophilic halogen in negatively substituted aryl derivatives [44] was used. Treatment of the 2,3,4,6-tetra-*O*-benzyl-1-thio-d-gluco- or 2,3,4,6-tetra-*O*-benzyl-1-thio-d-galactopyranose with 2-chloro-5-nitropyridine gives corresponding heteroaryl thioglycosides. The key step in the substitution of the chlorine atom in the 2-chloro-5-nitropyridine molecule was carried out in the field of ultrasound. After purification by column chromatography on silica gel (5-nitro-2-pyridyl) 2,3,4,6-tetra-*O*-benzyl-1-thio-d-glucopyranoside **5** (79% yield) or (5-nitro-2-pyridyl) 2,3,4,6-tetra-*O*-benzyl-1-thio-d-galactopyranoside **6** (77% yield) were obtained as mixtures, in which the α-anomer was the main product.

**Scheme 3:**
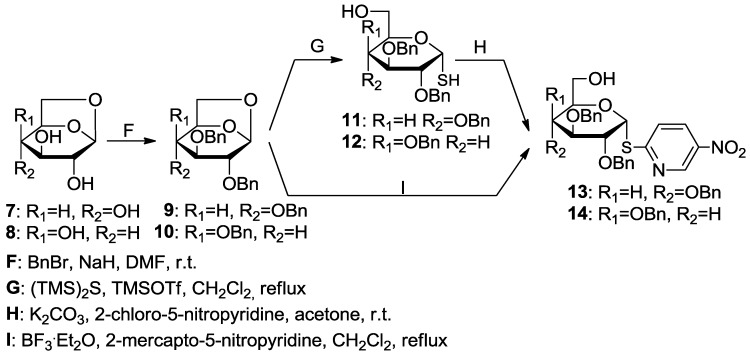
Application of 1,6-anhydrosugars in α-1-thioglyosides synthesis.

Because a mixture of anomeric 1-thioglycosides was obtained as a result of the described method using *N*-methyl thiolcarbamates in the next attempt to synthesise α-thioglycosides the ring opening reaction of 1,6-anhydrosugar with thiol catalysed by Lewis acids was applied [45]. The reactions of 2-mercapto-5-nitropyridine with per-*O*-benzyl-1,6-anhydroglucose **9** or per-*O*-benzyl-1,6-anhydrogalactose **10** were carried out in the presence of BF_3_ Et_2_O under an inert gas atmosphere (Scheme 3, procedure I). The desired compounds were obtained in the form of optically pure α-anomers, but with relatively low yields (36% for the d-glucose derivative **13** and 12% for the d-galactose derivative **14**). The undoubted advantage of this method is the creation of products with only the α configuration. Another variant of this method was the synthesis of α- thiosugar in the reaction of (TMS)_2_S with 1,6-anhydrosugar derivatives, in the presence of a Lewis acid, such as TMSOTf (Scheme 3, procedure G) [46,47]. The obtained thiosugars **11** and **12** in the reaction of aromatic nucleophilic substitution with 2-chloro-5-nitropyridine led to (5-nitro-2-pyridyl)1-thiogluco- (**13**) or (5-nitro-2-pyridyl)1-thiogalactopyranoside (**14**) in good yields (Scheme 3, procedure H, Table 1).

In the final step of the sugar derivatives synthesis the nitro group in aglycon of 1-thioglycosides was reduced to the amino group. After the comparison of conventional reduction procedures of (5-nitro-2-pyridyl) 1-thioglycosides, the method that uses iron dust and acetic acid in the THF-methanol 10:1 *v*/*v* solvent mixture, carried out in the field of ultrasound, proved to be the most effective [48,49]. The crude products of the nitro group reduction in α-thioglycosides **5**, **6**, **13**, and **14**, as well as in their acetylated analogues **15** and **16** with β-configuration at the anomeric centre were purified by column chromatography to give (5-amino-2-pyridyl) 1-thioglycosides **17**–**22** in good yields (Scheme 1).

**Scheme 4:**
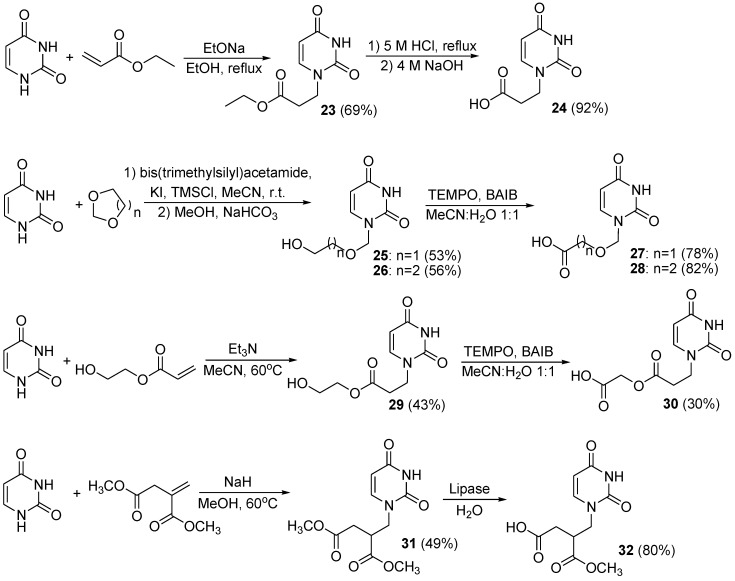
Synthesis of acyclic uridine derivatives.

In the next step of these studies, a synthesis of carboxylic group-containing uridine acyclic derivatives, intermediates in the construction of final glycoconjugates was carried out. The structure and corresponding procedures for the synthesis of acyclic uridine derivatives are shown in Scheme 4.

The acyclic derivative of uracil, 3-(2,4-dioxo-3,4-dihydropyrimidin-1(2*H*)-yl) propanoic acid **24** (Scheme 4) was obtained from 3-(2,4-dioxo-3,4-ethyl dihydropyrimidine-1(2*H*)-yl) propionate **23** by hydrolysis with 5M HCl at an elevated temperature [50]. Next, [(2,4-2-[(2,4-dioxo-3,4-dihydropyrimidin-1-(2*H*)-yl) methoxy]acetic acid **27** and 2-[(2,4-dioxo-3,4)-dihydropyrimidine-1-(2*H*)-yl) methoxy]propanoic acid **28** were obtained. The substrates for their preparation, 1-[(2-hydroxyethoxy)methyl]pyrimidine-2,4 (1*H*,3*H*)-dione **25** and 1-[(2-hydroxypropoxy)methyl]pyrimidine-2,4(1*H*,3*H*)-dione **26**, were synthesised according procedure found in the literature using 1,3-dioxolane or 1,3-dioxane [51]. For the oxidation of compounds **25** and **26**, the TEMPO/BAIB oxidation system has been implemented to obtain the desired products [52,53]. This oxidation system proved to be able to give the corresponding carboxylic derivatives **27** and **28** in good yields. The next obtained compound was 2-hydroxyethyl 3-(2,4-dioxo-3,4-dihydropyrimidin-1(2*H*)-yl)propanoate **29** [54], which was subjected to the oxidation of the terminal hydroxy group by a reaction with the TEMPO/BAIB system in a MeCN:H_2_O solvent mixture in analogy to the procedure for the preparation of compounds **27** and **28**. In this case, ([3-(2,4-dioxo-3,4-dihydropyrimidin-1(2*H*)-yl)propanoyl] oxyacetic acid **30** was obtained in a moderate yield.

The last synthesised intermediate for the synthesis of acyclic uridine derivatives containing the carboxyl group was dimethyl 2-[(2,4-dioxo-3,4-dihydropyrimidin-1(2*H*)-yl)methyl]succinate **31** [55]. For selective hydrolysis of one of the ester groups, **31** was subjected to the action of a hydrolytic enzyme. Ester-protecting groups can be removed with proteases or esterases; however, lipases are most often used for this purpose. Methods of enzymatic protection and deprotection of organic compounds are very attractive due to the regio- and stereoselectivity [56]. Attempts of selective deprotection were carried out with the use of lipase immobilised on a fixed bed of Novozyme^®^ 435. The reaction was carried out for 48 h at room temperature using water as the solvent. After isolation and purification of the crude product on silica gel, pure 3-[(2,4-dioxo-3,4-dihydropyrimidin-1(2*H*)-yl)methyl]-4-oxobutanoic acid **32** was obtained in good yield. 

**Scheme 5:**
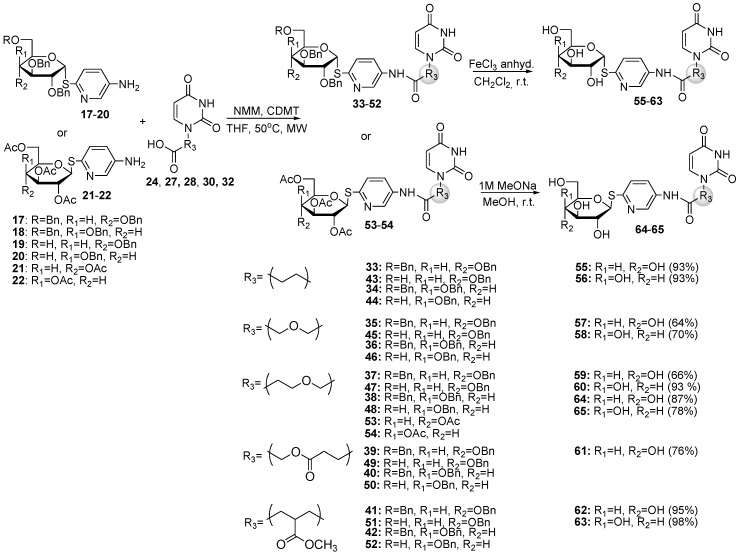
Synthesis of glycoconjugates of acyclic uridine derivatives.

Having all the substrates needed for conjugation, the synthesis of glycoconjugates, the potential donor type inhibitors of the GTs, was performed. A condensation reaction of (5-amino-2-pyridyl) 1-thioglycosides with acyclic uracil derivatives was applied to form an amide bond. Among the many known methods of chemical formation of the amide bond, it was decided to use the method described by Kamiński et al. [57] involving the creation of the so-called superactive esters. This method uses a condensing agent 4-(4,6-dimethoxy-1,3,5-triazin-2-yl)-4-methylmorpholinium chloride (DMTMM), which is obtained in the reaction between 2-chloro-4,6-disubstituted-1,3,5-triazines (CDMT) and N-methylmorpholine (NMM). This reagent can be generated directly in the reaction mixture. In the procedure used for the preparation of glycoconjugates, CDMT and NMM were added to the amine and the acyclic uracil derivative solution in THF. Microwave irradiation allowed for the reduction of the reaction time to a maximum of 4 hours (Scheme 5, Table 2). In order to compare the effect of the opposite configuration at the anomeric centre of the sugar on the final activity of glycoconjugates the synthesis of two glycoconjugates using (5-amino-2-pyridyl) 1-thio-β-d-glycosides **21** and **22** and acyclic uridine derivative **28** was also performed. As a result of the carried out reactions, the desired glycoconjugates **33**–**54** were obtained in moderate and good yields (Table 2).

In the glycoconjugates synthesis stages discussed so far, the protection of hydroxyl groups was necessary to ensure high regio- and stereoselectivity of individual steps of the synthesis. However, the obtained glycoconjugates, protected with acetyl or benzyl groups, are poorly soluble in water; therefore, deprotection was necessary before applying glycoconjugates for biological studies. The removal of the acetyl groups in glycoconjugates **53** and **54** was carried out according to Zemplen’s classic reaction conditions by transesterification in methanol with the addition of sodium methoxide [58] allowing the obtainment of glycoconjugates **64** and **65**, respectively. In turn, the benzyl group, commonly used in sugar chemistry, is stable in a wide range of reaction conditions and is relatively easy to be removed by catalytic hydrogenation [59,60]. However, due to the presence of a sulphur atom, which is the poison of the catalysts used in the removal reactions of benzyl groups [61,62,63], the deprotection of the obtained glycoconjugates should be carried out using Lewis acids [64]. For this purpose, anhydrous FeCl_3_ was applied for the deprotection of compounds **33**–**52** [65]. The reactions were carried out in an inert gas atmosphere. The crude products were purified on silica gel to obtain products **55**–**63** in moderate and good yields (Scheme 5). Spectroscopic data for a new compounds can be found in Supplementary Materials.

All of the obtained glycoconjugates **33**–**65** have been tested for their ability to inhibit β-1,4-galactosyltransferase (β4GalT) from bovine milk. The method was developed to perform a preliminary screening for large group of potential inhibitors and is based on the observation of the formation of the product in a reaction catalysed by β4GalT where a d-galactose unit from UDP-Gal molecule is transferred to (6-esculetinyl) β-d-glucopyranoside (esculine) acting as an acceptor molecule. Results are then compared to reactions carried out under the same conditions with the addition of synthesised glycoconjugates. Changes in concentrations of the substrate and the product are determined using RP-HPLC [66]. 

To determine the inhibition of the enzymatic reaction compounds were screened at 0.8 mM concentrations. To check whether the buffer used in the assay has an effect on the obtained results, several enzymatic reactions with the deprotected glycoconjugates **55**–**65** were repeated using Hepes buffer and citrate buffer in the pH 5.4. The results of enzymatic reactions were comparable in both cases.

However, none of the protected glycoconjugates demonstrated the ability to inhibit β4GalT. Surprisingly, it was found that the deprotected glycoconjugates **55**–**63**, in which the configuration at the anomeric centre of the sugar unit was the same as in the natural enzyme substrate UDP-Gal, were also not able to inhibit the tested bovine milk β-1,4-galactosyltransferase. 

Glycoconjugate **64**, which is a β-analogue of the inactive glycoconjugate **59**, demonstrated the ability for β4GalT I inhibition with the IC_50_ value of 0.71 mM, however this value is not low enough for the glycoconjugate to be a potent inhibitor. As an effective inhibitor, a compound showing an IC_50_ value less than 30 µM would be considered. 

It is significant that d-glucose derivative shows the ability to inhibit the enzyme activity, while the corresponding glycoconjugate **65**, derivative of d-galactose**,** is inactive. The presented results indicate that the connection of (5-amino-2-pyridyl) 1-thio-β-d-glucoside with an acyclic uridine derivative **28** via an amide bond allows for the synthesis of the UDP-sugar analogues that can act as glycosyltransferase inhibitors. Because of the relatively low activity of the obtained inhibitor we focused our research on the synthesis of large set of glycoconjugates and not on the mechanism of their action against β4GalT. It cannot be excluded that compound **64** competes with the rest of the glucose used in the acceptor. However, to determine the mechanism of their action further studies are needed.

The next step of the research will be a synthesis of a wide range of glycoconjugates of acyclic derivatives of uridine and 1-thioglycosides with β-configuration at the anomeric center. This will allow us to study the influence of the structure of acyclic uridine derivatives on the inhibitory activity of glycoconjugates and design a new class of inhibitors. In case of outstanding results indicating a high inhibitory activity, the IC_50_ value determined using the above assay, will be verified by using the procedure described by J.P. Praly and coworkers [26].

## 3. Materials and Methods

### 3.1. General Information

NMR spectra were recorded on Agilent Technologies spectrometer at a frequency of 400 MHz using NMR solvents were purchased from ACROS Organics (Geel, Belgium). Coupling constants (*J*) are in hertz (Hz). Chemical shifts (δ) are expressed in ppm downfield from TMS as an internal standard when CDCl_3_, DMSO-d6, or CD_3_OD were used as a solvents or DSS as an internal standard when D_2_O was used as a solvent. ^1^H-NMR and ^13^C-NMR signals of some compounds were assigned with the aid of COSY, HMQC, and HMBC. Microwave irradiation reactions were carried out with Discover BenchMate (CEM Microwave Technology Ltd., Buckingham, United Kingdom). Optical rotations were measured on a JASCO P-2000 polarimeter (JASCO International Co. Ltd., Tokyo, Japan) using a sodium lamp (589.3 nm) at a room temperature. High-resolution mass spectra were obtained using WATERS LCT Premier XE system (high resolution mass spectrometer with TOF analyser). Melting point measurements were performed on OptiMelt (MPA 100) (Stanford Research Systems, Sunnywale, CA, USA). Thin layer chromatography (TLC) reaction controls were performed using fluorescent silica gel 60 F254 (Merck Millipore, Burlington, MA, USA). TLC plates were visualised under UV illumination at 254 nm or charring after spraying with 10% sulphuric acid in ethanol. Crude products were purified using column chromatography performed on Silica Gel 60 (70-230 mesh, Fluka, Bucharest, Romania) developed with CHCl_3_/MeOH, hexane/EtOAc, or toluene/EtOAc solvent systems. The chromatographic separations (RP-HPLC) were performed using JASCO LC 2000 apparatus equipped with a fluorescence detector on a reverse phase column (Nucleosil 100 C18, 5 µm, 25 × 0.4 cm; mobile phase: H_2_O/MeCN 90:10, flow rate 0.8 mL/min.). Fluorescence for substrate and product was read at 385 nm excitation/540 nm emission. IC_50_ value for compound **64** was calculated using the computer program CalcuSyn.

2,3,4,6-Tetra-*O*-benzyl-d-glucopyranose (**1**), 2,3,4,6-tetra-*O*-benzyl-d-galactopyranose (**2**), 1,6-anhydro-d-glucopyranose (**7**) and 1,6-anhydro-d-galactopyranose (**8**) were purchased from Carbosynth Limited. 2,3,4,6-Tetra-*O*-benzyl-1-*O*-[*N*-methyl thionocarbamoyl]-d-glucopyranose (**1a**), 2,3,4,6-tetra-*O*-benzyl-1-*O*-[*N*-methyl thionocarbamoyl]-d-galactopyranose (**2a**), 2,3,4,6-tetra-*O*-benzyl-1-thio-[*N*-methyl thiolcarbamoyl]-d-glucopyranose (**1b**), 2,3,4,6-tetra-*O*-benzyl-1-thio-[*N*-methyl thiolcarbamoyl]-d-galactopyranose (**2b**), 2,3,4,6-tetra-*O*-benzyl-1-thio-d-glucopyranose (**3**), 2,3,4,6-tetra-*O*-benzyl-1-thio-d-galactopyranose (**4**), (5-nitro-2-pyridyl) 2,3,4,6-tetra-*O*-benzyl-1-thio-d-glucopyranoside (**5**), (5-nitro-2-pyridyl) 2,3,4,6-tetra-*O*-benzyl-1-thio-d-galactopyranoside (**6**) [43], 2,3,4-tri-*O*-benzyl-1,6-anhydro-β-d-glucopyranose (**9**), 2,3,4-tri-*O*-benzyl-1,6-anhydro-β-d-galactopyranose (**10**) [67], 2,3,4-tri-*O*-benzyl-1-thio-α-d-glucopyranoside (**11**), 2,3,4-tri-*O*-benzyl-1-thio-α-d-galactopyranoside (**12**) [47], (5-nitro-2-pyridyl) 2,3,4,6-tetra-*O*-acetyl-1-thio-β-d-glucopyranoside (**15**), (5-nitro-2-pyridyl) 2,3,4,6-tetra-*O*-acetyl-1-thio-β-d-galactopyranoside (**16**) [68], (5-amino-2-pyridyl) 2,3,4,6-tetra-*O*-acetyl-1-thio-β-d-glucopyranoside (**21**), (5-amino-2-pyridyl) 2,3,4,6-tetra-*O*-acetyl-1-thio-β-d-galactopyranoside (**22**) [34], 3-(2,4-dioxo-3,4-ethyl dihydropyrimidine-1(2*H*)-yl) propionate (**23**), 3-(2,4-dioxo-3,4-dihydropyrimidin-1(2*H*)-yl) propanoic acid (**24**) [50], 1-[(2-hydroxyethoxy)methyl]pyrimidine-2,4(1*H*,3*H*)-dione (**25**), 1-[(2-hydroxypropoxy)methyl]pyrimidine-2,4(1*H*,3*H*)-dione (**26**) [51], 2-hydroxyethyl 3-(2,4-dioxo-3,4-dihydropyrimidin-1(2*H*)-yl)propanoate (**29**) [54], and 2-[(2,4-dioxo-3,4-dihydropyrimidin-1(2*H*)-yl)methyl]succinate (**31**) [55] were prepared according to the respective published procedures. 1,6-Anhydro-β-d-glucopyranose (**7**) and 1,6-anhydro-β-d-galactopyranose (**8**) were purchased as ready-made compounds. All used chemicals were purchased from Aldrich, Fluka, and ACROS Organics and were used without purification. Bovine milk β-1,4-galactosyltransferase I was purchased from Sigma-Aldrich. Immobilised lipase Novozym^®^ 435 was provided by Novozymes A/S, Bagsvaerd, Denmark.

### 3.2. Chemistry

#### 3.2.1. Synthesis of (5-nitro-2-pyridyl) 2,3,4-tri-*O*-benzyl-1-thio-α-d-glycopyranosides **13** and **14**

**Procedure A.** To a solution of **7** or **8** (100 mg, 0.23 mmol) in dry CH_2_Cl_2_ (5 mL) 2-mercapto-5-nitropyridine (119 mg, 0.76 mmol) and BF_3_.Et_2_O (30 µL, 0.23 mmol) were added. The resulting mixture was heated under reflux for 32–40 h. The reaction progress was monitored on TLC plate in toluene:AcOEt (4:1 *v*/*v*) solvent system. After completion, the reaction mixture was filtered through a celite pad, filtrate was diluted with CH_2_Cl_2_ (10 mL) and washed with saturated NaHCO_3_ (10 mL) and brine (10 mL), dried with anhydr. MgSO_4_, filtered, and concentrated under vacuum. The crude products **13** or **14** were purified by column chromatography. 

**Procedure B**. To a solution of **9** or **10** (466 mg, 1 mmol) in dry acetone (10 mL) 2-chloro-5-nitropyridine (174 mg, 1.1 mmol) and anhydrous K_2_CO_3_ (552 mg, 4 mmol) were added. The resulting mixture was stirred at room temperature. The reaction progress was monitored on TLC plate in toluene:AcOEt (4:1 *v*/*v*) solvent system. The resulting suspension was stirred for 2 h at room temperature. K_2_CO_3_ was filtered off and the filtrate was evaporated. The crude products **13** or **14** were purified by column chromatography (toluene:AcOEt; gradient 12:1 to 4:1 *v*/*v*).

*(5-Nitro-2-pyridyl) 2,3,4-tri-O-benzyl-1-thio-α-d-glucopyranoside* (**13**): white solid. **Procedure A**: (49 mg, 36%). **Procedure B**: (512 mg, 87%): m.p. of 113–115 °C; ${\left[\alpha \right]}_{D}^{25}$ 77.9 (c 1.0, CHCl_3_). ^1^H-NMR (400 MHz, CDCl_3_): δ 3.64 (dd, 1H, *J* = 8.9 Hz, *J* = 9.8 Hz, H-4), 3.69–3.74 (m, 2H, H-6a, H-6b), 3.81 (dd~t, 1H, *J* < 1 Hz, *J* = 9.2 Hz, H-3), 3.90 (ddd~dt, 1H, *J* = 3.2 Hz, *J* = 9.9 Hz, H-5), 3.96 (dd, 1H, *J* = 5.4 Hz, *J* = 9.6 Hz, H-2), 4.69, 4.72 (qAB, 2H, *J* = 11.4 Hz, CH_2_Ph), 4.66, 4.89 (qAB, 2H, *J* = 11.0 Hz, CH_2_Ph), 4.83, 4.99 (qAB, 2H, *J* = 10.9 Hz, CH_2_Ph), 6.67 (d, 1H, *J* = 5.4 Hz, H-1), 7.25–7.39 (m, 16H, H-Ph, H-3_pyr_), 8.27 (dd, 1H, *J* = 2.7 Hz, *J* = 8.8 Hz, H-4_pyr_), 9.25 (d, 1H, *J* = 2.6 Hz, H-6_pyr_). ^13^C-NMR (100 MHz, CDCl_3_): δ 61.91 (C-6), 72.93, 75.21, 75.71 (CH_2_Ph), 74.10 (C-5); 76.59 (C-4), 78.99 (C-2), 82.75 (C-3), 83.16 (C-1), 122.79 (C-3_pyr_), 127.75, 127.95, 127.99, 128.01, 128.03, 128.07, 128.39, 128.43, 128.47, 128.52 (C-Ph), 130.97 (C-4_pyr_), 137.26, 137.89, 138.38 (C-Ph), 141.70 (C-5_pyr_), 145.03 (C-6_pyr_), 165.31 (C-2_pyr_). HRMS (ESI) (*m*/*z*): [M + Na]^+^ calcd for C_32_H_32_N_2_NaO_7_S, 611.1828; found, 611.1804.

*(5-Nitro-2-pyridyl) 2,3,4-tri-O-benzyl-1-thio-α-d-galactopyranoside* (**14**): white solid. **Procedure A**: (16 mg, 12%). **Procedure B**: (476 mg, 81%): m.p. of 93–94 °C; ${\left[\alpha \right]}_{D}^{25}$ 43.6 (c 1.0, CHCl_3_). ^1^H-NMR (400 MHz, CDCl_3_): δ 3.53 (ddd, 1H, *J* = 5.2 Hz, *J* = 9.2 Hz, *J* = 11.6 Hz, H-6a), 3.77 (dd, 1H, *J* = 2.9 Hz, *J* = 9.0 Hz, H-3), 3.84 (m, 1H, H-6b), 3.96 (dd, 1H, *J* < 1 Hz, J = 2.5 Hz, H-4), 4.03 (ddd, 1H, *J* = 2.0 Hz, *J* = 5.2 Hz, *J* = 7.1 Hz, H-5), 4.38 (dd, 1H, *J* = 4.9 Hz, *J* = 9.0 Hz, H-2), 4.72 (s, 2H, CH_2_Ph); 4.76, 4.84 (qAB, 2H, *J* = 11.8 Hz, CH_2_Ph), 4.64, 4.91 (qAB, 2H, *J* = 11.6 Hz, CH_2_Ph), 6.61 (d, 1H, *J* = 4.9 Hz, H-1), 7.25–7.38 (m, 16H, H-Ph, H-3_pyr_), 8.25 (dd, 1H, *J* = 2.7 Hz, *J* = 8.8 Hz, H-4_pyr_), 9.23 (dd, 1H, *J* = 0.7 Hz, *J* = 2.7 Hz, H-6_pyr_). ^13^C-NMR (100 MHz, CDCl_3_): δ 61.26 (C-6); 73.07, 73.99, 74.08 (CH_2_Ph), 73.70 (C-5), 74.16 (C-4); 76.11 (C-2), 78.86 (C-3), 82.67 (C-1), 122.50 (C-3_pyr_), 127.64, 127.80, 127.90, 127.93, 128.04, 128.32, 128.39, 128.48, 128.51 (C-Ph), 130.91 (C-4_pyr_), 137.54, 137.94, 138.21 (C-Ph), 141.57 (C-5_pyr_), 145.05 (C-6_pyr_), 165.96 (C-2_pyr_). HRMS (ESI) (*m*/*z*): [M + Na]^+^ calcd for C_32_H_32_N_2_NaO_7_S, 611.1828; found, 611.1840.

#### 3.2.2. Synthesis of (5-amino-2-pyridyl) *O*-benzyl-1-thio-α-d-glycosides **17**–**20**

**General procedure**: Corresponding (5-nitro-2-pyridyl) 1-thioglycoside **5**, **6**, **13**, or **14** (2.83 mmol) was dissolved in THF (56 mL). Then AcOH (1.13 mL, 20.0 mmol) and powdered iron (3.17 g, 56.60 mmol) were added. The resulting suspension was sonicated for 1 h in 50°C and MeOH (28 mL) was added. After complete consumption of the substrate (1.5 h–2 h) the solids were filtered off and the filtrate was evaporated. The residue was dissolved in toluene (100 mL), washed with water (3 × 100 mL) and brine (1 × 50 mL). The organic layer was dried over MgSO_4_.

*(5-Amino-2-pyridyl) 2,3,4,6-tetra-O-benzyl-1-thio-α-d-glucopyranoside* (**17**): The residue was purified by column chromatography with toluene:EtOAc solvents system (16:1 to 6:1 (*v*/*v*)) to give an orange syrup (1.596 g, 87%): ${\left[\alpha \right]}_{D}^{20}$ 20.2 (c 1.0, CHCl_3_). ^1^H-NMR (400 MHz, CDCl_3_): δ 3.64 (bs, 2H, NH_2_), 3.57 (dd, 1H, *J* = 1.8 Hz, *J* = 10.8 Hz, H-6a), 3.66 (dd~t, 1H, *J* = 9.4 Hz, H-4), 3.73 (dd, 1H, *J* = 3.9 Hz, *J* = 10.8 Hz, H-6b), 3.86 (dd~t, 1H, *J* = 9.2 Hz, H-3), 3.94 (dd, 1H, *J* = 5.3 Hz, *J* = 9.6 Hz, H-2), 4.23 (dd, 1H, *J* = 1.9 Hz, *J* = 3.7 Hz, *J* = 9.9 Hz, H-5), 4.37, 4.53 (qAB, 2H, *J* = 12.1 Hz, CH_2_Ph), 4.63, 4.83 (qAB, 2H, *J* = 11.6 Hz, CH_2_Ph), 4.49, 4.84 (qAB, 2H, *J* = 11.0 Hz, CH_2_Ph), 4.78, 4.99 (qAB, 2H, *J* = 10.9 Hz, CH_2_Ph), 6.28 (d, 1H, *J* = 5.3 Hz, H-1), 6.80 (dd, 1H, *J* = 2.9 Hz, *J* = 8.4 Hz, H-4_pyr_), 7.12–7.39 (m, 21H, H-Ph, H-3_pyr_), 8.01 (d, 1H, *J* = 2.9 Hz, H-6_pyr_). ^13^C-NMR (100 MHz, CDCl_3_): δ 68.66 (C-6), 71.89 (C-5), 72.01, 73.27, 75.02, 75.65 (CH_2_Ph), 77.41 (C-4); 79.36 (C-2), 82.80 (C-3), 84.92 (C-1), 122.85 (C-3_pyr_), 126.39 (C-4_pyr_), 127.51, 127.52, 127.60, 127.79, 127.82, 127.99, 128.09, 128.25, 128.32 (C-Ph), 137.62 (C-6_pyr_), 137.80, 138.06, 138.33, 138.76 (C-Ph), 140.78 (C-5_pyr_), 144.03 (C-2_pyr_). HRMS (ESI) (*m*/*z*): [M + Na]^+^ calcd for C_39_H_40_N_2_NaO_5_S, 671.2556; found, 671.2515.

*(5-Amino-2-pyridyl) 2,3,4,6-tetra-O-benzyl-1-thio-α-d-galactopyranoside* (**18**): The residue was purified by column chromatography with toluene:EtOAc solvents system (16:1 to 6:1 [*v*/*v*]) to give an orange syrup (1.431 g, 78%): ${\left[\alpha \right]}_{D}^{26}$ 79.1 (c 0.8, CHCl_3_). ^1^H-NMR (400 MHz, CDCl_3_): δ 3.55 (bs, 2H, NH_2_); 3.46 (dd, 1H, *J* = 6.1 Hz, *J* = 9.6 Hz, H-6a), 3.55 (dd, 1H, *J* = 6.7 Hz, *J* = 9.6 Hz, H-6b), 3.82 (dd, 1H, *J* = 2.9 Hz, *J* = 9.9 Hz, H-3), 3.98 (dd, 1H, *J* = 1.1 Hz, *J* = 2.8 Hz, H-4), 4.29–4.42 (m, 4H, H-2, H-5, CH_2_Ph), 4.66, 4.83 (qAB, 2H, *J* = 11.6 Hz, CH_2_Ph), 4.72, 4.86 (qAB, 2H, *J* = 11.9 Hz, CH_2_Ph), 4.57, 4.95 (qAB, 2H, *J* = 11.6 Hz, CH_2_Ph), 6.25 (d, 1H, *J* = 5.4 Hz, H-1), 6.73 (dd, 1H, *J* = 2.9 Hz, *J* = 8.4 Hz, H-4_pyr_), 7.12–7.40 (m, 21H, H-Ph, H-3_pyr_), 7.97 (dd, 1H, *J* = 0.5 Hz, *J* = 2.9 Hz, H-6_pyr_). ^13^C-NMR (100 MHz, CDCl_3_): δ 68.76 (C-6); 70.86 (C-5), 72.21, 73.16, 73.37, 74.74 (CH_2_Ph), 75.15 (C-4), 76.22 (C-2), 79.57 (C-3), 85.53 (C-1), 122.81 (C-3_pyr_), 126.55 (C-4_pyr_), 127.43, 127.46, 127.47, 127.49, 127.66, 127.88, 128.04, 128.16, 128.20, 128.25, 128.30 (C-Ph), 137.53 (C-6_pyr_), 138.14, 138.17, 138.68, 138.74 (C-Ph), 140.73 (C-5_pyr_), 144.27 (C-2_pyr_). HRMS (ESI) (*m*/*z*): [M + Na]^+^ calcd for C_39_H_40_N_2_NaO_5_S, 671.2556; found, 671.2498.

*(5-Amino-2-pyridyl) 2,3,4-tri-O-benzyl-1-thio-α-d-glucopyranoside* (**19**): The residue was purified by column chromatography with toluene:EtOAc solvents system (1:1 to 1:4 [*v*/*v*]) to give an orange syrup (1.042 g, 66%): ${\left[\alpha \right]}_{D}^{26}$ 176.0 (c 1.0, CHCl_3_). ^1^H-NMR (400 MHz, CDCl_3_): δ 3.54 (m, 1H, H-4), 3.65 (dd, 1H, *J* = 4.9 Hz, *J* = 11.8 Hz, H-6a), 3.72 (bs, 2H, NH_2_), 3.75 (d, *J* = 2.4 Hz, 1H, H-6b), 3.84–3.93 (m, 2H, H-2, H-3), 4.17 (ddd, 1H, *J* = 2.7 Hz, *J* = 4.8 Hz, *J* = 9.9 Hz, H-5), 4.64, 4.82 (qAB, 2H, *J* = 11.6 Hz, CH_2_Ph), 4.63, 4.87 (qAB, 2H, *J* = 11.0 Hz, CH_2_Ph), 4.80, 4.98 (qAB, 2H, *J* = 10.9 Hz, CH_2_Ph), 6.23 (d, 1H, *J* = 4.7 Hz, H-1), 6.89 (dd, 1H, *J* = 2.9 Hz, *J* = 8.4 Hz, H-4_pyr_), 7.16 (d, 1H, *J* = 7.2 Hz, H-3_pyr_); 7.25–7.40 (m, 15H, H-Ph), 8.02 (d, 1H, *J* = 2.9 Hz, H-6_pyr_). ^13^C-NMR (100 MHz, CDCl_3_): δ 61.95 (C-6), 72.58 (C-5), 72.16, 75.03, 75.63 (CH_2_Ph), 77.32 (C-4), 79.49 (C-2), 82.68 (C-3), 84.51 (C-1), 122.86 (C-4_pyr_), 126.74 (C-3_pyr_), 127.57, 127.76, 127.78, 127.81, 127.97, 127.98, 128.09, 128.34, 128.36, 128.43 (C-Ph), 137.69, 138.13, 138.67 (C-Ph), 137.69 (C-6_pyr_), 141.39 (C-5_pyr_), 143.49 (C-2_pyr_). HRMS (ESI) (*m*/*z*): [M + Na]^+^ calcd for C_32_H_34_N_2_NaO_5_S, 581.2086; found, 581.2094.

*(5-Amino-2-pyridyl) 2,3,4-tri-O-benzyl-1-thio-α-d-galactopyranoside* (**20**): The residue was purified by column chromatography with toluene:EtOAc solvents system (4:1 to 1:4 [*v*/*v*]) to give an orange syrup (1.216 g, 77%): ${\left[\alpha \right]}_{D}^{26}$ 124.2 (c 1.0, CHCl_3_). ^1^H-NMR (400 MHz, CDCl_3_): δ 3.48 (dd, 1H, *J* = 4.1 Hz, *J* = 11.6 Hz, H-6a), 3.65 (bs, 2H, NH_2_), 3.84 (dd, 1H, *J* = 3.0 Hz, *J* = 8.8 Hz, H-3), 3.89 (dd, 1H, *J* = 8.0 Hz, *J* = 11.5 Hz, H-6b), 3.92 (dd~t, *J* = 2.5 Hz, H-4), 4.26–4.35 (m, 2H, H-2, H-5), 4.61, 4.80 (qAB, 2H, *J* = 11.7 Hz, CH_2_Ph), 4.72, 4.82 (qAB, 2H, *J* = 11.9 Hz, CH_2_Ph), 4.67, 4.87 (qAB, 2H, *J* = 11.7 Hz, CH_2_Ph), 6.11 (d, 1H, *J* = 4.8 Hz, H-1), 6.87 (dd, 1H, *J* = 2.6 Hz, *J* = 8.4 Hz, H-4_pyr_), 7.17 (d, 1H, *J* = 8.4 Hz, H-3_pyr_), 7.26–7.41 (m, 15H, H-Ph), 7.98 (d, 1H, *J* = 2.6 Hz, H-6_pyr_). ^13^C-NMR (100 MHz, CDCl_3_): δ 61.76 (C-6), 72.52, 73.65, 73.97 (CH_2_Ph), 72.88 (C-5), 74.85 (C-4), 76.83 (C-2), 78.75 (C-3), 83.85 (C-1), 122.82 (C-3_pyr_), 126.97 (C-4_pyr_), 127.61, 127.64, 127.67, 127.82, 127.97, 128.21, 128.30, 128.39 (C-Ph), 137.67 (C-5_pyr_), 138.02, 138.19, 138.54 (C-Ph), 141.10 (C-6_pyr_), 143.73 (C-2_pyr_). HRMS (ESI) (*m*/*z*): [M + Na]^+^ calcd for C_32_H_34_N_2_NaO_5_S, 581.2086; found, 581.2095.

#### 3.2.3. Synthesis of Acyclic Uridine Derivatives 

General procedure for the synthesis of 2-[(2,4-dioxo-3,4-dihydropyrimydine-1(2H)-yl) methoxy] acetic acid (**27**), 2-[(2,4-dioxo-3,4-dihydropyrimydine-1(2*H*)-yl)methoxy]propanoic acid (**28**), and ([3-(2,4-dioxo-3,4-dihydropyrimydin-1(2*H*)-ylo)propanoil]oxy)acetic acid (**30**). Corresponding substrate **25**, **26**, or **29** (1.70 mmol) was dissolved in MeCN:H_2_O solvent system (1:1 (*v*/*v*)) (20 mL). TEMPO (106 mg, 0.34 mmol) and BAIB (747 mg, 3.77 mmol) were added. The resulting mixture was stirred for 120 h at room temperature. After complete consumption of the substrate solution was evaporated. To the residue Et_2_O (5 mL) was added, stirred for 5 min and the supernatant was decanted. The operation was repeated three times. The crude product was crystallised from ethanol.

*2-[(2,4-Dioxo-3,4-dihydropyrimidin-1(2H)-yl)methoxy]acetic acid* (**27**) white solid (284 mg, 78%): m.p. 179–180 °C. ^1^H-NMR (400 MHz, DMSO): δ 4.14 (s, 2H, CH_2_COO), 5.14 (s, 2H, CH_2_N), 5.60 (dd, 1H, *J* = 7.9 Hz, *J* = 2.1 Hz, H-5_ur_); 7.71 (d, 1H, *J* = 7.9 Hz, H-6_ur_), 11.30 (bs, 1H, NH), 12.73 (bs, 1H, OH). ^13^C-NMR (100 MHz, DMSO): δ 66.11 (CH_2_O), 76.53 (CH_2_N), 101.68 (C-5_ur_), 145.00 (C-6_ur_), 151.22 (C-2_ur_), 163.15 (C-4_ur_), 171.06 (COO). HRMS (ESI) (*m*/*z*): [M − H]^+^ calcd for C_7_H_9_N_2_O_5_, 201.0511; found, 201.0512.

*2-[(2,4-Dioxo-3,4-dihydropyrimidin-1(2H)-yl)methoxy]propanoic acid* (**28**) white solid (279 mg, 82%): m.p. 164–166 °C. ^1^H-NMR (400 MHz, DMSO): δ 2.45 (t, 2H, *J* = 6.2 Hz, CH_2_COO), 3.68 (t, 2H, *J* = 6.2 Hz, CH_2_O), 5.06 (s, 2H, CH_2_N), 5.60 (d, 1H, *J* = 7.9 Hz, H-5_ur_), 7.68 (d, 1H, *J* = 7.9 Hz, H-6_ur_), 11.31 (bs, 1H, NH). ^13^C-NMR (100 MHz, DMSO): δ 34.46 (CH_2_COO), 64.53 (CH_2_O), 76.22 (CH_2_N), 101.54 (C-5_ur_), 144.86 (C-6_ur_), 151.05 (C-2_ur_), 163.54 (C-4_ur_), 172.26 (COO). HRMS (ESI) (*m*/*z*): [M − H]^+^ calcd for C_8_H_11_N_2_O_5_, 215.0668; found, 215.0667.

*([3-(2,4-Dioxo-3,4-dihydropyrimydin-1(2H)-ylo)propanoil]oxy)acetic acid* (**30**) white solid (123 mg, 30%): m.p. 176–178 °C. ^1^H-NMR (400 MHz, DMSO): δ 2.78 (t, 2H, *J* = 6.8 Hz, CH_2_CO), 3.90 (t, 2H, *J* = 6.8 Hz, CH_2_CO), 4.57 (s, 2H, OCH_2_CO), 5.51 (dd, 1H, *J* = 2.0 Hz, *J* = 7.6 Hz, H-5_ur_), 7.63 (d, 1H, *J* = 7.6 Hz, H-6_ur_), 11.24 (s, 1H, NH). ^13^C-NMR (100 MHz, DMSO): δ 32.23 (CH_2_), 43.89 (CH_2_N), 60.78 (CH_2_O), 100.63 (C-5_ur_), 146.04 (C-6_ur_), 150.81 (C-2_ur_), 163.69 (C-4_ur_), 168.86, 170.35 (COO). HRMS (ESI) (*m*/*z*): [M − H]^−^ calcd for C_9_H_9_N_2_O_6_, 241.0461; found, 241.0479.

##### Synthesis of 3-[(2,4-dioxo-3,4-dihydropyrimidin-1(2*H*)-yl)methyl]-methoxy-4-oxobutanoic acid (**32**)

Dimethyl 2-[(2,4-dioxo-3,4-dihydropyrimidin-1(2*H*)-yl)methyl]succinate (**31**) (135 mg, 0.50 mmol) was dissolved in distilled water (5 mL). Immobilised lipase (Novozym^®^ 435) was added. The resulting suspension was stirred for 48 h at room temperature. After complete consumption of the substrate the solids were filtered off and the filtrate was evaporated. The residue was dissolved in MeOH (5 mL), the silica gel was added, solvent was evaporated and purified by column chromatography with CHCl3:MeOH solvent system (5:1 to 2:1 [*v*/*v*]). Product **32** was obtained as white crystals (93 mg, 80%): m.p. >130 °C with decomposition; ^1^H-NMR (400 MHz, DMSO): δ 2.30 (dd, 1H, *J* = 5.2 Hz, *J* = 16.6 Hz, CHHCOO), 2.40 (dd, 1H, *J* = 8.6 Hz, *J* = 16.6 Hz, CHHCOO), 3.03 (m, 1H, CH), 3.53 (s, 3H, CH_3_), 3.81 (dd, 1H, *J* = 6.1 Hz, *J* = 13.7 Hz, CHHN), 3.88 (dd, 1H, *J* = 8.2 Hz, *J* = 13.7 Hz, CHHN), 5.53 (d, 1H, *J* = 7.9 Hz, H-5_ur_), 7.56 (d, 1H, *J* = 7.9 Hz, H-6_ur_), 11.24 (bs, 1H, NH). ^13^C-NMR (100 MHz, DMSO): δ 36.97 (CH_2_COO), 41.71 (CH), 49.12 (CH_2_N), 51.25 (CH_3_), 100.49 (C-5_ur_), 146.05 (C-6_ur_), 150.87 (C-2_ur_), 163.65 (C-4_ur_), 172.32, 173.85 (COOH, COOCH_3_). HRMS (ESI) (*m*/*z*): [M − H]^−^ calcd for C_10_H_11_N_2_O_6_, 255.0617; found, 255.0622.

#### 3.2.4. Synthesis of Glycoconjugates **33**–**54**

General procedure. The corresponding amine derivative **17**–**22** (0.25 mmol) and acyclic uracil derivative **24**, **27**, **28**, **30**, or **32** (0.40 mmol) were dissolved in dry THF (6 mL) and MeOH (1 mL). The CDMT (70 mg, 0.40 mmol) and *N*-methylmorpholine (55 mg, 0.55 mmol) were added. The resulting mixture was microwaved in a reactor set at 50 °C for 1.5–4 h. The progress of the reaction was monitored on TLC plate in toluene:AcOEt (1:1) solvent system. After completion, the reaction mixtures were concentrated, dissolved in CH_2_Cl_2_ (50 mL), washed with water (20 mL), saturated NaHCO_3_ (20 mL), and with brine (20 mL). The organic layer was dried over MgSO_4_, the adsorbent was filtered off and the filtrate was concentrated to give crude products **33**–**54** which were purified directly by column chromatography with an appropriate solvent system as indicated.

Glycoconjugate (**33**) Starting from amine derivative **17** and uracil derivative **24**, purified by column chromatography in CHCl_3_:MeOH solvent system (100:1 do 25:1 (*v*/*v*)) to give thick syrup (75 mg, 37%): ${\left[\alpha \right]}_{D}^{25}$ 134.5 (c 1.0, CHCl_3_). ^1^H-NMR (400 MHz, CDCl_3_): δ 2.78 (m, 2H, CH_2_CO), 3.57 (dd, 1H, *J* = 1.7 Hz, *J* = 10.7 Hz, H-6a), 3.67 (dd, 1H, *J* = 8.8 Hz, *J* = 10.0 Hz, H-4), 3.72 (dd, 1H, *J* = 3.8 Hz, *J* = 10.9 Hz, H-6b), 3.81 (dd~t, 1H, *J* = 9.2 Hz, H-3), 3.92–4.01 (m, 3H, H-2, CH_2_N), 4.13 (ddd, 1H, *J* = 2.0 Hz, *J* = 3.5 Hz, *J* = 10.1 Hz, H-5), 4.35, 4.51 (qAB, 2H, *J* = 12.1 Hz, CH_2_Ph), 4.60, 4.78 (qAB, 2H, *J* = 11.3 Hz, CH_2_Ph), 4.48, 4.83 (qAB, 2H, *J* = 11.0 Hz, CH_2_Ph), 4.78, 4.97 (qAB, 2H, *J* = 10.9 Hz, CH_2_Ph), 5.42 (d, 1H, *J* = 7.9 Hz, H-5_ur_), 6.47 (d, 1H, *J* = 5.4 Hz, H-1), 7.12–7.34 (m, 22H, H-Ph, H-6_ur_, H-3_pyr_), 7.95 (dd, 1H, *J* = 2.5 Hz, *J* = 8.7 Hz, H-4_pyr_), 8.54 (d, 1H, *J* = 2.5 Hz, H-6_pyr_), 9.14 (s, 1H, NH), 10.68 (bs, 1H, NH). ^13^C-NMR (100 MHz, CDCl_3_): δ 35.48 (CH_2_CO), 46.10 (CH_2_N), 68.53 (C-6), 72.38 (C-5), 72.17, 73.21, 75.07, 75.64 (CH_2_Ph), 77.18 (C-4); 79.10 (C-2), 82.83 (C-3), 83.96 (C-1), 101.67 (C-5_ur_), 124.14 (C-3_pyr_), 127.60, 127.67, 127.77, 128.80, 127.82, 127.96, 127.98, 128.29, 128.33, 128.34 (C-Ph, C-4pyr), 132.70 (C-5_pyr_), 137.59, 137.82, 138.14, 138.59 (C-Ph), 141.47 (C-6_pyr_), 146.42 (C-6_ur_), 151.19 (C-2_pyr_), 151.43 (C-2_ur_), 164.88 (C-4_ur_), 169.06 (NHCO). HRMS (ESI) (*m*/*z*): [M + Na]^+^ calcd for C_46_H_46_N_4_NaO_8_S, 837.2934; found, 837.3016.

Glycoconjugate (**34**) Starting from amine derivative **18** and uracil derivative **24**, purified by column chromatography in CHCl_3_:MeOH solvent system (100:1 to 25:1 (*v*/*v*)) to give thick syrup (136 mg, 67%): ${\left[\alpha \right]}_{D}^{23}$ 70.3 (c 1.0, CHCl_3_). ^1^H-NMR (400 MHz, CDCl_3_): δ 2.71 (m, 2H, CH_2_CO), 3.47 (dd, 1H, *J* = 6.1 Hz, *J* = 9.5 Hz, H-6a), 3.54 (dd, 1H, *J* = 6.7 Hz, *J* = 9.6 Hz, H-6b), 3.75 (dd, 1H, *J* = 2.8 Hz, *J* = 9.9 Hz, H-3), 3.89 (m, 2H, CH_2_N), 3.96 (m, 1H, H-4), 4.28 (m, 1H, H-5), 4.29, 4.34 (qAB, 2H, *J* = 11.7 Hz, CH_2_Ph), 4.40 (dd, 1H, *J* = 5.4 Hz, *J* = 9.9 Hz, H-2), 4.64, 4.75 (qAB, 2H, *J* = 11.6 Hz, CH_2_Ph), 4.72, 4.84 (qAB, 2H, *J* = 11.9 Hz, CH_2_Ph), 4.55, 4.93 (qAB, 2H, *J* = 11.4 Hz, CH_2_Ph), 5.34 (d, 1H, *J* = 7.9 Hz, H-5_ur_), 6.44 (d, 1H, *J* = 5.4 Hz, H-1), 7.12–7.39 (m, 22H, H-Ph, H-6_ur_, H-3_pyr_), 7.92 (dd, 1H, *J* = 2.6 Hz, *J* = 8.7 Hz, H-4_pyr_), 8.50 (d, 1H, *J* = 2.6 Hz, H-6_pyr_), 9.10 (s, 1H, NH), 10.75 (bs, 1H, NH). ^13^C-NMR (100 MHz, CDCl_3_): δ 46.04 (CH_2_N), 35.44 (CH_2_CO), 68.64 (C-6), 71.38 (C-5), 72.34, 73.16, 73.31, 74.79 (CH_2_Ph), 74.88 (C-4), 75.87 (C-2); 79.66 (C-3); 84.67 (C-1), 101.63 (C-5_ur_), 124.11 (C-3_pyr_), 127.47, 127.54, 127.57, 127.64, 127.80, 128.09, 128.19, 128.22, 128.29, 128.31 (C-Ph, C-4_pyr_), 132.61 (C-5_pyr_), 137.85, 137.97, 138.50, 138.57 (C-Ph), 141.43 (C-6_pyr_), 143.46 (C-6_ur_), 151.42 (C-2_pyr_), 151.56 (C-2_ur_), 164.89 (C-4_ur_), 169.03 (NHCO). HRMS (ESI) (*m*/*z*): [M + Na]^+^ calcd for C_46_H_46_N_4_NaO_8_S, 837.2934; found, 837.2966.

Glycoconjugate (**35**) Starting from amine derivative **17** and uracil derivative **27**, purified by column chromatography in CHCl_3_:MeOH solvent system (100:1 to 25:1 (*v*/*v*)) to give thick syrup (81 mg, 39%): ${\left[\alpha \right]}_{D}^{24}$ 122.0 (c 1.0, CHCl3). 1H-NMR (400 MHz, CDCl_3_): δ 3.57 (dd, 1H, *J* = 1.9 Hz, *J* = 10.8 Hz, H-6a), 3.65–3.75 (m, 2H, H-6b, H-4), 3.82 (dd~t, 1H, J = 9.2 Hz, H-3), 3.97 (dd, 1H, *J* = 5.4 Hz, *J* = 9.6 Hz, H-2), 4.13 (ddd, 1H, *J* = 1.9 Hz, *J* = 3.5 Hz, *J* = 10.1 Hz, H-5), 4.21 (s, 2H, CH_2_N), 4.37, 4.52 (qAB, 2H, *J* = 12.0 Hz, CH_2_Ph), 4.64, 4.77 (qAB, 2H, *J* = 11.6 Hz, CH_2_Ph), 4.49, 4.83 (qAB, 2H, *J* = 10.8 Hz, CH_2_Ph), 4.79, 4.98 (qAB, 2H, *J* = 10.9 Hz, CH_2_Ph), 5.77 (d, 1H, *J* = 7.9 Hz, H-5_ur_), 6.51 (d, 1H, *J* = 5.4 Hz, H-1), 7.13–7.34 (m, 22H, H-Ph, H-6_ur_, H-3_pyr_), 8.05 (dd, 1H, *J* = 2.6 Hz, *J* = 8.7 Hz, H-4_pyr_), 8.62 (d, 1H, *J* = 2.6 Hz, H-6_pyr_), 8.85 (s, 1H, NH), 9.30 (bs, 1H, NH). ^13^C-NMR (100 MHz, CDCl_3_): δ 68.02 (CH_2_N), 68.51 (C-6), 72.43 (C-5), 72.25, 73.27, 75.07, 75.65 (CH_2_Ph), 77.18 (C-4), 78.02 (CH_2_O), 79.13 (C-2), 82.87 (C-3), 83.91 (C-1), 103.61 (C-5_ur_), 124.21 (C-3_pyr_), 127.57, 127.61, 127.66, 128.78, 127.83, 127.84, 127.97, 128.03, 128.16, 128.29, 128.33, 128.35 (C-Ph, C-4_pyr_), 131.78 (C-5_pyr_), 137.61, 137.85, 138.19, 138.62 (C-Ph), 141.33 (C-6_pyr_), 143.41 (C-6_ur_), 151.18 (C-2_pyr_), 151.84 (C-2_ur_), 162.94 (C-4_ur_), 166.86 (NHCO). HRMS (ESI) (*m*/*z*): [M + Na]^+^ calcd for C_46_H_46_N_4_NaO_9_S, 853.2883; found, 853.2696.

Glycoconjugate (**36**) Starting from amine derivative **18** and uracil derivative **27**, purified by column chromatography in CHCl_3_:MeOH solvent system (100:1 to 25:1 (*v*/*v*)) to give thick syrup (145 mg, 70%): ${\left[\alpha \right]}_{D}^{25}$ 96.5 (c 1.0, CHCl_3_). ^1^H-NMR (400 MHz, CDCl_3_) δ 3.46 (dd, 1H, *J* = 6.0 Hz, *J* = 9.6 Hz, H-6a), 3.55 (dd, 1H, *J* = 6.8 Hz, *J* = 9.6 Hz, H-6b), 3.77 (dd, 1H, *J* = 2.8 Hz, *J* = 10.0 Hz, H-3), 3.98 (dd, 1H, *J* = 1.0 Hz, *J* = 2.8 Hz, H-4), 4.18 (s, 2H, CH_2_N); 4.29 (dd~t, 1H, *J* = 6.7 Hz, H-5), 4.30, 4.36 (qAB, 2H, *J* = 11.7 Hz, CH_2_Ph), 4.42 (dd, 1H, *J* = 5.4 Hz, *J* = 10.0 Hz, H-2), 4.67, 4.78 (qAB, 2H, *J* = 11.6 Hz, CH_2_Ph); 4.73, 4.86 (qAB, 2H, *J* = 11.9 Hz, CH_2_Ph), 4.56, 4.95 (qAB, 2H, *J* = 11.4 Hz, CH_2_Ph), 5.11 (s, 2H, CH_2_O), 5.75 (d, 1H, *J* = 7.9 Hz, H-5_ur_), 6.48 (d, 1H, *J* = 5.4 Hz, H-1), 7.15–7.38 (m, 22H, H-Ph, H-6_ur_, H-3_pyr_), 8.02 (dd, 1H, *J* = 2.6 Hz, *J* = 8.7 Hz, H-4_pyr_), 8.58 (d, 1H, *J* = 2.6 Hz, H-6_pyr_), 8.82 (s, 1H, NH), 9.40 (bs, 1H, NH). ^13^C-NMR (100 MHz, CDCl_3_) δ 68.00 (CH_2_N), 68.59 (C-6), 71.40 (C-5), 72.43, 73.22, 73.34, 74.83 (CH_2_Ph), 74.94 (C-4), 75.91 (C-2), 78.00 (CH_2_O), 79.70 (C-3), 84.59 (C-1), 103.54 (C-5_ur_), 124.18 (C-3_pyr_), 127.49, 127.55, 127.57, 127.60, 127.72, 127.85, 128.10, 128.20, 128.24, 128.30, 128.33 (C-Ph, C-4_pyr_), 131.69 (C-5_pyr_), 137.88, 138.00, 138.54, 138.60 (C-Ph), 141.24 (C-6_pyr_), 143.46 (C-6_ur_), 151.14 (C-2_pyr_), 152.26 (C-2_ur_)_;_ 163.07 (C-4_ur_), 166.86 (NHCO). HRMS (ESI) (*m*/*z*): [M + Na]^+^ calcd for C_46_H_46_N_4_NaO_9_S, 853.2883; found, 853.2769.

Glycoconjugate (**37**) Starting from amine derivative **17** and uracil derivative **28**, purified by column chromatography in CHCl_3_:MeOH solvent system (100:1 to 25:1 (*v*/*v*)) to give thick syrup (103 mg, 49%): ${\left[\alpha \right]}_{D}^{24}$ 133.4 (c 1.0, CHCl3). ^1^H-NMR (400 MHz, CDCl_3_): δ 2.66 (t, 2H, *J* = 5.7 Hz, CH_2_CO), 3.56 (dd, 1H, *J* = 1.8 Hz, *J* = 10.8 Hz, H-6a), 3.64–3.75 (m, 2H, H-6b, H-4), 3.82 (dd~t, 1H, *J* = 9.2 Hz, H-3), 3.88 (t, 2H, *J* = 5.8 Hz, CH_2_O), 3.95 (dd, 1H, *J* = 5.4 Hz, *J* = 9.5 Hz, H-2), 4.14 (ddd, 1H, *J* = 1.9 Hz, *J* = 3.5 Hz, *J* = 10.0 Hz, H-5), 4.35, 4.51 (qAB, 2H, *J* = 12.0 Hz, CH_2_Ph), 4.61, 4.75 (qAB, 2H, *J* = 11.5 Hz, CH_2_Ph), 4.48, 4.83 (qAB, 2H, *J* = 10.8 Hz, CH_2_Ph), 4.78, 4.97 (qAB, 2H, *J* = 10.8 Hz, CH_2_Ph), 5.13 (s, 2H, CH_2_N), 5.77 (d, 1H, *J* = 7.9 Hz, H-5_ur_), 6.45 (d, 1H, *J* = 5.4 Hz, H-1), 7.11–7.34 (m, 22H, H-Ph, H-6_ur_, H-3_pyr_), 8.04 (dd, 1H, *J* = 2.6 Hz, *J* = 8.7 Hz, H-4_pyr_), 8.47 (d, 1H, *J* = 2.6 Hz, H-6_pyr_), 8.85 (s, 1H, NH), 9.54 (bs, 1H, NH). ^13^C-NMR (100 MHz, CDCl_3_): δ 37.29 (CH_2_CO), 65.30 (CH_2_O), 68.50 (C-6), 72.32 (C-5), 72.20, 73.27, 75.07, 75.65 (CH_2_Ph), 77.18 (C-4); 77.23 (CH_2_N), 79.13 (C-2), 82.82 (C-3), 84.04 (C-1), 103.25 (C-5_ur_), 124.36 (C-3_pyr_), 127.58, 127.62, 127.67, 128.78, 127.83, 127.86, 127.97, 128.00, 128.20, 128.29, 128.33, 128.35 (C-Ph, C-4_pyr_), 132.80 (C-5_pyr_), 137.59, 137.79, 138.17, 138.60 (C-Ph), 141.07 (C-6_pyr_), 143.53 (C-6_ur_), 151.07 (C-2_pyr_), 151.33 (C-2_ur_), 163.33 (C-4_ur_), 166.29 (NHCO). HRMS (ESI) (*m*/*z*) [M + Na]^+^ calc for C_47_H_48_N_4_NaO_9_S, 867.3040; found, 867.3086. 

Glycoconjugate (**38**) Starting from amine derivative **18** and uracil derivative **28**, purified by column chromatography in CHCl_3_:MeOH solvent system (100:1 to 25:1 (*v*/*v*)) to give thick syrup (126 mg, 60%): ${\left[\alpha \right]}_{D}^{23}$ 134.8 (c 1.0, CHCl_3_). ^1^H-NMR (400 MHz, CDCl_3_): δ 2.64 (t, 2H, *J* = 5.7 Hz, CH_2_CO), 3.45 (dd, 1H, *J* = 6.0 Hz, *J* = 9.5 Hz, H-6a), 3.54 (dd, 1H, *J* = 6.9 Hz, *J* = 9.5 Hz, H-6b), 3.77 (dd, 1H, *J* = 2.8 Hz, *J* = 10.0 Hz, H-3), 3.88 (t, 2H, *J* = 5.7 Hz, CH_2_O), 3.97 (dd, 1H, *J* = 0.9 Hz, *J* = 2.7 Hz, H-4), 4.26–4.31 (m, 2H, H-5, CHHPh), 4.35 (d, 1H, *J* = 11.8 Hz, CHHPh), 4.41 (dd, 1H, *J* = 5.4 Hz, *J* = 10.0 Hz, H-2), 4.65, 4.76 (qAB, 2H, *J* = 11.6 Hz, CH_2_Ph), 4.72, 4.84 (qAB, 2H, *J* = 11.8 Hz, CH_2_Ph), 4.55, 4.94 (qAB, 2H, *J* = 11.4 Hz, CH_2_Ph), 5.09 (s, 2H, CH_2_N), 5.68 (d, 1H, *J* = 7.9 Hz, H-5_ur_), 6.43 (d, 1H, *J* = 5.4 Hz, H-1), 7.14–7.38 (m, 22H, H-Ph, H-6_ur_, H-3_pyr_), 8.01 (dd, 1H, *J* = 2.6 Hz, *J* = 8.7 Hz, H-4_pyr_), 8.58 (d, 1H, *J* = 2.6 Hz, H-6_pyr_), 8.82 (s, 1H, NH), 9.44 (bs, 1H, NH). ^13^C-NMR (100 MHz, CDCl_3_): δ 37.30 (CH_2_CO), 65.28 (CH_2_O), 68.59 (C-6), 71.30 (C-5), 72.38, 73.22, 73.35, 74.83 (CH_2_Ph), 74.93 (C-4), 75.91 (C-2), 77.20 (CH_2_N), 79.67 (C-3), 84.74 (C-1), 103.20 (C-5_ur_), 124.39 (C-3_pyr_), 127.49, 127.55, 127.56, 127.61, 127.73, 127.83, 128.10, 128.20, 128.24, 128.30, 128.32 (C-Ph, C-4_pyr_), 132.70 (C-5_pyr_), 137.87, 137.99, 138.55, 138.60 (C-Ph), 141.00 (C-6_pyr_), 143.49 (C-6_ur_), 152.25 (C-2_ur_), 151.48 (C-2_pyr_), 163.28 (C-4_ur_), 169.25 (NHCO). HRMS (ESI) (*m*/*z*): [M + Na]^+^ calcd for C_47_H_48_N_4_NaO_9_S, 867.3040; found, 867.3057.

Glycoconjugate (**39**) Starting from amine derivative **17** and uracil derivative **30**, purified by column chromatography in CHCl_3_:MeOH solvent system (100:1 to 25:1 (*v*/*v*)) to give thick syrup (85 mg, 39%): ${\left[\alpha \right]}_{D}^{23}$ 3.1 (c 1.0, CHCl_3_). ^1^H-NMR (400 MHz, CDCl_3_): δ 2.90 (dd~t, 2H, *J* = 5.8 Hz, *J* = 0.7 Hz, CH_2_CO), 3.57 (dd, 1H, *J* = 1.9 Hz, *J* = 10.8 Hz, H-6a), 3.68 (dd, 1H, *J* = 9.1 Hz, *J* = 9.9 Hz, H-4), 3.73 (dd, 1H, *J* = 3.9 Hz, *J* = 10.9 Hz, H-6b), 3.82 (dd~t, 1H, *J* = 9.2 Hz, H-3), 3.96 (dd, 1H, *J* = 5.4 Hz, *J* = 9.5 Hz, H-2), 4.06 (dd~t, 2H, *J* = 6.0 Hz, CH_2_N), 4.13 (ddd, 1H, *J* = 1.9 Hz, *J* = 3.7 Hz, *J* = 10.1 Hz, H-5), 4.37, 4.52 (qAB, 2H, *J* = 11.9 Hz, CH_2_Ph), 4.64, 4.77 (qAB, 2H, *J* = 11.6 Hz, CH_2_Ph), 4.48, 4.84 (qAB, 2H, *J* = 10.5 Hz, CH_2_Ph), 4.79, 4.98 (qAB, 2H, *J* = 10.9 Hz, CH_2_Ph), 4.74 (s, 2H, CH_2_O), 5.69 (d, 1H, *J* = 7.9 Hz, H-5_ur_), 6.50 (d, 1H, *J* = 5.4 Hz, H-1), 7.11–7.35 (m, 22H, H-Ph, H-6_ur_, H-3_pyr_), 8.02 (dd, 1H, *J* = 2.5 Hz, *J* = 8.7 Hz, H-4_pyr_), 8.48 (s, 1H, NH), 8.57 (d, 1H, *J* = 2.5 Hz, H-6_pyr_), 9.07 (bs, 1H, NH). ^13^C-NMR (100 MHz, CDCl_3_): δ 33.56 (CH_2_CO), 45.09 (CH_2_N), 63.60 (CH_2_O), 68.55 (C-6), 72.40 (C-5), 72.28, 73.29, 75.10, 75.68 (CH_2_Ph), 77.20 (C-4), 79.13 (C-2), 82.87 (C-3), 83.88 (C-1), 102.68 (C-5_ur_), 124.18 (C-3_pyr_), 127.60, 127.63, 127.69, 127.81, 127.85, 127.86, 128.04, 128.31, 128.35, 128.38, 128.69 (C-Ph, C-4_pyr_), 131.62 (C-5_pyr_), 137.61, 137.83, 138.18, 138.62 (C-Ph), 141.62 (C-6_pyr_), 144.90 (C-6_ur_), 151.03 (C-2_pyr_), 152.13 (C-2_ur_), 163.20 (C-4_ur_), 165.28 (NHCO), 169.83 (COO). HRMS (ESI) (*m*/*z*): [M + Na]^+^ calcd for C_48_H_48_N_4_NaO_10_S, 895.2989; found, 895.2917. 

Glycoconjugate (**40**) Starting from amine derivative **18** and uracil derivative **30**, purified by column chromatography in CHCl_3_:MeOH solvent system (100:1 to 25:1 (*v*/*v*)) to give thick syrup (105 mg, 48%): ${\left[\alpha \right]}_{D}^{25}$ 103.2 (c 1.0, CHCl3). ^1^H-NMR (400 MHz, CDCl_3_): δ 2.82 (dd~t, 2H, *J* = 5.8 Hz, CH_2_CO), 3.47 (dd, 1H, *J* = 6.2 Hz, *J* = 9.6 Hz, H-6a), 3.53 (dd, 1H, *J* = 6.6 Hz, *J* = 9.6 Hz, H-6b), 3.76 (dd, 1H, *J* = 2.9 Hz, *J* = 10.0 Hz, H-3), 3.91–4.01 (m, 3H, H-4, CH_2_N), 4.28 (dd~t, 1H, *J* = 6.7 Hz, H-5), 4.30, 4.36 (qAB, 2H, *J* = 11.8 Hz, CH_2_Ph), 4.41 (dd, 1H, *J* = 5.4 Hz, *J* = 10.0 Hz, H-2), 4.66, 4.77 (qAB, 2H, *J* = 11.6 Hz, CH_2_Ph), 4.73, 4.85 (qAB, 2H, *J* = 11.8 Hz, CH_2_Ph), 4.55, 4.94 (qAB, 2H, *J* = 11.4 Hz, CH_2_Ph), 4.69 (s, 2H, CH_2_O), 5.61 (d, 1H, *J* = 7.9 Hz, H-5_ur_), 6.46 (d, 1H, *J* = 5.4 Hz, H-1), 7.14–7.37 (m, 22H, H-Ph, H-6_ur_, H-3_pyr_), 7.96 (dd, 1H, *J* = 2.6 Hz, *J* = 8.7 Hz, H-4_pyr_), 8.53 (d, 1H, *J* = 2.6 Hz, H-6_pyr_), 8.76 (s, 1H, NH), 9.81 (bs, 1H, NH). ^13^C-NMR (100 MHz, CDCl_3_) δ 33.28 (CH_2_CO), 45.06 (CH_2_N), 63.47 (CH_2_O), 68.66 (C-6), 71.39 (C-5), 72.40, 73.22, 73.34, 74.83 (CH_2_Ph), 74.92 (C-4), 75.86 (C-2), 79.67 (C-3), 84.56 (C-1), 102.33 (C-5_ur_), 124.09 (C-3_pyr_), 127.48, 127.50, 127.57, 127.63, 127.73, 127.82, 128.12, 128.21, 128.24, 128.30, 128.32 (C-Ph, C-4_pyr_), 131.78 (C-5_pyr_), 137.76, 137.97, 138.49, 138.57 (C-Ph), 141.50 (C-6_pyr_), 145.26 (C-6_ur_), 151.16 (C-2_pyr_), 152.18 (C-2_ur_), 163.85 (C-4_ur_), 165.49 (NHCO), 170.09 (COO). HRMS (ESI) (*m*/*z*): [M + Na]^+^ calcd for C_48_H_48_N_4_NaO_10_S, 895.2989; found, 895.2966.

Glycoconjugate (**41**) Starting from amine derivative **17** and uracil derivative **32**, purified by column chromatography in CHCl_3_:MeOH solvent system (100:1 to 25:1 (*v*/*v*)) to give thick syrup (69 mg, 31%): ${\left[\alpha \right]}_{D}^{27}$ 123.5 (c 1.0, CHCl3). ^1^H-NMR (400 MHz, CDCl_3_): δ 2.77–2.92 (m, 2H, CH_2_CO), 3.31–3.40 (m, 1H, CH), 3.56 (dd, 1H, *J* = 1.7 Hz, *J* = 10.8 Hz, H-6a), 3.64–3.79 (m, 5H, H-6b, H-4, CH_3_), 3.82 (dd~t, 1H, *J* = 9.2 Hz, *J* = 0.8 Hz, H-3), 3.95 (dd, 1H, *J* = 5.4 Hz, *J* = 9.6 Hz, H-2), 3.98–4.08 (m, 1H, CHHN), 4.10–4.19 (m, 2H, H-5, CHHN), 4.36 4.51 (qAB, 2H, *J* = 12.0 Hz, CH_2_Ph), 4.62, 4.76 (qAB, 2H, *J* = 11.6 Hz, CH_2_Ph), 4.50, 4.83 (qAB, 2H, *J* = 11.4 Hz, CH_2_Ph), 4.78, 4.98 (qAB, 2H, *J* = 10.9 Hz, CH_2_Ph), 5.70 (d, 1H, *J* = 7.9 Hz, H-5_ur_), 6.47 (d, 1H, *J* = 5.4 Hz, H-1), 7.12–7.33 (m, 22H, H-Ph, H-6_ur_, H-3_pyr_), 7.99 (dd, 1H, *J* = 2.8 Hz, *J* = 8.7 Hz, H-4_pyr_), 8.49 (d, 1H, *J* = 2.5 Hz, H-6_pyr_), 8.81 (s, 1H, NH), 9.96 (bs, 1H, NH). ^13^C-NMR (100 MHz, CDCl_3_): δ 35.90 (CH_2_COO), 41.11 (CH), 49.47 (CH_2_N), 52.62 (CH_3_), 68.50 (C-6), 72.32 (C-5), 72.18, 73.27, 75.06, 75.65 (CH_2_Ph), 77.18 (C-4), 79.12 (C-2), 82.84 (C-3), 83.99 (C-1), 102.67 (C-5_ur_), 124.27 (C-3_pyr_), 127.57, 127.61, 127.65, 127.77, 127.82, 127.86, 127.97, 128.02, 128.12, 128.28, 128.32, 128.35 (C-Ph, C-4_pyr_), 132.73 (C-5_pyr_), 137.60, 137.80, 138.18, 138.62 (C-Ph), 141.13 (C-6_pyr_), 145.51 (C-6_ur_), 151.09 (C-2_pyr_), 152.16 (C-2_ur_), 163.67 (C-4_ur_), 168.92 (NHCO), 172.62 (COOCH_3_). HRMS (ESI) (*m*/*z*): [M + Na]^+^ calcd for C_49_H_50_N_4_NaO_10_S, 909.3145; found, 909.3187. 

Glycoconjugate (**42**): Starting from amine derivative **18** and uracil derivative **32**, purified by column chromatography in CHCl_3_:MeOH solvent system (100:1 to 25:1 [*v*/*v*]) to give thick syrup (125 mg, 56%): ${\left[\alpha \right]}_{D}^{25}$ 99.0 (c 1.0, CHCl_3_). ^1^H-NMR (400 MHz, CDCl_3_): δ 3.29–3.38 (m, 1H, CH), 3.46 (dd, 1H, *J* = 6.0 Hz, *J* = 9.6 Hz, H-6a), 3.55 (dd, 1H, *J* = 6.9 Hz, *J* = 9.6 Hz, H-6b), 3.72 (s, 3H, CH_3_), 3.77 (dd, 1H, *J* = 2.8 Hz, *J* = 10.0 Hz, H-3), 3.98 (dd, 1H, *J* = 1.1 Hz, *J* = 2.7 Hz, H-4), 4.04 (m, 1H, CHHN), 4.14 (m, 1H, CHHN), 4.28 (m, 1H, H-5), 4.29, 4.35 (qAB, 2H, *J* = 11.7 Hz, CH_2_Ph), 4.42 (dd, 1H, *J* = 5.4 Hz, *J* = 10.0 Hz, H-2), 4.66, 4.77 (qAB, 2H, *J* = 11.6 Hz, CH_2_Ph), 4.72, 4.85 (qAB, 2H, *J* = 11.8 Hz, CH_2_Ph), 4.56, 4.94 (qAB, 2H, *J* = 11.4 Hz, CH_2_Ph), 5.70 (d, 1H, *J* = 7.9 Hz, H-5_ur_), 6.44 (d, 1H, J = 5.4 Hz, H-1), 7.12–7.39 (m, 22H, H-Ph, H-6_ur_, H-3_pyr_), 7.96 (dd, 1H, *J* = 2.8 Hz, *J* = 8.7 Hz, H-4_pyr_), 8.45 (d, 1H, *J* = 2.8 Hz, H-6_pyr_), 8.53 (s, 1H, NH), 9.57 (bs, 1H, NH). ^13^C-NMR (100 MHz, CDCl_3_): δ 35.96 (CH_2_COO), 41.09 (CH), 49.49 (CH_2_N), 52.64 (CH_3_), 68.60 (C-6), 71.31 (C-5), 72.41, 73.22, 73.37, 74.82 (CH_2_Ph), 74.93 (C-4), 75.94 (C-2), 79.68 (C-3), 84.67 (C-1), 102.66 (C-5_ur_), 124.26 (C-3_pyr_), 127.50, 127.54, 127.57, 127.60, 127.73, 128.87, 128.10, 128.15, 128.20, 128.25, 128.30, 128.33 (C-Ph, C-4_pyr_), 132.45 (C-5_pyr_), 137.90, 138.00, 138.56, 138.63 (C-Ph), 141.08 (C-6_pyr_), 145.39 (C-6_ur_), 151.73 (C-2_pyr_), 151.96 (C-2_ur_), 163.46 (C-4_ur_), 168.75 (NHCO), 172.58 (COOCH_3_). HRMS (ESI) (*m*/*z*): [M + Na]^+^ calcd for C_49_H_50_N_4_NaO_10_S, 909.3145; found, 909.3117.

Glycoconjugate (**43**) Starting from amine derivative **19** and uracil derivative **24**, purified by column chromatography in CHCl_3_:MeOH solvent system (100:1 to 20:1 (*v*/*v*)) to give thick syrup (58 mg, 32%): ${\left[\alpha \right]}_{D}^{27}$ −23.1 (c 1.0, CHCl3). ^1^H-NMR (400 MHz, CDCl_3_): δ 2.65–2.85 (m, 2H, CH_2_CO), 3.57 (dd~t, 1H, *J* = 9.2 Hz, H-4), 3.69 (m, 2H, H-6a, H-6b), 3.81 (dd~t, 1H, *J* = 9.0 Hz, H-3), 3.89 (dd, 1H, *J* = 5.3 Hz, *J* = 9.4 Hz, H-2), 3.95–4.07 (m, 3H, CH_2_N, H-5), 4.61, 4.73 (qAB, 2H, *J* = 11.6 Hz, CH_2_Ph), 4.63, 4.86 (qAB, 2H, *J* = 10.9 Hz, CH_2_Ph), 4.79, 4.96 (qAB, 2H, *J* = 11.0 Hz, CH_2_Ph), 5.45 (d, 1H, *J* = 7.7 Hz, H-5_ur_), 6.44 (d, 1H, *J* = 5.2 Hz, H-1), 7.18–7.34 (m, 17H, H-Ph, H-6_ur_, H-3_pyr_), 8.00 (dd, 1H, *J* = 2.4 Hz, *J* = 8.6 Hz, H-4_pyr_), 8.52 (d, 1H, *J* = 2.4 Hz, H-6_pyr_), 9.42 (s, 1H, NH), 11.03 (bs, 1H, NH). ^13^C-NMR (100 MHz, CDCl_3_): δ 35.65 (CH_2_CO), 46.29 (CH_2_N), 61.47 (C-6), 73.35 (C-5), 72.27, 75.08, 75.56 (CH_2_Ph), 77.05 (C-4), 79.12 (C-2), 82.64 (C-3), 83.48 (C-1), 101.62 (C-5_ur_), 124.50 (C-3_pyr_), 127.60, 127.80, 127.83, 127.91, 127.98, 128.01, 128.34, 128.36, 128.44, 128.53 (C-Ph, C-4_pyr_), 132.68 (C-5_pyr_), 137.55, 138.06, 138.56 (C-Ph), 141.63 (C-6_pyr_), 146.56 (C-6_ur_), 150.99 (C-2_pyr_), 151.45 (C-2_ur_), 165.31 (C-4_ur_), 169.58 (NHCO). HRMS (ESI) (*m*/*z*): [M + Na]^+^ calcd for C_39_H_40_N_4_NaO_8_S, 747.2465; found, 747.2447. 

Glycoconjugate (**44**) Starting from amine derivative **20** and uracil derivative **24**, purified by column chromatography in CHCl_3_:MeOH solvent system (100:1 to 20:1 (*v*/*v*)) to give thick syrup (45 mg, 25%): ${\left[\alpha \right]}_{D}^{27}$ 48.0 (c 0.5, CHCl3). ^1^H-NMR (400 MHz, CDCl_3_) δ 2.64–2.78 (m, 2H, CH_2_CO), 3.50 (dd, 1H, *J* = 4.0 Hz, *J* = 11.5 Hz, H-6a), 3.78 (dd, 1H, *J* = 2.8 Hz, *J* = 8.6 Hz, H-3), 3.84–3.96 (m, 4H, H-4, H-6b, CH_2_N), 4.18 (m, 1H, H-5), 4.26 (dd, 1H, *J* = 4.5 Hz, *J* = 8.4 Hz, H-2), 4.63, 4.73 (qAB, 2H, *J* = 11.6 Hz, CH_2_Ph), 4.70, 4.79 (qAB, 2H, *J* = 11.8 Hz, CH_2_Ph), 4.57, 4.83 (qAB, 2H, *J* = 11.6 Hz, CH2Ph), 5.41 (d, 1H, *J* = 7.8 Hz, H-5_ur_), 6.27 (d, 1H, *J* = 4.5 Hz, H-1), 7.22–7.37 (m, 16H, H-Ph, H-6_ur_), 7.19 (d, 1H, *J* = 8.7 Hz, H-3_pyr_), 7.94 (dd, 1H, *J* = 2.3 Hz, *J* = 8.7 Hz, H-4_pyr_), 8.46 (d, 1H, *J* = 2.3 Hz, H-6_pyr_), 9.25 (s, 1H, NH), 10.60 (bs, 1H, NH). ^13^C-NMR (100 MHz, CDCl_3_) δ 35.41 (CH_2_CO), 46.96 (CH_2_N), 61.23 (C-6), 73.55 (C-5); 72.62, 73.61, 74.03 (CH_2_Ph), 74.60 (C-4), 76.54 (C-2), 78.53 (C-3), 82.61 (C-1), 101.71 (C-5_ur_), 124.57 (C-3_pyr_), 127.61, 127.64, 127.76, 127.83, 127.96, 128.20, 128.28, 128.33, 128.38, 128.39 (C-Ph, C-4_pyr_), 132.948 (C-5_pyr_), 137.81, 138.13, 138.41 (C-Ph), 141.41 (C-6_pyr_), 146.30 (C-6_ur_), 151.10 (C-2_pyr_), 151.45 (C-2_ur_), 164. 80 (C-4_ur_), 169.31 (NHCO). HRMS (ESI) (*m*/*z*): [M + Na]^+^ calcd for C_39_H_40_N_4_NaO_8_S, 747.2465; found, 747.2487.

Glycoconjugate (**45**) Starting from amine derivative **19** and uracil derivative **27**, purified by column chromatography in CHCl_3_: MeOH solvent system (100:1 to 20:1 (*v*/*v*)) to give thick syrup (68 mg, 37%): ${\left[\alpha \right]}_{D}^{27}$ 133.0 (c 1.0, CHCl_3_). ^1^H-NMR (400 MHz, CDCl_3_): δ 3.56 (dd, 1H, *J* = 8.7 Hz, *J* = 9.8 Hz, H-4), 3.67 (dd, 1H, *J* = 4.6 Hz, *J* = 12.0 Hz, H-6a), 3.73 (dd, 1H, *J* = 2.5 Hz, *J* = 12 Hz, H-6b), 3.85 (dd~t, 1H, *J* = 9.0 Hz, H-3), 3.92 (dd, 1H, *J* = 5.3 Hz, *J* = 9.4 Hz, H-2), 4.05 (ddd, 1H, *J* = 2.6 Hz, *J* = 4.5 Hz, *J* = 9.8 Hz, H-5), 4.22 (s, 2H, CH_2_N), 4.63, 4.77 (qAB, 2H, *J* = 11.4 Hz, CH_2_Ph), 4.64, 4.87 (qAB, 2H, *J* = 11.4 Hz, CH_2_Ph), 4.80, 4.98 (qAB, 2H, *J* = 10.9 Hz, CH_2_Ph), 5.17 (s, 2H, CH_2_O), 5.79 (d, 1H, J = 7.9 Hz, H-5_ur_), 6.48 (d, 1H, *J* = 5.3 Hz, H-1), 7.24–7.34 (m, 17H, H-Ph, H-6_ur_, H-3_pyr_), 8.05 (dd, 1H, *J* = 2.6 Hz, *J* = 8.6 Hz, H-4_pyr_), 8.65 (d, 1H, *J* = 2.6 Hz, H-6_pyr_), 8.86 (s, 1H, NH), 9.08 (bs, 1H, NH). ^13^C-NMR (100 MHz, CDCl_3_): δ 61.76 (C-6), 68.03 (CH_2_N), 73.21 (C-5), 72.34, 75.07, 75.62 (CH_2_Ph), 77.07 (C-4), 78.16 (CH_2_O), 79.25 (C-2), 82.69 (C-3), 83.48 (C-1), 103.71 (C-5_ur_), 124.60 (C-3_pyr_), 127.62, 127.85, 127.96, 128.02, 128.05, 128.17, 128.36, 128.40, 128.45 (C-Ph, C-4_pyr_), 131.89 (C-5_pyr_), 137.56, 138.06, 138.58 (C-Ph), 141.42 (C-6_pyr_), 143.30 (C-6_ur_), 151.14 (C-2_pyr_), 151.51 (C-2_ur_), 162.70 (C-4_ur_), 166.89 (NHCO). HRMS (ESI) (*m*/*z*): [M + Na] ^+^ calcd for C_39_H_40_N_4_NaO_9_S, 763.2414; found, 763.2427.

Glycoconjugate (**46**) Starting from amine derivative **20** and uracil derivative **27**, purified by column chromatography in CHCl_3_:MeOH solvent system (100:1 to 20:1 (*v*/*v*)) to give thick syrup (43 mg, 23%): ${\left[\alpha \right]}_{D}^{27}$ 51.6 (c 1.0, CHCl3). ^1^H-NMR (400 MHz, CDCl_3_): δ 3.52 (dd, 1H, *J* = 3.9 Hz, *J* = 11.7 Hz, H-6a), 3.79 (dd, 1H, *J* = 2.8 Hz, *J* = 8.6 Hz, H-3), 3.89–4.00 (m, 2H, H-4, H-6b), 4.14 (s, 2H, CH_2_N), 4.20 (m, 1H, H-5), 4.28 (dd, 1H, *J* = 4.6 Hz, *J* = 8.4 Hz, H-2), 4.64, 4.74 (qAB, 2H, *J* = 11.6 Hz, CH_2_Ph), 4.70, 4.79 (qAB, 2H, *J* = 11.8 Hz, CH_2_Ph), 4.58, 4.83 (qAB, 2H, *J* = 11.5 Hz, CH_2_Ph), 5.05 (s, 2H, CH_2_O), 5.66 (d, 1H, *J* = 7.9 Hz, H-5_ur_), 6.29 (d, 1H, *J* = 4.5 Hz, H-1), 7.17–7.41 (m, 17H, H-Ph, H-6_ur_, H-3_pyr_), 8.05 (dd, 1H, *J* = 2.4 Hz, *J* = 8.7 Hz, H-4_pyr_), 8.53 (d, 1H, *J* = 2.4 Hz, H-6_pyr_), 9.14 (s, 1H, NH), 10.02 (bs, 1H, NH). ^13^C-NMR (100 MHz, CDCl_3_): δ 68.04 (CH_2_N), 61.23 (C-6), 73.52 (C-5), 72.62, 73.64, 74.05 (CH_2_Ph), 74.67 (C-4), 76.53 (C-2), 78.07 (CH_2_O), 78.53 (C-3), 82.67 (C-1), 103.33 (C-5_ur_), 124.63 (C-3_pyr_), 127.59, 127.64, 127.75, 127.84, 127.94, 128.19, 128.28, 128.32, 128.38 (C-Ph, C-4_pyr_), 132.23 (C-5_pyr_), 137.79, 138.08, 138.38 (C-Ph), 141.31 (C-6_pyr_), 143.71 (C-6_ur_), 151.44 (C-2_pyr_), 151.54 (C-2_ur_), 163.49 (C-4_ur_), 167.54 (NHCO). HRMS (ESI) (*m*/*z*): [M + Na] ^+^ calcd for C_39_H_40_N_4_NaO_9_S, 763.2414; found, 763.2471.

Glycoconjugate (**47**) Starting from amine derivative **19** and uracil derivative **28**, purified by column chromatography in CHCl_3_:MeOH solvent system (100:1 to 20:1 (*v*/*v*)) to give thick syrup (81 mg, 43%): ${\left[\alpha \right]}_{D}^{27}$ 126.6 (c 1.0, CHCl_3_). ^1^H-NMR (400 MHz, CDCl_3_): δ 2.62 (t, 2H, *J* = 5.3 Hz, CH_2_CO), 3.49 (dd, 1H, *J* = 8.7 Hz, *J* = 9.4 Hz, H-4), 3.64 (dd, 1H, *J* = 5.3 Hz, *J* = 12.0 Hz, H-6a), 3.73 (dd, 1H, *J* = 2.1 Hz, *J* = 12 Hz, H-6b), 3.80–3.94 (m, 4H, H-2, H-3, CH_2_O), 4.08 (ddd, 1H, *J* = 2.3 Hz, *J* = 4.9 Hz, *J* = 9.5 Hz, H-5), 4.59, 4.74 (qAB, 2H, *J* = 11.6 Hz, CH_2_Ph), 4.60, 4.85 (qAB, 2H, *J* = 11.6 Hz, CH_2_Ph), 4.78, 4.95 (qAB, 2H, *J* = 11.0 Hz, CH_2_Ph), 5.07 (s, 2H, CH_2_N), 5.65 (d, 1H, *J* = 7.9 Hz, H-5_ur_), 6.39 (d, 1H, *J* = 5.2 Hz, H-1), 7.17–7.36 (m, 17H, H-Ph, H-6_ur_, H-3_pyr_), 8.00 (dd, 1H, *J* = 2.3 Hz, *J* = 8.7 Hz, H-4_pyr_), 8.49 (d, 1H, *J* = 2.3 Hz, H-6_pyr_), 8.86 (s, 1H, NH), 9.96 (bs, 1H, NH). ^13^C-NMR (100 MHz, CDCl_3_): δ 37.29 (CH_2_CO), 61.68 (C-6), 65.34 (CH_2_O), 73.23 (C-5), 72.23, 75.01, 75.57 (CH_2_Ph), 77.00 (C-4), 77.22 (CH_2_N), 79.24 (C-2), 82.54 (C-3), 83.63 (C-1), 103.04 (C-5_ur_), 124.96 (C-3_pyr_), 127.61, 127.81, 127.83, 127.93, 127.97, 128.33, 128.37, 128.42 (C-Ph, C-4_pyr_), 131.82 (C-5_pyr_), 137.54, 138.00, 138.50 (C-Ph), 141.15 (C-6_pyr_), 143.80 (C-6_ur_), 150.56 (C-2_pyr_), 151.42 (C-2_ur_), 163.78 (C-4_ur_), 169.68 (NHCO). HRMS (ESI) (*m*/*z*): [M + Na] ^+^ calcd for C_40_H_42_N_4_NaO_9_S, 777.2570; found, 777.2588. 

Glycoconjugate (**48**) Starting from amine derivative **20** and uracil derivative **28**, purified by column chromatography in CHCl_3_: MeOH solvent system (100:1 to 20:1 (*v*/*v*)) to give thick syrup (77 mg, 41%): ${\left[\alpha \right]}_{D}^{27}$ 84.2 (c 1.0, CHCl_3_). ^1^H-NMR (400 MHz, CDCl_3_): δ 2.57 (t, 2H, *J* = 5.2 Hz, CH_2_CO), 3.51 (dd, 1H, *J* = 3.8 Hz, *J* = 11.6 Hz, H-6a), 3.75–3.85 (m, 3H, H-3, CH_2_O), 3.86–3.97 (m, 2H, H-6b, H-4), 4.20 (m, 1H, H-5), 4.29 (dd, 1H, *J* = 4.7 Hz, *J* = 8.7 Hz, H-2), 4.64, 4.74 (qAB, 2H, *J* = 11.6 Hz, CH_2_Ph), 4.70, 4.79 (qAB, 2H, *J* = 12.0 Hz, CH_2_Ph), 4.57, 4.84 (qAB, 2H, *J* = 11.6 Hz, CH_2_Ph), 5.02 (s, 2H, CH_2_N), 5.59 (d, 1H, *J* = 7.9 Hz, H-5_ur_), 6.29 (d, 1H, *J* = 4.6 Hz, H-1), 7.19–7.36 (m, 17H, H-Ph, H-6_ur_, H-3_pyr_), 7.99 (dd, 1H, *J* = 2.4 Hz, *J* = 8.7 Hz, H-4_pyr_), 8.42 (d, 1H, *J* = 2.4 Hz, H-6_pyr_), 9.03 (s, 1H, NH), 10.09 (bs, 1H, NH). ^13^C-NMR (100 MHz, CDCl_3_): δ 37.23 (CH_2_CO), 55.28 (CH_2_O), 61.35 (C-6), 73.55 (C-5), 72.53, 73.46, 74.14 (CH_2_Ph), 74.76 (C-4), 76.43 (C-2), 77.17 (CH_2_N), 78.70 (C-3), 83.00 (C-1), 102.89 (C-5_ur_), 124.84 (C-3_pyr_), 127.55, 127.60, 127.71, 127.80, 127.90, 128.19, 128.29, 128.34, 128.36 (C-Ph, C-4_pyr_), 132.20 (C-5_pyr_), 137.81, 138.11, 138.39 (C-Ph); 141.04 (C-6_pyr_), 143.85 (C-6_ur_), 150.70 (C-2_pyr_), 151.38 (C-2_ur_), 163.89 (C-4_ur_), 169.80 (NHCO). HRMS (ESI) (*m*/*z*): [M + Na]^+^ calcd for C_40_H_42_N_4_NaO_9_S, 777.2570; found, 777.2578.

Glycoconjugate (**49**) Starting from amine derivative **19** and uracil derivative **30**, purified by column chromatography in CHCl_3_:MeOH solvent system (100:1 to 20:1 (*v*/*v*)) to give thick syrup (96 mg, 49%): ${\left[\alpha \right]}_{D}^{27}$ 127.4 (c 1.0, CHCl_3_). ^1^H-NMR (400 MHz, CDCl_3_): δ 2.62 (dd~t, 2H, *J* = 5.3 Hz, CH_2_CO), 3.48 (dd, 1H, *J* = 8.6 Hz, *J* = 9.8 Hz, H-4), 3.59 (dd, 1H, *J* = 5.5 Hz, *J* = 12.0 Hz, H-6a), 3.73 (dd, 1H, *J* = 2.1 Hz, *J* = 11.9 Hz, H-6b), 3.80–3.94 (m, 4H, H-2, H-3, CH_2_N), 4.08 (ddd, 1H, *J* = 2.3 Hz, *J* = 4.9 Hz, *J* = 9.5 Hz, H-5), 4.59, 4.74 (qAB, 2H, *J* = 11.6 Hz, CH_2_Ph), 4.60, 4.85 (qAB, 2H, *J* = 11.6 Hz, CH_2_Ph), 4.78, 4.96 (qAB, 2H, *J* = 11.0 Hz, CH_2_Ph), 5.07 (s, 2H, CH_2_O), 5.67 (d, 1H, *J* = 8.0 Hz, H-5_ur_), 6.51 (d, 1H, *J* = 5.4 Hz, H-1), 7.17–7.36 (m, 17H, H-Ph, H-6_ur_, H-3_pyr_), 7.93 (dd, 1H, *J* = 2.6 Hz, *J* = 8.7 Hz, H-4_pyr_), 8.43 (d, 1H, *J* = 2.3 Hz, H-6_pyr_), 8.33 (s, 1H, NH), 10.06 (bs, 1H, NH). ^13^C-NMR (100 MHz, CDCl_3_): δ 37.29 (CH_2_CO), 45.16 (CH_2_N), 61.60 (C-6), 65.34 (CH_2_O), 73.04 (C-5), 72.14, 74.95, 75.54 (CH_2_Ph), 77.20 (C-4), 79.23 (C-2), 82.51 (C-3), 83.66 (C-1), 102.88 (C-5_ur_), 124.90 (C-3_pyr_), 127.77, 127.89, 127.83, 127.91, 127.94, 128.28, 128.33, 128.37 (C-Ph, C-4_pyr_), 132.13 (C-5_pyr_), 137.52, 137.98, 138.49 (C-Ph), 141.71 (C-6_pyr_), 144.08 (C-6_ur_), 150.71 (C-2_pyr_), 151.06 (C-2_ur_), 163.48 (C-4_ur_), 168.10 (COO), 172.40 (NHCO). HRMS (ESI) (*m*/*z*): [M + Na]^+^ calcd for C_41_H_42_N_4_NaO_10_S, 805.2519; found, 805.2631. 

Glycoconjugate (**50**) Starting from amine derivative **20** and uracil derivative **30**, purified by column chromatography in CHCl_3_:MeOH solvent system (100:1 to 20:1 (*v*/*v*)) to give thick syrup (92 mg, 47%): ${\left[\alpha \right]}_{D}^{27}$ 87.5 (c 1.0, CHCl_3_). ^1^H-NMR (400 MHz, CDCl_3_): δ 2.83 (m, 2H, CH_2_CO), 3.45 (dd, 1H, *J* = 3.7 Hz, *J* = 11.9 Hz, H-6a), 3.84–4.05 (m, 5H, H-3, H-4, H-6b, CH_2_N), 4.16 (m, 1H, H-5), 4.27 (dd, 1H, *J* = 4.5 Hz, *J* = 8.4 Hz, H-2), 4.65, 4.75 (qAB, 2H, *J* = 11.6 Hz, CH_2_Ph), 4.70, 4.79 (qAB, 2H, *J* = 11.9 Hz, CH_2_Ph), 4.57, 4.83 (qAB, 2H, *J* = 11.6 Hz, CH_2_Ph), 4.66 (s, 2H, CH_2_O), 5.66 (d, 1H, *J* = 7.8 Hz, H-5_ur_), 6.24 (d, 1H, *J* = 4.4 Hz, H-1), 7.20–7.36 (m, 17H, H-Ph, H-6_ur_, H-3_pyr_), 7.95 (dd, 1H, *J* = 2.7 Hz, *J* = 8.7 Hz, H-4_pyr_), 8.40 (d, 1H, *J* = 2.6 Hz, H-6_pyr_), 8.75 (s, 1H, NH), 10.16 (bs, 1H, NH). ^13^C-NMR (100 MHz, CDCl_3_): δ 33.26 (CH_2_CO), 45.56 (CH_2_N), 61.46 (C-6), 63.45 (CH_2_O), 73.35 (C-5), 72.62, 73.55, 74.00 (CH_2_Ph), 74.57 (C-4), 76.63 (C-2), 78.40 (C-3), 82.99 (C-1), 103.19 (C-5_ur_), 125.11 (C-3_pyr_), 127.63, 127.82, 127.93, 128.18, 128.34, 128.37, 128.40, 128.32 (C-Ph, C-4_pyr_), 132.17 (C-5_pyr_), 137.81, 138.11, 138.41 (C-Ph), 141.36 (C-6_pyr_), 143.77 (C-6_ur_), 150.81 (C-2_pyr_), 151.30 (C-2_ur_), 162.97 (C-4_ur_), 168.09 (NHCO), 171.33 (COO). HRMS (ESI) (*m*/*z*): [M + Na]^+^ calcd for C_41_H_42_N_4_NaO_10_S, 805.2519; found, 805.2601.

Glycoconjugate (**51**) Starting from amine derivative **19** and uracil derivative **32**, purified by column chromatography in CHCl_3_:MeOH solvent system (100:1 to 20:1 (*v*/*v*)) to give thick syrup (60 mg, 30%): ${\left[\alpha \right]}_{D}^{19}$ 120.8 (c 1.0, CHCl_3_). ^1^H-NMR (400 MHz, CDCl3): δ 2.78 (dd, 1H, *J* = 6.5 Hz, *J* = 16.4 Hz, CH_2_CO), 2.85 (dd, 1H, *J* = 6.3 Hz, *J* = 16.3 Hz, CH_2_CO), 3.34 (m, 1H, CH), 3.57 (dd~t, 1H, *J* = 9.2 Hz, H-4), 3.65 (dd, 1H, *J* = 5.0 Hz, *J* = 12.0 Hz, H-6a), 3.68–3.76 (m, 4H, H-6b, CH_3_), 3.81 (dd~t, 1H, *J* = 8.8 Hz, H-3), 3.90 (dd, 1H, *J* = 5.2 Hz, *J* = 9.4 Hz, H-2), 4.02–4.13 (m, 3H, CH_2_N, H-5), 4.61, 4.75 (qAB, 2H, *J* = 11.8 Hz, CH_2_Ph), 4.61, 4.86 (qAB, 2H, *J* = 10.8 Hz, CH_2_Ph), 4.79, 4.96 (qAB, 2H, *J* = 10.9 Hz, CH_2_Ph), 5.67 (d, 1H, *J* = 7.9 Hz, H-5_ur_), 6.40 (d, 1H, *J* = 5.2 Hz, H-1), 7.19–7.34 (m, 17H, H-Ph, H-6_ur_, H-3_pyr_), 7.98 (dd, 1H, *J* = 2.5 Hz, *J* = 8.6 Hz, H-4_pyr_), 8.49 (d, 1H, *J* = 2.5 Hz, H-6_pyr_); 9.06 (d, 1H, NH), 10.11 (bs, 1H, NH). ^13^C-NMR (100 MHz, CDCl_3_): δ 35.80 (CH_2_COO), 40.97 (CH), 49.25 (CH_2_N), 52.63 (CH_3_), 61.71 (C-6), 73.21 (C-5), 72.25, 75.03, 75.59 (CH_2_Ph), 77.15 (C-4), 79.23 (C-2), 82.58 (C-3), 83.62 (C-1), 102.57 (C-5_ur_), 124.90 (C-3_pyr_), 127.61, 127.82, 127.84, 127.94, 127.98, 128.00, 128.34, 128.37, 128.43 (C-Ph, C-4_pyr_), 133.00 (C-5_pyr_), 137.53, 138.02, 138.53 (C-Ph), 141.15 (C-6_pyr_), 145.53 (C-6_ur_), 150.58 (C-2_pyr_), 152.02 (C-2_ur_), 163.90 (C-4_ur_), 169.09 (NHCO), 172.75 (COOCH_3_). HRMS (ESI) (*m*/*z*): [M + Na]^+^ calcd for C_42_H_44_N_4_NaO_10_S, 819.2676; found, 819.2681. 

Glycoconjugate (**52**) Starting from amine derivative **20** and uracil derivative **32**, purified by column chromatography in CHCl_3_:MeOH solvent system (100:1 to 20:1 (*v*/*v*)) to give thick syrup (46 mg, 23%): ${\left[\alpha \right]}_{D}^{27}$ 72.4 (c 1.0, CHCl_3_). ^1^H-NMR (400 MHz, CDCl_3_): δ 2.77 (dd, 1H, *J* = 6.5 Hz, *J* = 16.4 Hz, CH_2_CO), 2.84 (dd, *J* = 6.0 Hz, *J* = 16.2 Hz, CH_2_CO), 3.21 (m, 1H, CH), 3.50 (dd, 1H, *J* = 4.2 Hz, *J* = 11.7 Hz, H-6a), 3.70 (s, 3H, CH_3_), 3.80 (dd, 1H, *J* = 2.9 Hz, *J* = 8.6 Hz, H-3), 3.88–3.97 (m, 2H, H-6b, H-4), 4.02–4.07 (m, 2H, CH_2_N), 4.20 (m, 1H, H-5), 4.27 (dd, 1H, *J* = 4.5 Hz, *J* = 8.6 Hz, H-2), 4.64, 4.74 (qAB, 2H, *J* = 11.5 Hz, CH_2_Ph), 4.71, 4.80 (qAB, 2H, *J* = 11.8 Hz, CH_2_Ph), 4.58, 4.84 (qAB, 2H, *J* = 11.6 Hz, CH_2_Ph), 5.64 (d, 1H, *J* = 7.9 Hz, H-5_ur_), 6.28 (d, 1H, *J* = 4.4 Hz, H-1), 7.21 (d, 1H, *J* = 8.7 Hz, H-3_pyr_), 7.24–7.36 (m, 16H, H-Ph, H-6_ur_), 7.99 (dd, 1H, *J* = 2.5 Hz, *J* = 8.7 Hz, H-4_pyr_), 8.46 (d, 1H, *J* = 2.5 Hz, H-6_pyr_), 9.02 (s, 1H, NH), 9.88 (bs, 1H, NH). ^13^C-NMR (100 MHz, CDCl_3_): δ 35.85 (CH_2_COO), 40.97 (CH), 49.24 (CH_2_N), 52.59 (CH3), 61.28 (C-6), 73.54 (C-5), 72.63, 73.59, 74.01 (CH_2_Ph), 74.64 (C-4), 76.59 (C-2), 78.49 (C-3), 82.64 (C-1), 102.57 (C-5_ur_), 124.68 (C-3_pyr_), 127.63, 127.66, 127.76, 127.85, 127.97, 128.21, 128.33, 128.40 (C-Ph, C-4_pyr_), 132.98 (C-5_pyr_), 137.82, 138.12, 138.42 (C-Ph), 141.06 (C-6_pyr_), 145.46 (C-6_ur_), 150.99 (C-2_pyr_), 151.86 (C-2_ur_), 163.72 (C-4_ur_), 169.07 (NHCO), 172.76 (COOCH_3_). HRMS (ESI) (*m*/*z*): [M + Na]^+^ calcd for C_42_H_44_N_4_NaO_10_S, 819.2676; found, 819.2684.

Glycoconjugate (**53**) Starting from amine derivative **21** and uracil derivative **28**, purified by column chromatography in CHCl_3_:MeOH solvent system (100:1 to 25:1 (*v*/*v*)) to give thick syrup (114 mg, 70%): ${\left[\alpha \right]}_{D}^{24}$ −0.8 (c 0.5, CHCl_3_). ^1^H-NMR (400 MHz, CDCl_3_): δ 2.01, 2.02, 2.03, 2.04 (4s, 12H, CH_3_CO), 2.70 (t, 2H, *J* = 5.7 Hz, CH_2_O), 3.87 (ddd, 1H, *J* = =2.4 Hz, *J* = 4.6 Hz, *J* = 10.0 Hz, H-5), 3.95 (t, 2H, *J* = 5.8 Hz, CH_2_O), 4.11 (dd, 1H, *J* = 2.3 Hz, *J* = 12.4 Hz, H-6a), 4.25 (dd, 2H, *J* = 4.7 Hz, *J* = 12.3 Hz, H-6b), 5.11–5.24 (m, 4H, CH_2_N, H-2, H-4), 5.34 (dd~t, 1H, *J* = 9.3 Hz, H-3), 5.60 (d, 1H, *J* = 10.4 Hz, H-1), 5.75 (d, 1H, *J* = 7.9 Hz, H-5_ur_), 7.23 (dd, 1H, *J* = 0.4 Hz, *J* = 8.7 Hz, H-3_pyr_), 7.34 (d, 1H, *J* = 7.9 Hz, H-6_ur_), 8.05 (dd, 1H, *J* = 2.5 Hz, *J* = 8.7 Hz, H-4_pyr_), 8.54 (dd, 1H, *J* = 2.5 Hz, H-6_pyr_), 8.58 (s, 1H, NH), 9.80 (bs, 1H, NH). ^13^C-NMR (100 MHz, CDCl_3_): δ 20.56, 20.57, 20.64, 20.71 (CH_3_CO), 37.30 (CH_2_CO), 61.99 (C-6); 65.20 (CH_2_O), 68.27 (C-4), 69.58 (C-2), 73.98 (C-3), 75.78 (C-5), 77.24 (CH_2_N), 82.54 (C-1), 103.23 (C-5_ur_), 123.90 (C-3_pyr_), 128.23 (C-4_pyr_), 132.22 (C-5_pyr_), 141.13 (C-6_pyr_), 143.63 (C-6_ur_), 149.40 (C-2_pyr_), 151.39 (C-2_ur_), 163.54 (C-4_ur_), 169.38 (NHCO), 169.42, 169.51, 170.13, 170.73 (CH_3_CO). HRMS (ESI) (*m*/*z*): [M + Na]^+^ calcd for C_27_H_32_N_4_NaO_13_S, 675.1584; found, 675.1590.

Glycoconjugate (**54**) Starting from amine derivative **22** and uracil derivative **28**, purified by column chromatography in CHCl_3_:MeOH solvent system (100:1 to 25:1 (*v*/*v*)) to give thick syrup (106 mg, 65%): ${\left[\alpha \right]}_{D}^{24}$ 0.1 (c 0.5, CHCl_3_). ^1^H-NMR (400 MHz, CDCl_3_): δ 2.00, 2.01, 2.02, 2.16 (4s, 12H, CH_3_CO), 2.70 (t, 2H, *J* = 5.7 Hz, CH_2_CO), 3.96 (t, 2H, *J* = 5.7 Hz, CH_2_O), 4.04–4.15 (m, 3H, H-6a, H-6b, H-5), 5.15–5.23 (s, 3H, H-3, CH_2_N), 5.38 (dd~t, 1H, *J* = 10.1 Hz, H-2), 5.48 (d, 1H, *J* = 3.3 Hz, H-4), 5.60 (d, 1H, *J* = 10.3 Hz, H-1), 5.76 (d, 1H, *J* = 7.9 Hz, H-5_ur_), 7.27 (d, 1H, *J* = 8.7 Hz, H-3_pyr_), 7.31 (d, 1H, *J* = 7.9 Hz, H-6_ur_), 8.08 (dd, 1H, *J* = 2.6 Hz, *J* = 8.7 Hz, H-4_pyr_), 8.40 (s, 1H, NH), 8.51 (d, 1H, *J* = 2.6 Hz, H-6_pyr_), 9.47 (bs, 1H, NH). ^13^C-NMR (100 MHz, CDCl_3_): δ 20.58, 20.67, 20.75 (CH_3_CO), 37.35 (CH_2_CO), 61.26 (C-6), 65.19 (CH_2_O), 66.92 (C-2), 67.29 (C-4), 72.02 (C-3), 74.44 (C-5), 77.27 (CH_2_N), 83.06 (C-1), 103.33 (C-5_ur_), 123.86 (C-3_pyr_), 128.28 (C-4_pyr_), 133.09 (C-5_pyr_), 141.12 (C-6_pyr_), 143.48 (C-6_ur_), 149.76 (C-2_pyr_), 151.30 (C-2_ur_), 163.25 (C-4_ur_), 169.29 (NHCO), 169.69, 170.03, 170.24, 170.49 (CH_3_CO). HRMS (ESI) (*m*/*z*): [M + Na]^+^ calcd for C_27_H_32_N_4_NaO_13_S, 675.1584; found, 675.1592.

#### 3.2.5. Protecting Groups Removal

Debenzylation: Corresponding glycoconjugate 33–52 (0.06 mmol) was dissolved in dry CH_2_Cl_2_ (2 mL) and anhydrous FeCl_3_ (97 mg, 0.60 mmol) was added. The resulting mixture was stirred under argon. After 30 minutes the reaction mixture was diluted with CH_2_Cl_2_ (10 mL) and washed with water. Resulting emulsion was centrifuged (6000 rpm) and the supernatant was collected and evaporated. The residue was dissolved in MeOH (5 mL), the silica gel was added, and solvent was evaporated and purified by column chromatography with CHCl_3_:MeOH solvent system (10:1 to 3:1 [*v*/*v*]).

Glycoconjugate (**55**) White solid (24 mg, 93%): ${\left[\alpha \right]}_{D}^{23}$ 149.2 (c 0.3, CH_3_OH). ^1^H-NMR (400 MHz, CD_3_OD): δ 2.83 (t, 2H, *J* = 6.3 Hz, CH_2_CO), 3.39 (dd, 1H, *J* = 8.9 Hz, *J* = 9.8 Hz, H-4), 3.55 (m, 1H, H-3), 3.66–3.75 (m, 2H, H-6a, H-6b), 3.84 (dd, 1H, *J* = 5.4 Hz, *J* = 9.8 Hz, H-2), 3.93 (ddd, *J* = 3.0 Hz, *J* = 4.5 Hz, *J* = 9.8 Hz, 1H, H-5), 4.09 (t, 2H, *J* = 6.3 Hz, CH_2_N), 5.62 (d, 1H, *J* = 7.9 Hz, H-5_ur_), 6.10 (d, 1H, *J* = 5.4 Hz, H-1), 7.48 (d, 1H, *J* = 8.7 Hz, H-3_pyr_), 7.64 (d, 1H, *J* = 7.9 Hz, H-6_ur_), 7.92 (dd, 1H, *J* = 2.7 Hz, *J* = 8.7 Hz, H-4_pyr_), 8.62 (d, 1H, *J* = 2.7 Hz, H-6_pyr_). ^13^C-NMR (100 MHz, CD_3_OD): δ 36.18 (CH_2_CO), 46.36 (CH_2_N), 62.35 (C-6), 71.43 (C-4), 72.93 (C-2), 75.49 (C-5), 76.01 (C-3), 88.12 (C-1), 101.94 (C-5_ur_), 125.96 (C-3_pyr_), 129.77 (C-4_pyr_), 134.75 (C-5_pyr_), 142.11 (C-6_pyr_), 147.99 (C-6_ur_), 152.69 (C-2_ur_), 153.07 (C-2_pyr_), 166.75 (C-4_ur_), 171.33 (NHCO). HRMS (ESI) (*m*/*z*): [M + H]^+^ calcd for C_18_H_23_N_4_O_8_S, 455.1237; found, 455.1241.

Glycoconjugate (**56**) White solid (24 mg, 93%): ${\left[\alpha \right]}_{D}^{23}$ 83.6 (c 0.5, CH_3_OH). ^1^H-NMR (400 MHz, CD_3_OD): δ 2.84 (t, 2H, *J* = 6.4 Hz, CH_2_CO), 3.62–3.72 (m, 3H, H-6a, H-6b, H-3), 3.98 (dd, 1H, *J* = 1.5 Hz, *J* = 3.4 Hz, H-4), 4.10 (t, 2H, *J* = 6.4 Hz, CH_2_N), 4.16–4.20 (m, 1H, H-5), 4.23 (dd, 1H, *J* = 5.5 Hz, *J* = 10.2 Hz, H-2), 5.62 (d, 1H, *J* = 7.9 Hz, H-5_ur_), 6.13 (d, 1H, *J* = 5.5 Hz, H-1), 7.51 (d, 1H, *J* = 8.6 Hz, H-3_pyr_), 7.65 (d, 1H, *J* = 7.9 Hz, H-6_ur_), 7.92 (dd, 1H, *J* = 2.5 Hz, *J* = 8.6 Hz, H-4_pyr_), 8.63 (d, 1H, *J* = 2.5 Hz, H-6_pyr_). ^13^C-NMR (100 MHz, CD_3_OD) δ 36.21 (CH_2_CO), 46.35 (CH_2_N), 62.39 (C-6), 69.57 (C-2), 70.68 (C-4), 72.52 (C-3), 74.07 (C-5), 88.65 (C-1), 101.96 (C-5_ur_), 126.25 (C-3_pyr_), 129.79 (C-4_pyr_), 134.75 (C-5_pyr_), 142.12 (C-6_pyr_), 147.98 (C-6_ur_), 152.70 (C-2_ur_), 153.21 (C-2_pyr_), 166.76 (C-4_ur_), 171.36 (NHCO). HRMS (ESI) (*m*/*z*): [M + H]^+^ calcd for C_18_H_22_N_4_O_8_S, 455.1237; found, 455.1237.

Glycoconjugate (**57**) White solid (17 mg, 64%): ${\left[\alpha \right]}_{D}^{23}$ 145.9 (c 0.3, CH_3_OH). ^1^H-NMR (400 MHz, CD_3_OD): δ 3.40 (dd, 1H, *J* = 8.9 Hz, *J* = 9.9 Hz, H-4), 3.55 (m, 1H, H-3), 3.67–3.76 (m, 2H, H-6a, H-6b), 3.84 (dd, 1H, *J* = 5.4 Hz, *J* = 9.8 Hz, H-2), 3.93 (ddd, *J* = 3.1 Hz, *J* = 4.4 Hz, *J* = 9.8 Hz, 1H, H-5), 4.30 (s, 2H, CH_2_N), 5.30 (s, 2H, CH_2_O), 5.71 (d, 1H, *J* = 7.9 Hz, H-5_ur_), 6.13 (d, 1H, *J* = 5.4 Hz, H-1), 7.50 (dd, 1H, *J* = 8.7 Hz, H-3_pyr_), 7.73 (d, 1H, *J* = 7.9 Hz, H-6_ur_), 7.99 (dd, 1H, *J* = 2.4 Hz, *J* = 8.7 Hz, H-4_pyr_), 8.70 (d, 1H, *J* = 2.4 Hz, H-6_pyr_). ^13^C-NMR (100 MHz, CD_3_OD): δ 62.34 (C-6), 69.57 (CH_2_N), 71.42 (C-4), 72.92 (C-2), 75.52 (C-5), 76.01 (C-3), 79.07 (CH_2_O), 88.04 (C-1), 103.23 (C-5_ur_), 125.79 (C-3_pyr_), 130.25 (C-4_pyr_), 134.04 (C-5_pyr_), 142.61 (C-6_pyr_), 146.42 (C-6_ur_), 153.19 (C-2_ur_), 153.69 (C-2_pyr_), 166.34 (C-4_ur_), 170.39 (NHCO). HRMS (ESI) (*m*/*z*): [M + H]^+^ calcd for C_18_H_23_N_4_O_9_S, 471.1186; found, 471.1184. 

Glycoconjugate (**58**) White solid (19 mg, 70%): ${\left[\alpha \right]}_{D}^{23}$ 107.6 (c 0.5, CH_3_OH). ^1^H-NMR (400 MHz, CD_3_OD): δ 3.63–3.73 (m, 3H, H-6a, H-6b, H-3), 3.98 (dd, 1H, *J* = 1.2 Hz, *J* = 3.3 Hz, H-4), 4.18 (ddd, 1H, *J* = 1.5 Hz, *J* = 5.3 Hz, *J* = 6.7 Hz, H-5), 4.24 (dd, 1H, *J* = 5.5 Hz, *J* = 10.2 Hz, H-2), 4.30 (s, 2H, CH_2_N), 5.30 (s, 2H, CH_2_O), 5.72 (d, 1H, *J* = 7.9 Hz, H-5_ur_), 6.16 (d, 1H, *J* = 5.5 Hz, H-1), 7.52 (dd, 1H, *J* = 0.6 Hz, *J* = 8.7 Hz, H-3_pyr_), 7.69 (d, 1H, *J* = 7.9 Hz, H-6_ur_), 7.99 (dd, 1H, *J* = 2.5 Hz, *J* = 8.7 Hz, H-4_pyr_), 8.70 (d, 1H, *J* = 2.5 Hz, H-6_pyr_). ^13^C-NMR (100 MHz, CD_3_OD): δ 62.40 (C-6), 69.56 (CH_2_N, C-2), 70.67 (C-4), 72.53 (C-3), 74.12 (C-5), 79.08 (CH_2_O), 88.56 (C-1), 103.23 (C-5_ur_), 126.04 (C-3_pyr_), 130.23 (C-4_pyr_), 134.02 (C-5_pyr_), 142.58 (C-6_pyr_), 146.44 (C-6_ur_), 153.17 (C-2_ur_), 153.81 (C-2_pyr_), 166.32 (C-4_ur_), 170.39 (NHCO). HRMS (ESI) (*m*/*z*): [M + H]^+^ calcd for C_18_H_23_N_4_O_9_S, 471.1186; found, 471.1187.

Glycoconjugate (**59**) White solid (18 mg, 66%): ${\left[\alpha \right]}_{D}^{23}$ 67.5 (c 0.5, CH_3_OH). ^1^H-NMR (400 MHz, CD_3_OD): δ 2.65 (t, 2H, J = 5.9 Hz, CH_2_CO), 3.55 (m, 1H, H-3), 3.64–3.76 (m, 2H, H-6a, H-6b), 3.84 (dd, 1H, *J* = 5.4 Hz, *J* = 9.8 Hz, H-2), 3.93 (m, 1H, H-5), 5.17 (s, 2H, CH_2_N), 5.65 (d, 1H, *J* = 7.9 Hz, H-5_ur_), 6.10 (d, 1H, *J* = 5.4 Hz, H-1), 7.49 (dd, 1H, *J* = 0.7 Hz, *J* = 8.7 Hz, H-3_pyr_), 7.61 (d, 1H, *J* = 7.9 Hz, H-6_ur_), 7.95 (dd, 1H, *J* = 2.6 Hz, *J* = 8.9 Hz, H-4_pyr_), 8.62 (d, 1H, *J* = 2.6 Hz, H-6_pyr_). ^13^C-NMR (100 MHz, CD_3_OD): δ 38.08 (CH_2_CO), 62.38 (C-6), 66.33 (CH_2_O), 71.43 (C-4), 72.95 (C-2), 75.49 (C-5), 76.01 (C-3), 78.19 (CH_2_N), 88.15 (C-1), 103.00 (C-5_ur_), 125.99 (C-3_pyr_), 129.76 (C-4_pyr_), 134.87 (C-5_pyr_), 142.07 (C-6_pyr_), 146.21 (C-6_ur_), 152.70 (C-2_ur_), 153.22 (C-2_pyr_), 166.45 (C-4_ur_), 172.12 (NHCO). HRMS (ESI) (*m*/*z*): [M + H]^+^ calcd for C_19_H_25_N_4_O_9_S, 485.1342; found, 485.1339.

Glycoconjugate (**60**) White solid (25 mg, 93%): ${\left[\alpha \right]}_{D}^{23}$ 130.2 (c 1.0, CH_3_OH). ^1^H-NMR (400 MHz, CD_3_OD): δ 2.64 (t, 2H, *J* = 5.9 Hz, CH_2_CO), 3.64–3.73 (m, 3H, H-6a, H-6b, H-3), 3.91 (t, 2H, *J* = 5.9 Hz, CH_2_O), 3.98 (dd, 1H, *J* = 1.6 Hz, *J* = 3.3 Hz, H-4), 4.19 (ddd, 1H, *J* = 1.5 Hz, *J* = 5.3 Hz, *J* = 6.7 Hz, H-5), 4.24 (dd, 1H, *J* = 5.5 Hz, *J* = 10.2 Hz, H-2), 5.18 (s, 2H, CH_2_N), 5.65 (d, 1H, *J* = 7.9 Hz, H-5_ur_), 6.14 (d, 1H, *J* = 5.5 Hz, H-1), 7.51 (dd, 1H, *J* = 0.6 Hz, *J* = 8.7 Hz, H-3_pyr_), 7.62 (d, 1H, *J* = 7.9 Hz, H-6_ur_), 7.94 (dd, 1H, *J* = 2.6 Hz, *J* = 8.7 Hz, H-4_pyr_), 8.64 (d, 1H, *J* = 2.6 Hz, H-6_pyr_). ^13^C-NMR (100 MHz, CD_3_OD): δ 38.07 (CH_2_CO), 62.42 (C-6), 66.33 (CH_2_O), 69.57 (C-2), 70.70 (C-4), 72.51 (C-3), 74.07 (C-5), 78.20 (CH_2_N), 88.66 (C-1), 102.99 (C-5_ur_), 126.24 (C-3_pyr_), 129.70 (C-4_pyr_), 134.88 (C-5_pyr_), 142.06 (C-6_pyr_), 146.26 (C-6_ur_), 152.96 (C-2_ur_), 153.06 (C-2_pyr_), 166.44 (C-4_ur_), 172.13 (NHCO). HRMS (ESI) (*m*/*z*): [M + H]^+^ calcd for C_19_H_25_N_4_O_9_S, 485.1342, found, 485.1342.

Glycoconjugate (**61**) White solid (18 mg, 76%): ${\left[\alpha \right]}_{D}^{23}$ 90.2 (c 0.2, CH_3_OH). ^1^H-NMR (400 MHz, CD_3_OD): δ 2.93 (t, 2H, *J* = 6.3 Hz, CH_2_CO), 3.55 (dd, 1H, *J* = 8.8 Hz, *J* = 10.0 Hz, H-3), 3.67–3.75 (m, 2H, H-6a, H-6b), 3.84 (dd, 1H, *J* = 5.4 Hz, *J* = 9.8 Hz, H-2), 3.93 (ddd, 1H, *J* = 2.9 Hz, *J* = 4.6 Hz, *J* = 9.8 Hz, H-5), 4.07 (t, 2H, *J* = 6.3 Hz, CH_2_N), 4.74 (s, 2H, CH_2_O), 5.61 (d, 1H, *J* = 7.9 Hz, H-5_ur_), 6.13 (d, 1H, *J* = 5.4 Hz, H-1), 7.50 (dd, 1H, *J* = 0.6 Hz, *J* = 8.7 Hz, H-3_pyr_), 7.65 (d, 1H, *J* = 7.9 Hz, H-6_ur_), 7.95 (dd, 1H, *J* = 2.7 Hz, *J* = 8.7 Hz, H-4_pyr_), 8.60 (dd, 1H, *J* = 0.6 Hz, *J* = 2.7 Hz, H-6_pyr_). ^13^C-NMR (100 MHz, CD_3_OD): δ 33.61 (CH_2_CO), 46.04 (CH_2_N), 62.38 (C-6), 63.91 (CH_2_O), 71.44 (C-4), 72.94 (C-2), 75.54 (C-5), 76.04 (C-3), 88.03 (C-1), 101.97 (C-5_ur_), 125.84 (C-3_pyr_), 130.06 (C-4_pyr_), 134.14 (C-5_pyr_), 142.34 (C-6_pyr_), 148.01 (C-6_ur_), 152.75 (C-2_ur_), 153.65 (C-2_pyr_), 166.79 (C-4_ur_), 168.29 (COO), 172.25 (NHCO). HRMS (ESI) (*m*/*z*): [M + H]^+^ calcd for C_20_H_25_N_4_O_10_S, 513.1291, found, 513.1294.

Glycoconjugate (**62**) White solid (29 mg, 95%): ${\left[\alpha \right]}_{D}^{23}$ 105.4 (c 1.0, CH_3_OH). ^1^H-NMR (400 MHz, CD_3_OD): δ 2.73 (dd, 1H, *J* = 5.8 Hz, *J* = 16.2 Hz, CHHCO), 2.84 (dd, 1H, *J* = 7.7 Hz, *J* = 16.2 Hz, CHHCO), 3.39 (dd, 1H, *J* = 8.9 Hz, *J* = 9.9 Hz, H-4), 3.55 (m, 1H, H-3), 3.68 (s, 3H, CH_3_), 3.65–3.75 (m, 2H, H-6a, H-6b); 3.83 (dd, 1H, *J* = 5.4 Hz, *J* = 9.8 Hz, H-2), 3.93 (ddd, *J* = 3.0 Hz, *J* = 4.5 Hz, *J* = 9.8 Hz, 1H, H-5), 4.05 (ddd, 2H, *J* = 7.0 Hz, *J* = 14.0 Hz, *J* = 24.0 Hz, CH_2_N), 5.64 (d, 1H, *J* = 7.9 Hz, H-5_ur_), 6.10 (d, 1H, *J* = 5.4 Hz, H-1), 7.48 (dd, 1H, *J* = 0.5 Hz, *J* = 8.7 Hz, H-3_pyr_), 7.57 (d, 1H, *J* = 7.9 Hz, H-6_ur_), 7.91 (dd, 1H, *J* = 2.5 Hz, *J* = 8.7 Hz, H-4_pyr_), 8.61 (d, 1H, *J* = 0.5 Hz, *J* = 2.5 Hz, H-6_pyr_). ^13^C-NMR (100 MHz, CD_3_OD): δ 36.57 (CH_2_CO), 41.89 (CH), 50.62 (CH_2_N), 52.81 (CH_3_), 62.35 (C-6), 71.43 (C-4), 72.94 (C-2), 75.48 (C-5), 76.00 (C-3), 88.15 (C-1), 102.31 (C-5_ur_), 125.99 (C-3_pyr_), 129.66 (C-4_pyr_), 134.83 (C-5_pyr_), 142.02 (C-6_pyr_), 147.51 (C-6_ur_), 151.77 (C-2_ur_), 152.94 (C-2_pyr_), 166.71 (C-4_ur_), 171.31 (NHCO), 174.57 (COOCH_3_). HRMS (ESI) (*m*/*z*): [M + H] ^+^ calcd for C_21_H_27_N_4_O_10_S, 527.1448; found, 527.1450. 

Glycoconjugate (**63**) White solid (31 mg, 98%): ${\left[\alpha \right]}_{D}^{23}$ 43.5 (c 0.25, CH_3_OH). ^1^H-NMR (400 MHz, CD_3_OD): δ 2.74 (dd, 1H, *J* = 5.8 Hz, *J* = 16.2 Hz, CHHCO), 2.85 (dd, 1H, *J* = 7.7 Hz, *J* = 16.2 Hz, CHHCO), 3.37 (m, 1H, CH), 3.64–3.72 (m, 6H, H-6a, H-6b, H-3, CH_3_), 3.98 (dd, 1H, *J* = 1.2 Hz, *J* = 3.3 Hz, H-4), 4.06 (ddd, 2H, *J* = 7.0 Hz, *J* = 14.0 Hz, *J* = 20.1 Hz, CH_2_N), 4.18 (ddd, 1H, *J* = 1.3 Hz, *J* = 5.4 Hz, *J* = 6.7 Hz, 1H, H-5), 4.23 (dd, 1H, *J* = 5.5 Hz, *J* = 10.1 Hz, H-2), 5.64 (d, 1H, *J* = 7.9 Hz, H-5_ur_), 6.12 (d, 1H, *J* = 5.5 Hz, H-1), 7.51 (dd, 1H, *J* = 0.5 Hz, *J* = 8.7 Hz, H-3_pyr_), 7.59 (d, 1H, *J* = 7.9 Hz, H-6_ur_), 7.91 (dd, 1H, *J* = 2.6 Hz, *J* = 8.7Hz, H-4_pyr_), 8.61 (dd, 1H, *J* = 0.5 Hz, *J* = 2.6 Hz, H-6_pyr_). ^13^C-NMR (100 MHz, CD_3_OD): δ 36.56 (CH_2_CO), 41.88 (CH), 49.64 (CH_2_N), 52.83 (CH_3_), 62.39 (C-6), 69.58 (C-2), 70.68 (C-4), 72.52 (C-3), 74.07 (C-5), 88.68 (C-1), 101.28 (C-5_ur_), 126.28 (C-3_pyr_), 129.66 (C-4_pyr_), 134.84 (C-5_pyr_), 142.01 (C-6_pyr_), 147.59 (C-6_ur_), 152.86 (C-2_ur_), 153.07 (C-2_pyr_), 166.60 (C-4_ur_), 171.32 (NHCO), 174.55 (COOCH3). HRMS (ESI) (*m*/*z*): [M + H] ^+^ calcd for C_21_H_26_N_4_O_10_NaS, 549.1267; found, 549.1262.

Deacetylation: The corresponding glycoconjugate **53** or **54** (0.12 mmol) was dissolved in MeOH (10 mL) and 1 M MeONa in MeOH (0.2 mmol, 0.2 mL) was added. The resulting mixture was stirred at room temperature. The progress of the reaction was monitored on TLC plate in MeOH:CHCl_3_ (2:1) solvent system. After completion (30 min.), the reaction mixture was neutralised with silica gel, the solvent was evaporated purified by column chromatography with CHCl_3_: MeOH solvent system (10:1 to 2:1 [*v*/*v*]).

Glycoconjugate (**64**) Purified by column chromatography in CHCl_3_:MeOH solvent system (10:1 to 2:1 (*v*/*v*)) to give white solid (50 mg, 87%): ${\left[\alpha \right]}_{D}^{21}$ −43.4 (c 1.0, CH_3_OH). ^1^H-NMR (400 MHz, CD_3_OD): δ 2.64 (t, 2H, *J* = 5.9 Hz, CH_2_O), 3.32–3.47 (m, 4H, H-2, H-3, H4, H-5), 3.66 (dd, 1H, *J* = 5.6 Hz, *J* = 12.1 Hz, H-6a), 3.85 (dd, 1H, *J* = 2.2 Hz, *J* = 12.1 Hz, H-6b), 3.90 (t, 2H, *J* = 5.9 Hz, CH_2_O), 5.08 (d, 1H, *J* = 9.9 Hz, H-1), 5.17 (s, 2H, CH_2_N), 5.64 (d, 1H, *J* = 7.9 Hz, H-5_ur_), 7.46 (dd, 1H, *J* = 0.6 Hz, *J* = 8.7 Hz, H-3_pyr_), 7.60 (d, 1H, *J* = 7.9 Hz, H-6_ur_), 7.97 (dd, 1H, *J* = 2.6 Hz, *J* = 8.7 Hz, H-4_pyr_), 8.60 (dd, 1H, *J* = 0.6 Hz, *J* = 2.6 Hz, H-6_pyr_). ^13^C-NMR (100 MHz, CD_3_OD) δ 38.09 (CH_2_CO), 62.78 (C-6), 66.33 (CH_2_O), 71.31 (C-4), 73.89 (C-2), 78.18 (CH_2_N), 79.71 (C-3), 82.16 (C-5), 86.91 (C-1), 103.01 (C-5_ur_), 125.38 (C-3_pyr_), 129.77 (C-4_pyr_), 134.92 (C-5_pyr_), 141.88 (C-6_pyr_), 146.18 (C-6_ur_), 152.85 (C-2_pyr_), 152.95 (C-2_ur_), 166.44 (C-4_ur_), 172.09 (NHCO). HRMS (ESI) (*m*/*z*): [M + H]^+^ calcd for C_19_H_25_N_4_O_9_S, 485.1342; found 485.1341. 

Glycoconjugate (**65**) Purified by column chromatography in CHCl_3_:MeOH solvent system (10:1 to 2:1 (*v*/*v*)) to give white solid ( 45 mg, 78%): ${\left[\alpha \right]}_{D}^{22}$ −34. 7 (c 1.0, CH_3_OH). ^1^H-NMR (400 MHz, CD_3_OD): δ 2.64 (t, 2H, *J* = 5.9 Hz, CH_2_CO), 3.56 (dd, 1H, *J* = 3.3 Hz, *J* = 9.2 Hz, H-3), 3.64–3.78 (m, 4H, H-2, H-5, H-6a, H-6b), 3.90 (t, 2H, *J* = 5.9 Hz, CH_2_O), 3.93 (d, 1H, *J* = 3.1 Hz, H-4), 5.04 (d, 1H, *J* = 9.9 Hz, H-1), 5.17 (s, 2H, CH_2_N), 5.65 (d, 1H, *J* = 7.9 Hz, H-5_ur_), 7.49 (dd, 1H, *J* = 0.7 Hz, *J* = 8.7 Hz, H-3_pyr_), 7.60 (d, 1H, *J* = 7.9 Hz, H-6_ur_), 7.95 (dd, 1H, *J* = 2.6 Hz, *J* = 8.7 Hz, H-4_pyr_), 8.59 (dd, 1H, *J* = 0.5 Hz, *J* = 2.6 Hz, H-6_pyr_). ^13^C-NMR (100 MHz, CD_3_OD): δ 38.09 (CH_2_CO), 62.69 (C-6), 66.34 (CH_2_O), 70.52 (C-4), 70.90 (C-2), 76.35 (C-3), 78.18 (CH_2_N), 80.84 (C-5), 87.41 (C-1), 103.01 (C-5_ur_), 125.06 (C-3_pyr_), 129.78 (C-4_pyr_), 134.74 (C-5_pyr_), 141.78 (C-6_pyr_), 146.19 (C-6_ur_), 152.97 (C-2_pyr_), 153.34 (C-2_ur_), 166.47 (C-4_ur_), 172.08 (NHCO). HRMS (ESI) (*m*/*z*): [M + H]^+^ calcd for C_19_H_25_N_4_O_9_S, 485.1342; found 485.1337.

### 3.3. Biological Evaluation

#### Enzymatic Assay

β4GalT activity was assayed using UDP-Gal as a glycosyl donor and (6-esculetinyl) β-d-glucopyranoside (esculine) as a glycosyl acceptor in a total volume of 200 μL. The final concentrations of the reagents in the reaction mixtures were as follows:

Hepes buffer (pH 5.4) or citrate buffer (pH 5.4)—50 mM
MnCl_2_—10 mM,
BSA—2.0 mg/mL,
Esculine—200 μM,
UDP-Gal—40 μM,
MeOH—10 µL,
Glycoconjugate **33**–**65**—0.8 mM.

The enzymatic reactions were initiated by the addition of 0.1 mU β4GalT solution and subsequently incubated at 30 °C in a thermoblock. After 60 min. the enzyme was inactivated by heating the reaction mixture to 90 °C for 3 min. Resulting suspension was diluted with freshly distilled water (300 μL) and centrifuged for 20 min. (6000 rpm). The supernatant was filtered through M.E. Cellulose disc filter (0.2 µm × 13 mm) and the filtrate was injected into HPLC column. The inhibitory activity of the compounds was evaluated from the intensity of the peaks on the chromatogram referring to the product of the enzymatic reaction ((6-esculetinyl) 4′-*O*-β-d-galactopyranosyl-β-d-glucopyranoside). For compound **64** with the enzyme inhibiting activity IC_50_ value was determined using the same procedure using the reaction mixtures containing inhibitor in the concentrations of: 0.1, 0.2, 0.4, 0.8, and 1.6 mM and calculated using CalcuSyn software.

## 4. Conclusions

We have shown that it is convenient to steer the activity of small-molecule inhibitors by modifying some elements of their structure. In particular, the change of the linker structure and the glycoside configuration affects the inhibitory properties of the analogues towards the β4GalT.

In our study, we focused on a simple and efficient synthesis of the analogues of a natural glycosyl donor substrate of β4GalT. Within the glycoconjugates structure, we proposed for the ribose to be replaced with an acyclic linkage. Glycoconjugates exhibit improved stability against hydrolytic cleavage thanks to replacing the oxygen atom between the linker and sugar moiety with sulphur. Additionally, the stability of glycoconjugates was enhanced by the introduction of an amide bond between the linker and aminopyridyl 1-thioglycoside moiety. 

For the preparation of (5-nitro-2-pyridyl) 1-thio-α-d-glycosides, a number of synthetic procedures were used and, at last, we have applied one that was previously developed in our group. It is worth noting that products **27**, **28**, **30**, and **32** are new compounds not described in the literature so far. Also important is the successful use of lipase to obtain the product **32** in a stereoselective way. As a result of the studies a series of glycoconjugates containing both benzyl and acetyl protections in the sugar unit was obtained. Their deprotection allowed for the obtainment of a series of analogues of natural β4GalT substrates.

The biological activity of glycoconjugates has been checked using commercially available β4GalT. No inhibition against β4GalT when using glycoconjugates **55**–**63** suggests that the α-d-thiogalacto- and α-d-thioglucopyranoside motif connected with uridine is not sufficient to ensure binding at the active site of the enzyme. Unexpectedly, it turned out that the glucoconjugate **64** with the β configuration at the sugar anomeric centre is able to inhibit the enzyme, and what is particularly interesting, the d-gluco- derivative is more active than d-galacatose conjugate. A similar pattern was observed earlier in the case of uridine glycoconjugate in which the pyrophosphate linker was replaced with *O*-methylene triazole unit [66]. At the time, it was suspected that this state of affairs is due to the relatively rigid structure of the linker. In the case of currently described glycoconjugates, it was assumed that replacing the rigid *O*-methylene triazole linker connected with C-5 of the ribose unit by a more flexible aliphatic link would allow a better fit to the active enzyme centre. However, to determine the mechanism of their action further studies are needed.

The results presented in this paper provide evidence for the efficacy of inhibitor mimicking parts of the β4GalT natural substrate structure. Although compound **64** bearing a β-d-glucose fragment is β4GalT inhibitor in vitro, further experiments are needed for testing whether the described results can be extrapolated to other glycosyltransferases.

## Figures and Tables

**Table 1:** Preparation of compounds **3**–**14**.

Entry	Substrate	Product	Procedure	Yield [%]	α:β
1	**1**	**3**	**A**	46	1:2
2	**2**	**4**	**A**	26	5:1
3	**1b**	**5**	**E’**	79	4:1
4	**2b**	**6**	**E’**	77	1.4:1
5	**9**	**13**	**I**	36	only α
6	**10**	**14**	**I**	12	only α
7	**9**	**11**	**G**	98	only α
8	**10**	**12**	**G**	94	only α
9	**11**	**13**	**H**	87	only α
10	**12**	**14**	**H**	81	only α

**Table 2:** Yields of glycoconjugates **33**–**54.**

Entry	Thioglycoside	Uridine Derivative	Product	Reaction Time [h]	Yield [%]
1	**17**	**24**	**33**	2	37
2	**18**	**24**	**34**	2	67
3	**17**	**27**	**35**	2	39
4	**18**	**27**	**36**	2	70
5	**17**	**28**	**37**	3	49
6	**18**	**28**	**38**	2	60
7	**17**	**30**	**39**	2	39
8	**18**	**30**	**40**	2	48
9	**17**	**32**	**41**	3	31
10	**18**	**32**	**42**	2	56
11	**19**	**24**	**43**	4	32
12	**20**	**24**	**44**	3	25
13	**19**	**27**	**45**	3	37
14	**20**	**27**	**46**	3	23
15	**19**	**28**	**47**	3	43
16	**20**	**28**	**48**	3	41
17	**19**	**30**	**49**	2	49
18	**20**	**30**	**50**	3	47
19	**19**	**32**	**51**	3	30
20	**20**	**32**	**52**	3	23
21	**21**	**28**	**53**	1.5	70
22	**22**	**28**	**54**	1.5	65

## Supplementary Materials

Supplementary materials are available online. Figure S1–S86: ^1^H- and ^13^C-NMR spectra of compounds **13**, **14**, **17**–**20**, **27**, **28**, **30**, and **30**–**65**.
