# Supplementary material for: Synthesis and Preliminary Evaluation of Biological Activity of Glycoconjugates Analogues of Acyclic Uridine Derivatives
Source: Molecules. 2018 Aug 13;23(8):2017. doi: 10.3390/molecules23082017 (PMC6222857; doi:10.3390/molecules23082017)
Supplement: Supplementary file 1 [file molecules-23-02017-s001.pdf]

Supporting information

Synthesis and preliminary evaluation of biological activity of glycoconjugates, analogues of acyclic uridine derivatives

Roman Komor[1*], Gabriela Pastuch-Gawołek[1,2]*,Ewelina Krol[3] and Wiesław Szeja[1]

[1]*Silesian University of Technology, Faculty of Chemistry, Chair of Organic Chemistry, Bioorganic Chemistry and Biotechnology, Krzywoustego 4, 44-100 Gliwice, Poland*

[2]*Silesian University of Technology, Biotechnology Center, Krzywoustego 8, 44-100 Gliwice, Poland*

[3]*University of Gdansk and Medical University of Gdansk, Department of Recombinant Vaccines, Intercollegiate Faculty of Biotechnology, Abrahama 58, 80-307 Gdansk, Poland*

*Corresponding author. Phone: +48 32 237 21 38; e-mail: gabriela.pastuch@polsl.pl, roman.m.komor@gmail.com

1. Spectra

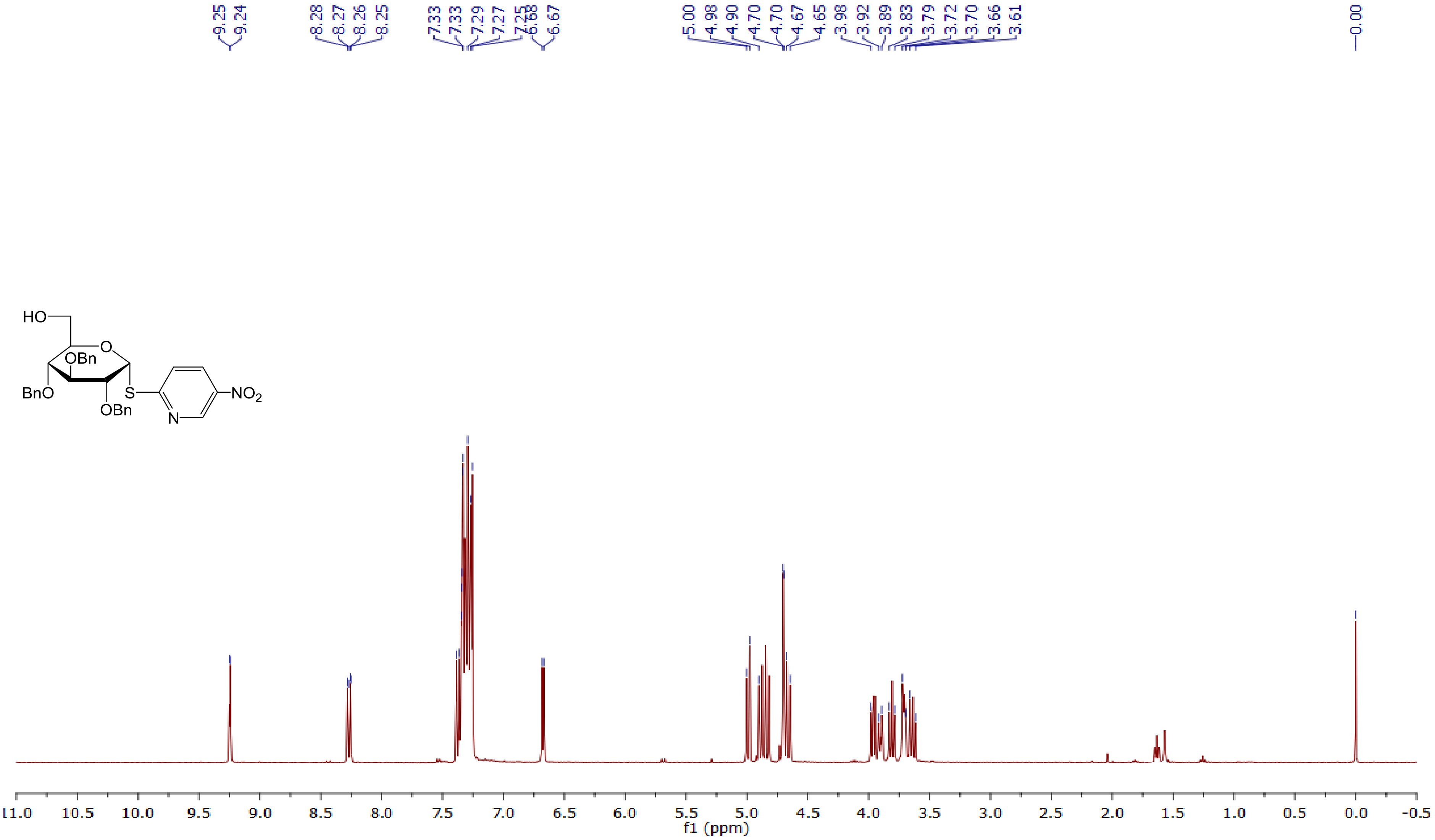

Fig. S1: ^{1}H NMR spectrum of (5-nitro-2-pyridyl) 2,3,4-tri-*O*-benzyl-1-thio-α-D-glucopyranoside **13**

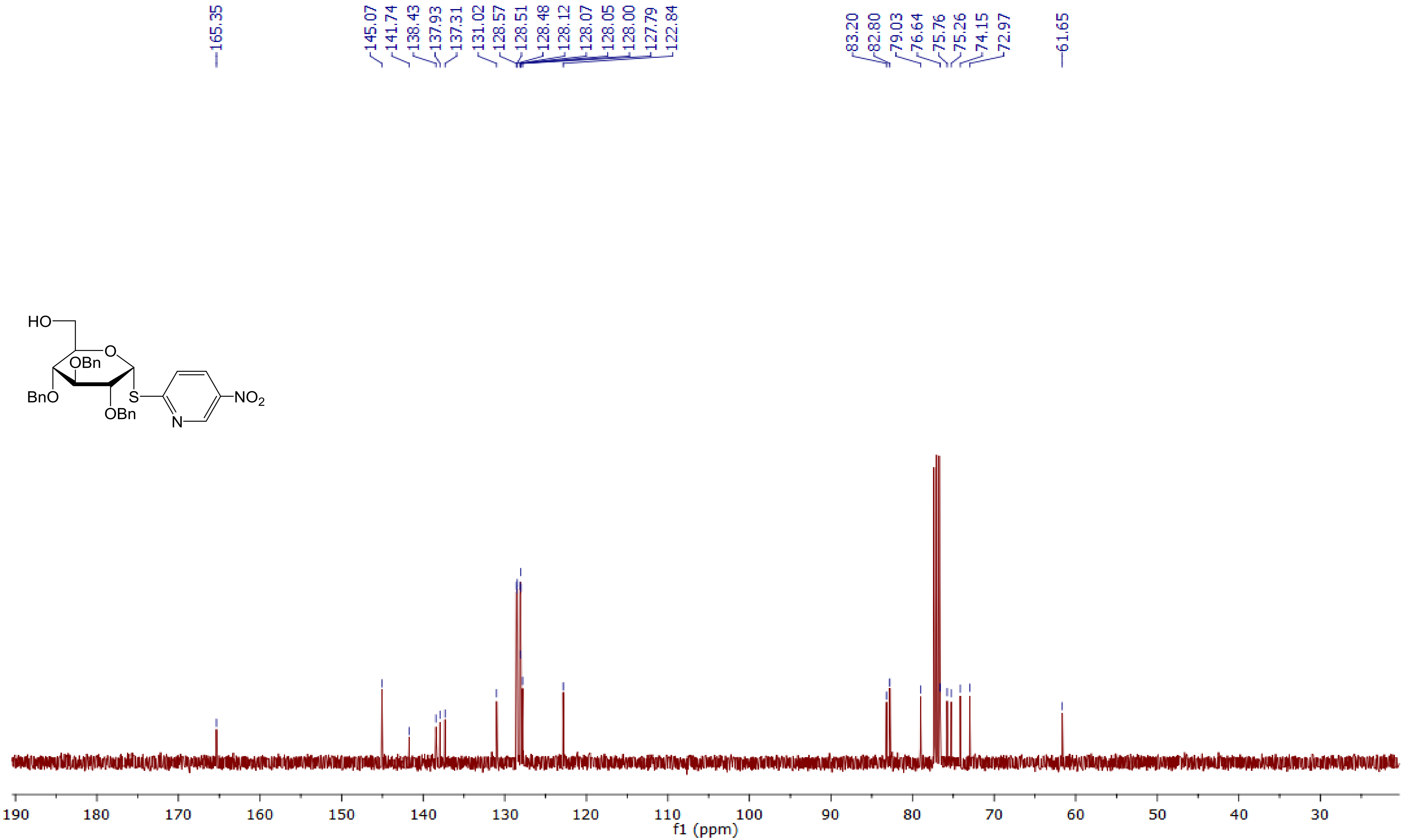

Fig. S2: ^{13}C NMR spectrum of (5-nitro-2-pyridyl) 2,3,4-tri-*O*-benzyl-1-thio-α-D-glucopyranoside **13**

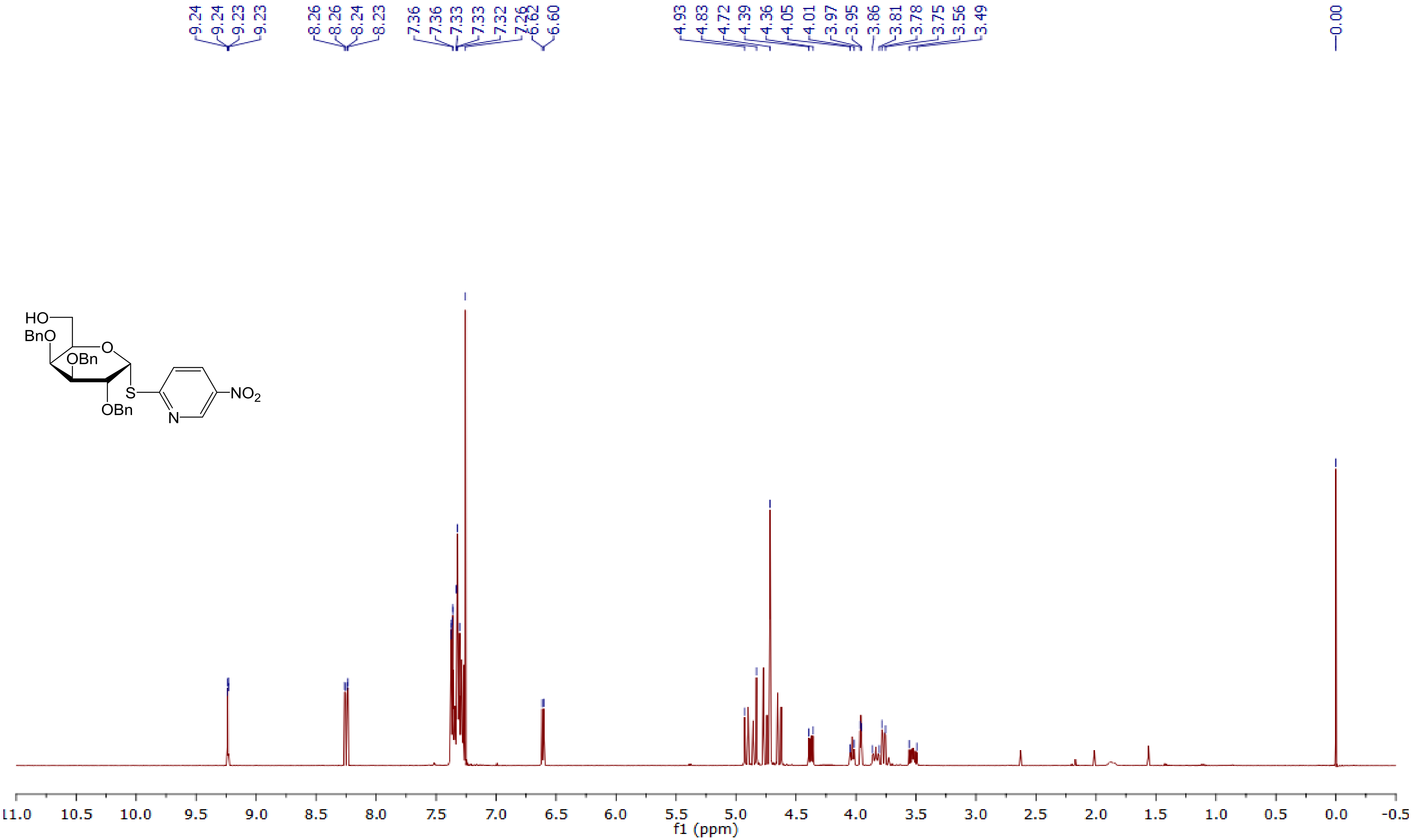

Fig. S3: ^{1}H NMR spectrum of (5-nitro-2-pyridyl) 2,3,4-tri-*O*-benzyl-1-thio-α-D-galactopyranoside **14**

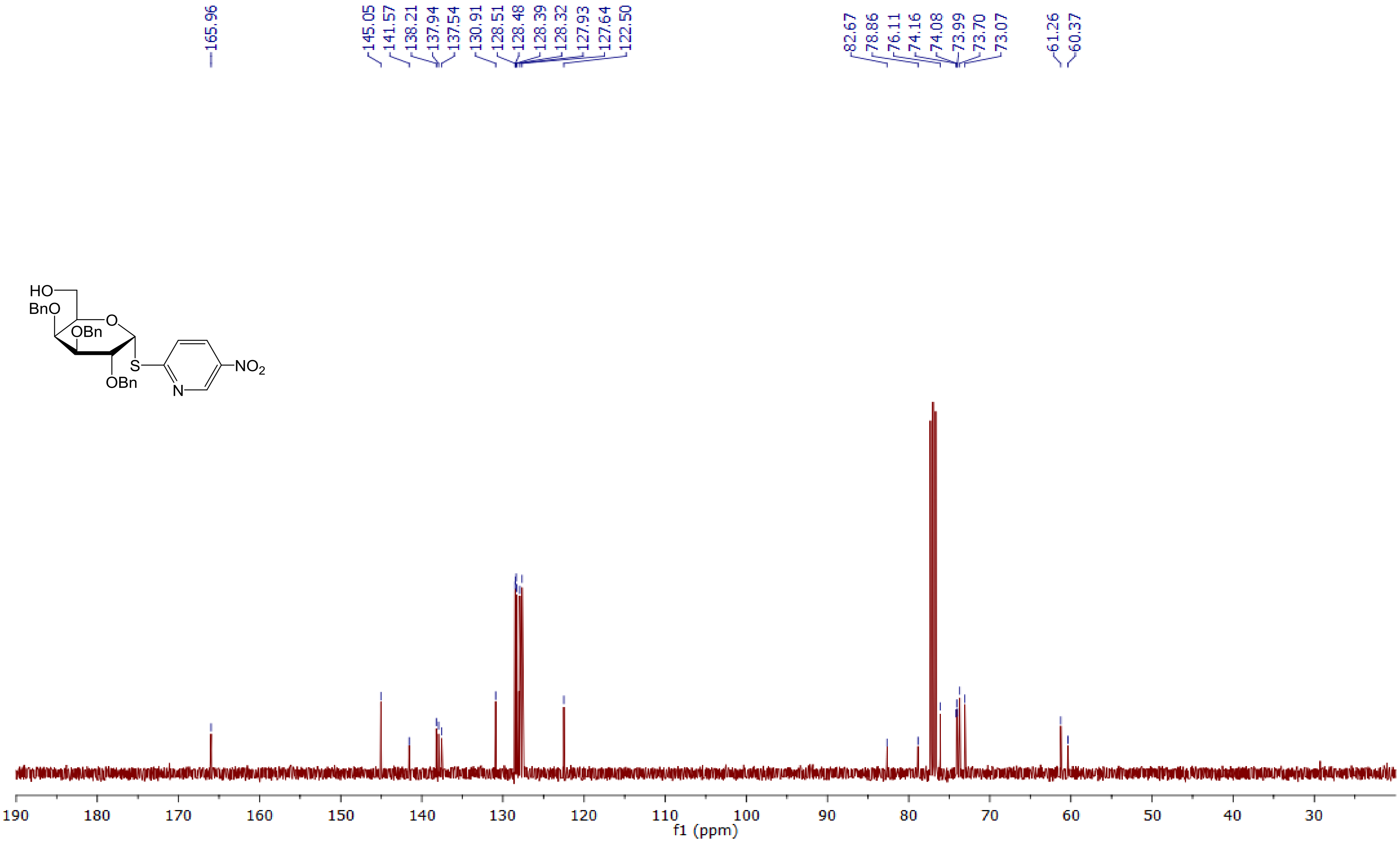

Fig. S4: ^{13}C NMR spectrum of (5-nitro-2-pyridyl) 2,3,4-tri-*O*-benzyl-1-thio-α-D-galactopyranoside **14**

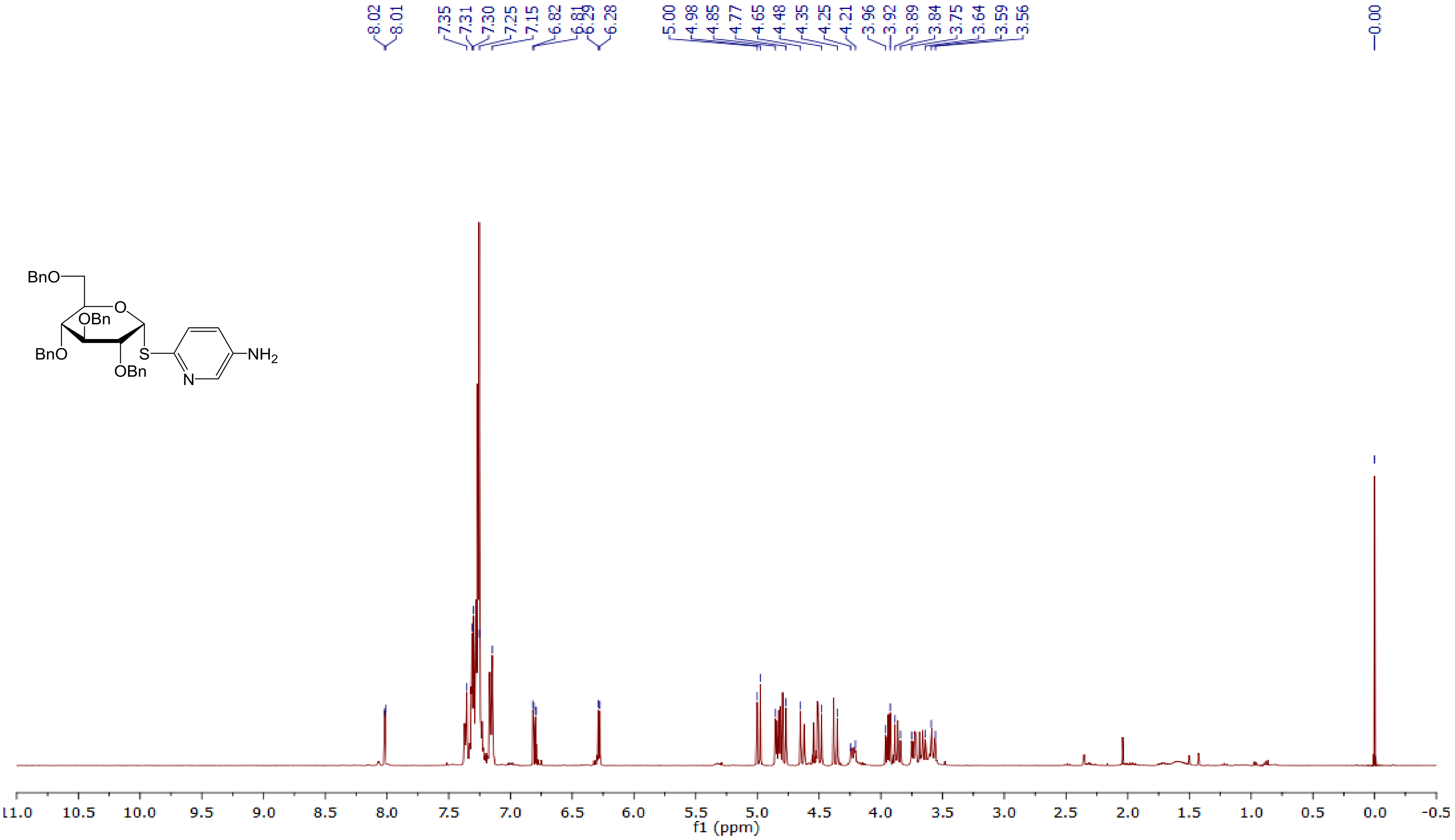

Fig. S5: ^{1}H NMR spectrum of (5-amino-2-pyridyl) 2,3,4,6-tetra-*O*-benzyl-1-thio-α-D-glucopyranoside **17**

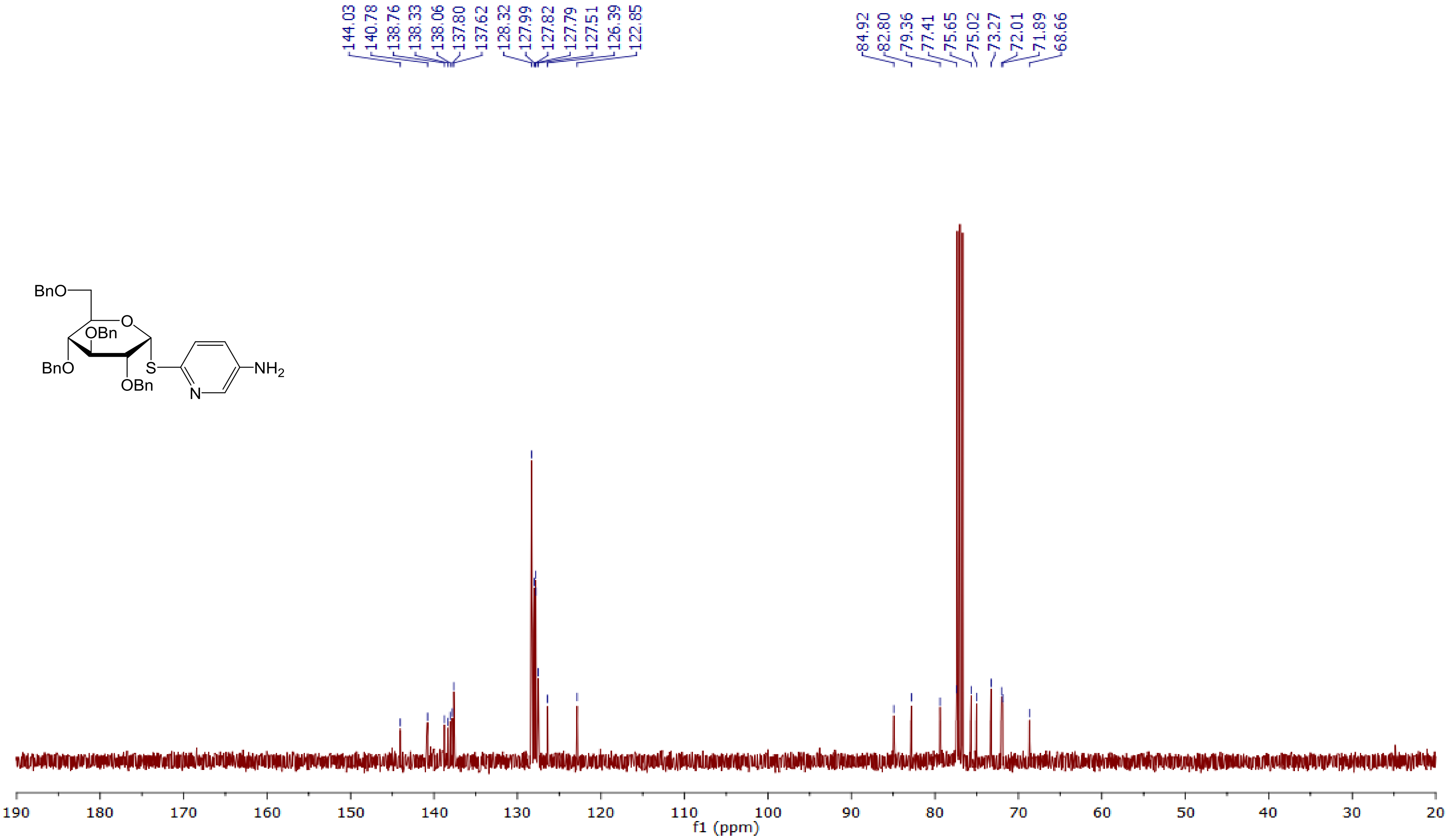

Fig. S6: ^{13}C NMR spectrum of (5-amino-2-pyridyl) 2,3,4,6-tetra-*O*-benzyl-1-thio-α-D-glucopyranoside **17**

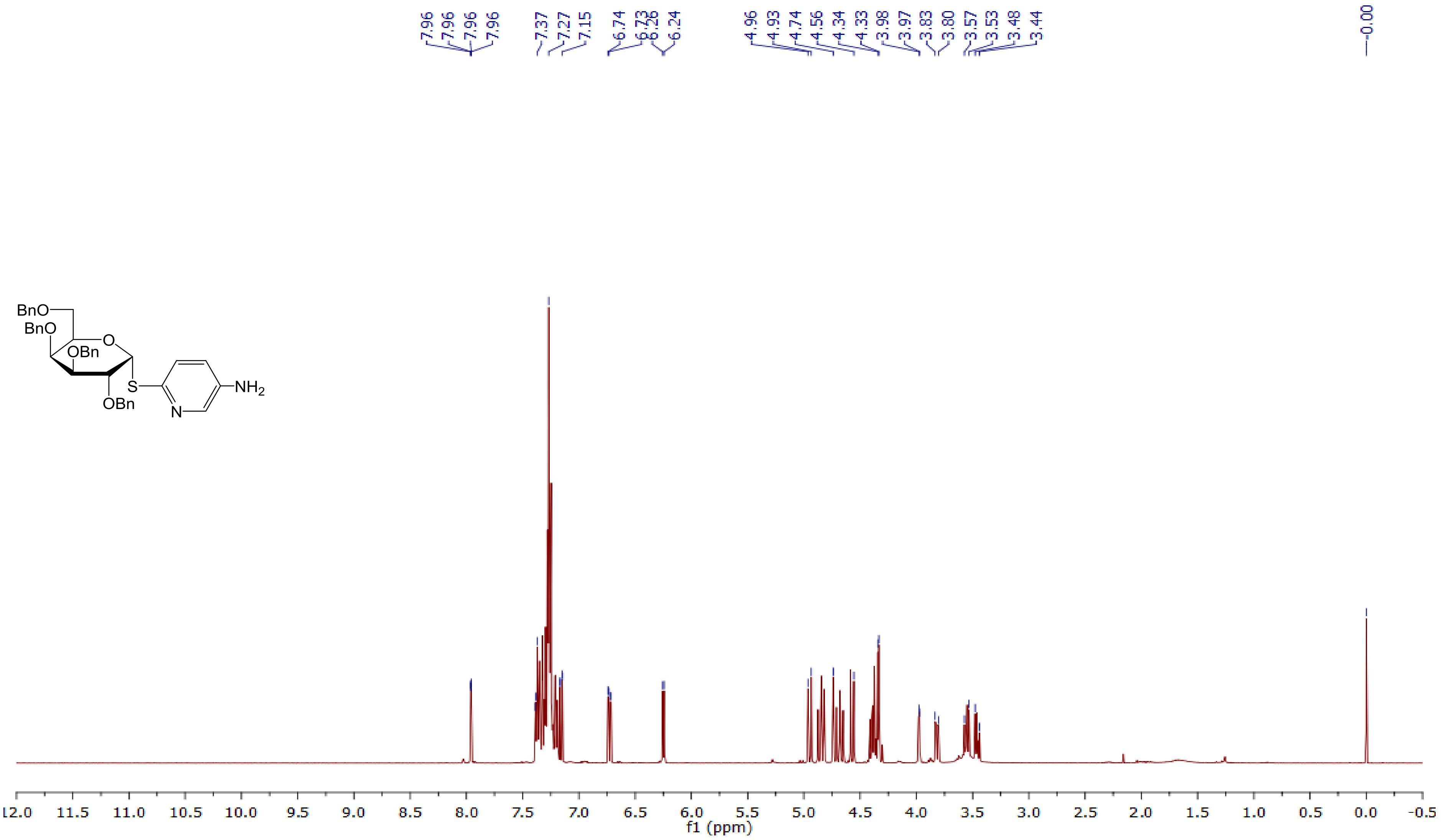

Fig. S7: ^{1}H NMR spectrum of (5-amino-2-pyridyl) 2,3,4,6-tetra-*O*-benzyl-1-thio-α-D-galactopyranoside **18**

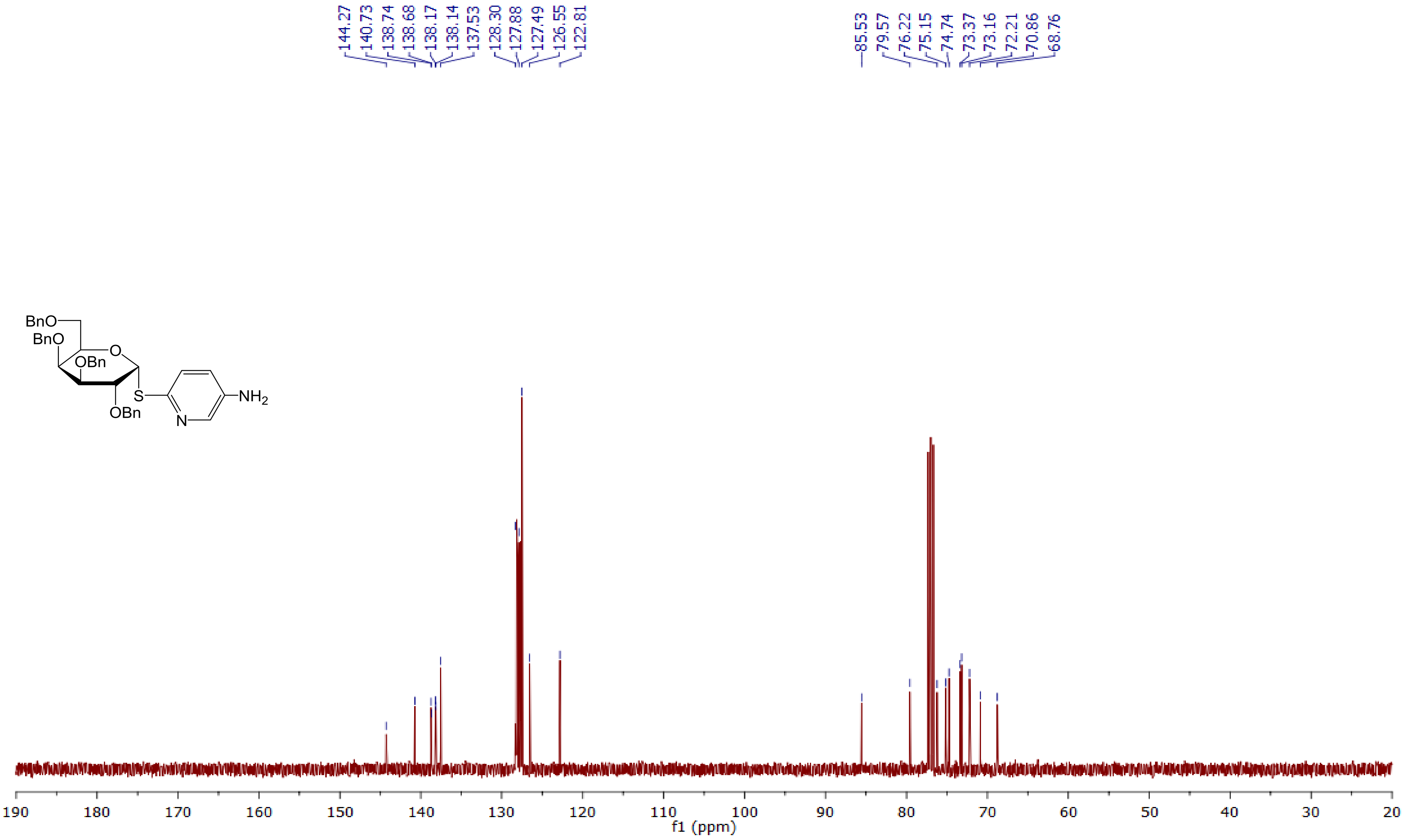

Fig. S8: ^{13}C NMR spectrum of (5-amino-2-pyridyl) 2,3,4,6-tetra-*O*-benzyl-1-thio-α-D-galactopyranoside **18**

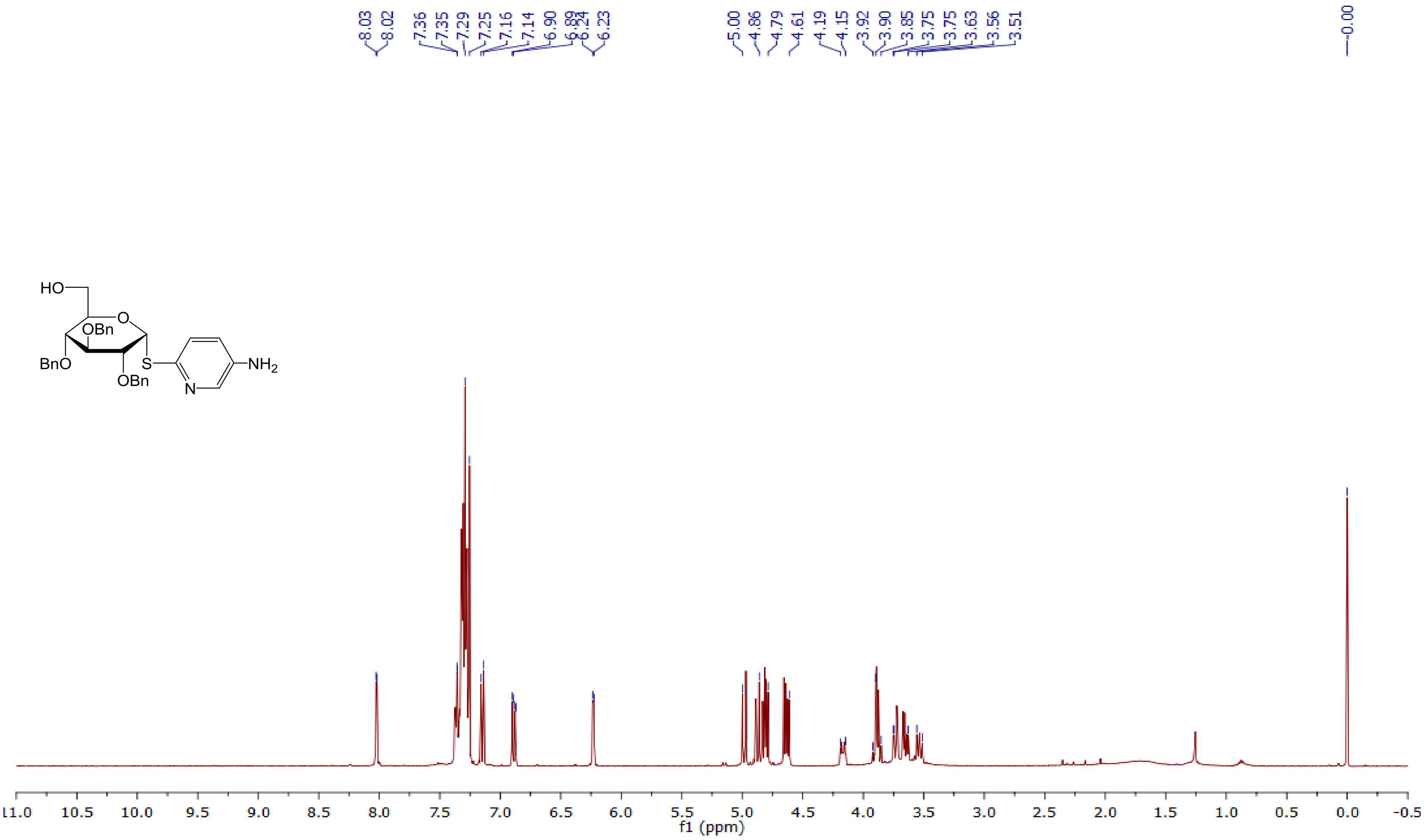

Fig. S9: ^{1}H NMR spectrum of (5-amino-2-pyridyl) 2,3,4-tri-*O*-benzyl-1-thio-α-D-glucopyranoside **19**

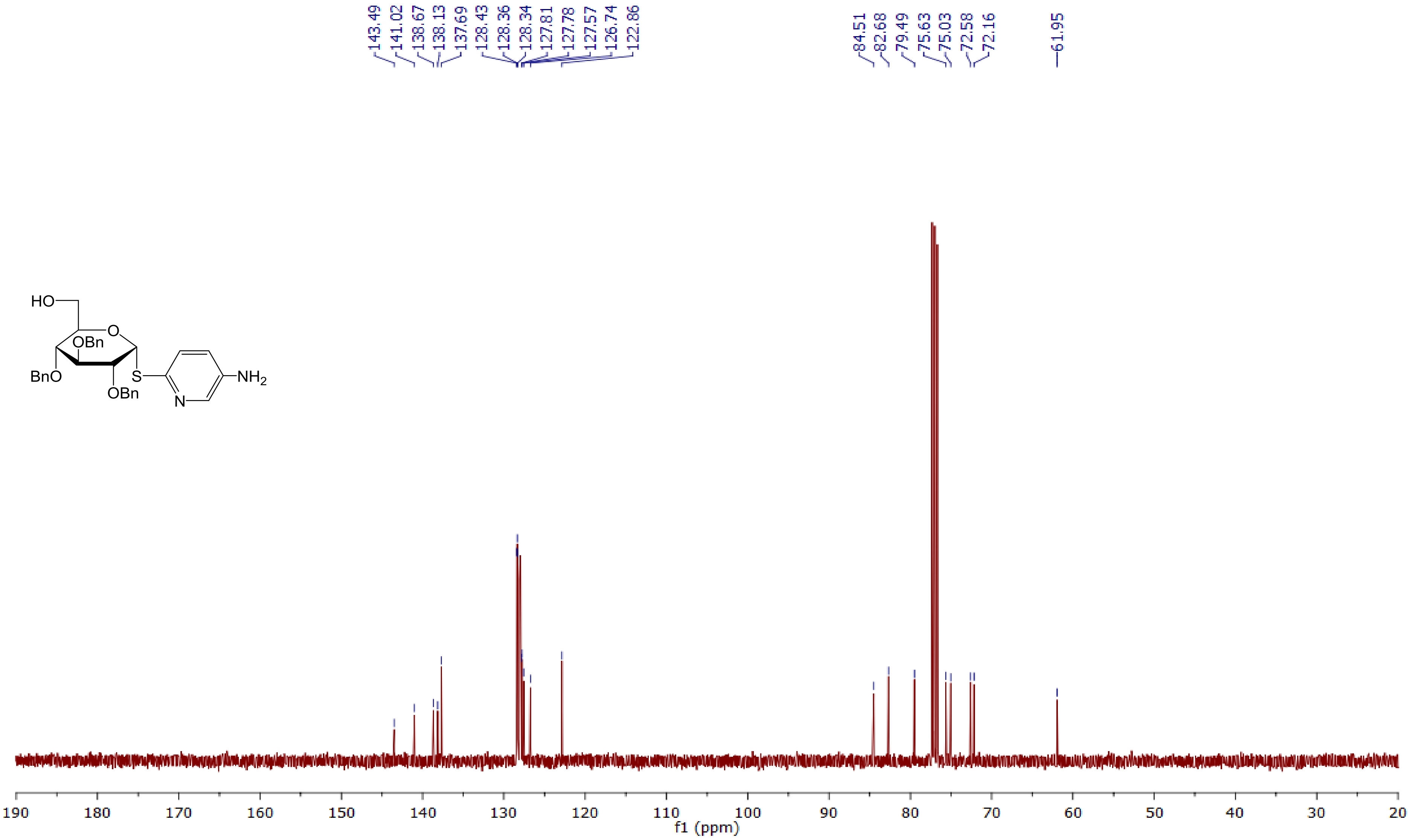

Fig. S10: ^{13}C NMR spectrum of (5-amino-2-pyridyl) 2,3,4-tri-*O*-benzyl-1-thio-α-D-glucopyranoside **19**

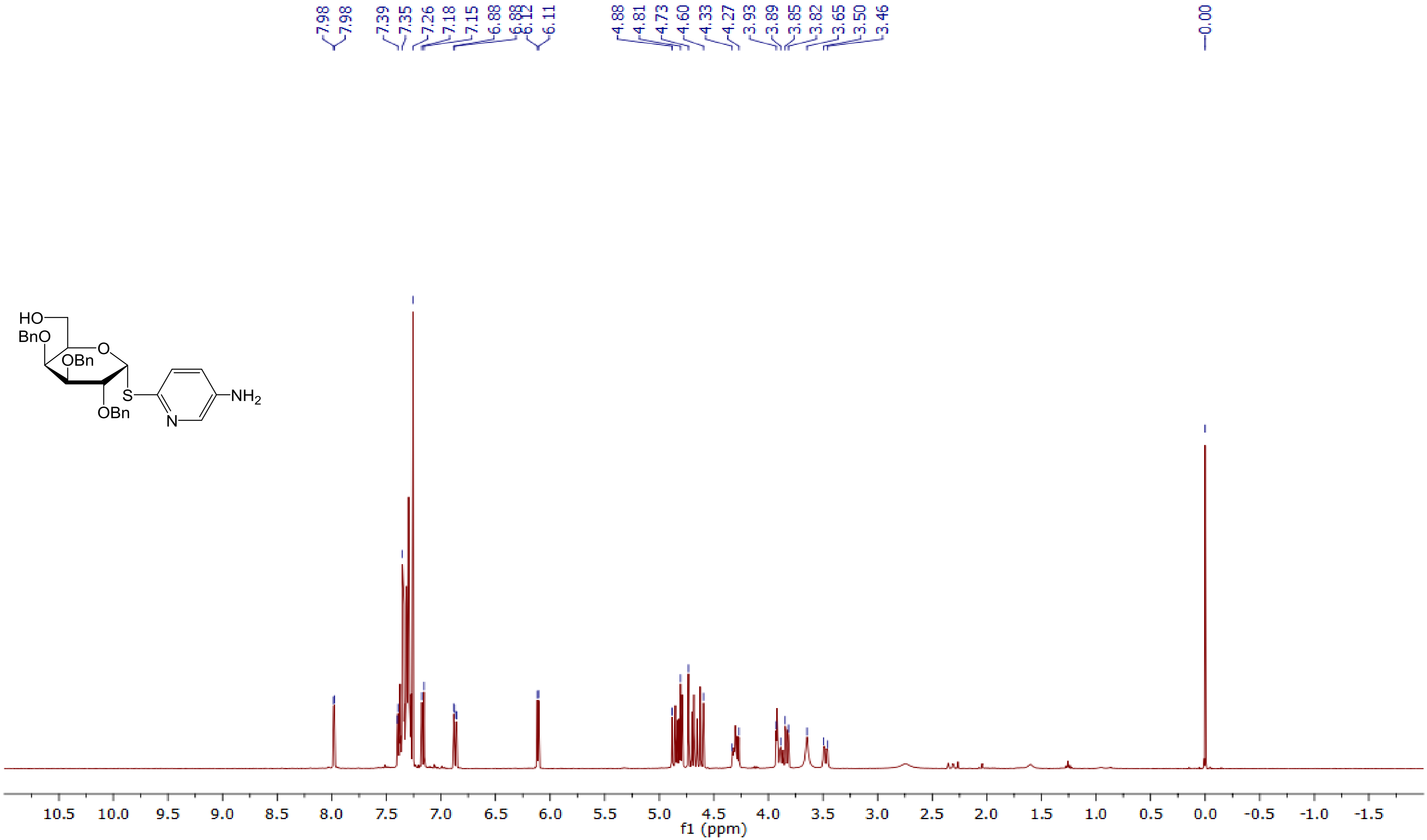

Fig. S11: ^{1}H NMR spectrum of (5-amino-2-pyridyl) 2,3,4-tri-*O*-benzyl-1-thio-α-D-galactopyranoside **20**

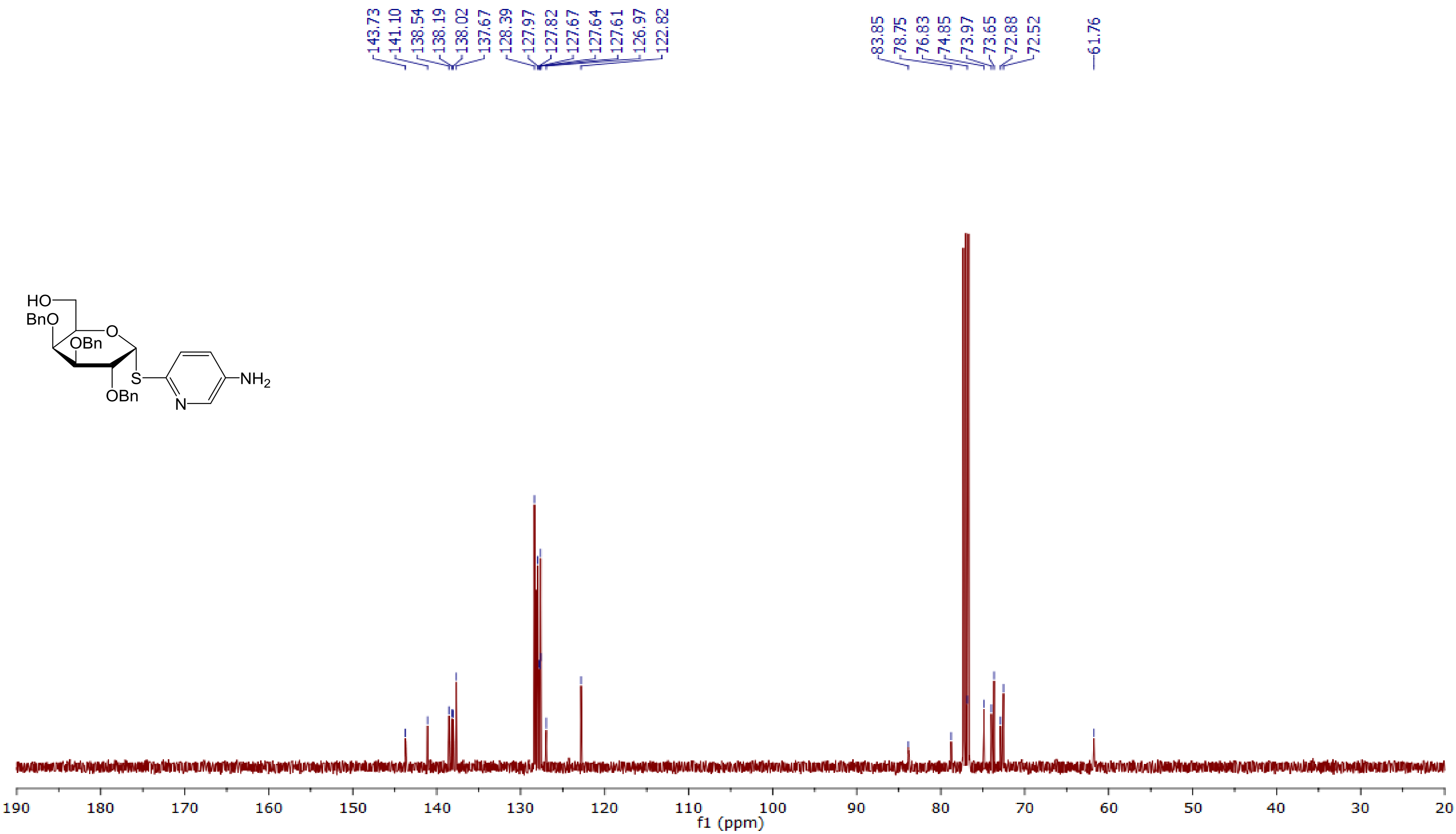

Fig. S12: ^{13}C NMR spectrum of (5-amino-2-pyridyl) 2,3,4-tri-*O*-benzyl-1-thio-α-D-galactopyranoside **20**

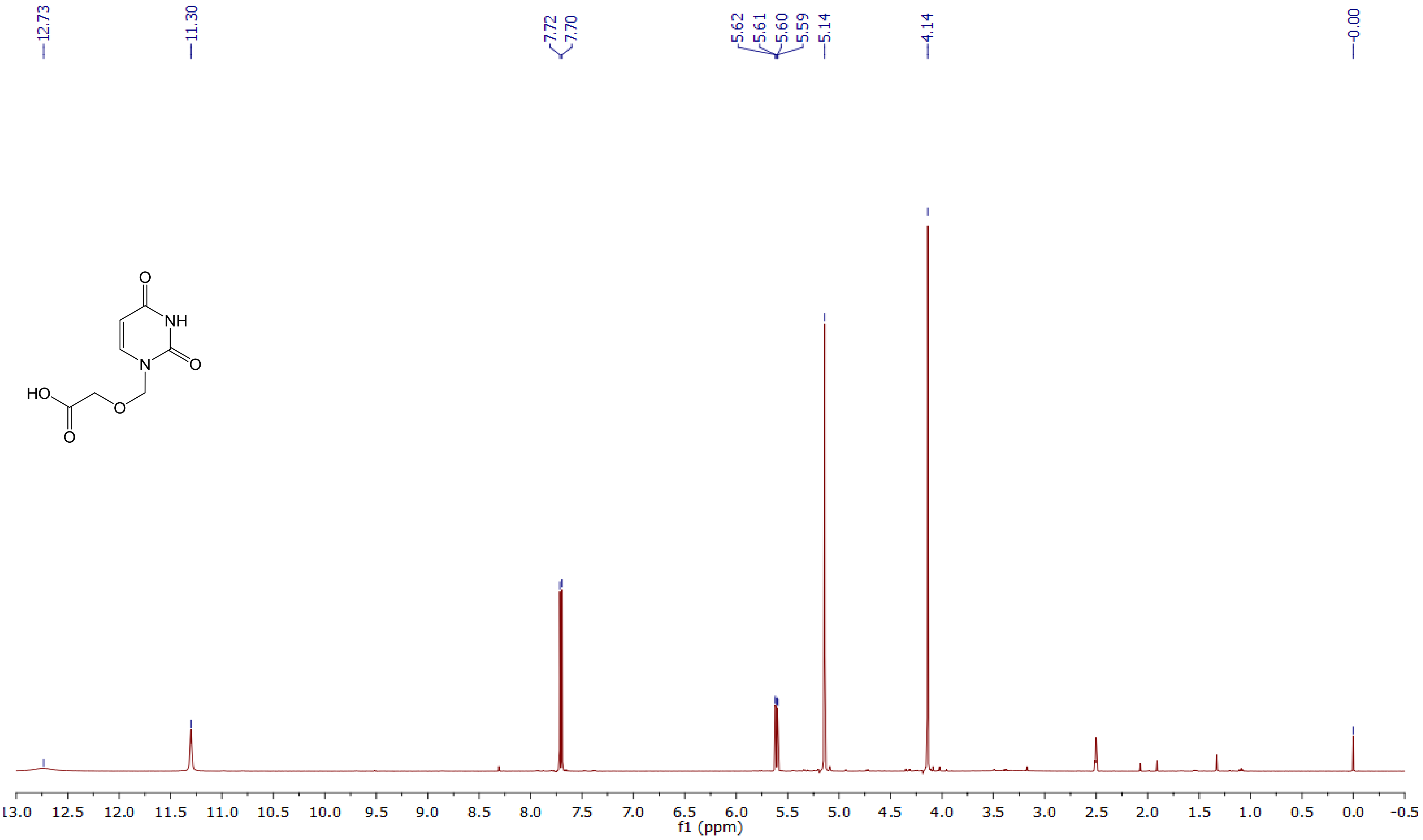

Fig. S13: ^{1}H NMR spectrum of [(2,4-2-[(2,4-dioxo-3,4-dihydropyrimidin-1-(2H)-yl) methoxy]acetic acid **27**

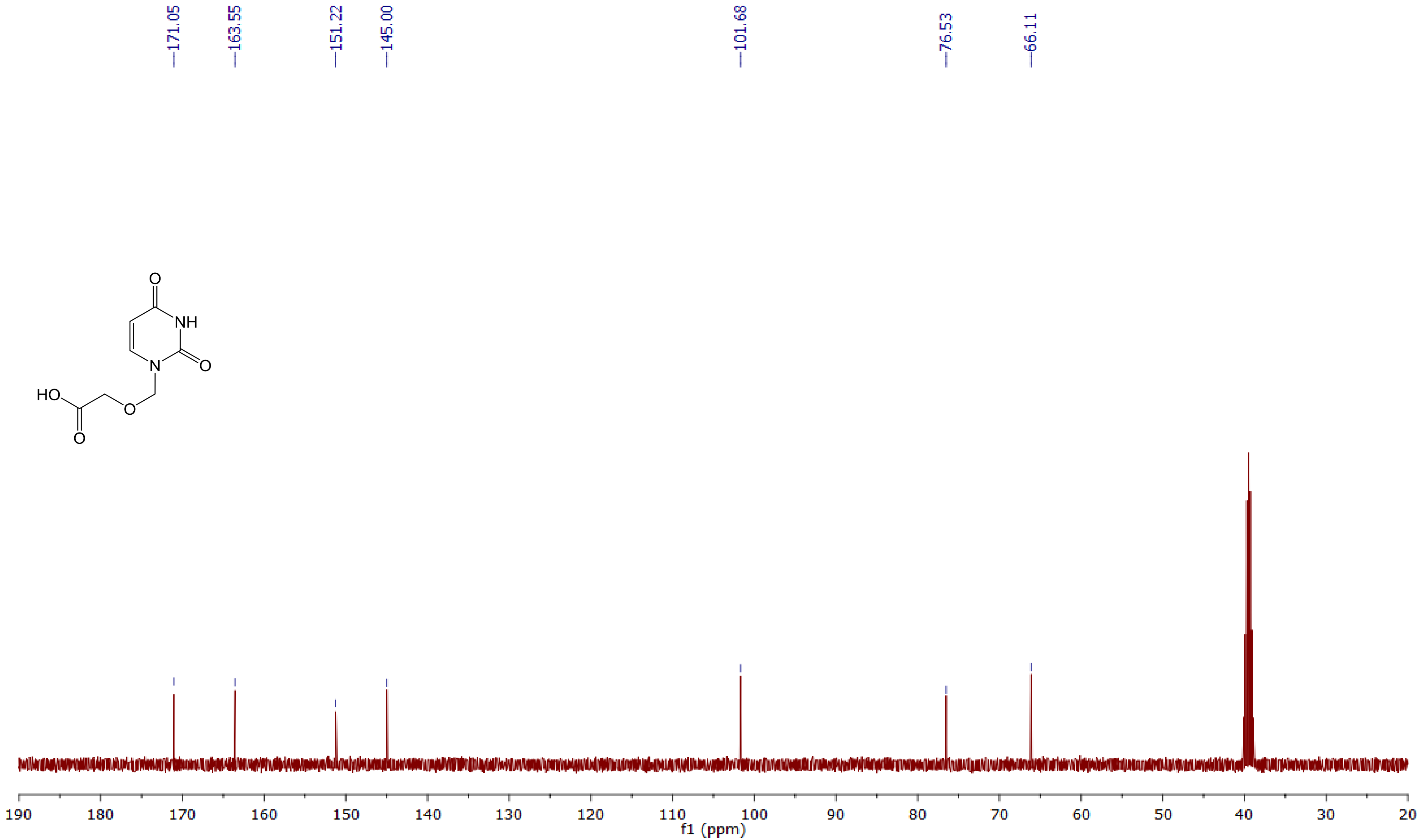

Fig. S14: ^{13}C NMR spectrum of [(2,4-2-[(2,4-dioxo-3,4-dihydropyrimidin-1-(2H)-yl) methoxy]acetic acid **27**

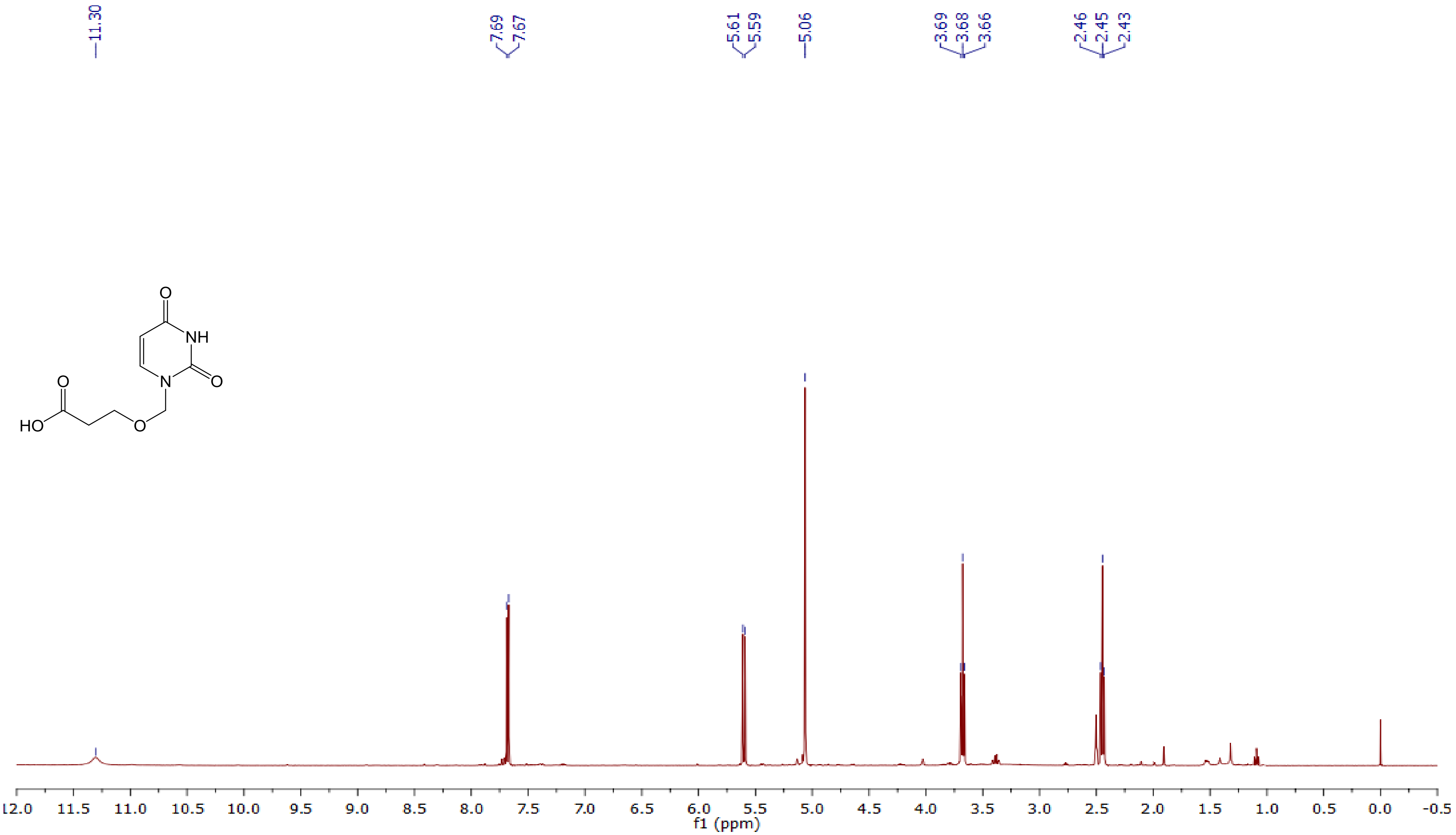

Fig. S15: ^{1}H NMR spectrum of 2-[(2,4-dioxo-3,4)-dihydropyrimidine-1-(2H)-yl) methoxy]propanoic acid **28**

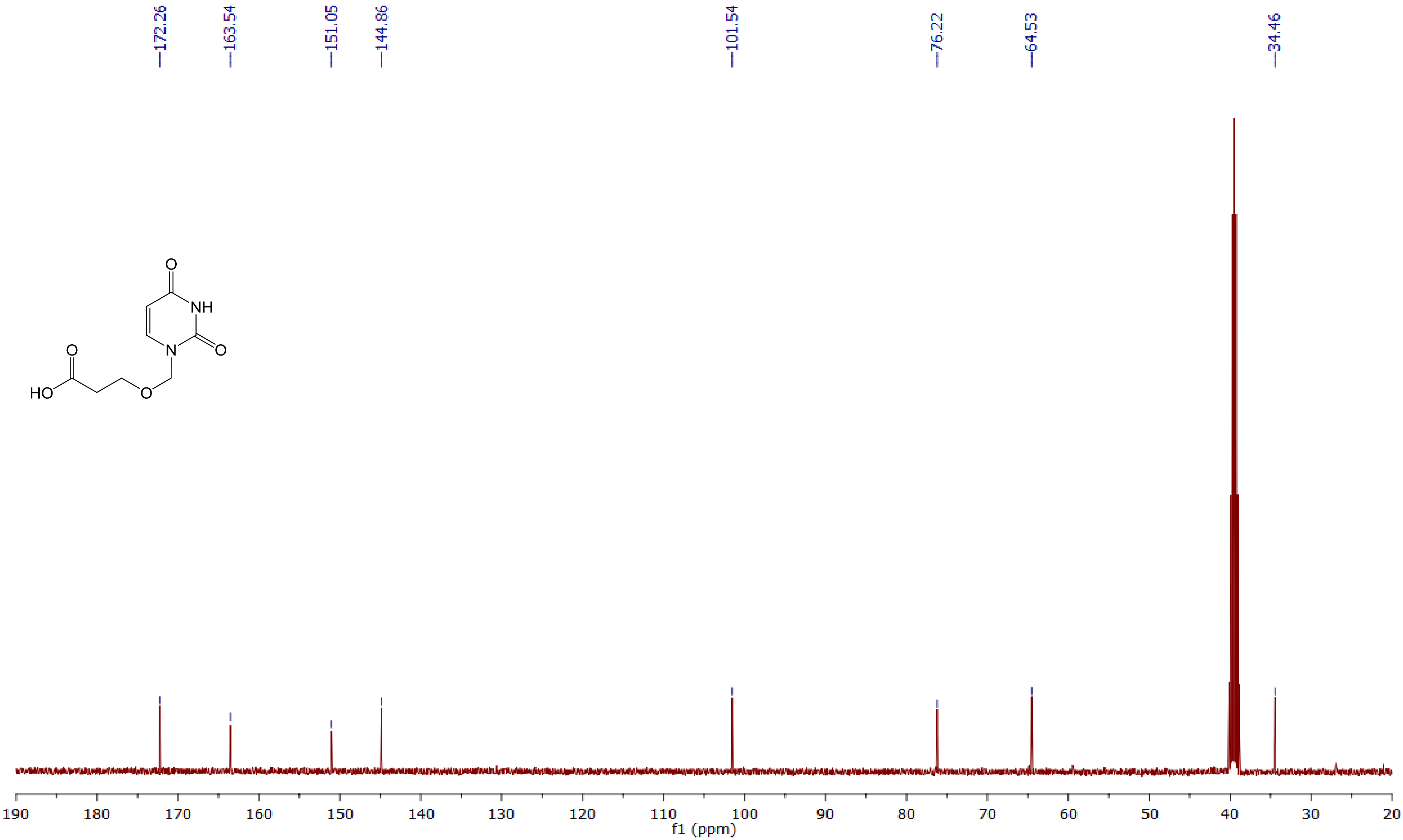

Fig. S16: ^{13}C NMR spectrum of 2-[(2,4-dioxo-3,4)-dihydropyrimidine-1-(2H)-yl) methoxy]propanoic acid **28**

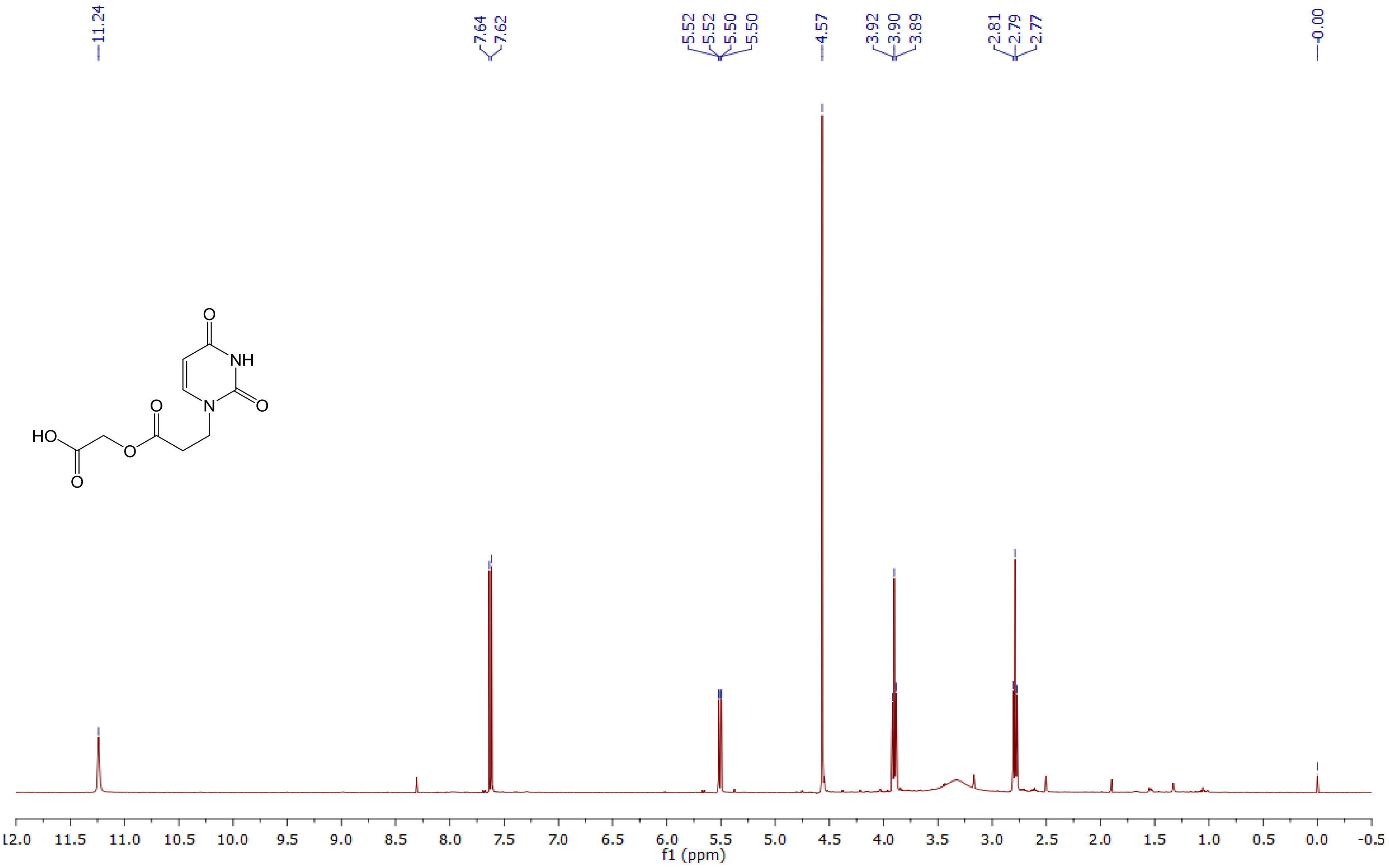

Fig. S17: ^{1}H NMR spectrum of ([3-(2,4-dioxo-3,4-dihydropyrimidin-1(2H)-yl)propanoyl] oxyacetic acid **30**

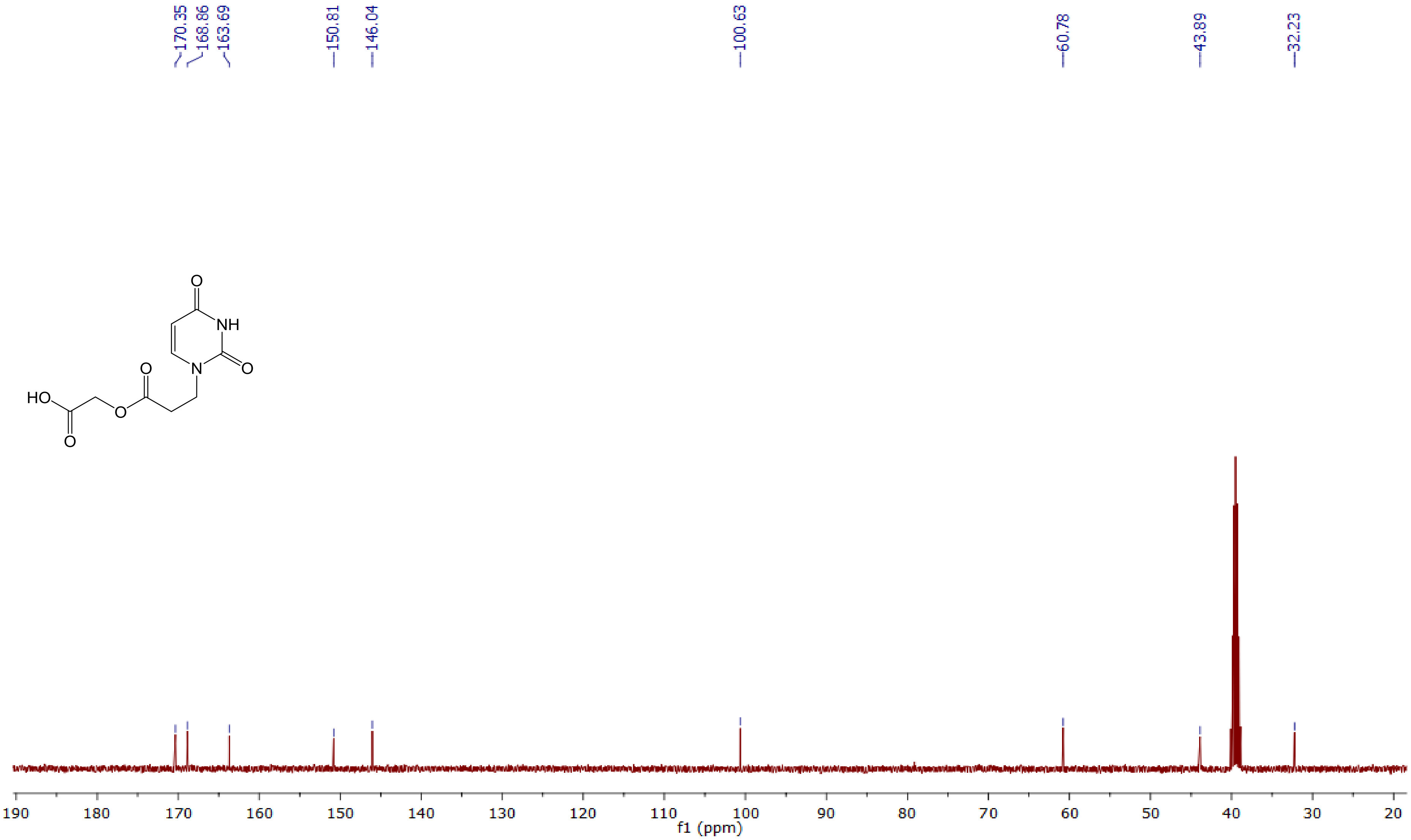

Fig. S18: ^{13}C NMR spectrum of ([3-(2,4-dioxo-3,4-dihydropyrimidin-1(2H)-yl)propanoyl] oxyacetic acid **30**

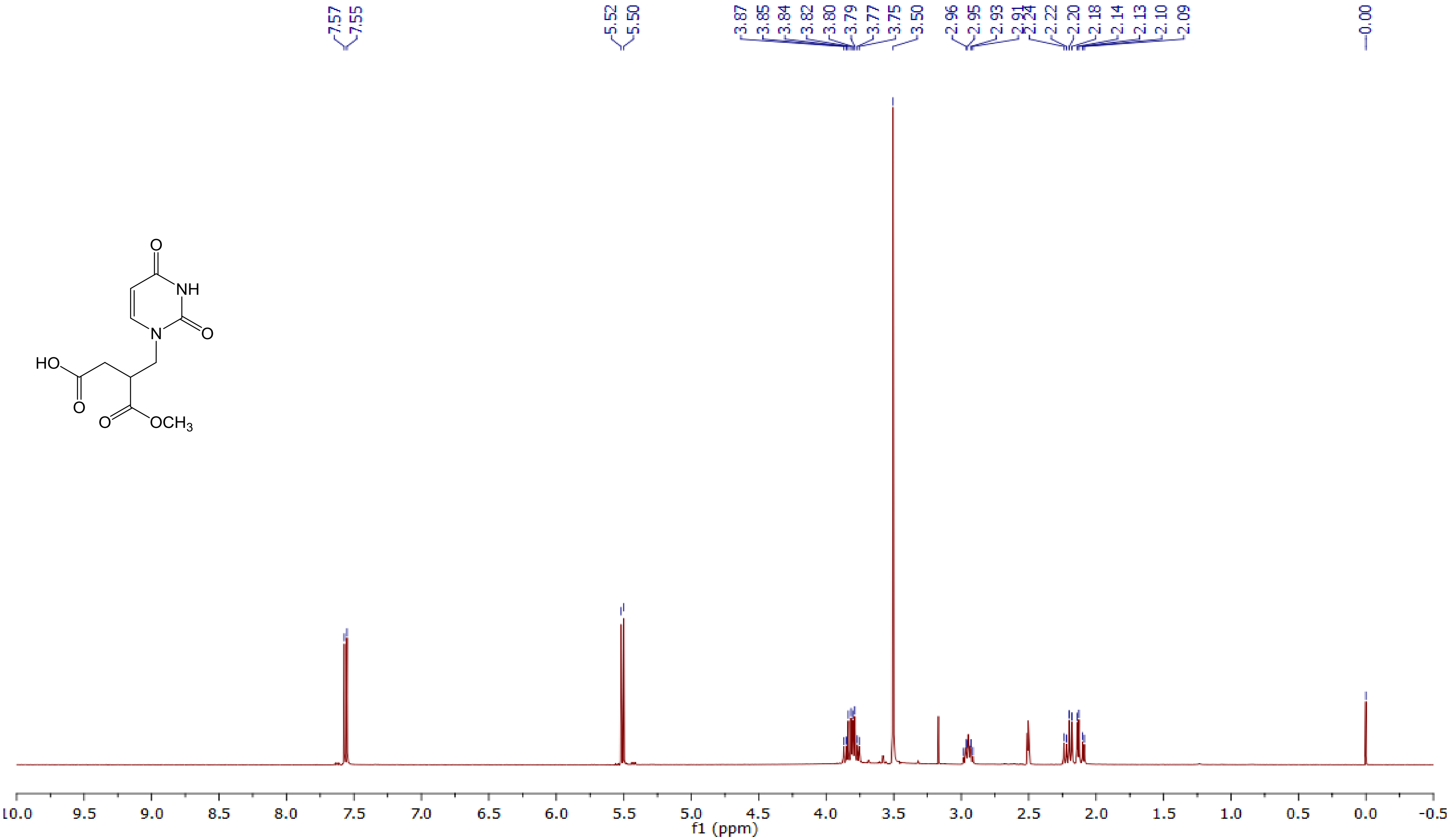

Fig. S19: ^{1}H NMR spectrum of 3-[(2,4-dioxo-3,4-dihydropyrimidin-1(2H)-yl)methyl]-4-oxobutanoic acid **32**

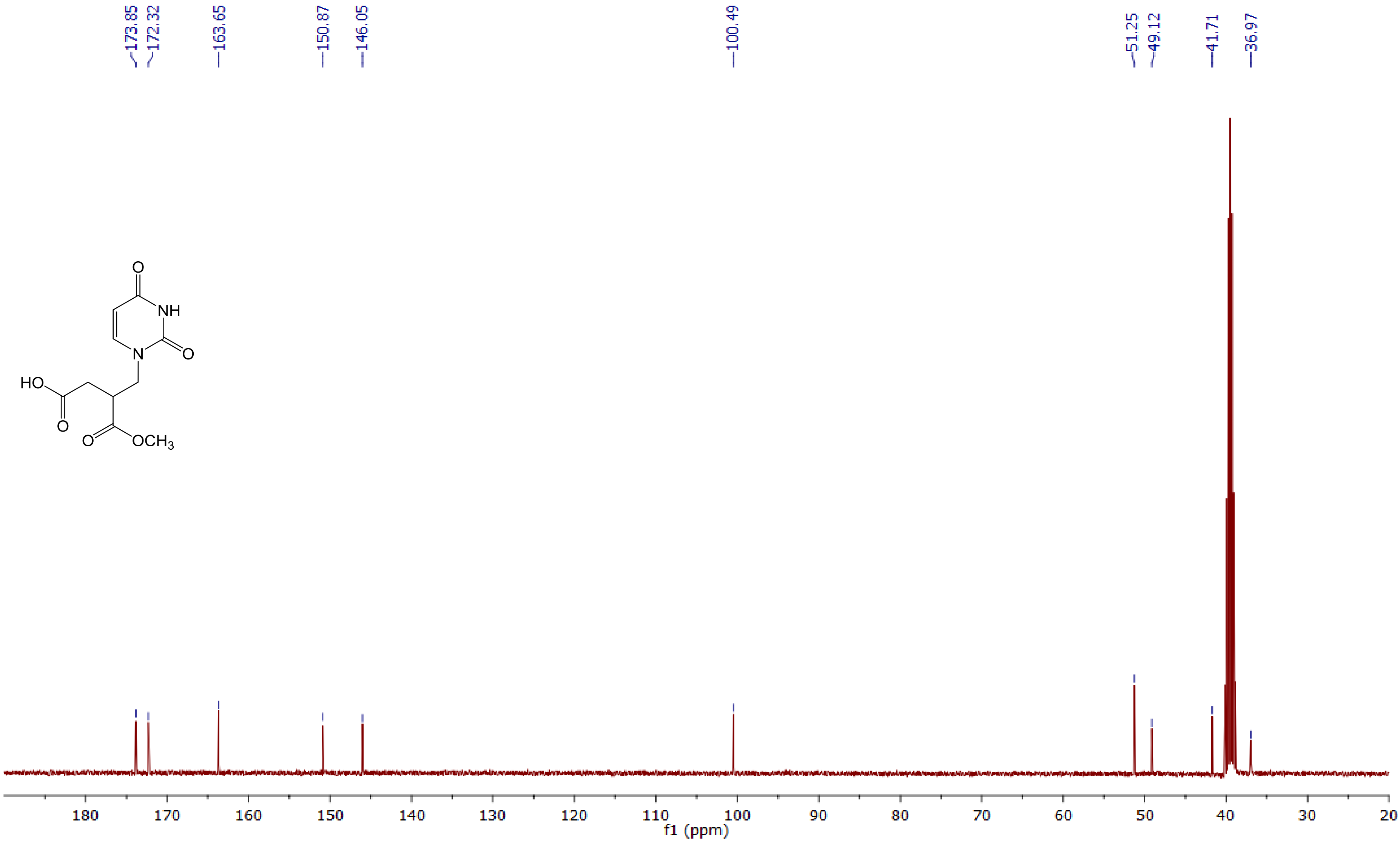

Fig. S20: ^{13}C NMR spectrum of 3-[(2,4-dioxo-3,4-dihydropyrimidin-1(2H)-yl)methyl]-4-oxobutanoic acid **32**

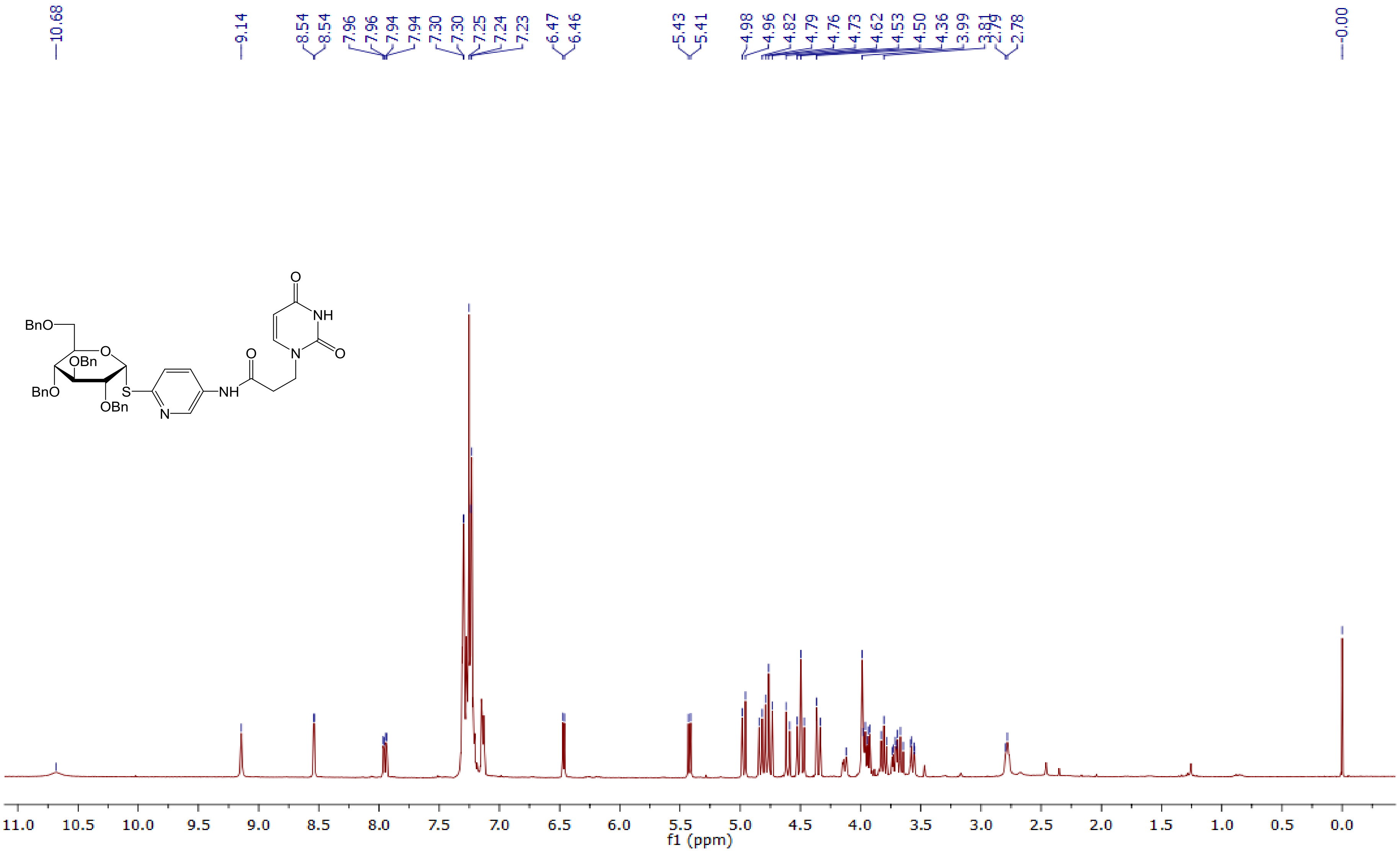

Fig. S21: ^{1}H NMR spectrum of glycoconjugate **33**

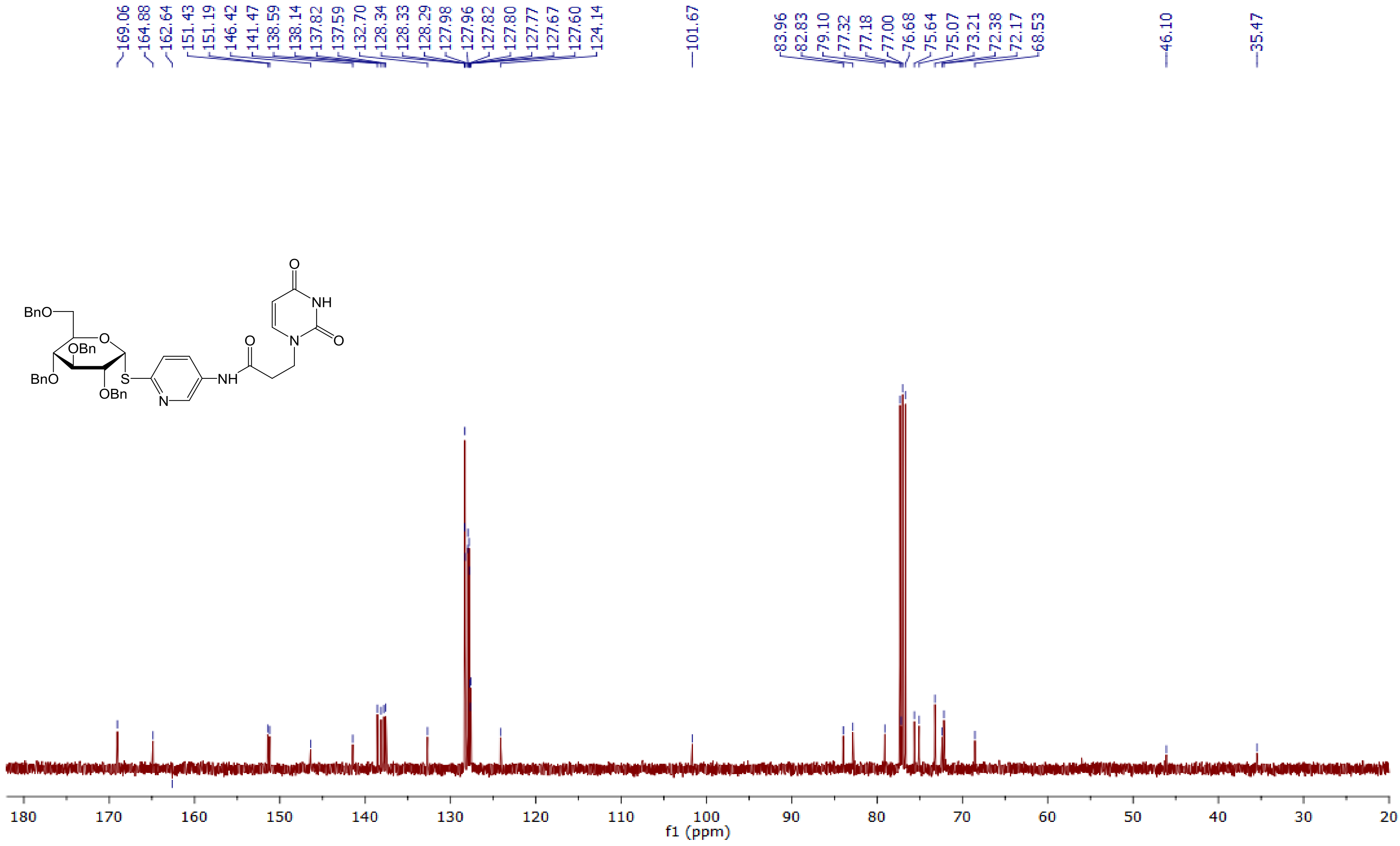

Fig. S22: ^{13}C NMR spectrum of glycoconjugate **33**

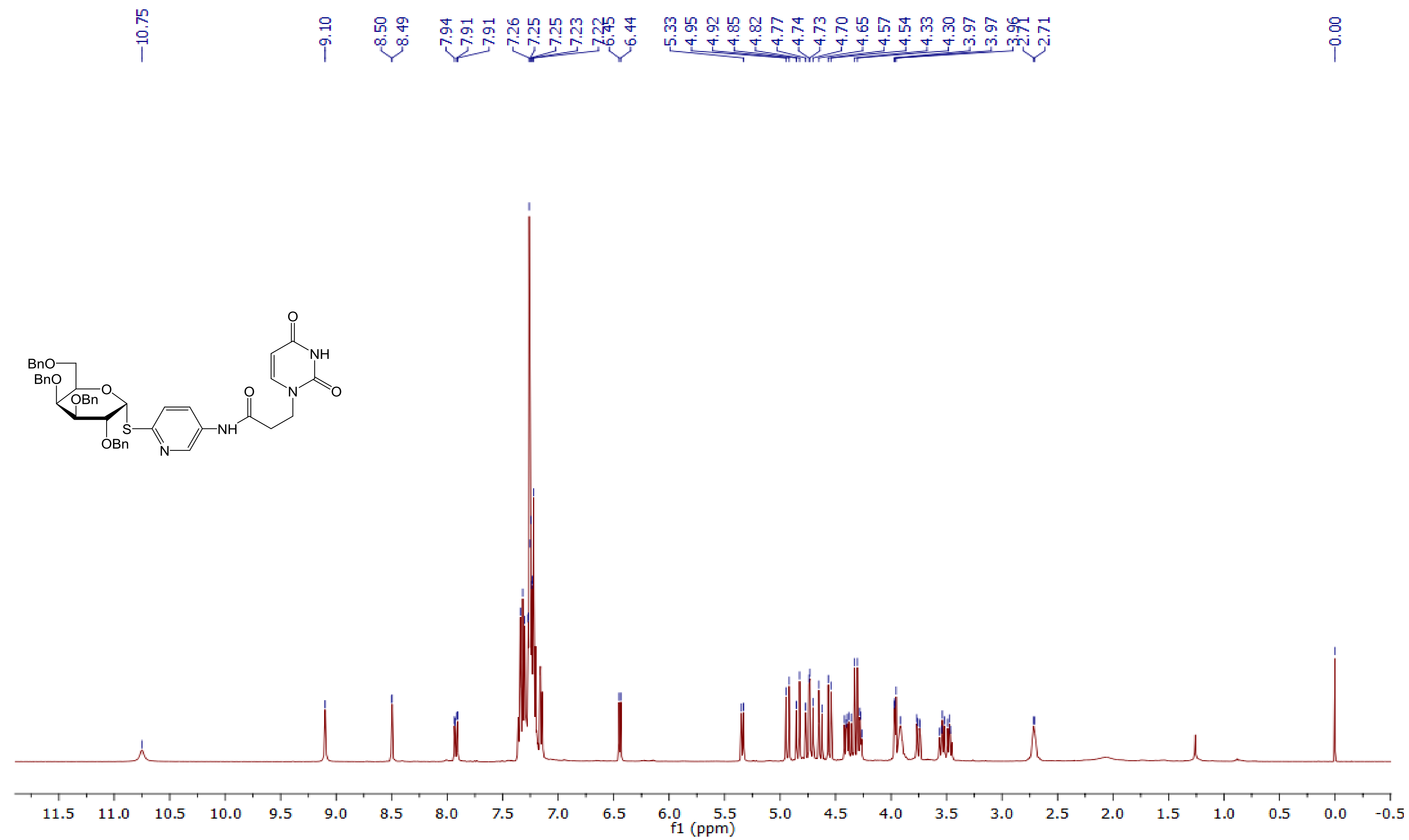

Fig. S23: ^{1}H NMR spectrum of glycoconjugate **34**

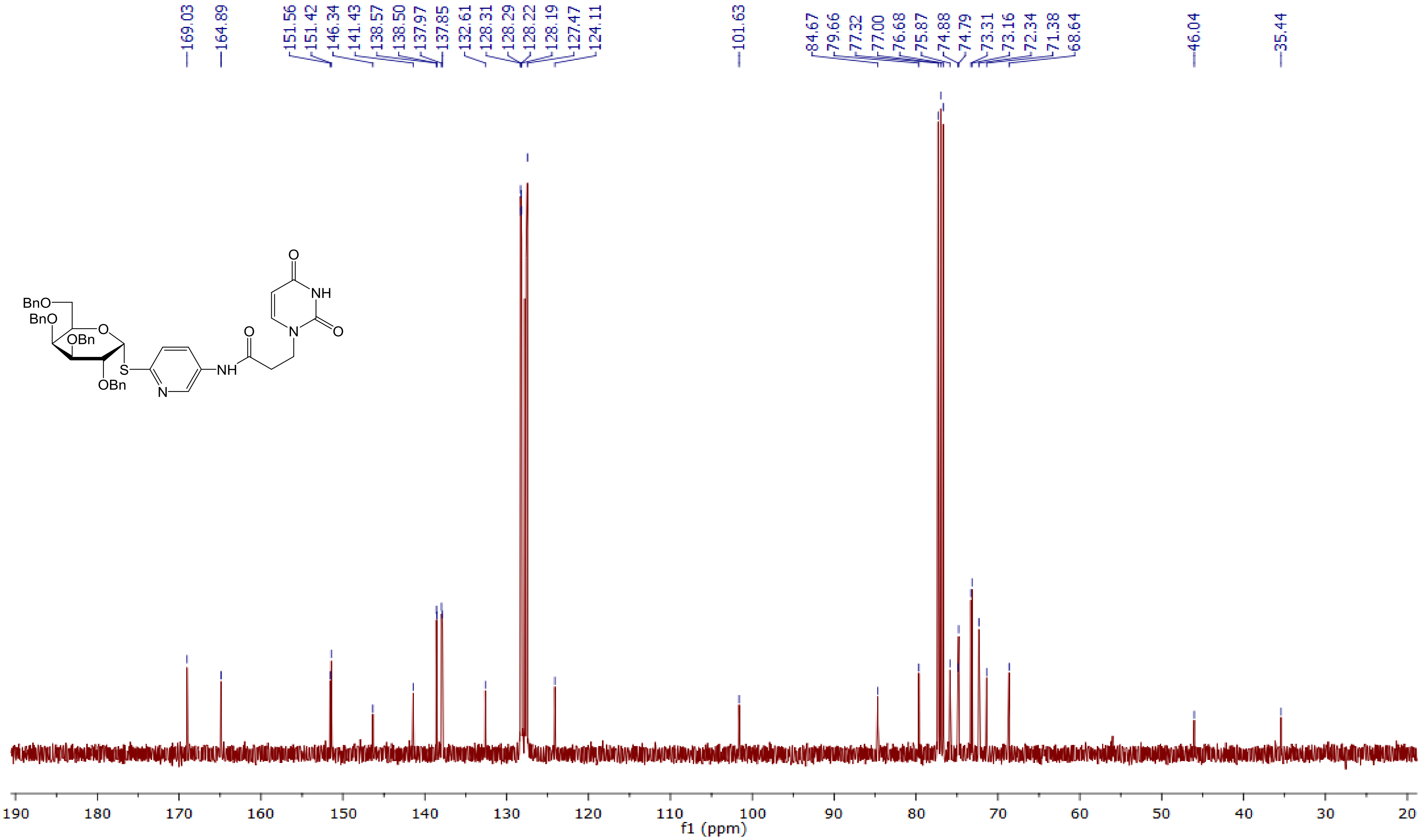

Fig. S24: ^{13}C NMR spectrum of glycoconjugate **34**

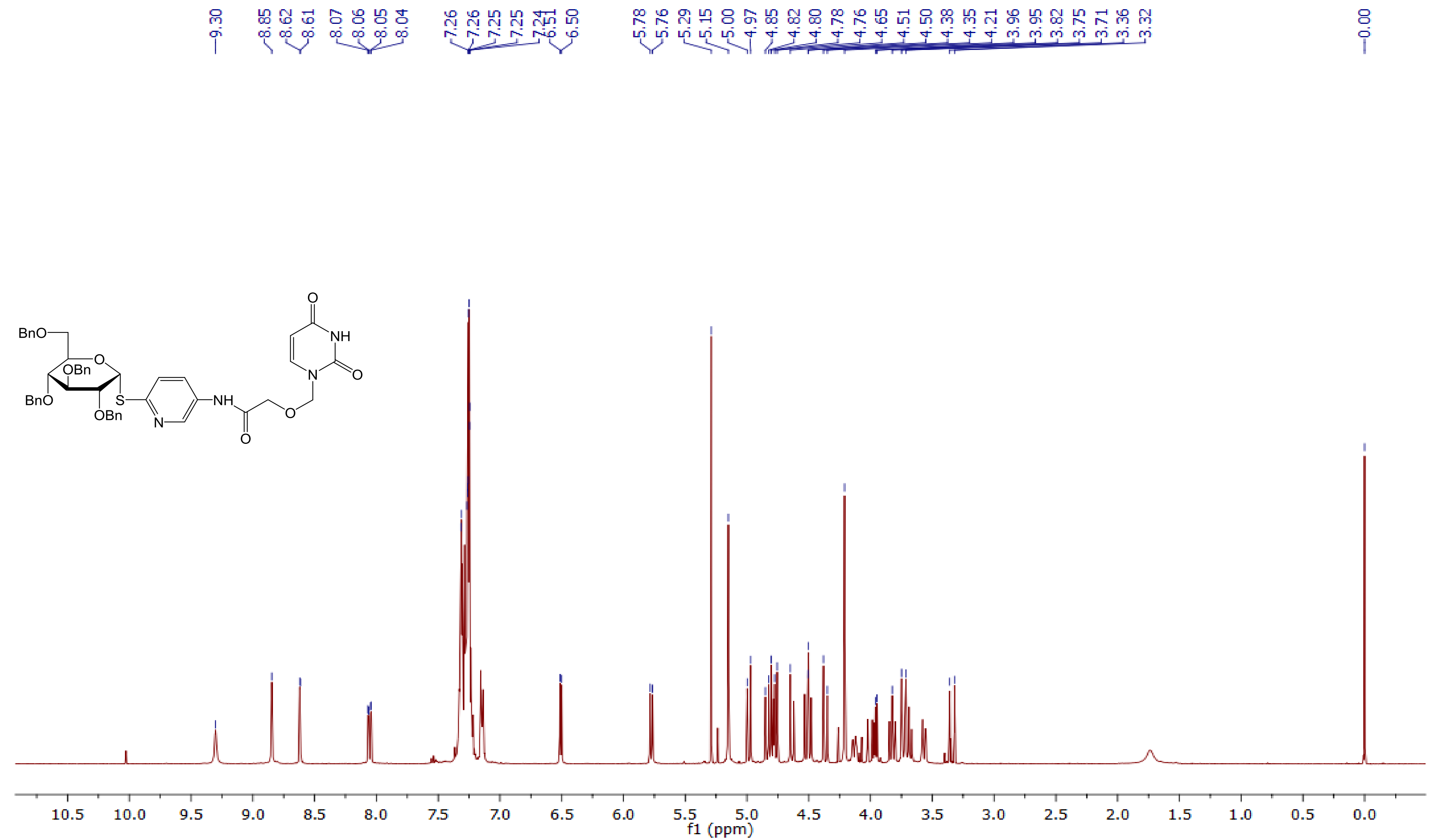

Fig. S25: ^{1}H NMR spectrum of glycoconjugate **35**

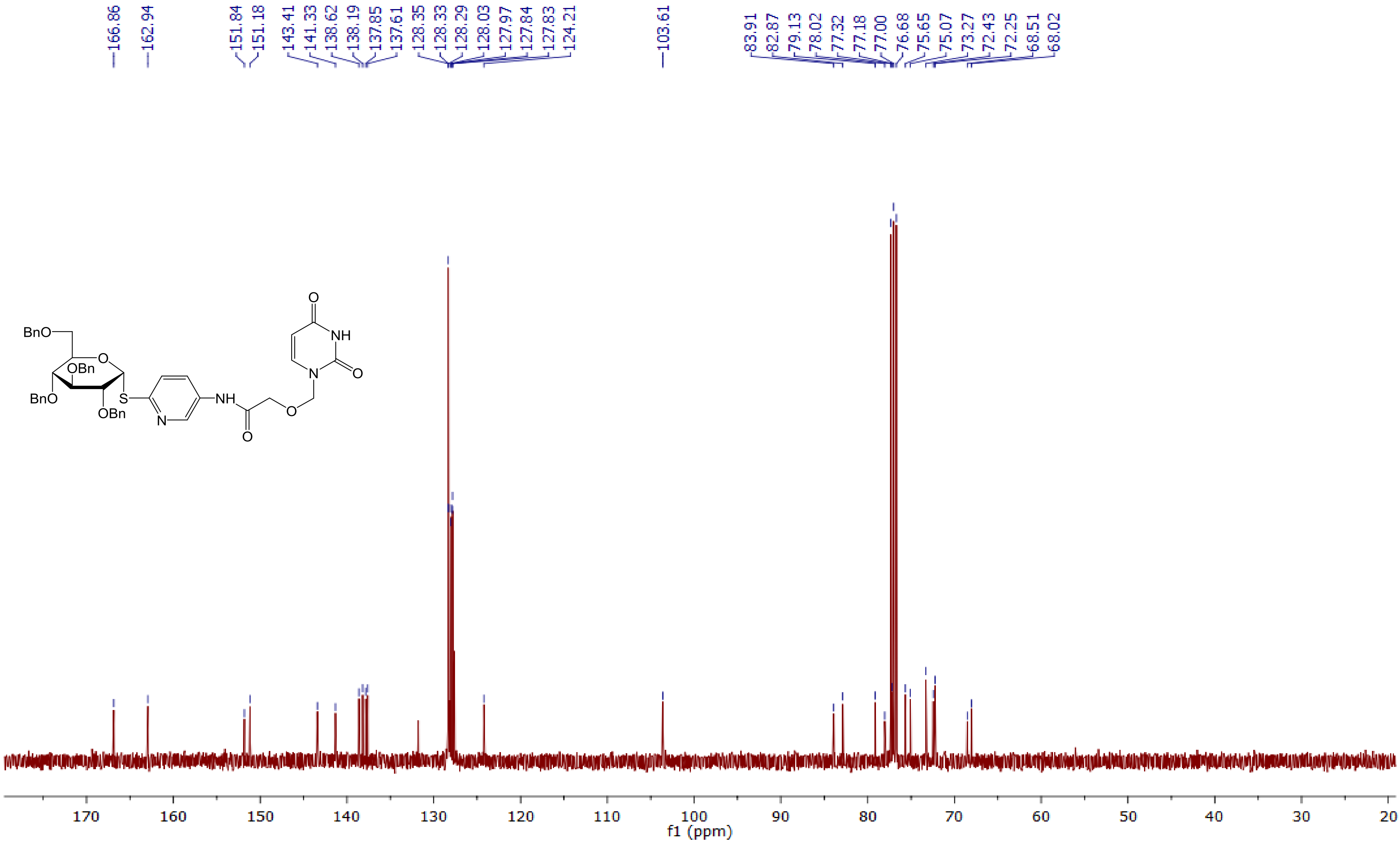

Fig. S26: ^{13}C NMR spectrum of glycoconjugate **35**

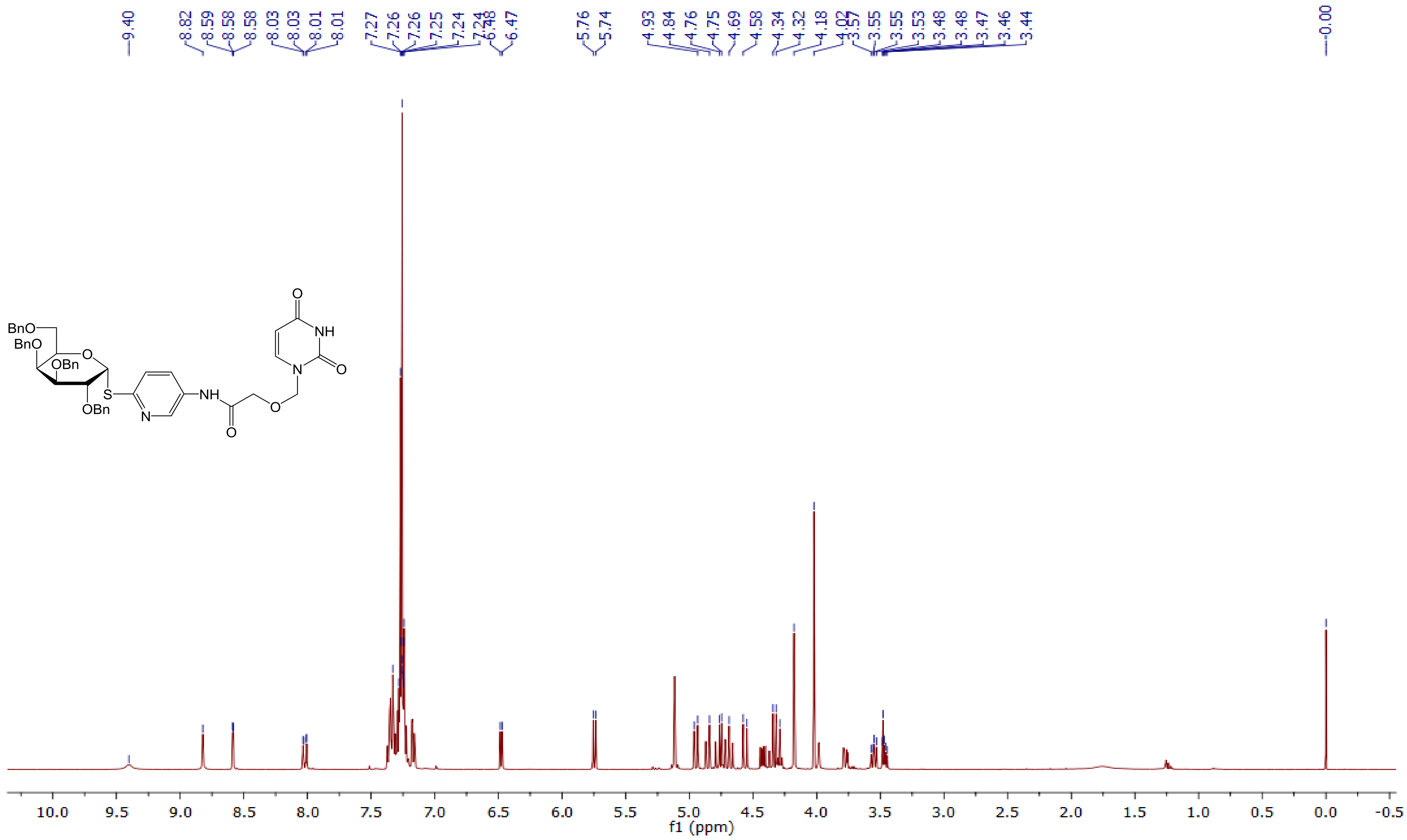

Fig. S27: ^{1}H NMR spectrum of glycoconjugate **36**

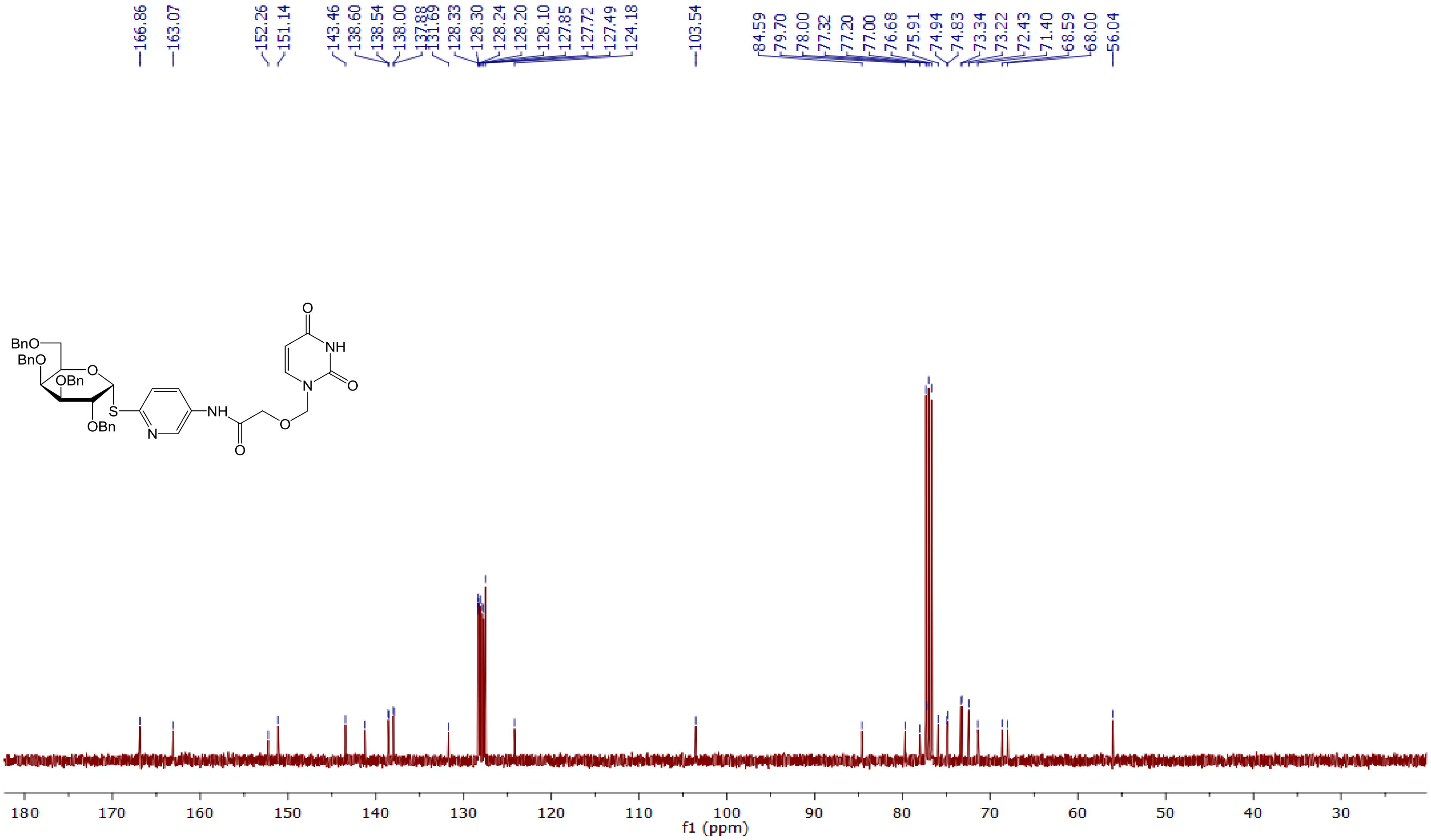

Fig. S28: ^{13}C NMR spectrum of glycoconjugate **36**

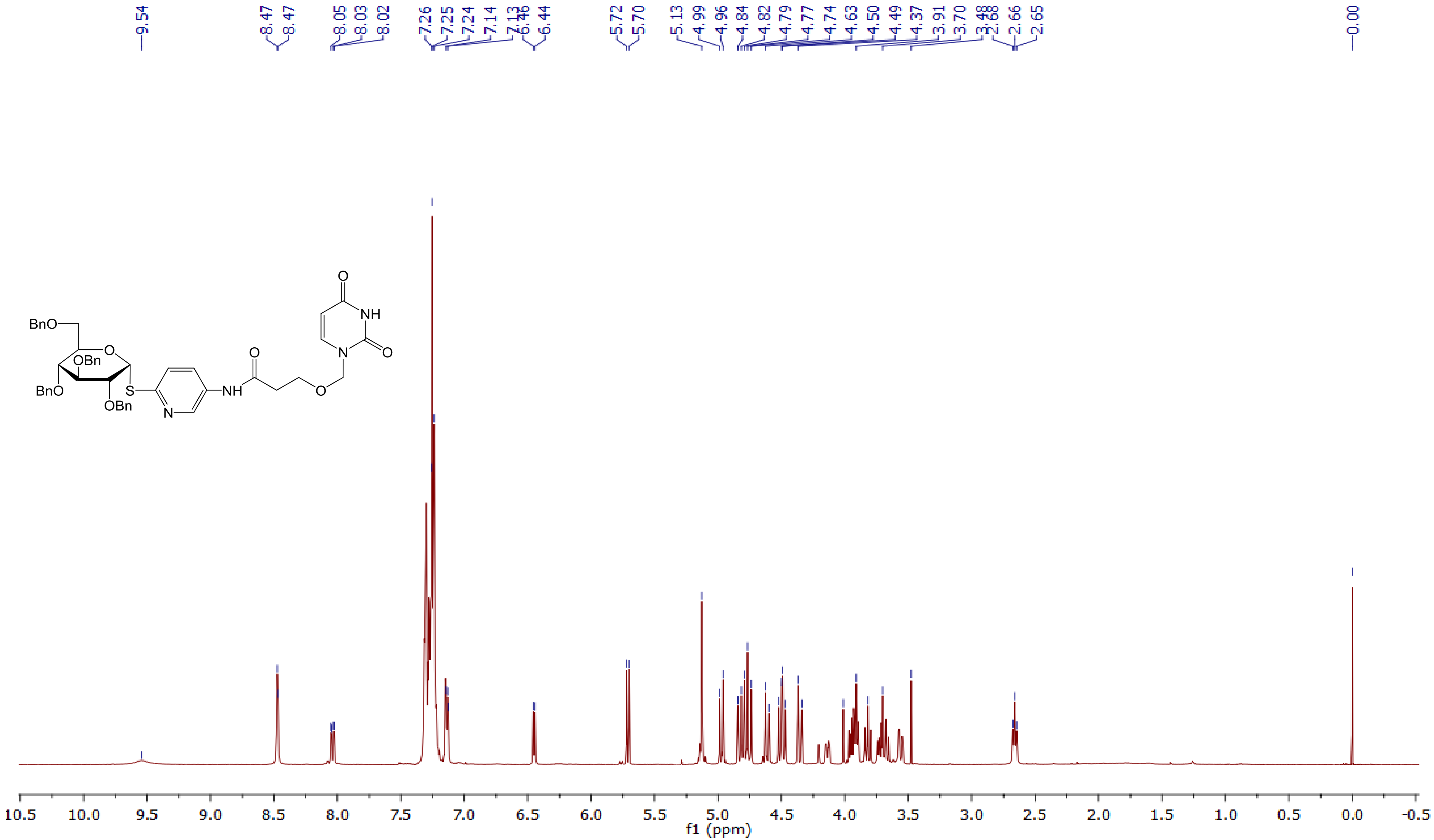

Fig. S29: ^{1}H NMR spectrum of glycoconjugate **37**

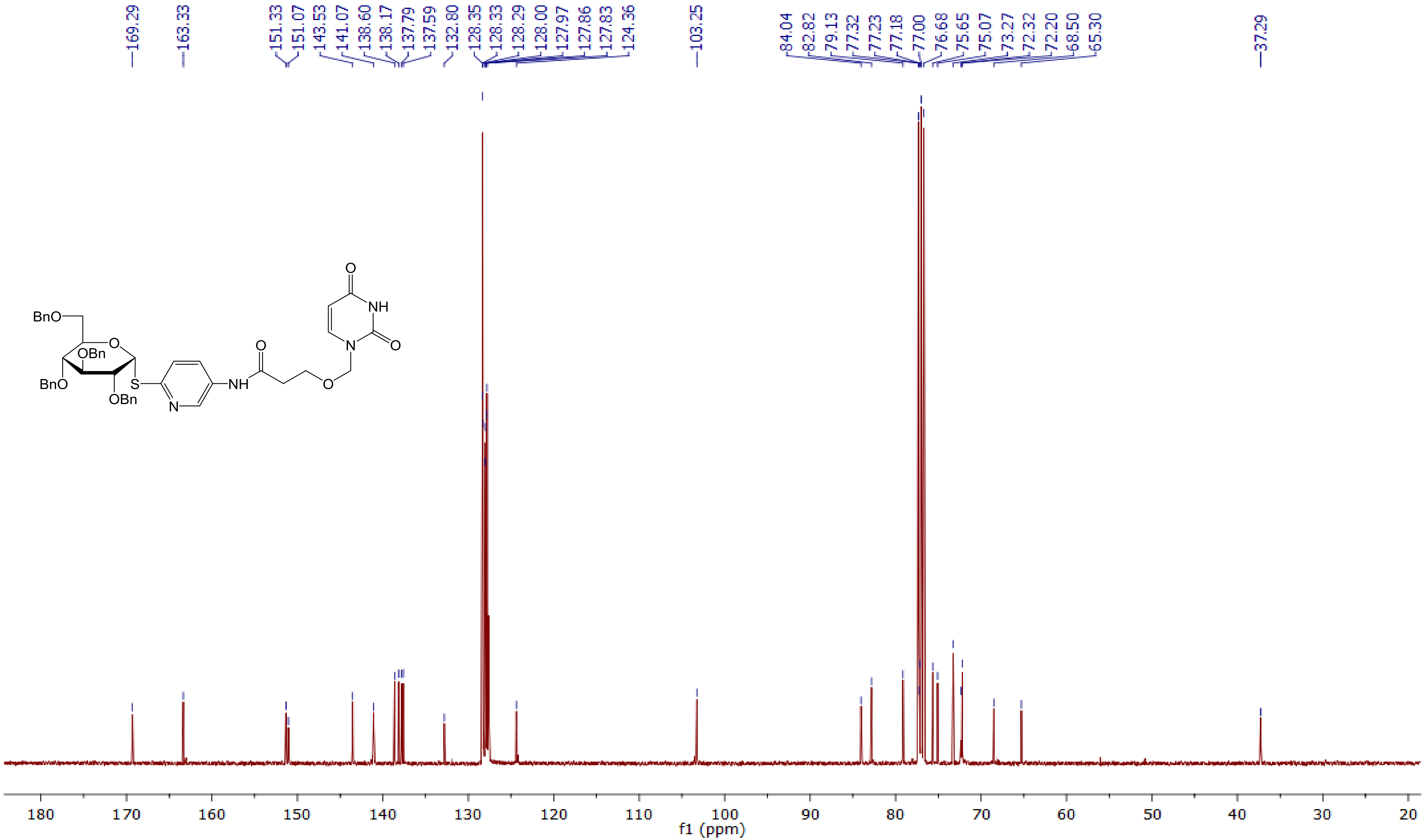

Fig. S30: ^{13}C NMR spectrum of glycoconjugate **37**

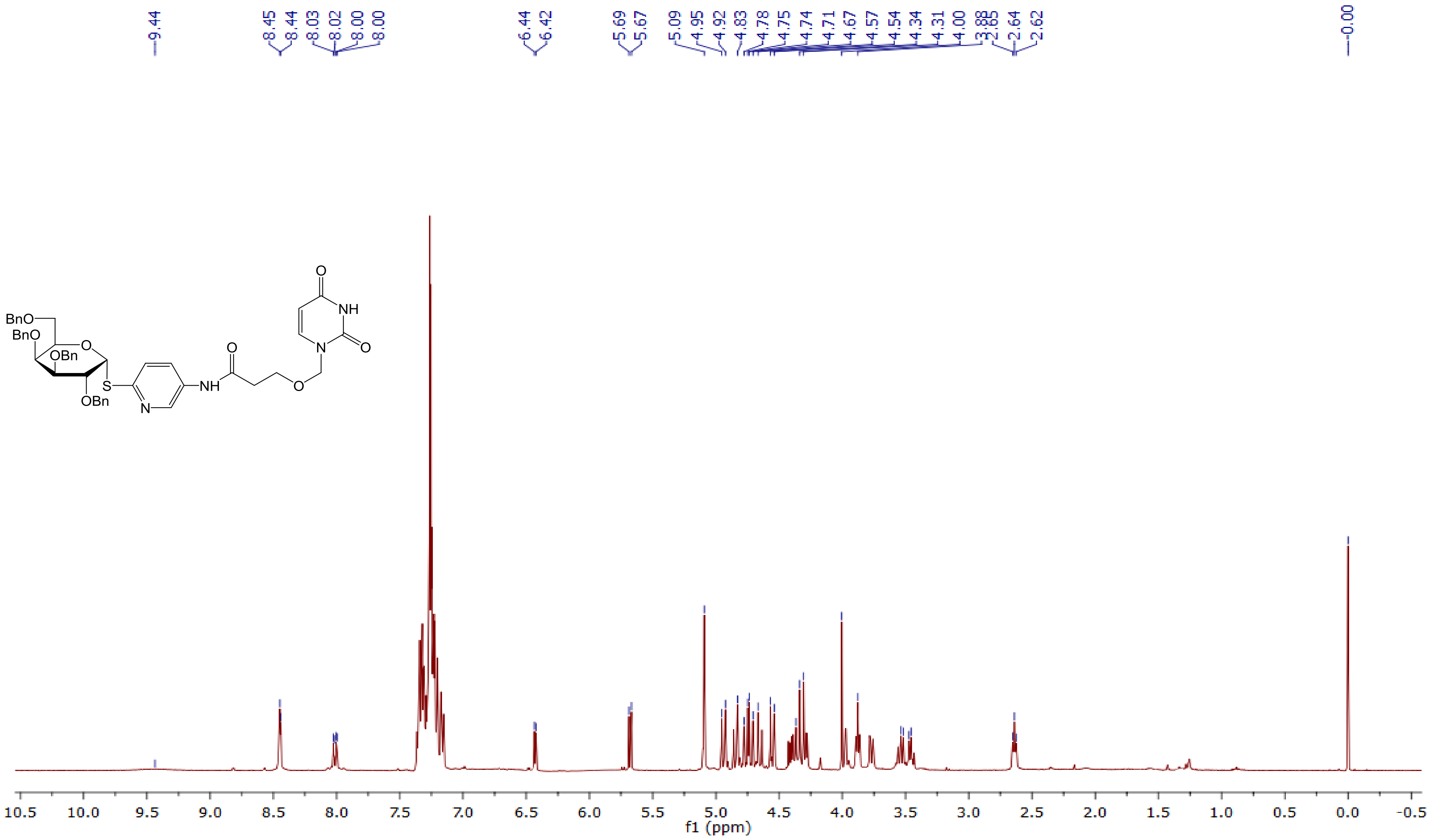

Fig. S31: ^{1}H NMR spectrum of glycoconjugate **38**

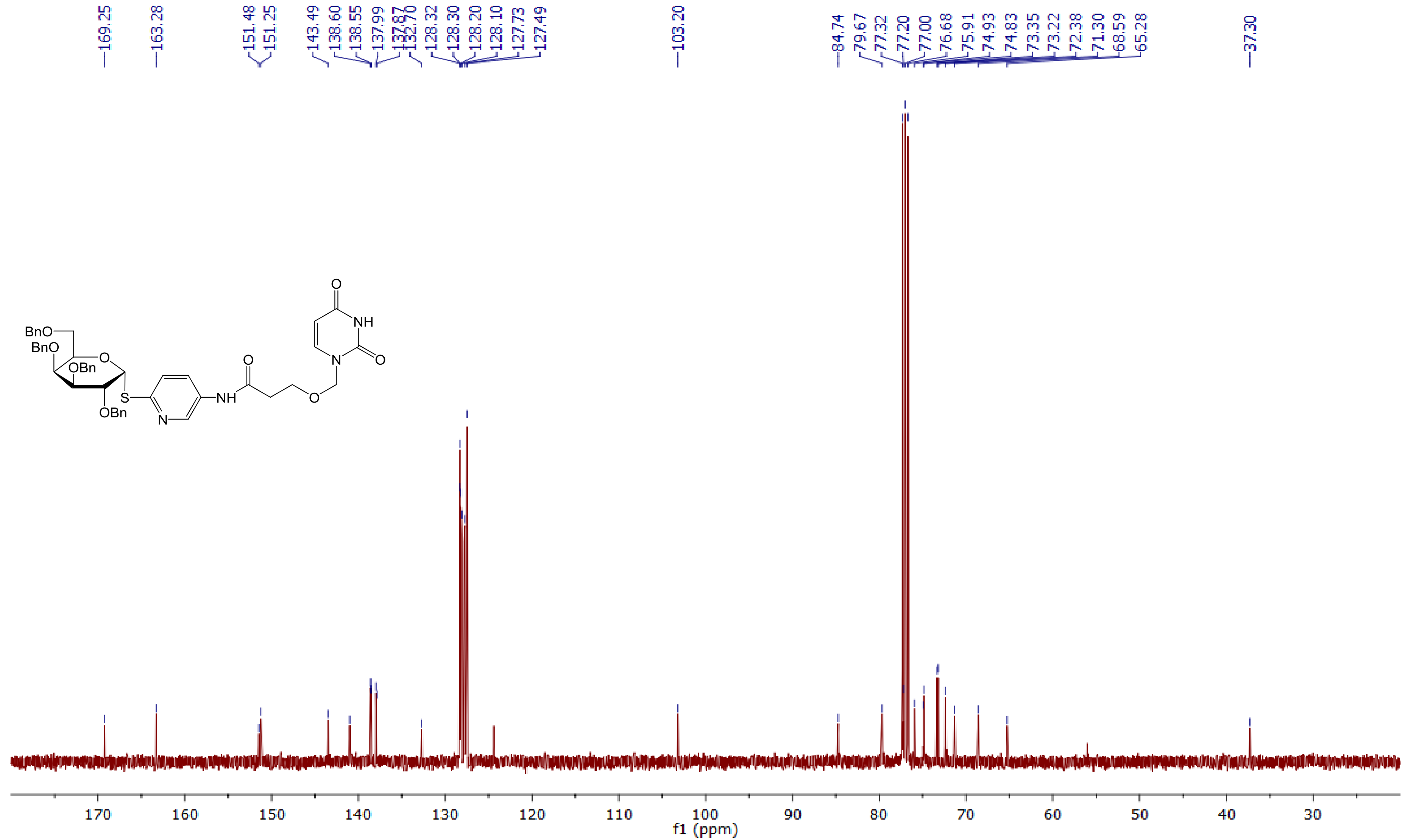

Fig. S32: ^{13}C NMR spectrum of glycoconjugate **38**

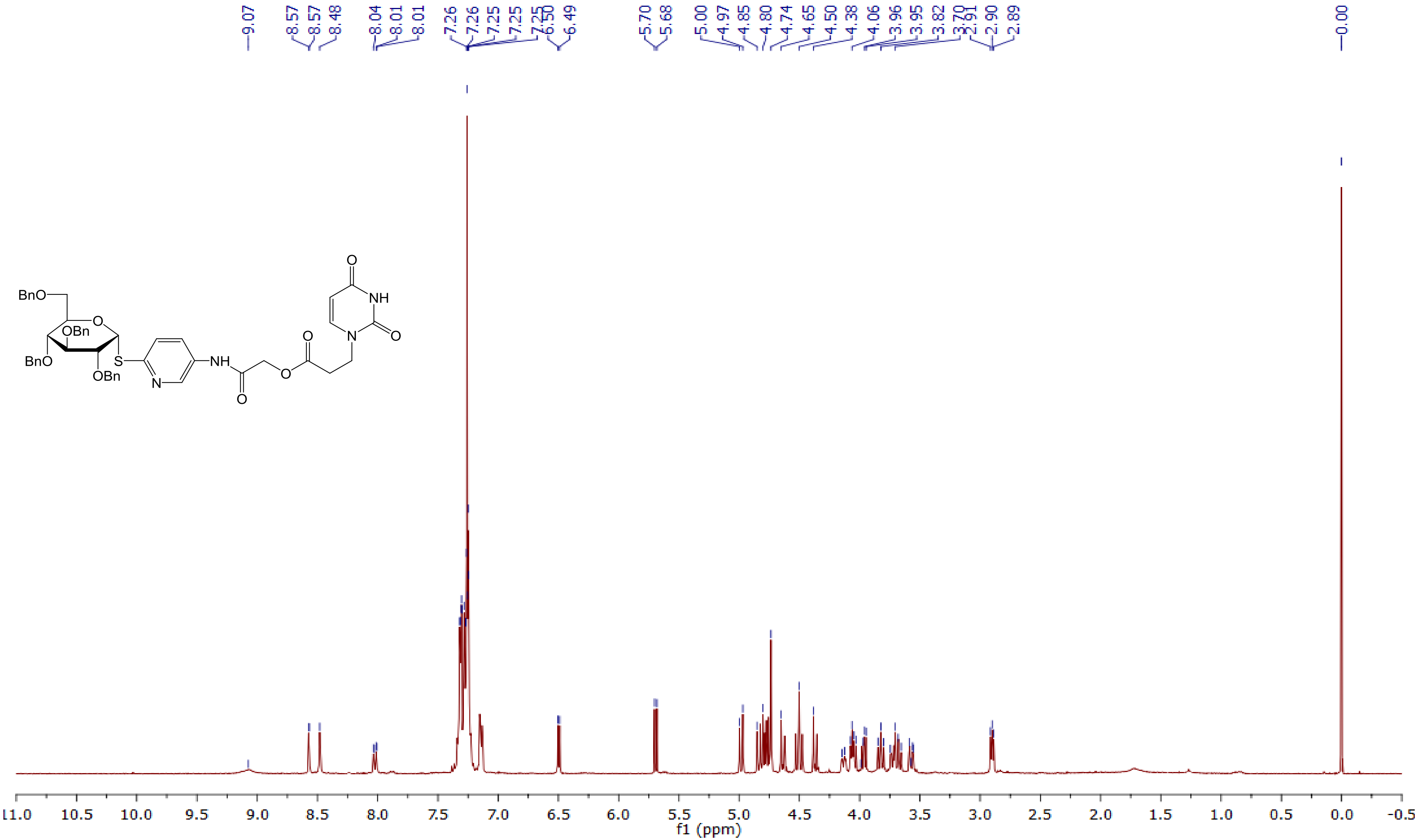

Fig. S33: ^{1}H NMR spectrum of glycoconjugate **39**.

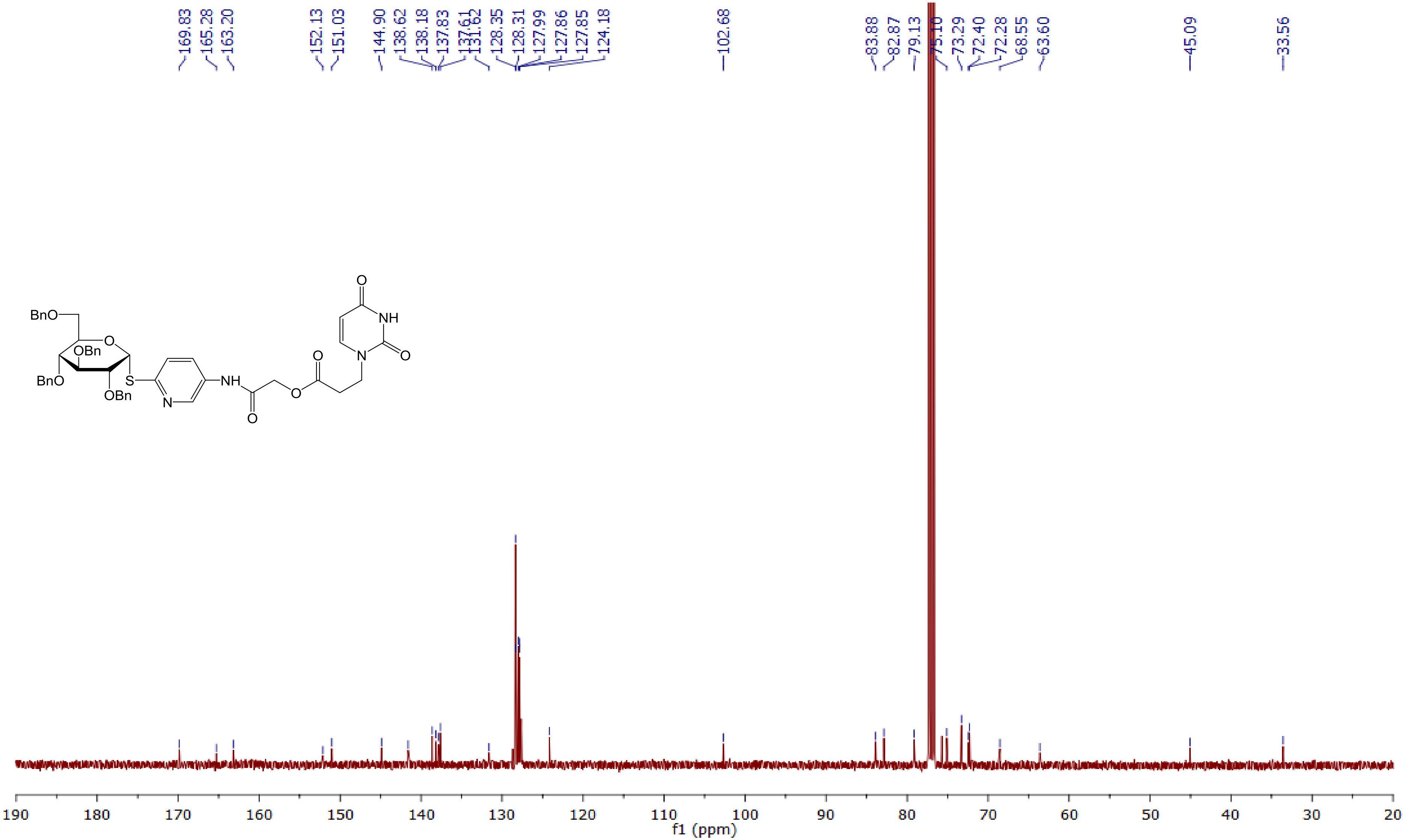

Fig. S34: ^{13}C NMR spectrum of glycoconjugate **39**

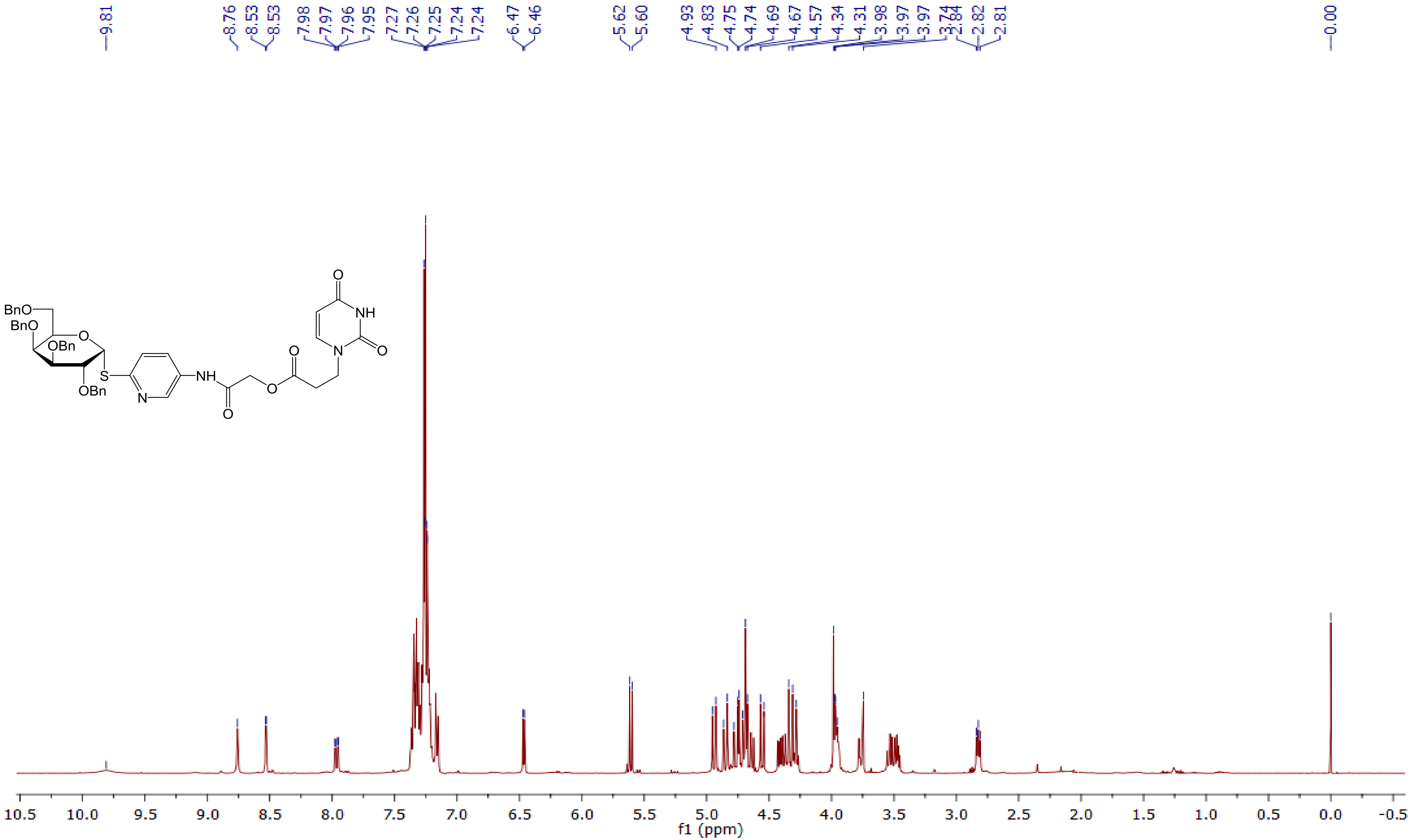

Fig. S35: ^{1}H NMR spectrum of glycoconjugate **40**

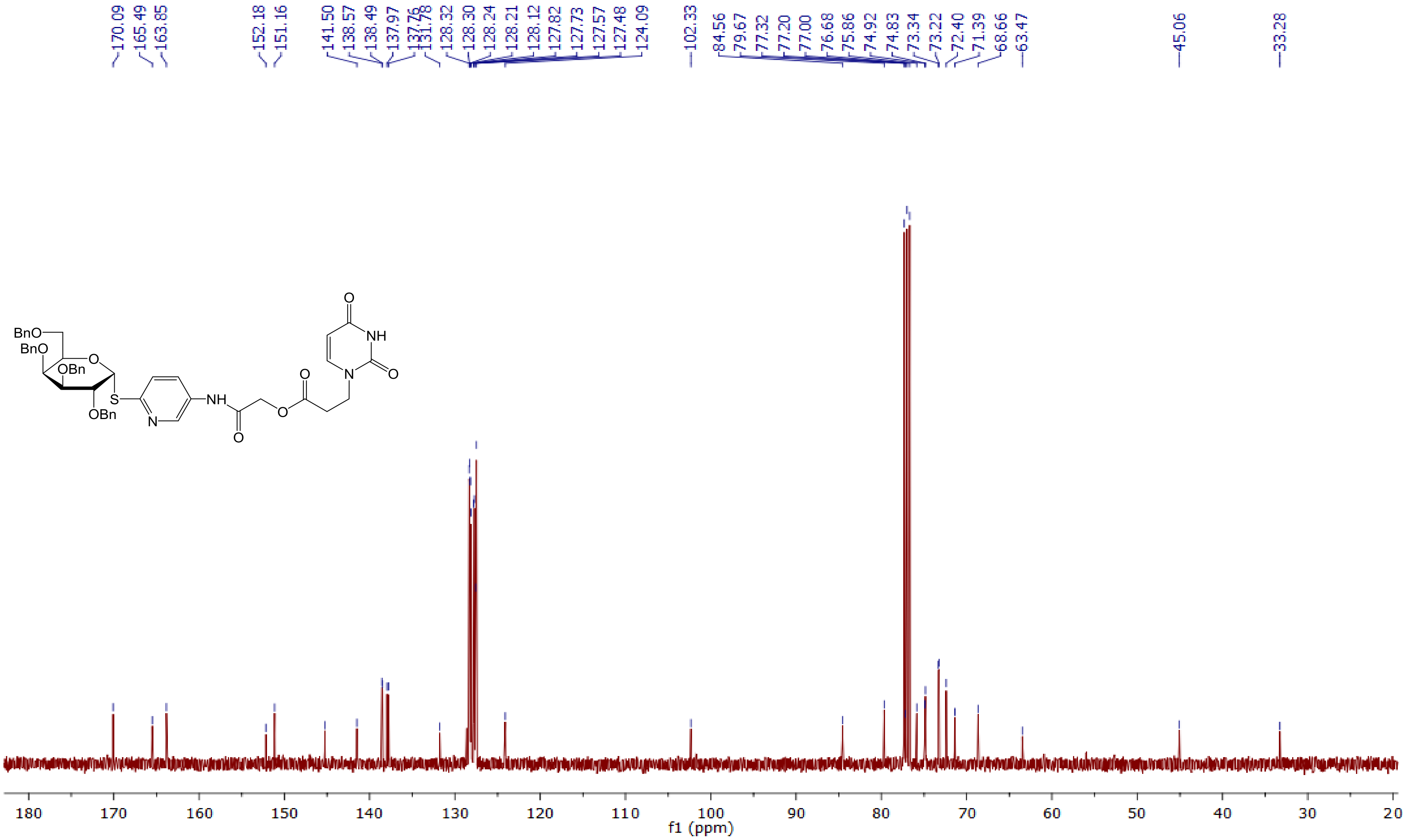

Fig. S36: ^{13}C NMR spectrum of glycoconjugate **40**

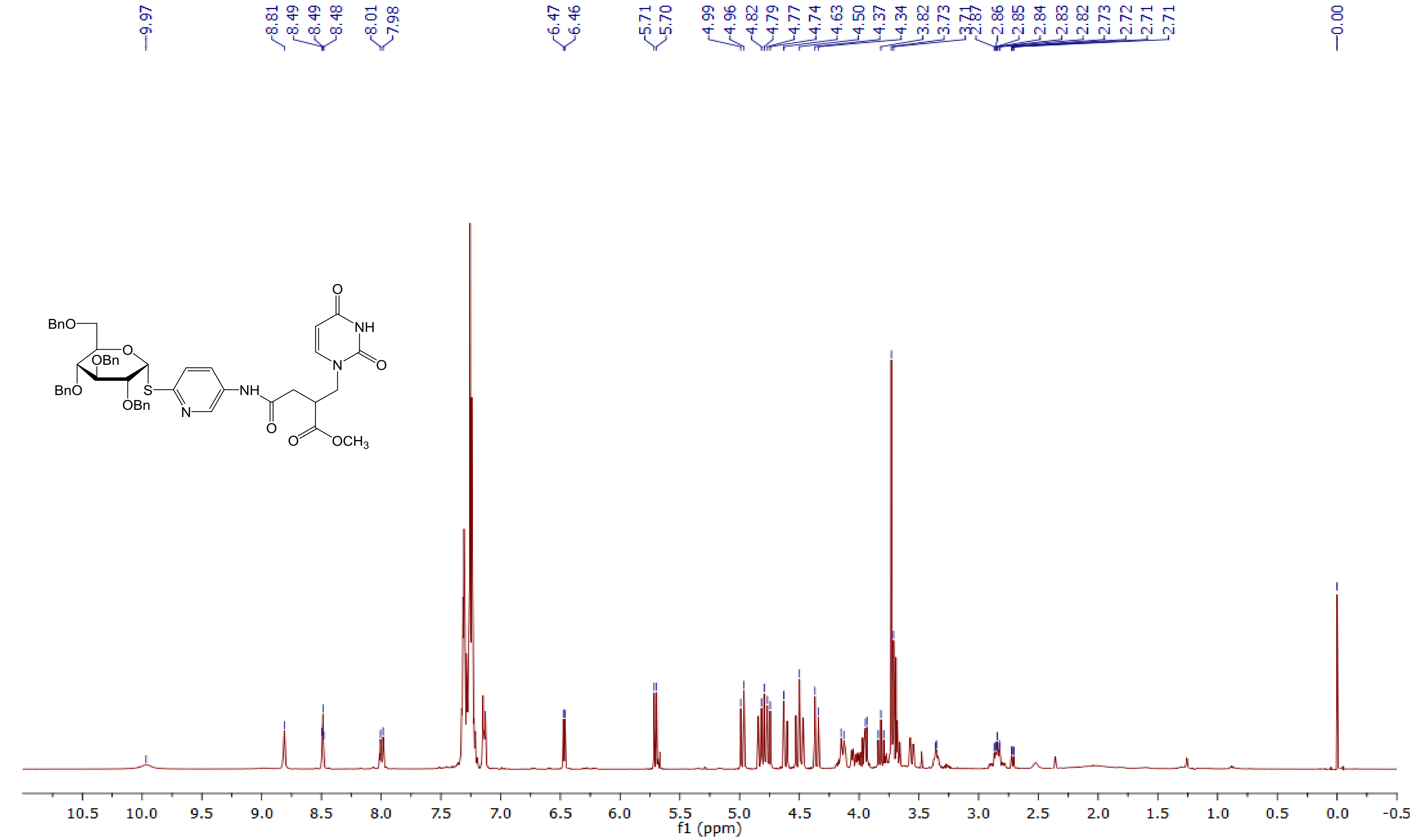

Fig. S37: ^{1}H NMR spectrum of glycoconjugate **41**

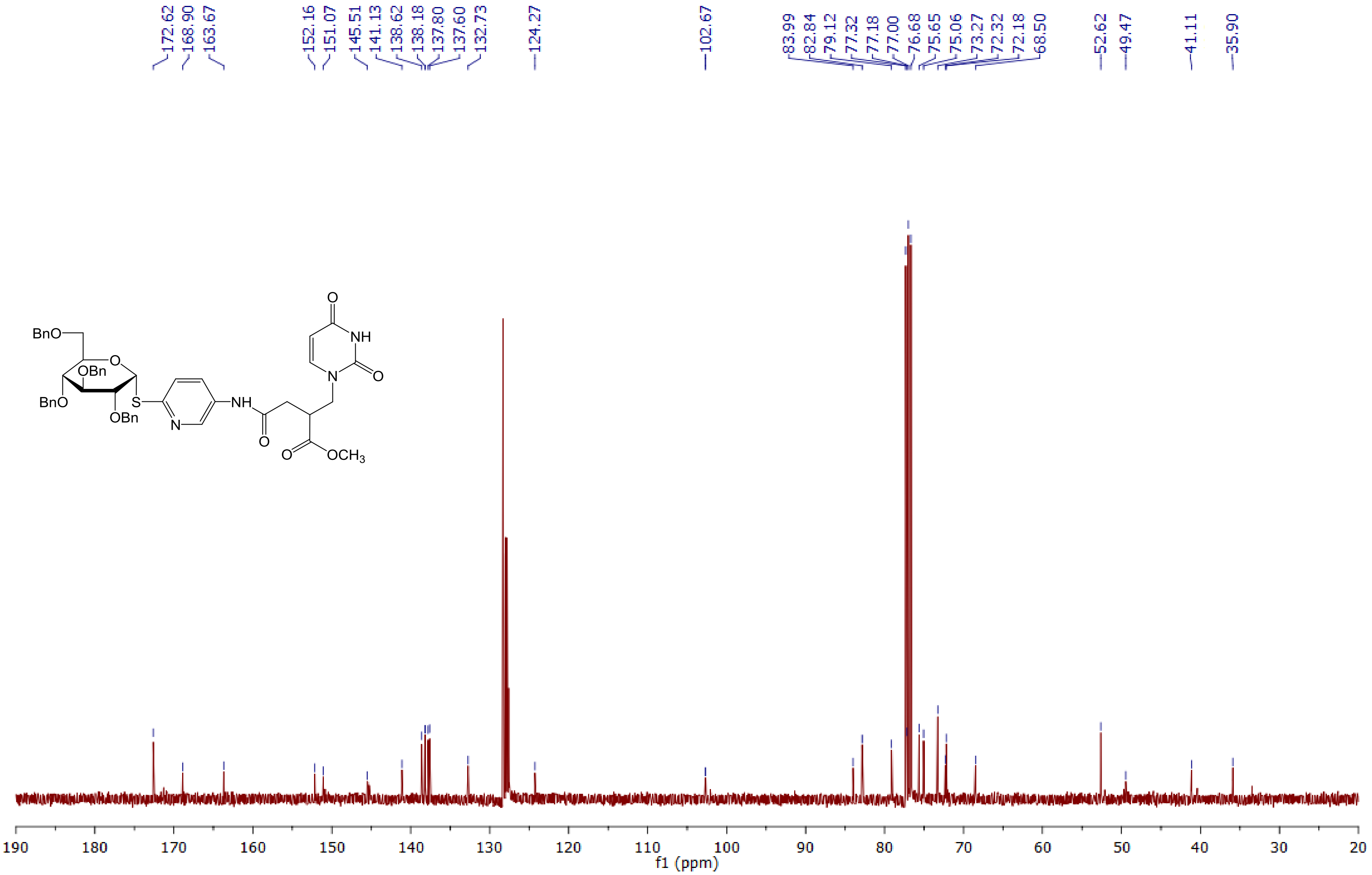

Fig. S38: ^{13}C NMR spectrum of glycoconjugate **41**

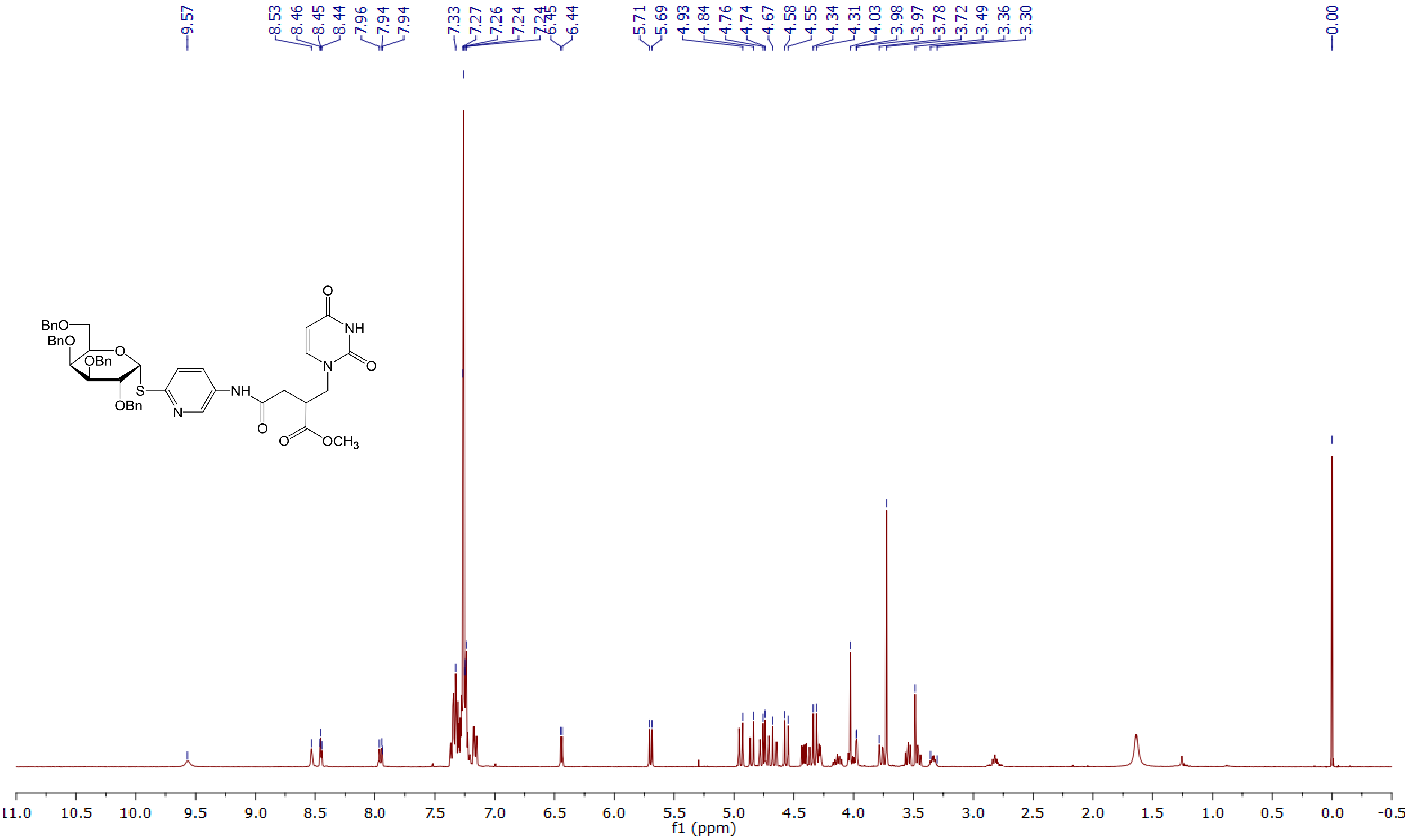

Fig. S39: ^{1}H NMR spectrum of glycoconjugate **42**

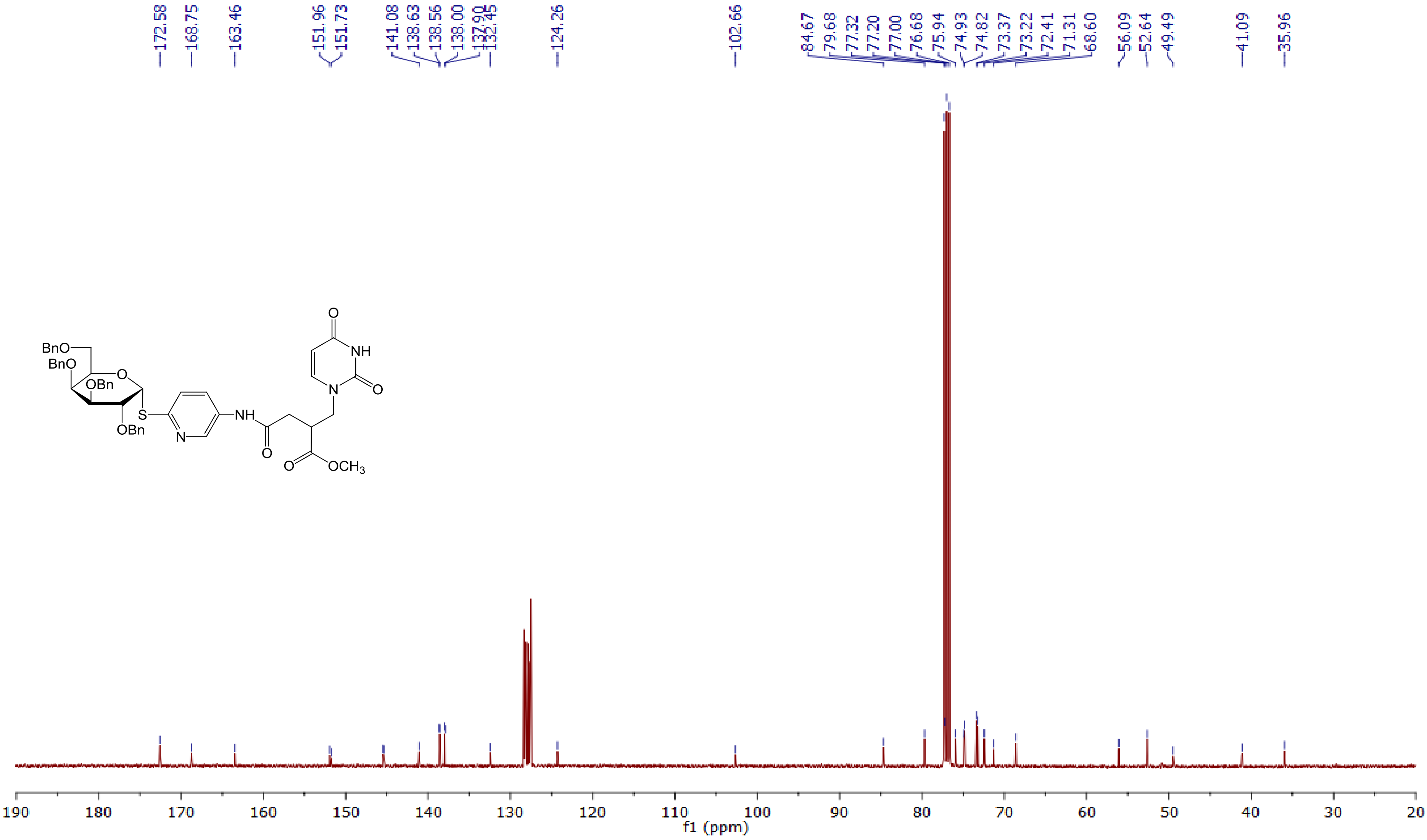

Fig. S40: ^{13}C NMR spectrum of glycoconjugate **42**

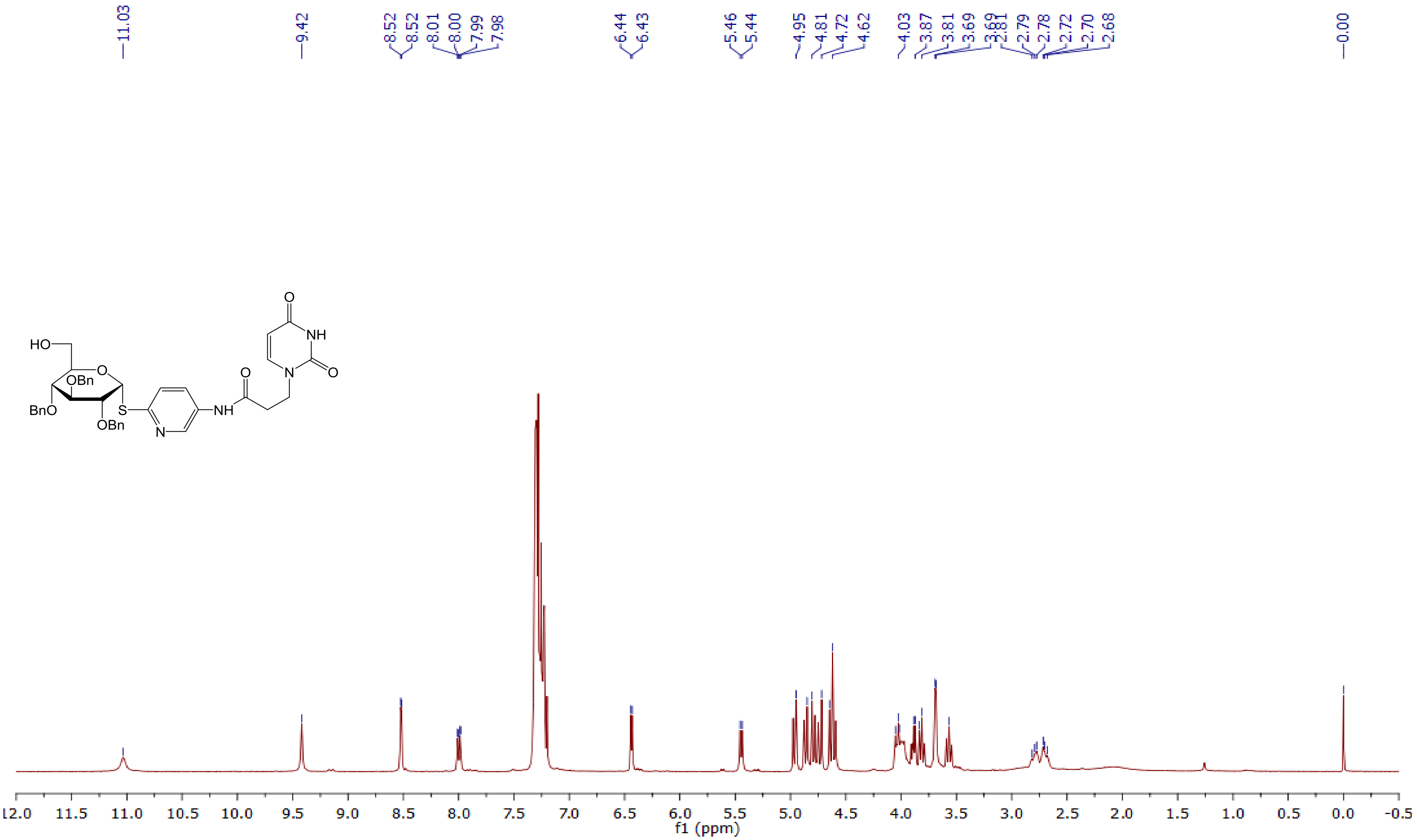

Fig. S41: ^{1}H NMR spectrum of glycoconjugate **43**

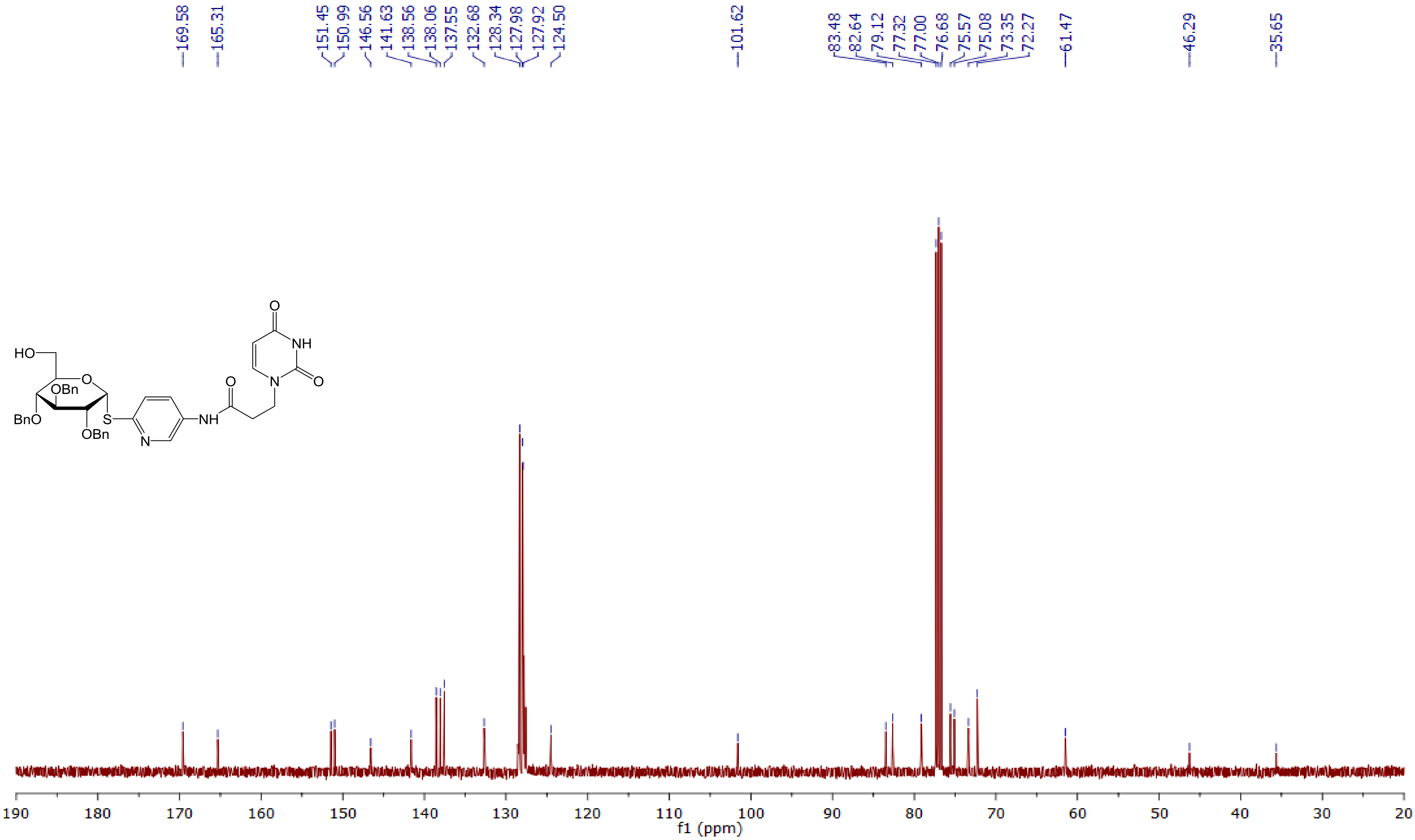

Fig. S42: ^{13}C NMR spectrum of glycoconjugate **43**

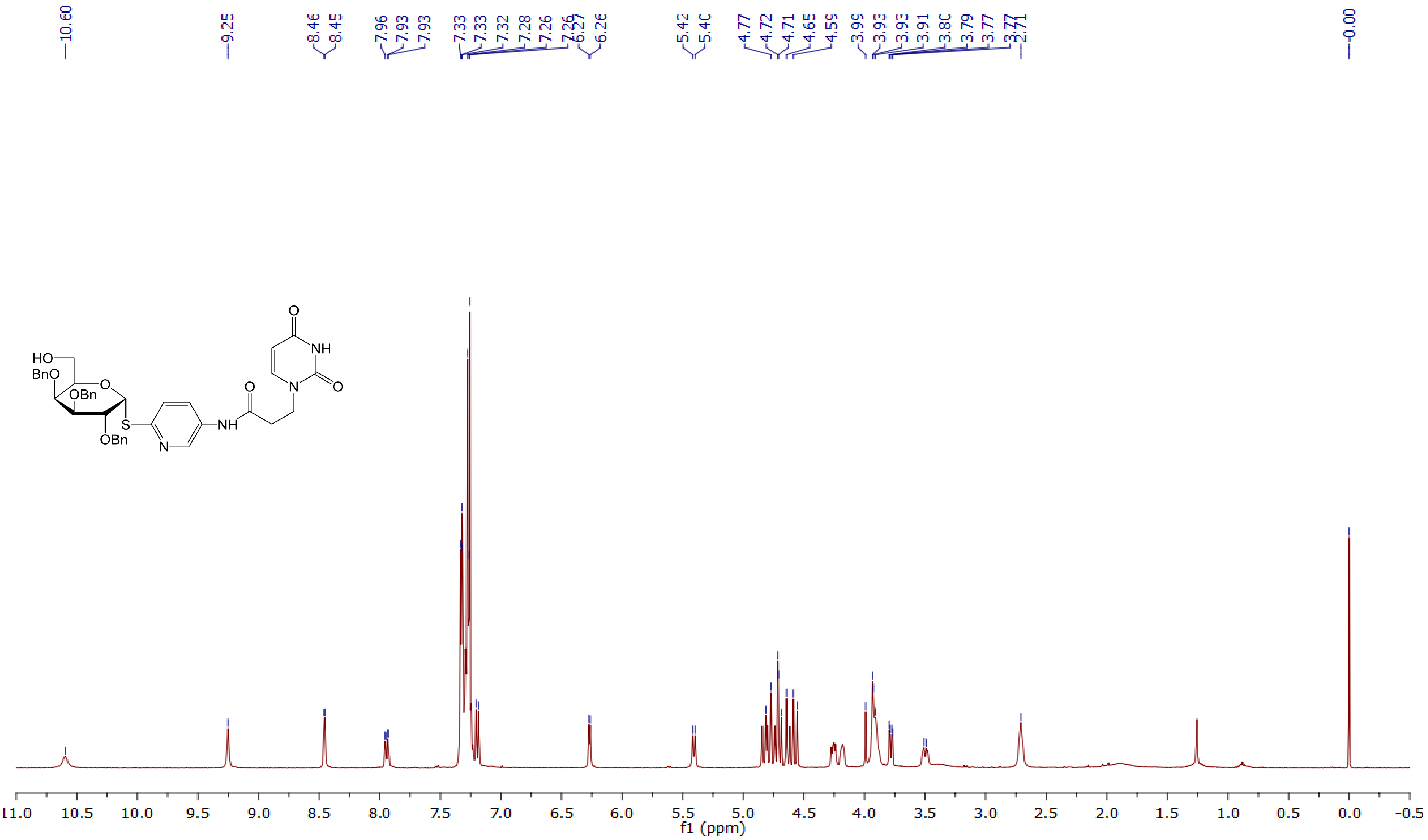

Fig. S43: ^{1}H NMR spectrum of glycoconjugate **44**

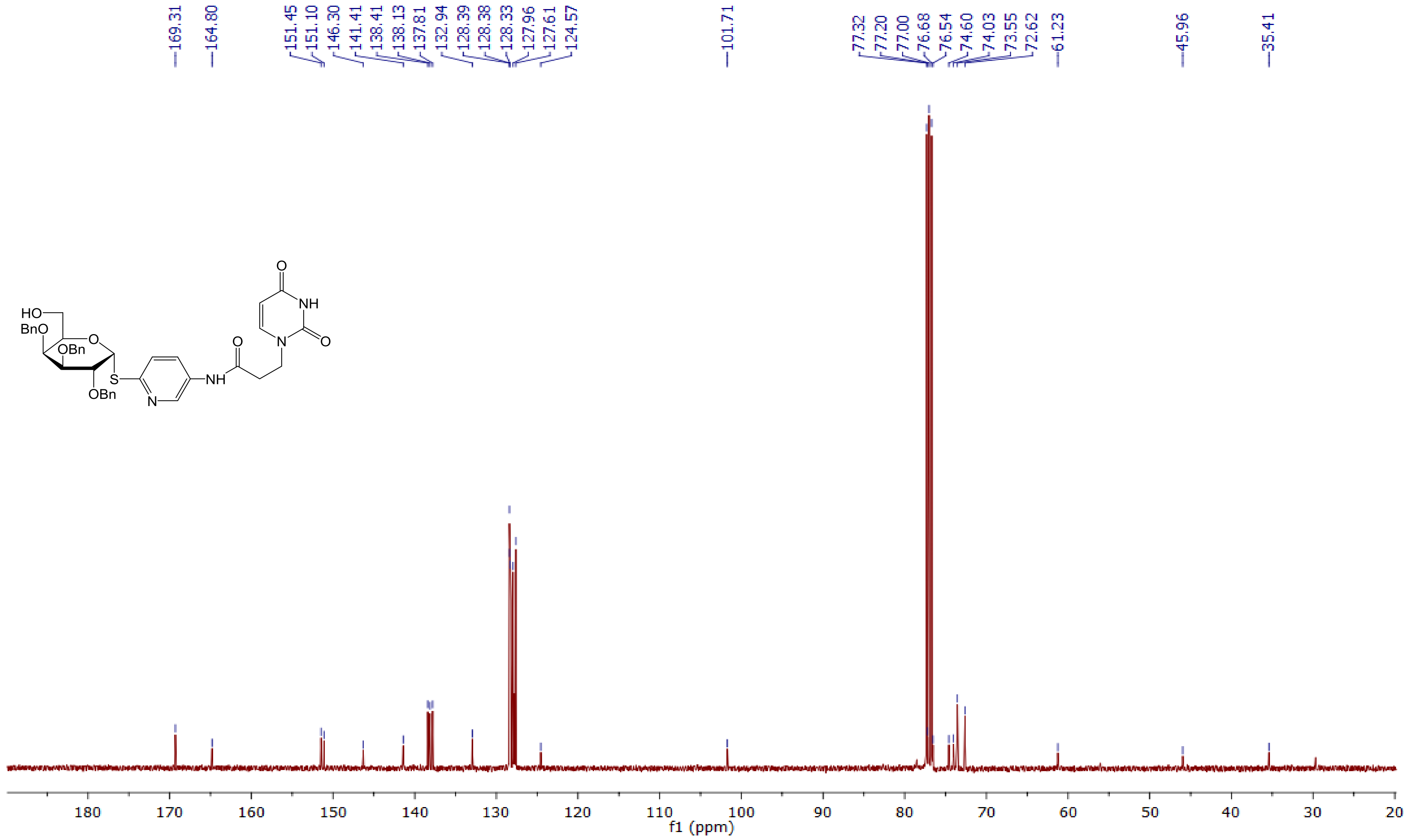

Fig. S44: ^{13}C NMR spectrum of glycoconjugate **44**

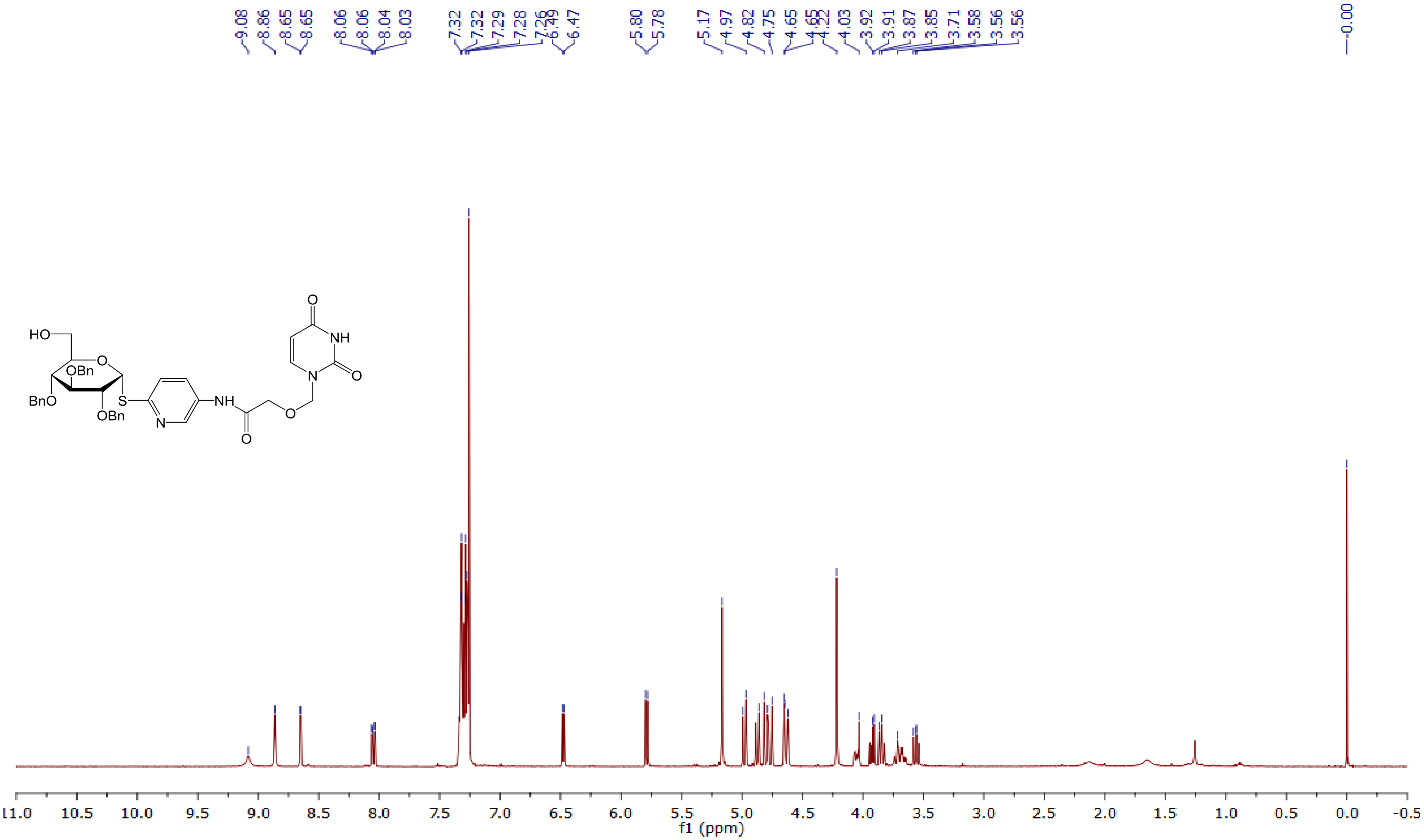

Fig. S45: ^{1}H NMR spectrum of glycoconjugate **45**

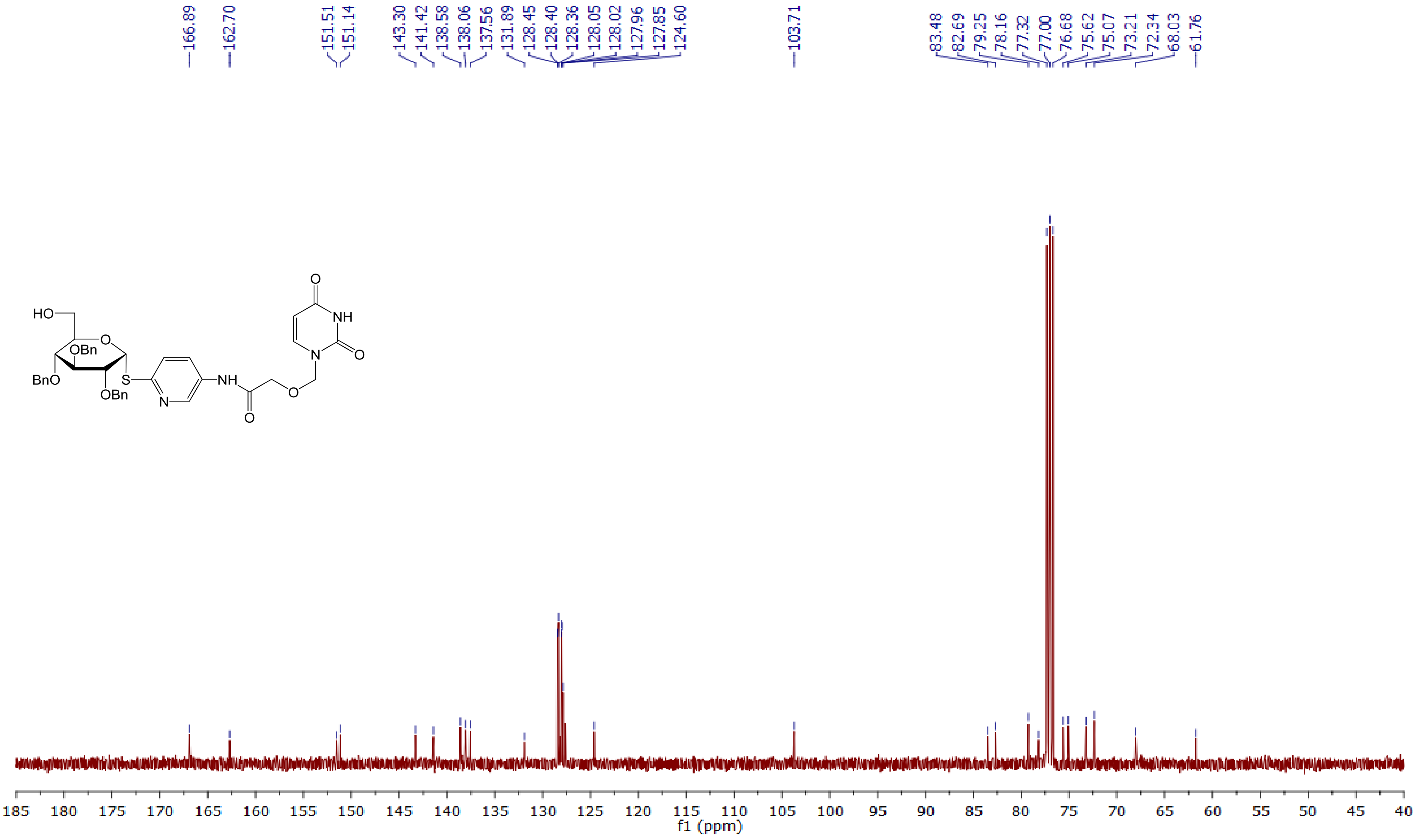

Fig. S46: ^{13}C NMR spectrum of glycoconjugate **45**

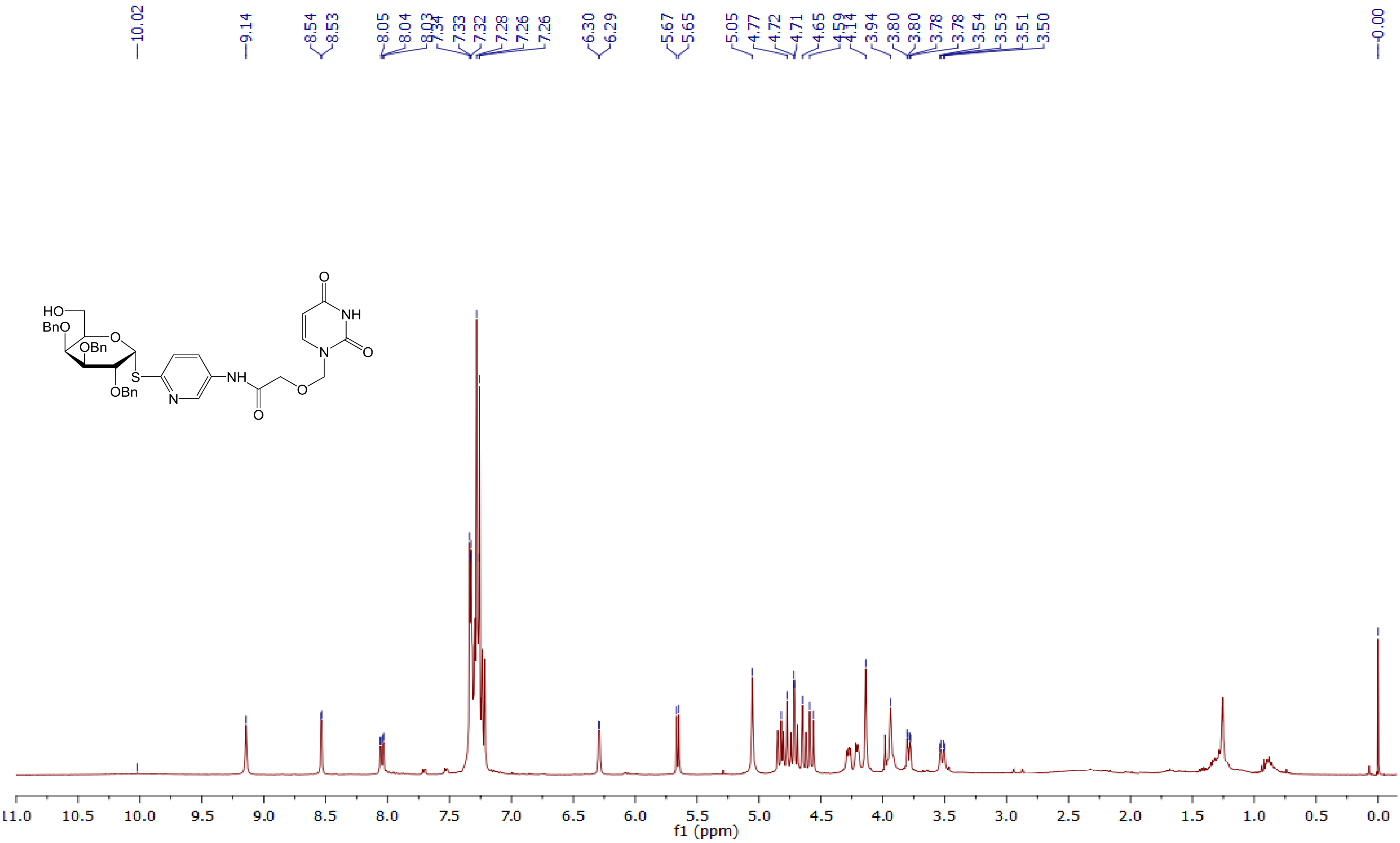

Fig. S47: ^{1}H NMR spectrum of glycoconjugate **46**

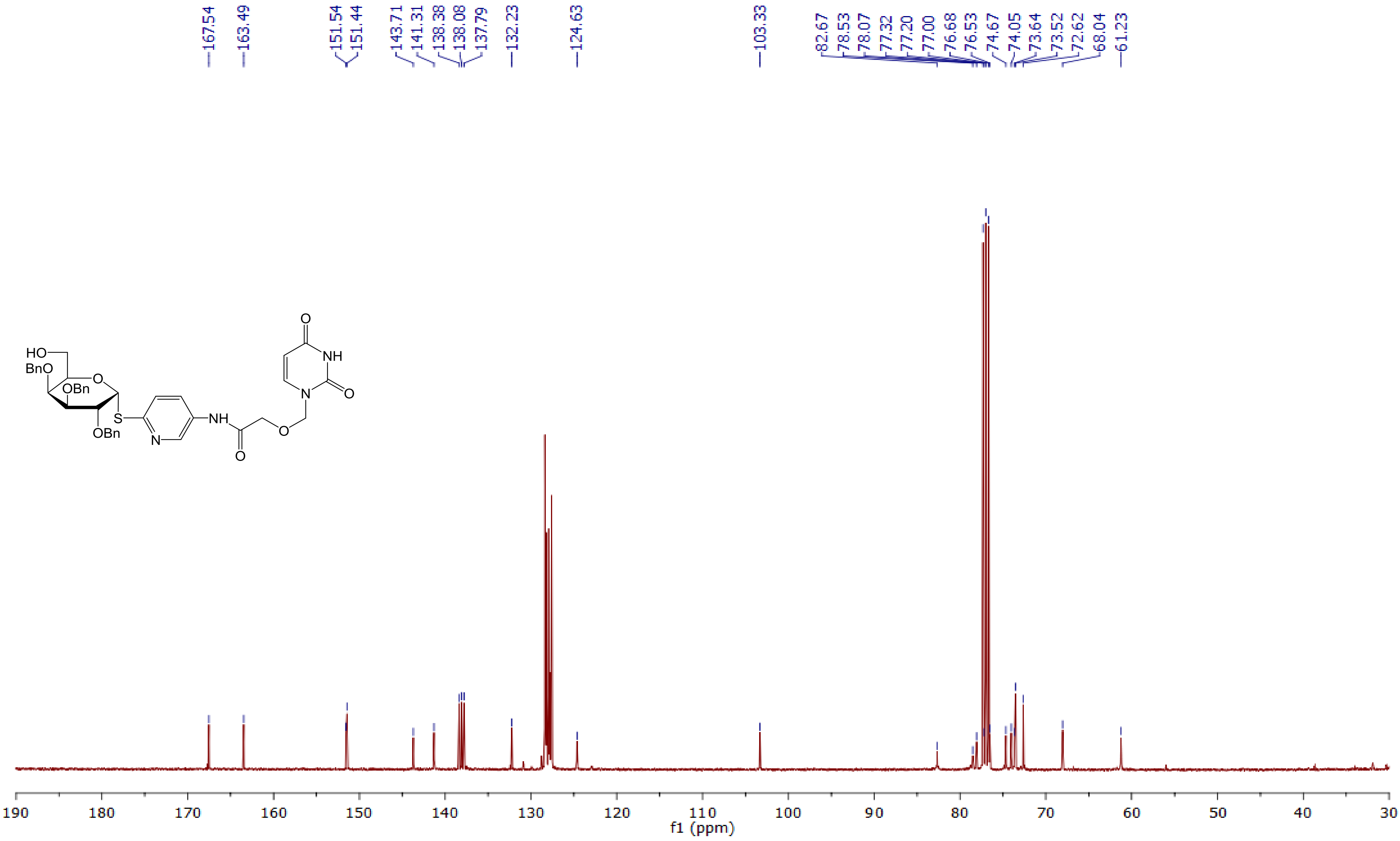

Fig. S48: ^{13}C NMR spectrum of glycoconjugate **46**

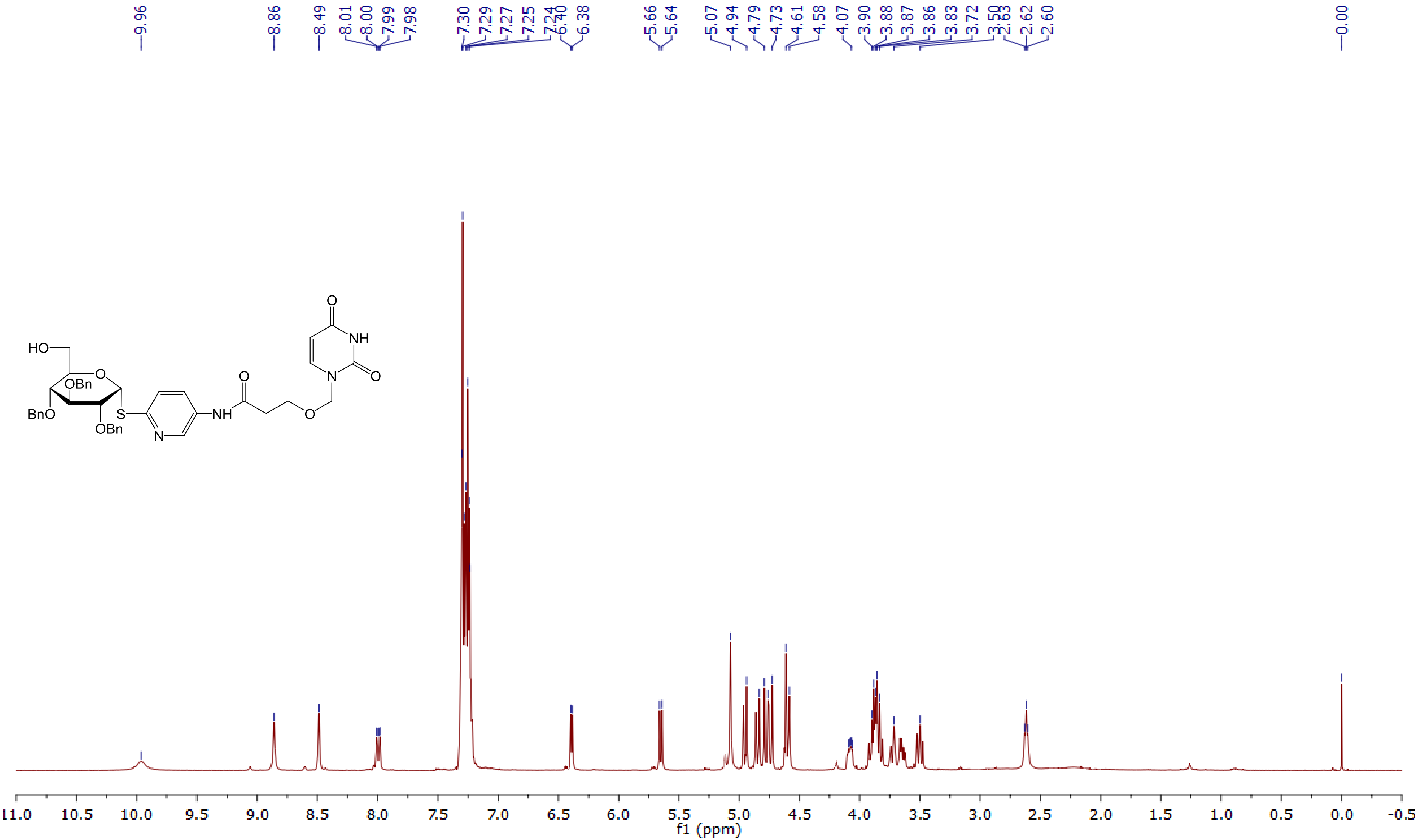

Fig. S49: ^{1}H NMR spectrum of glycoconjugate **47**

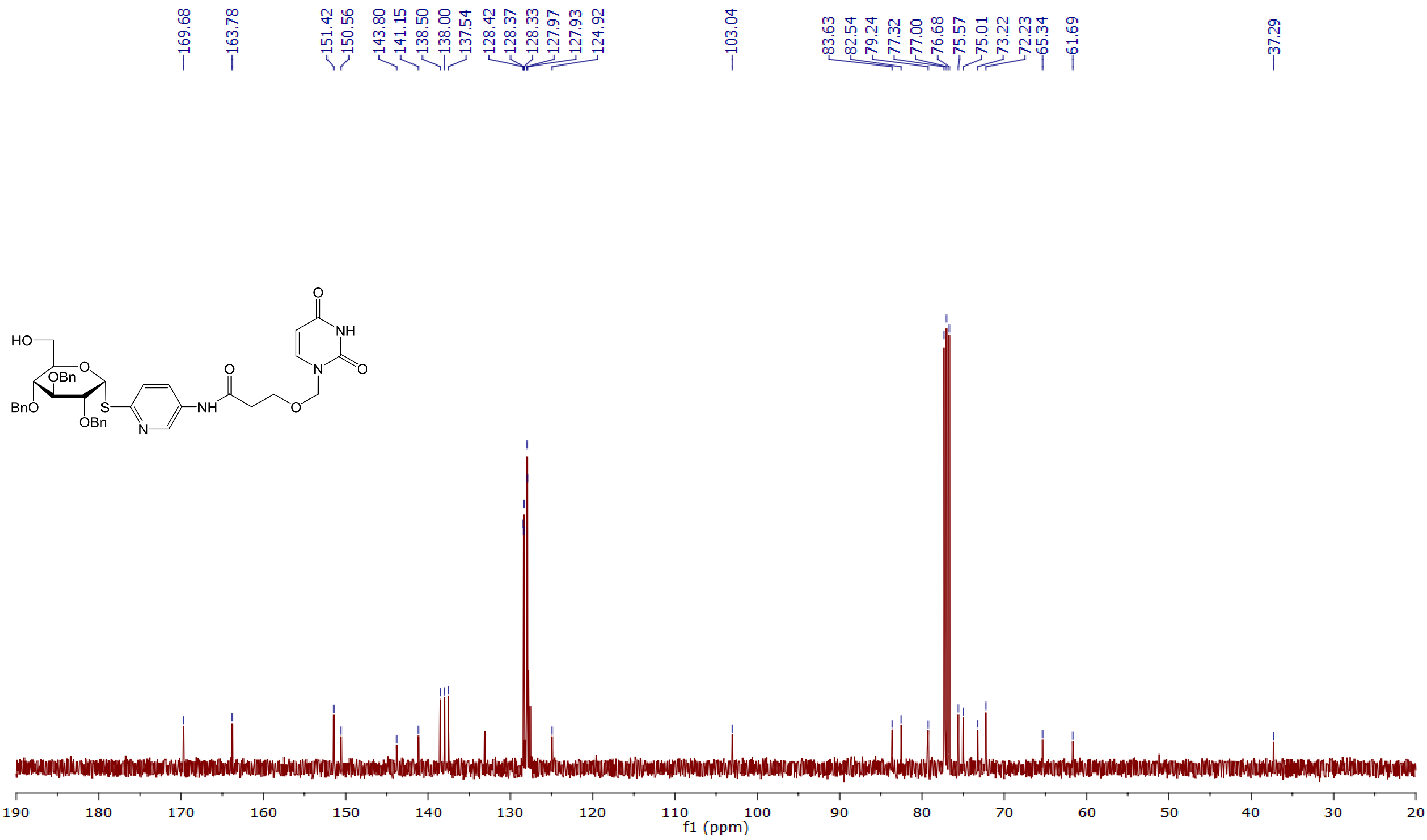

Fig. S50: ^{13}C NMR spectrum of glycoconjugate **47**

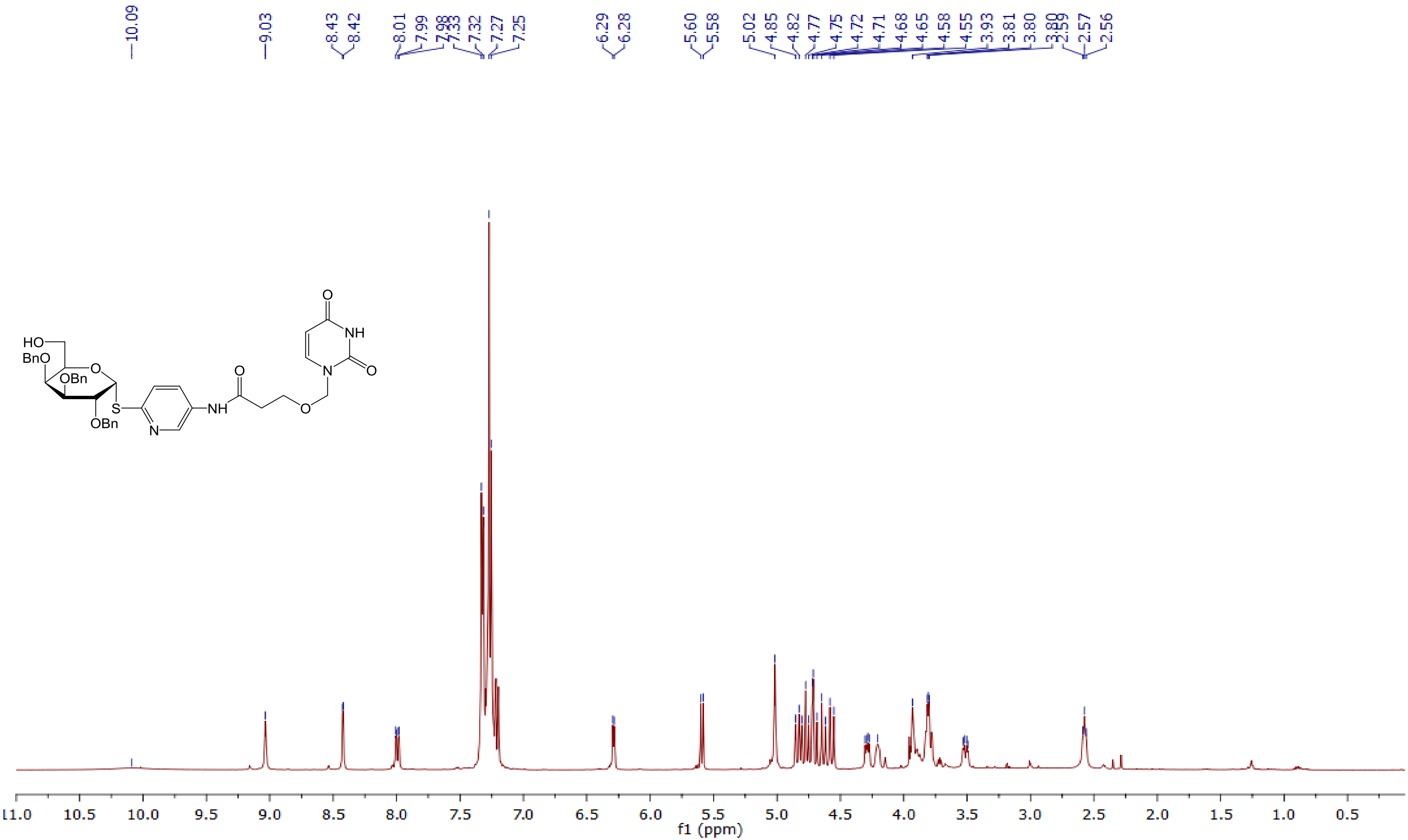

Fig. S51: ^{1}H NMR spectrum of glycoconjugate **48**

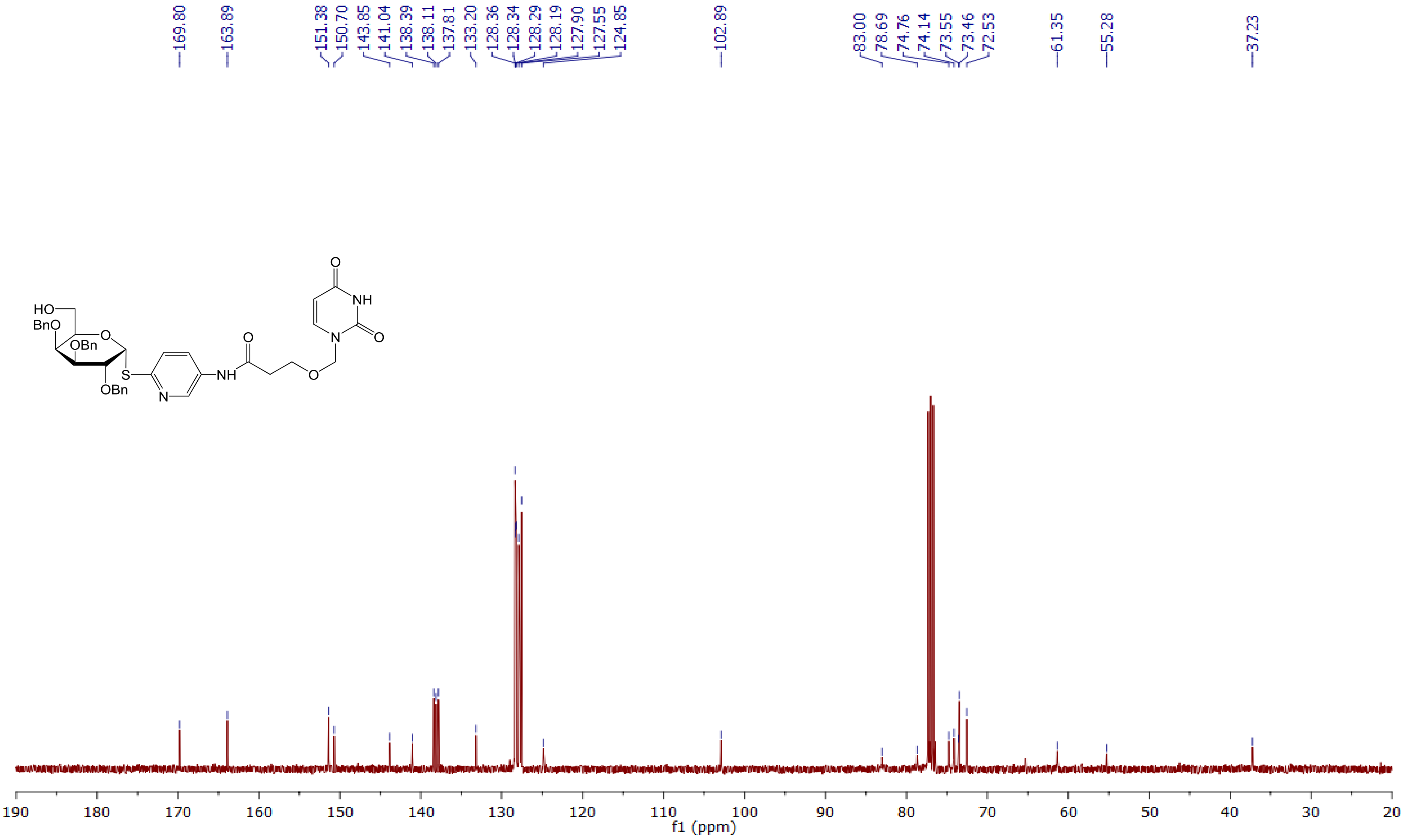

Fig. S52: ^{13}C NMR spectrum of glycoconjugate **48**

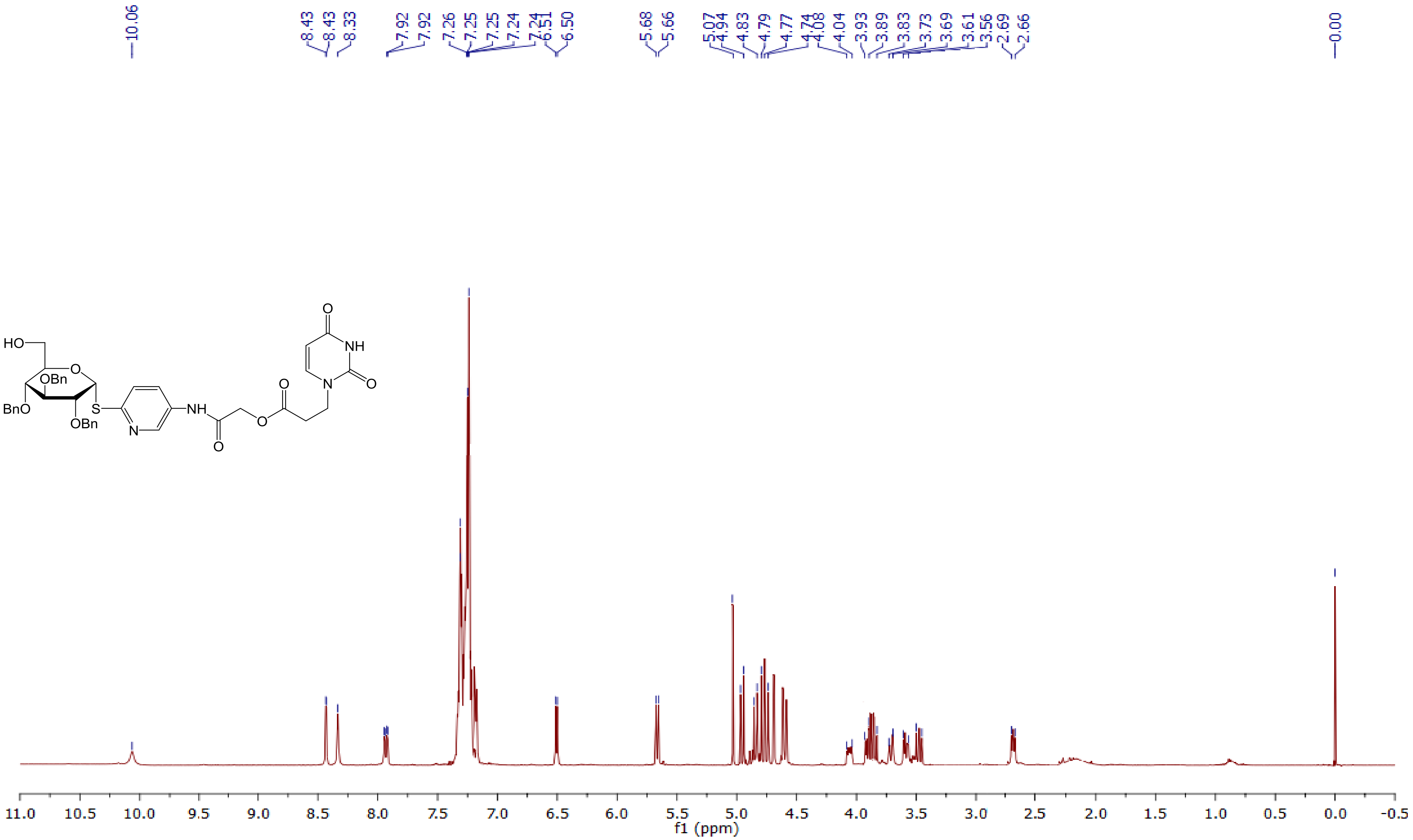

Fig. S53: ^{1}H NMR spectrum of glycoconjugate **49**

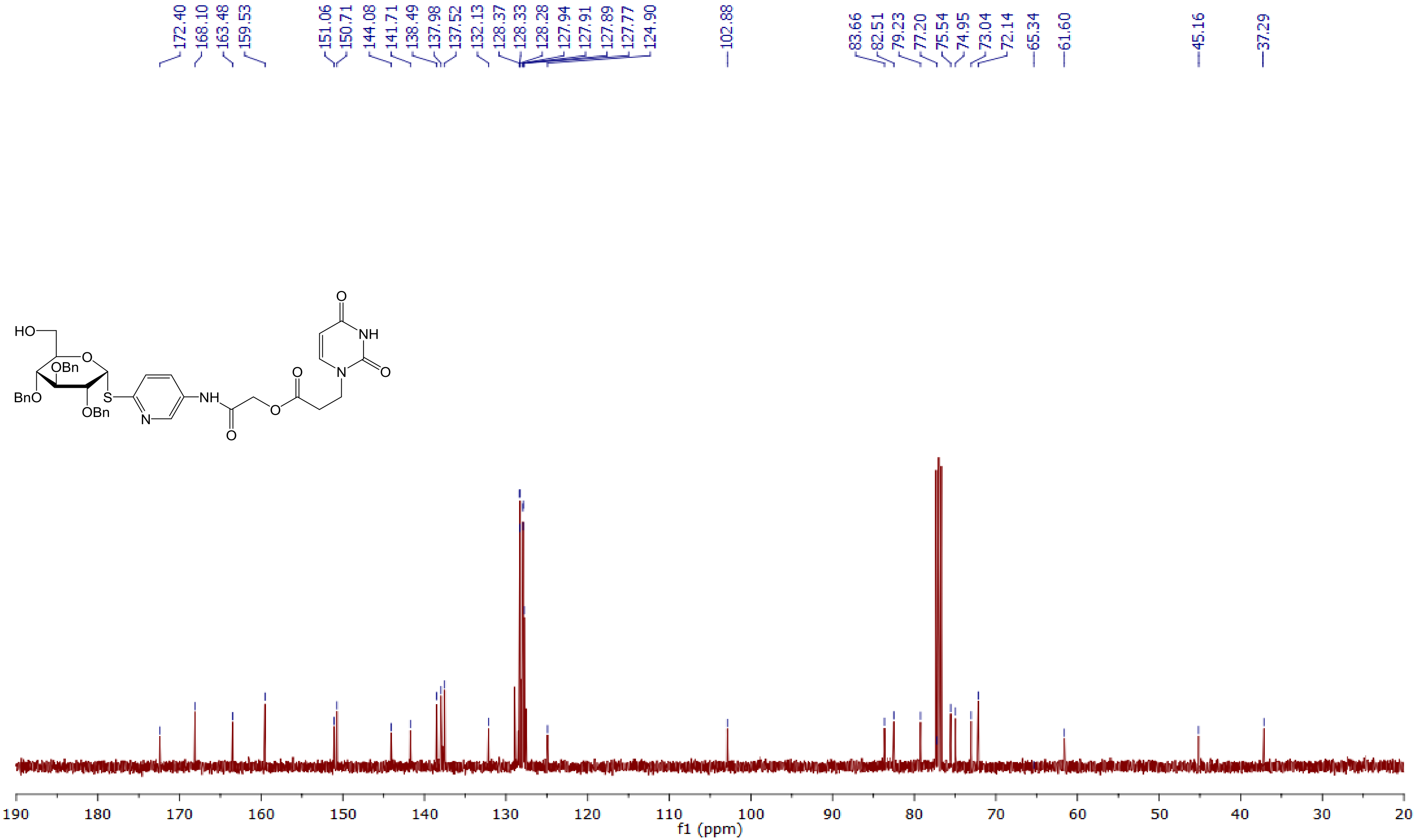

Fig. S54: ^{13}C NMR spectrum of glycoconjugate **49**

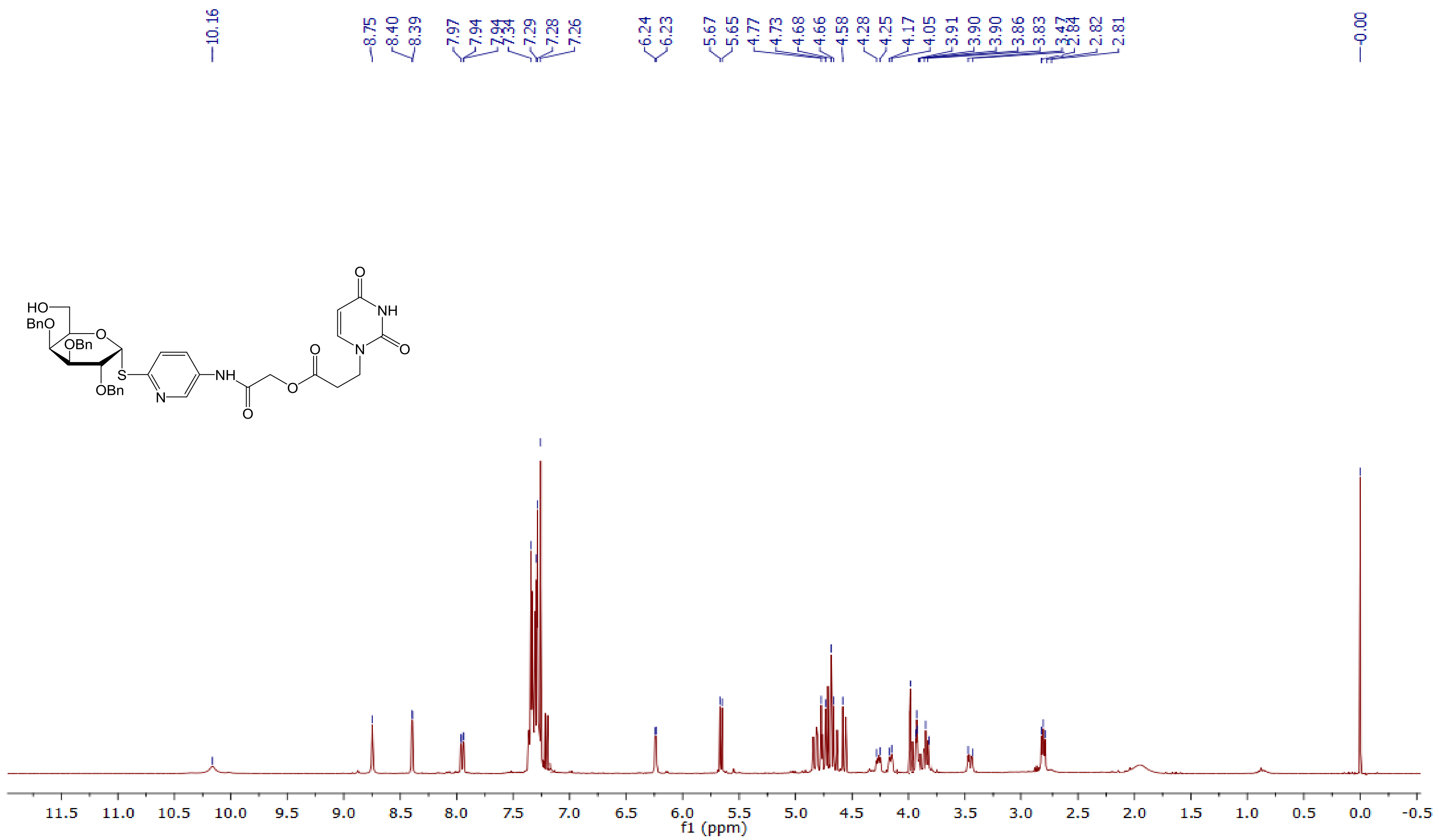

Fig. S55: ^{1}H NMR spectrum of glycoconjugate **50**

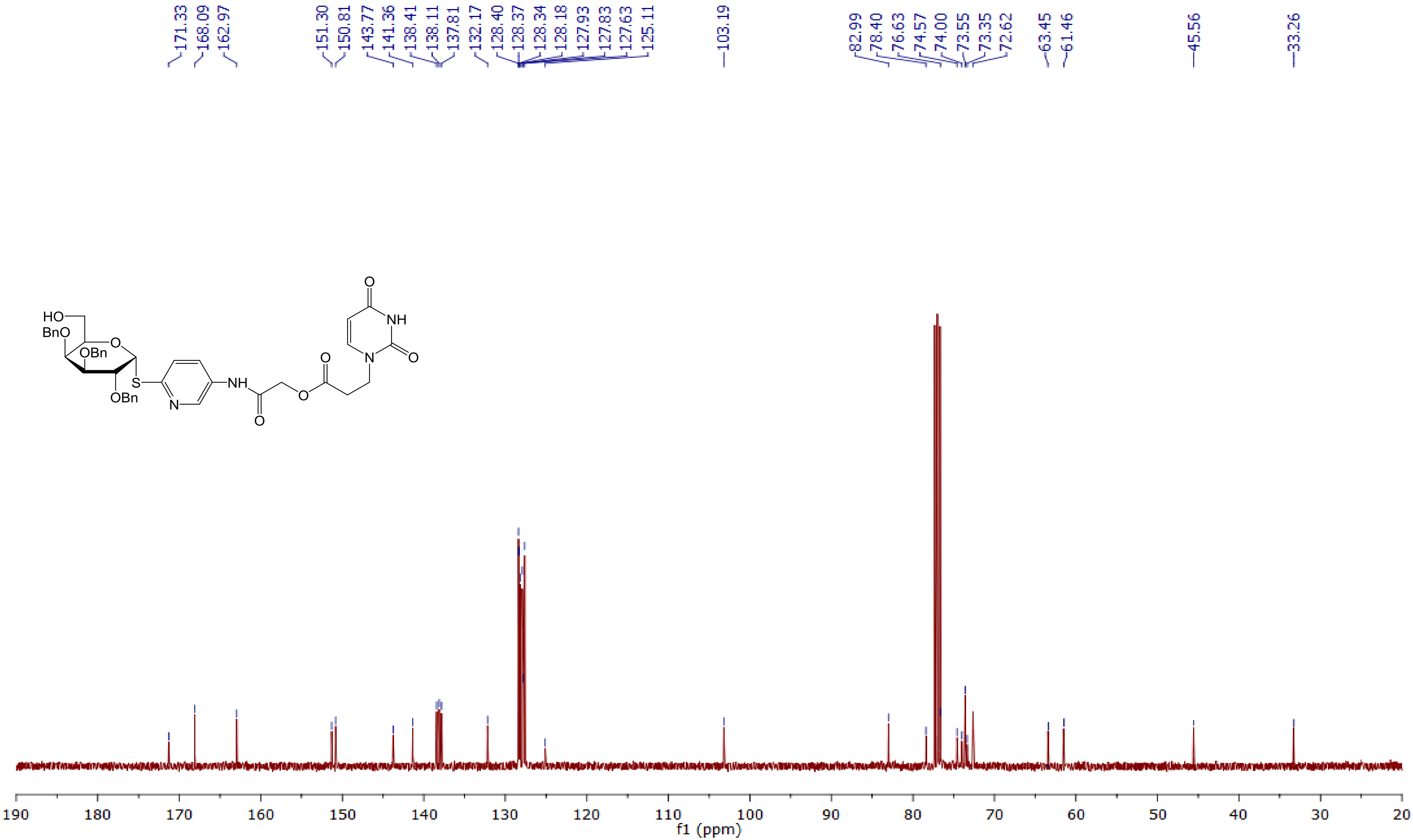

Fig. S56: ^{13}C NMR spectrum of glycoconjugate **50**

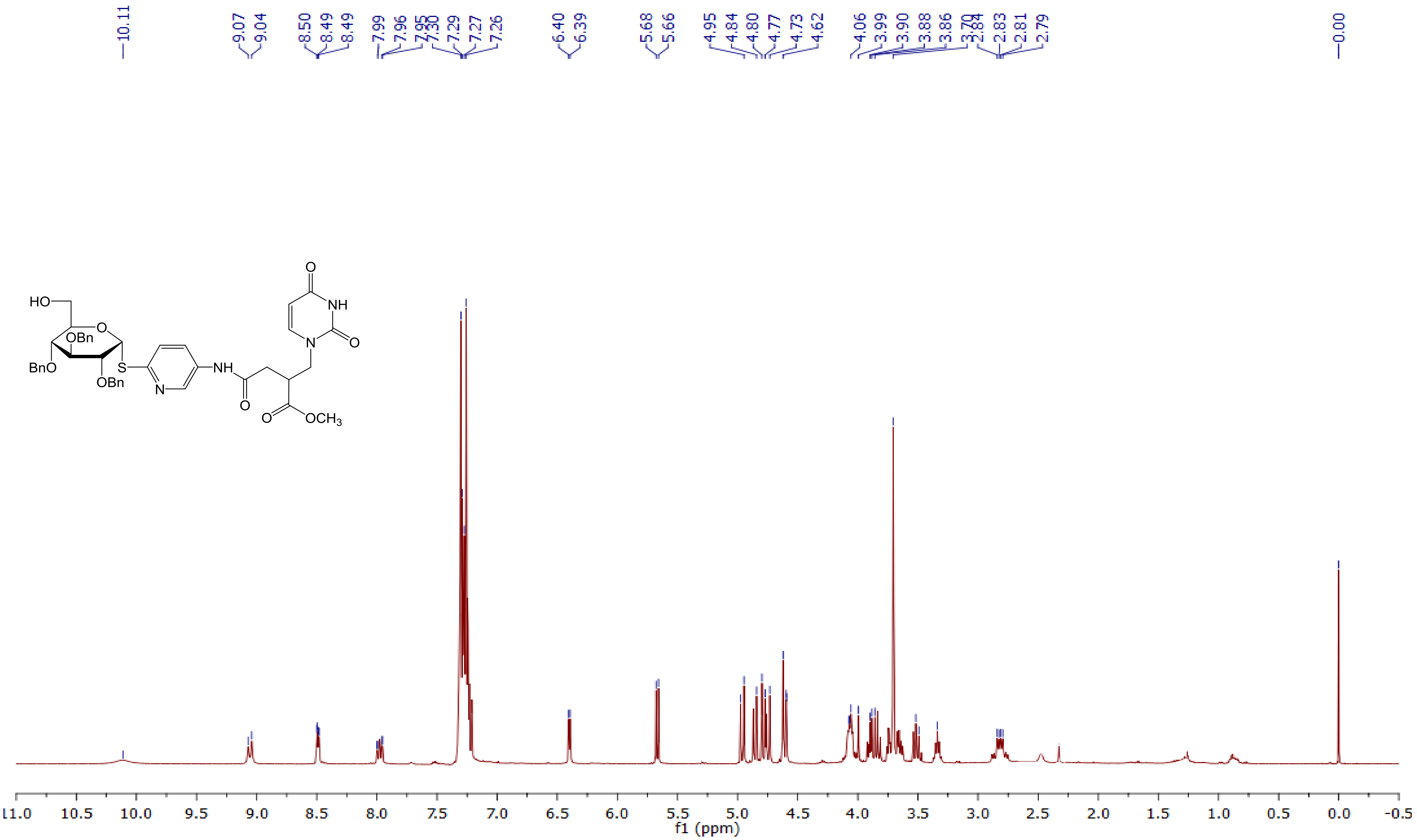

Fig. S57: ^{1}H NMR spectrum of glycoconjugate **51**

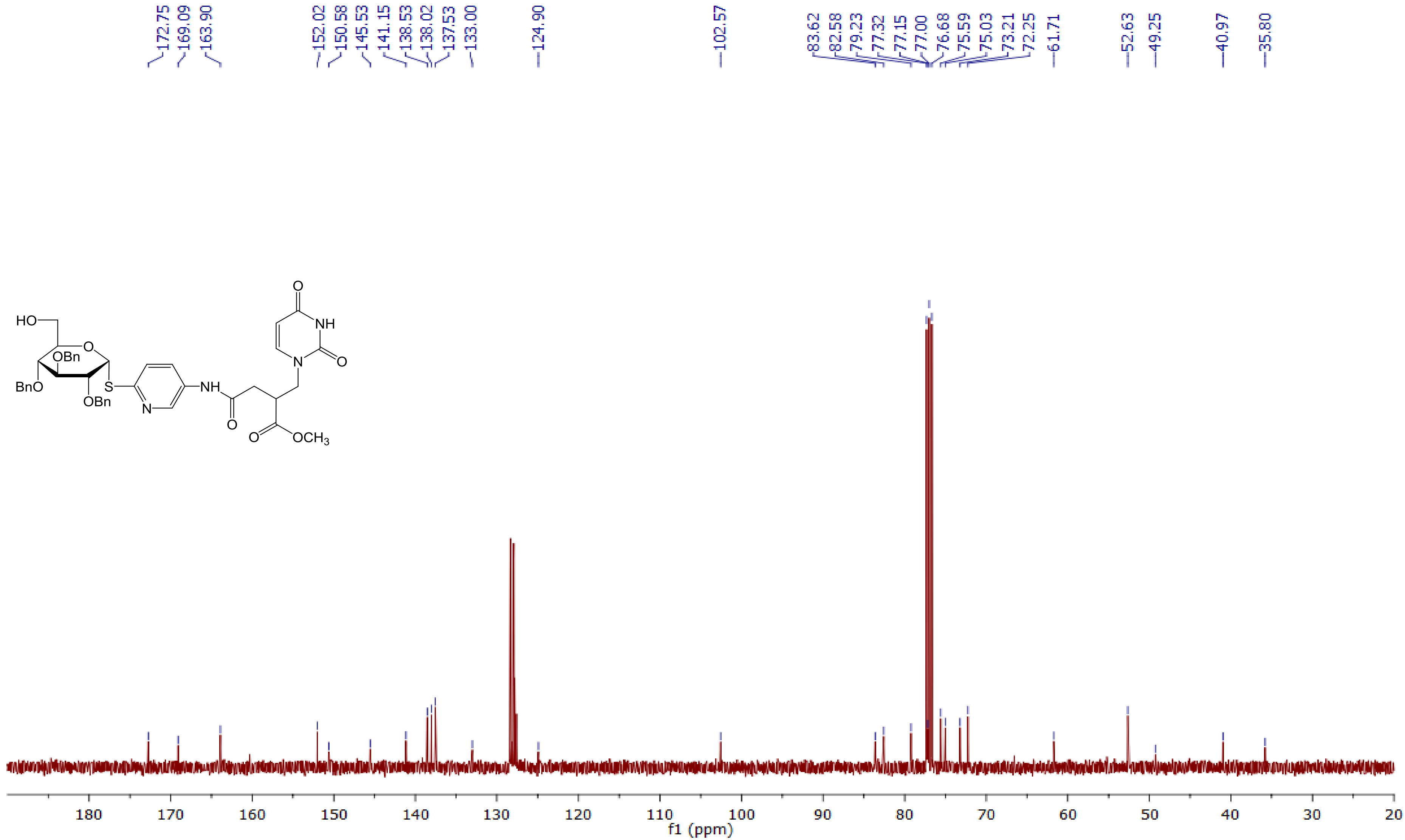

Fig. S58: ^{13}C NMR spectrum of glycoconjugate **51**

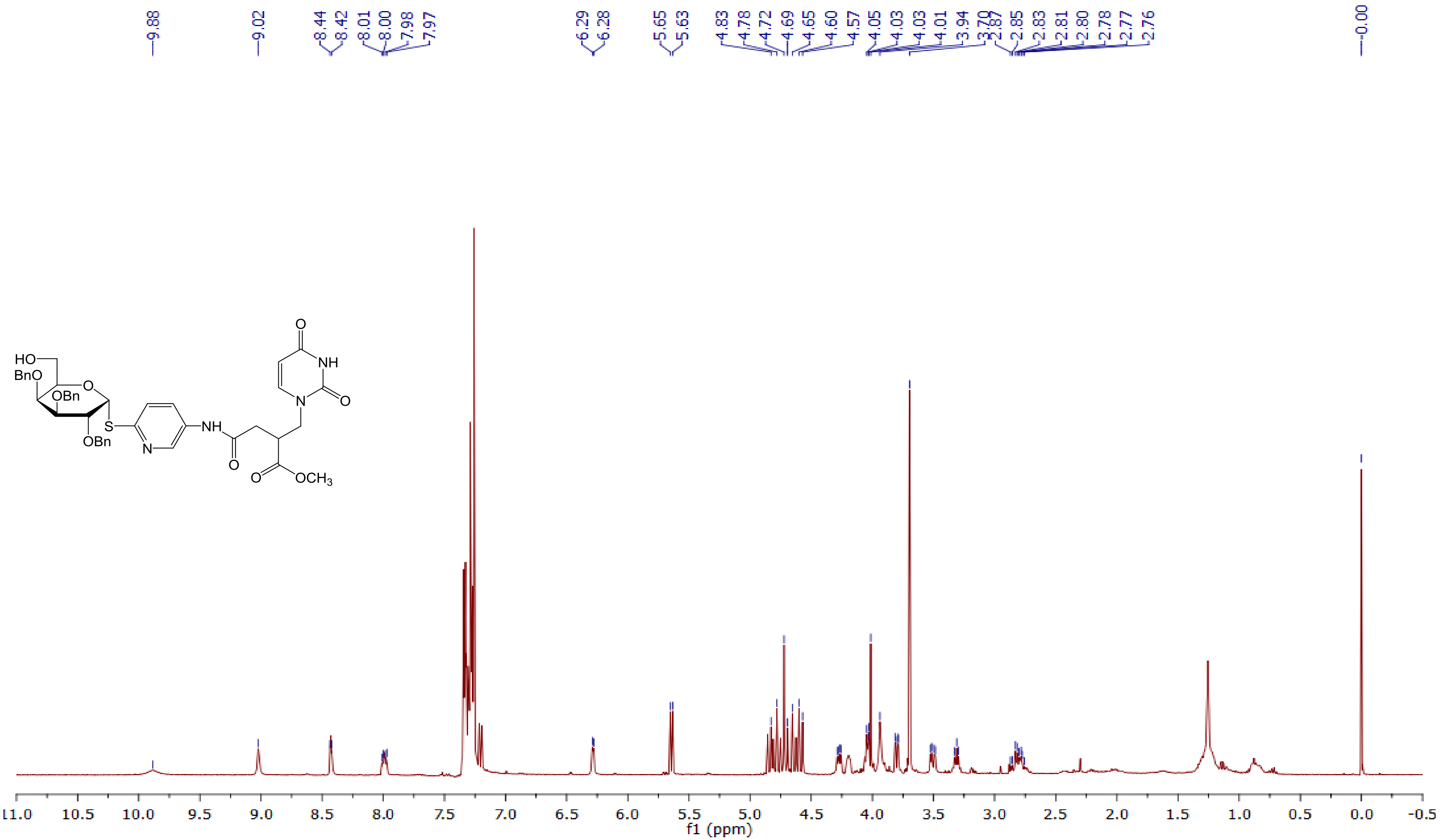

Fig. S59: ^{1}H NMR spectrum of glycoconjugate **52**

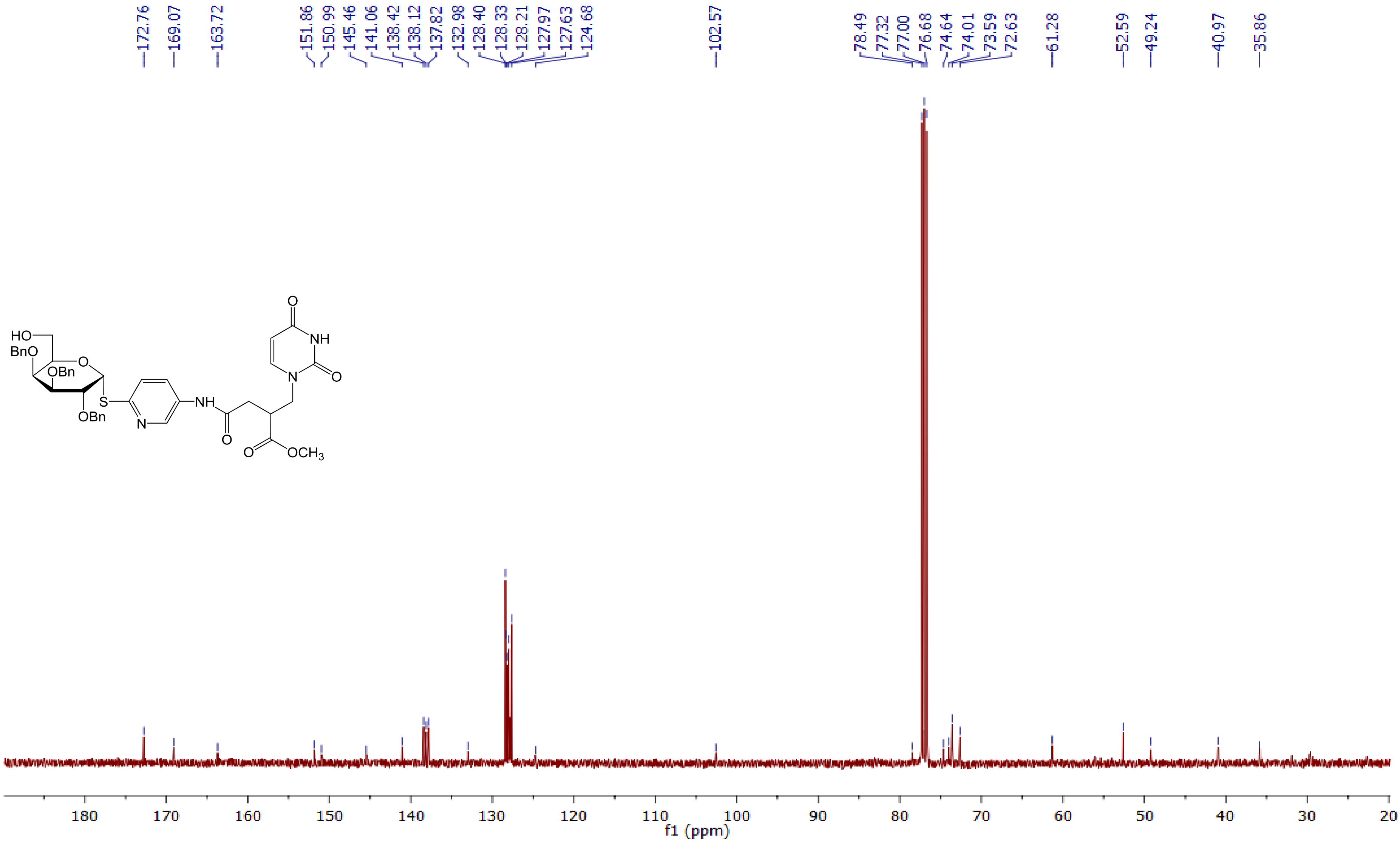

Fig. S60: ^{13}C NMR spectrum of glycoconjugate **52**

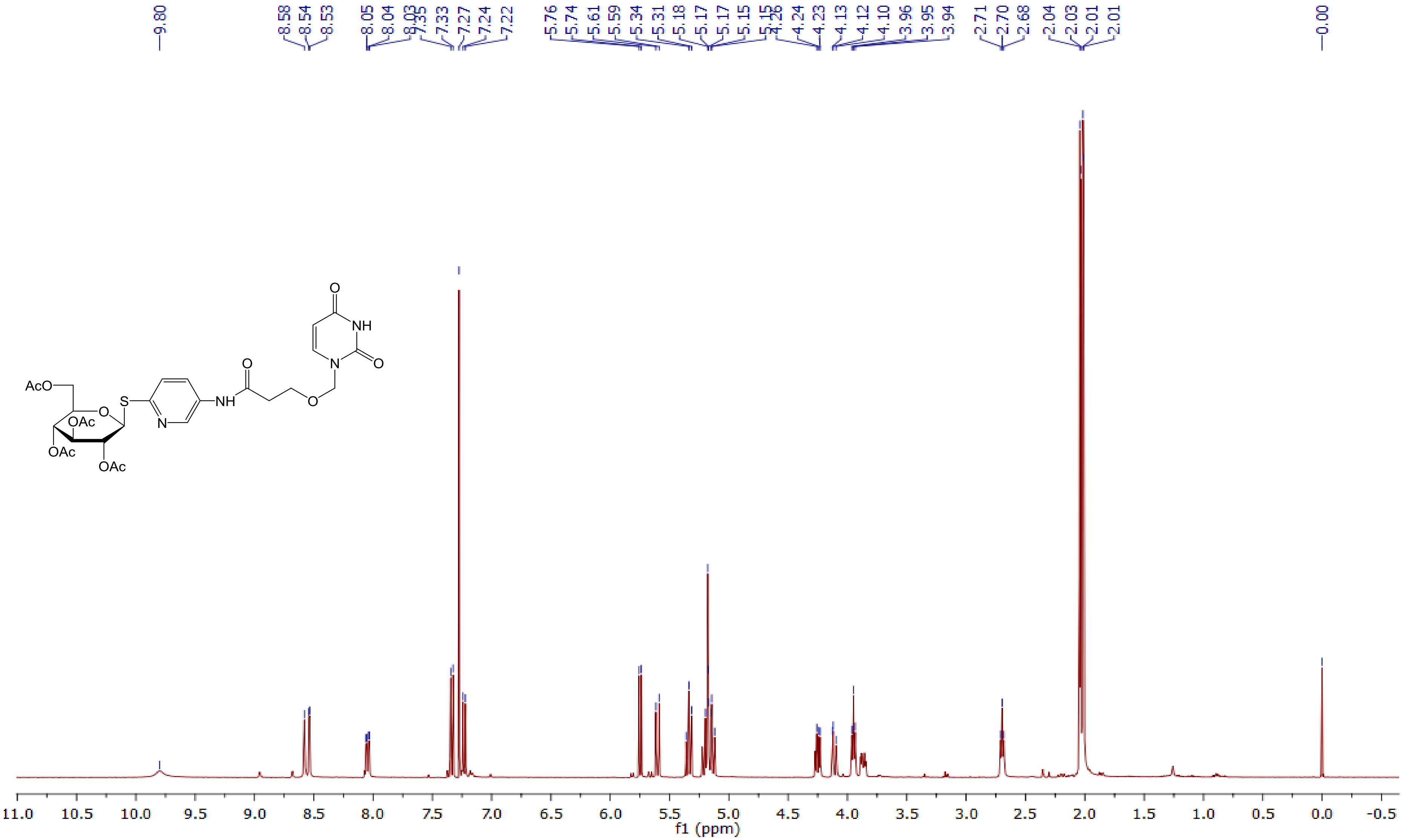

Fig. S61: ^{1}H NMR spectrum of glycoconjugate **53**

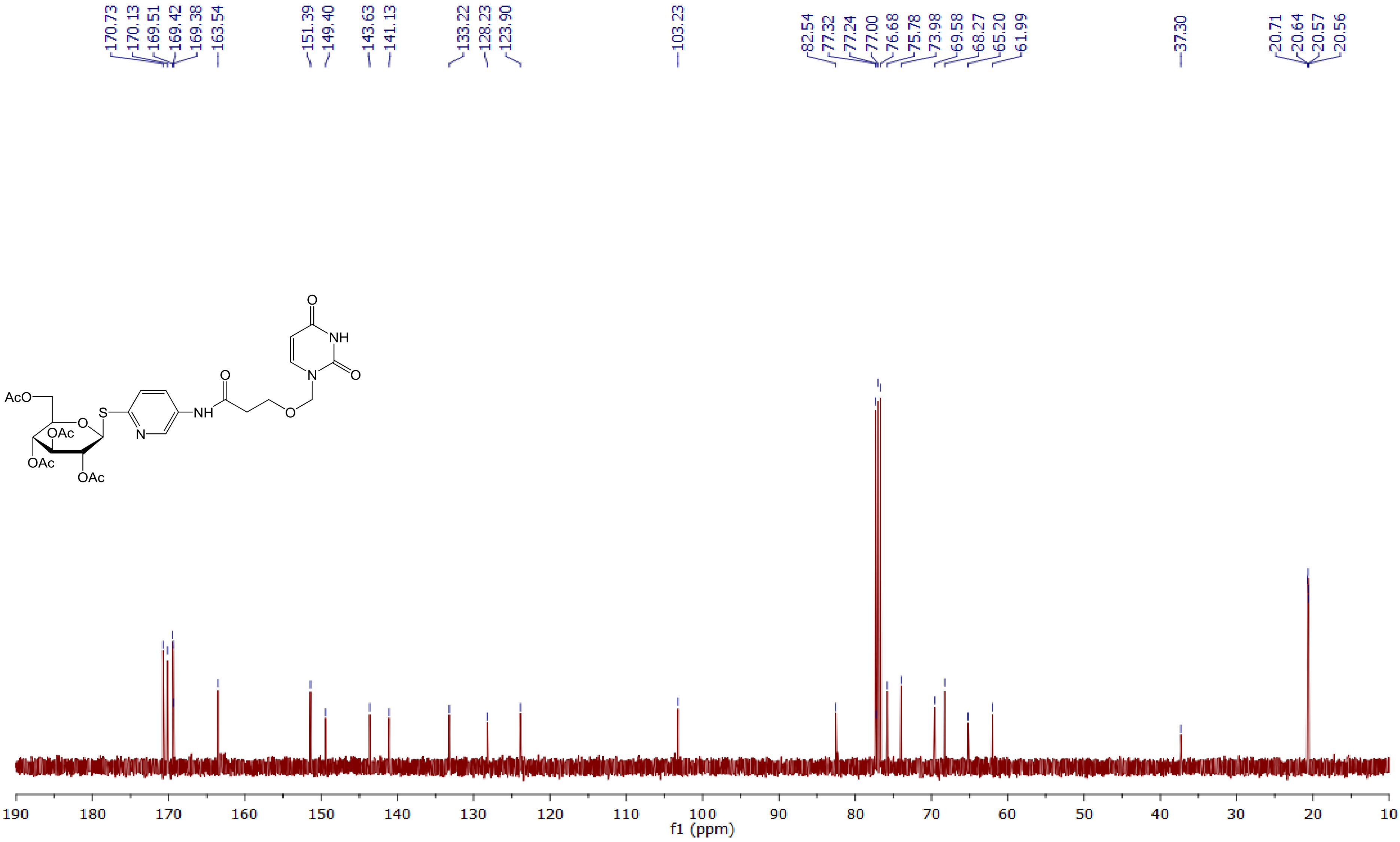

Fig. S62: ^{13}C NMR spectrum of glycoconjugate **53**

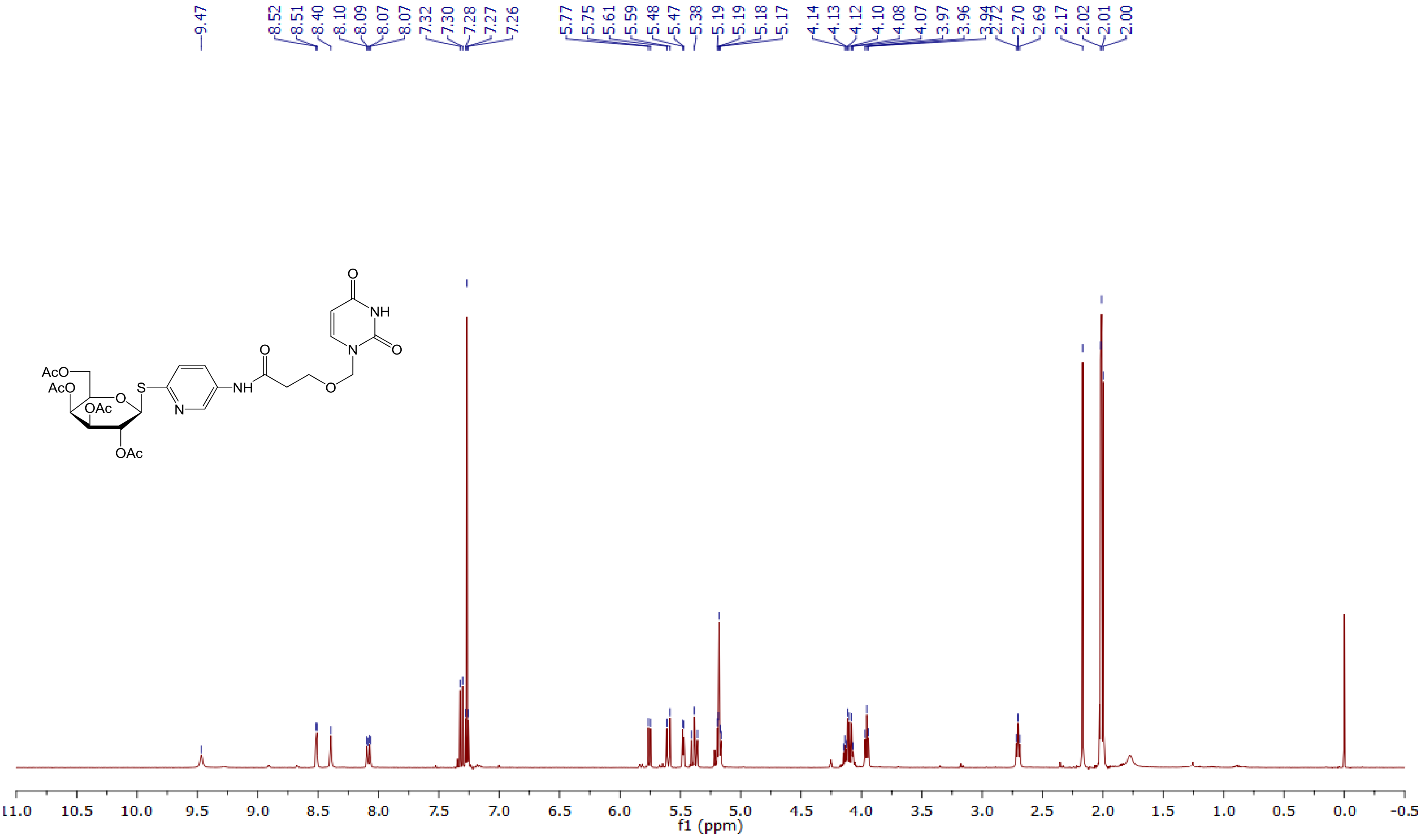

Fig. S63: ^{1}H NMR spectrum of glycoconjugate **54**

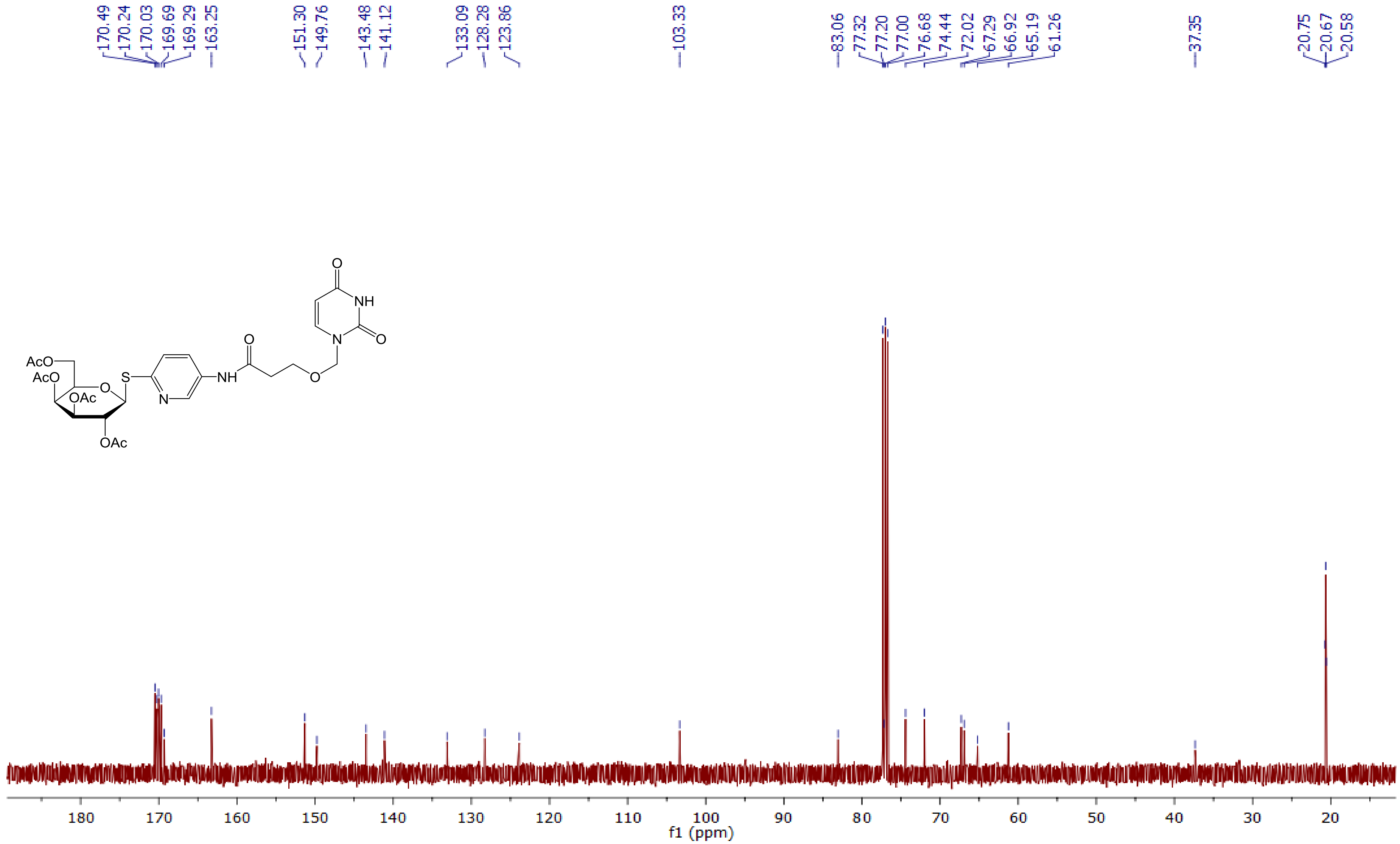

Fig. S64: ^{13}C NMR spectrum of glycoconjugate **54**

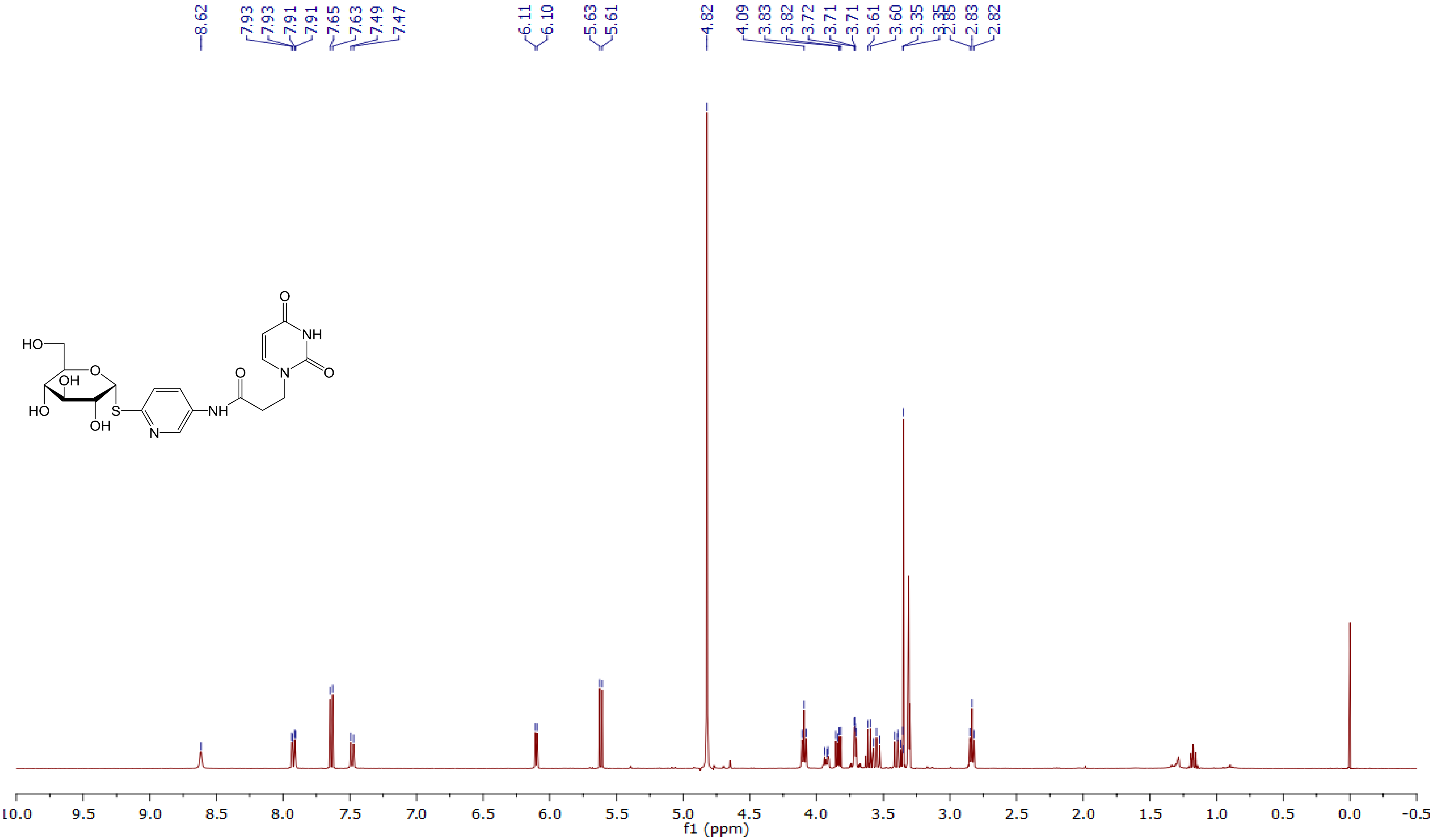

Fig. S65: ^{1}H NMR spectrum of glycoconjugate **55**

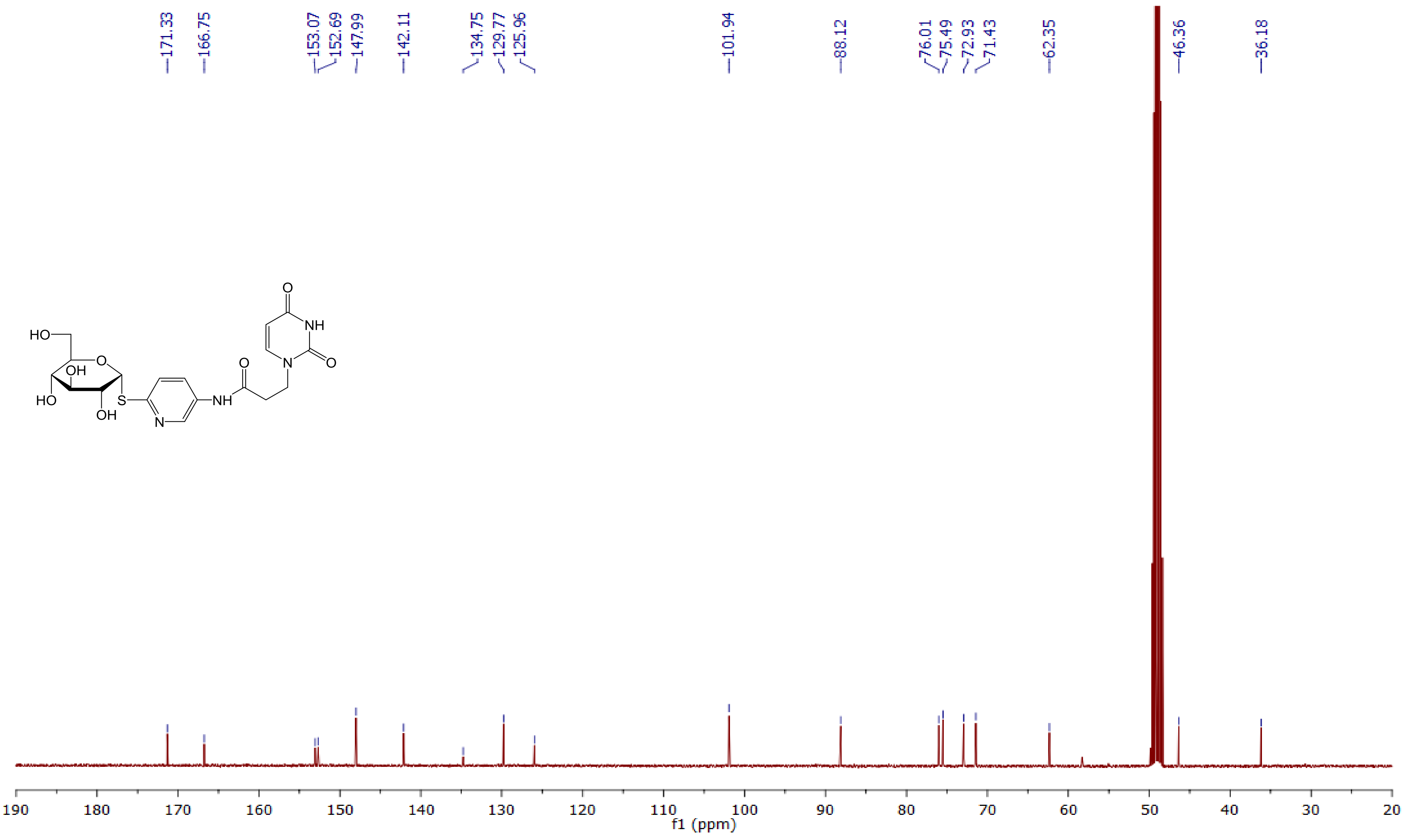

Fig. S66: ^{13}C NMR spectrum of glycoconjugate **55**

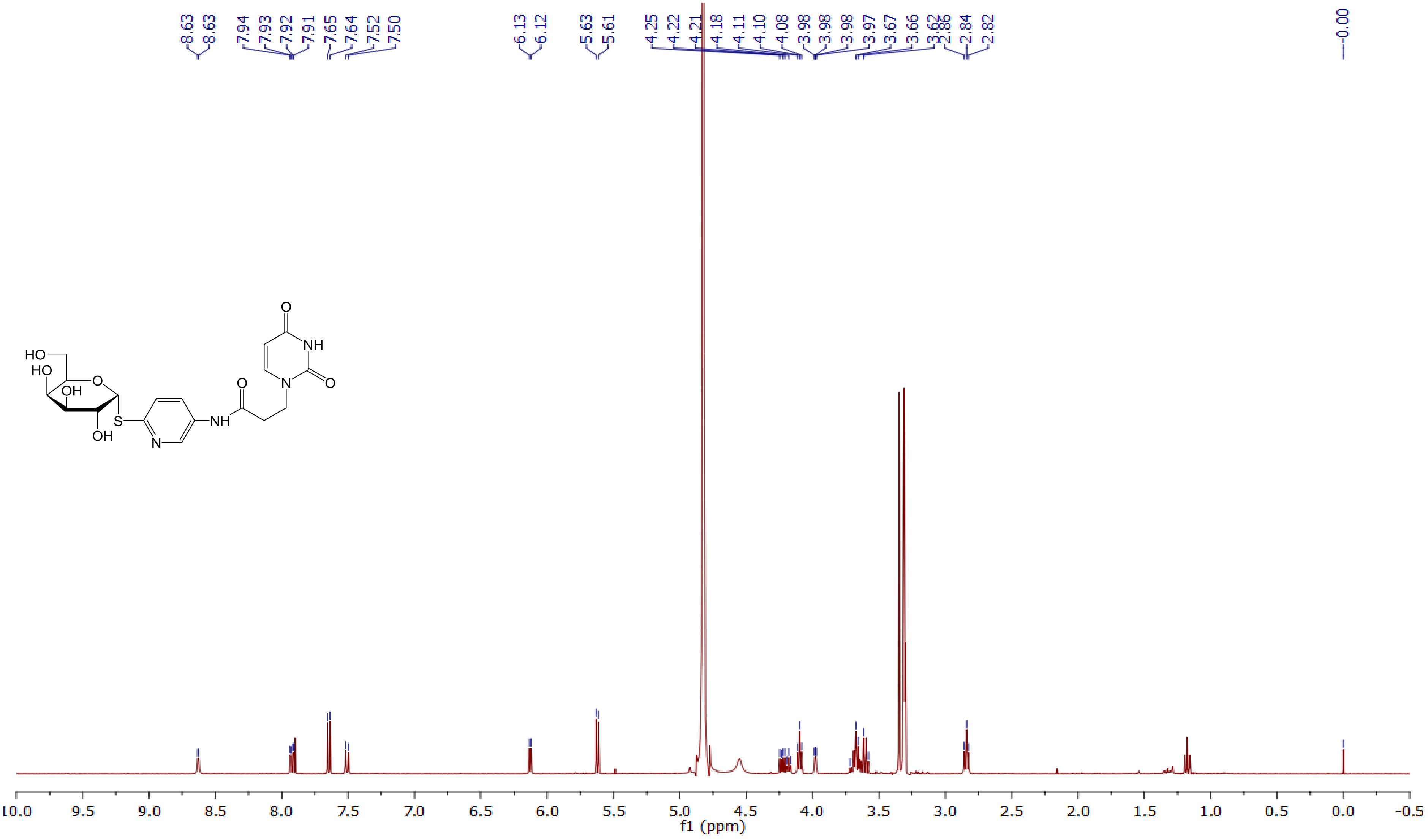

Fig. S67: ^{1}H NMR spectrum of glycoconjugate **56**

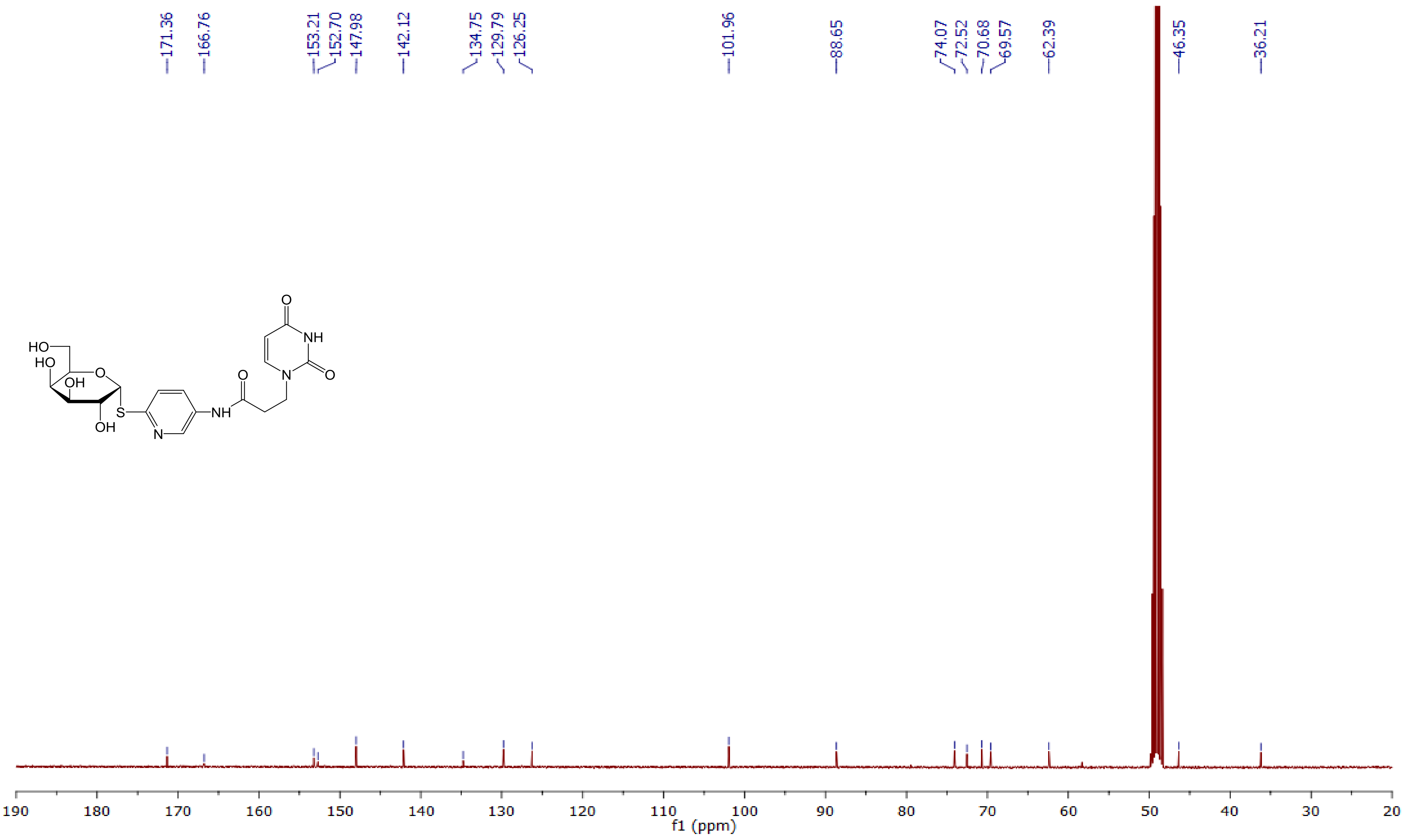

Fig. S68: ^{13}C NMR spectrum of glycoconjugate **56**

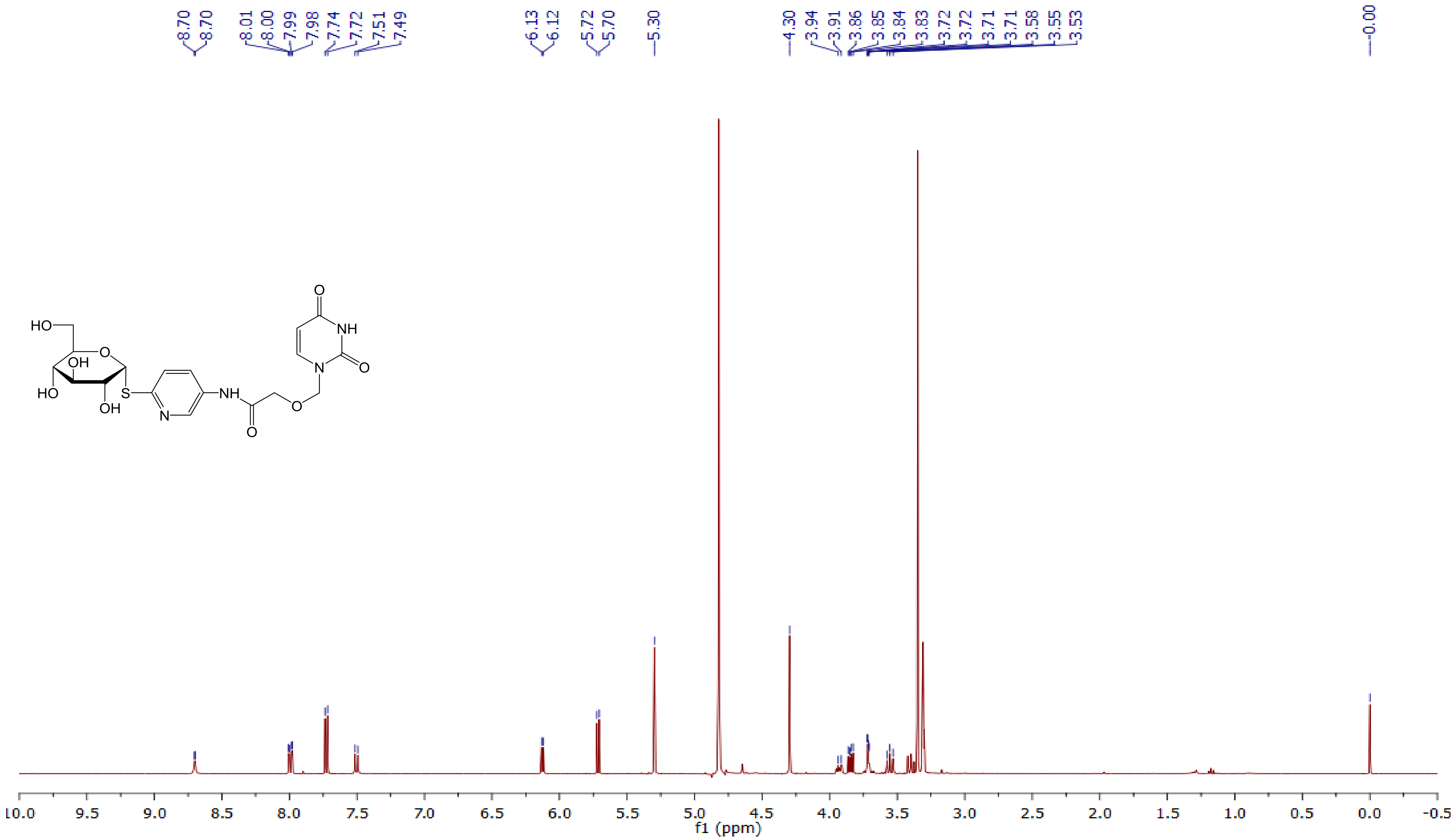

Fig. S69: ^{1}H NMR spectrum of glycoconjugate **57**

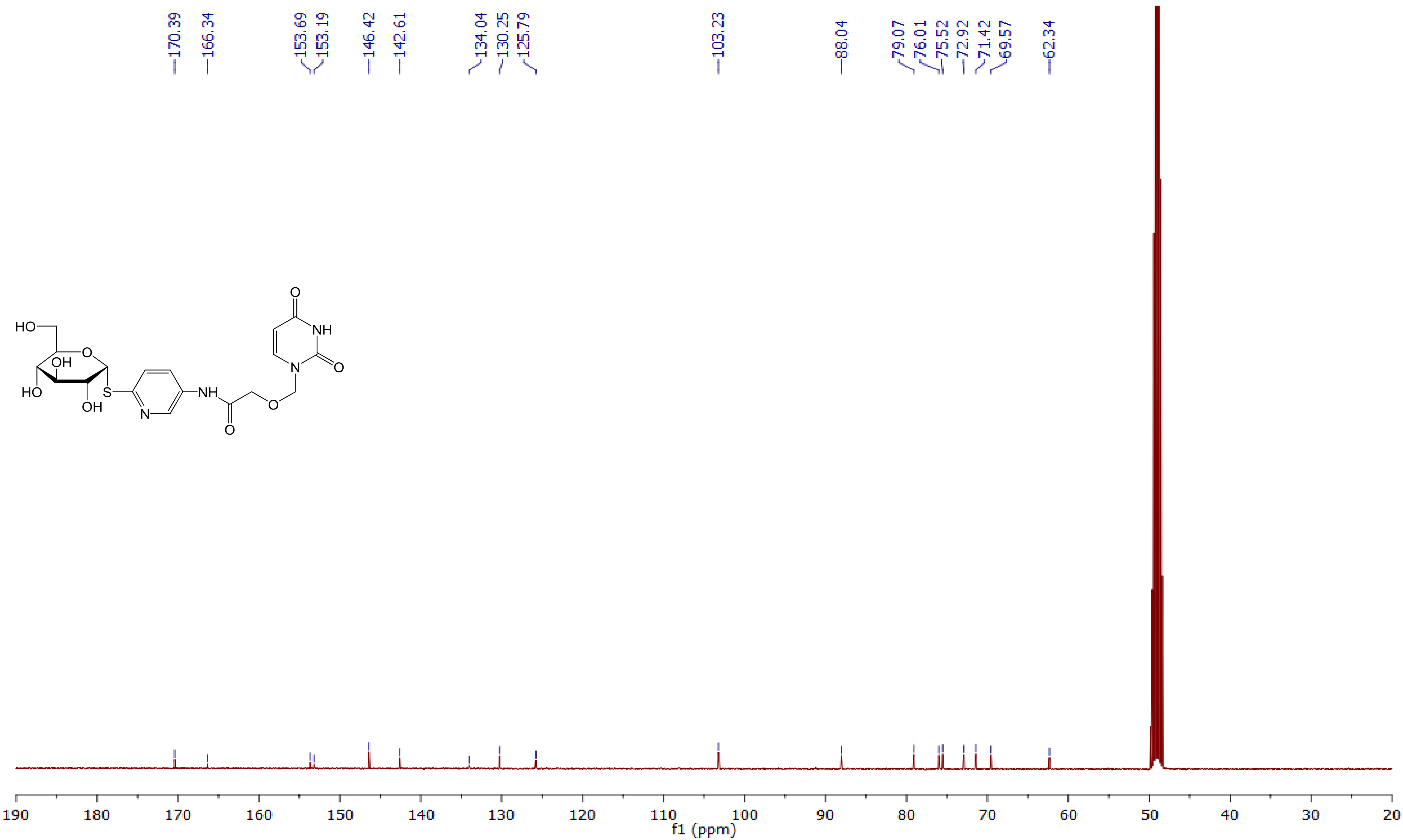

Fig. S70: ^{13}C NMR spectrum of glycoconjugate **57**

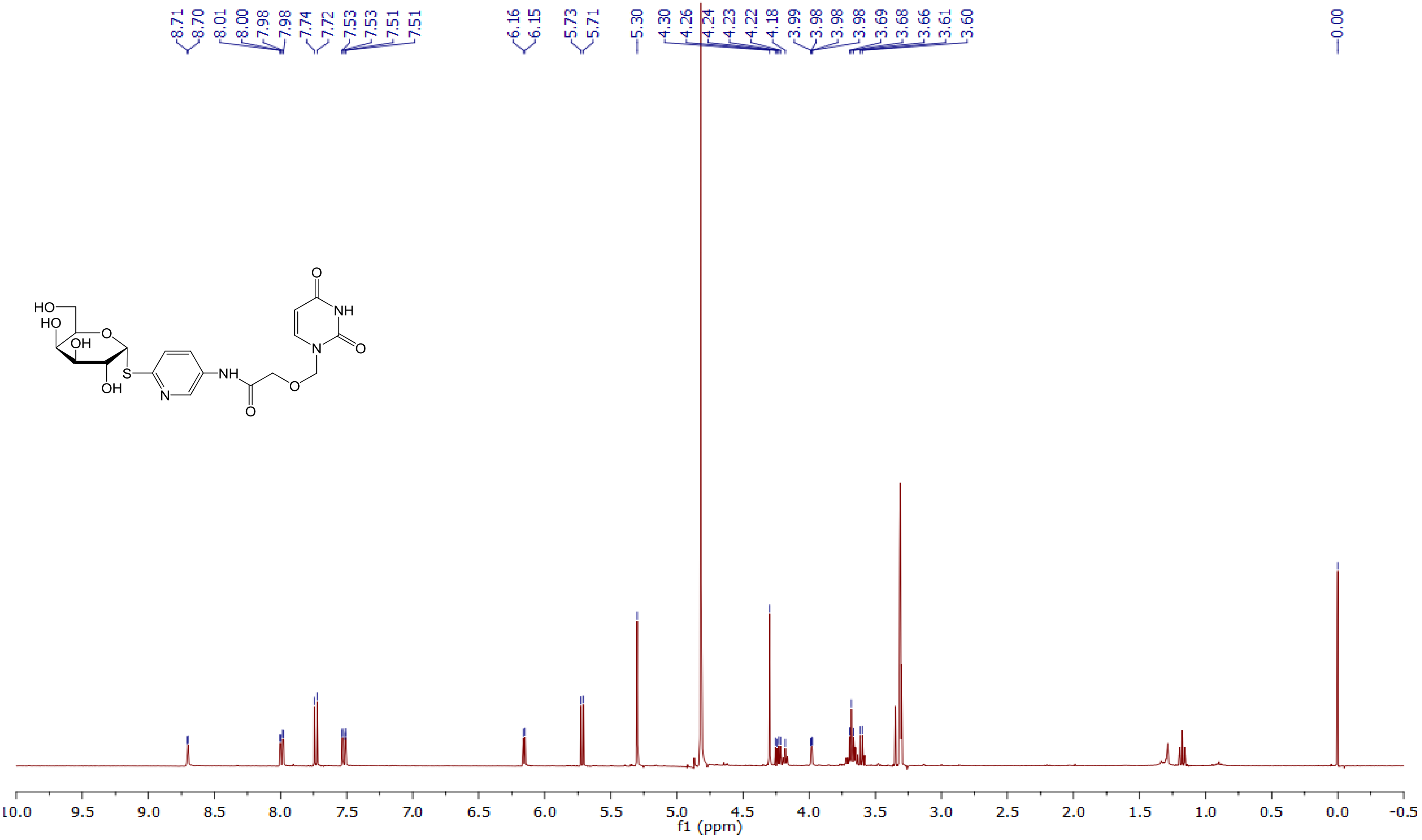

Fig. S71: ^{1}H NMR spectrum of glycoconjugate **58**

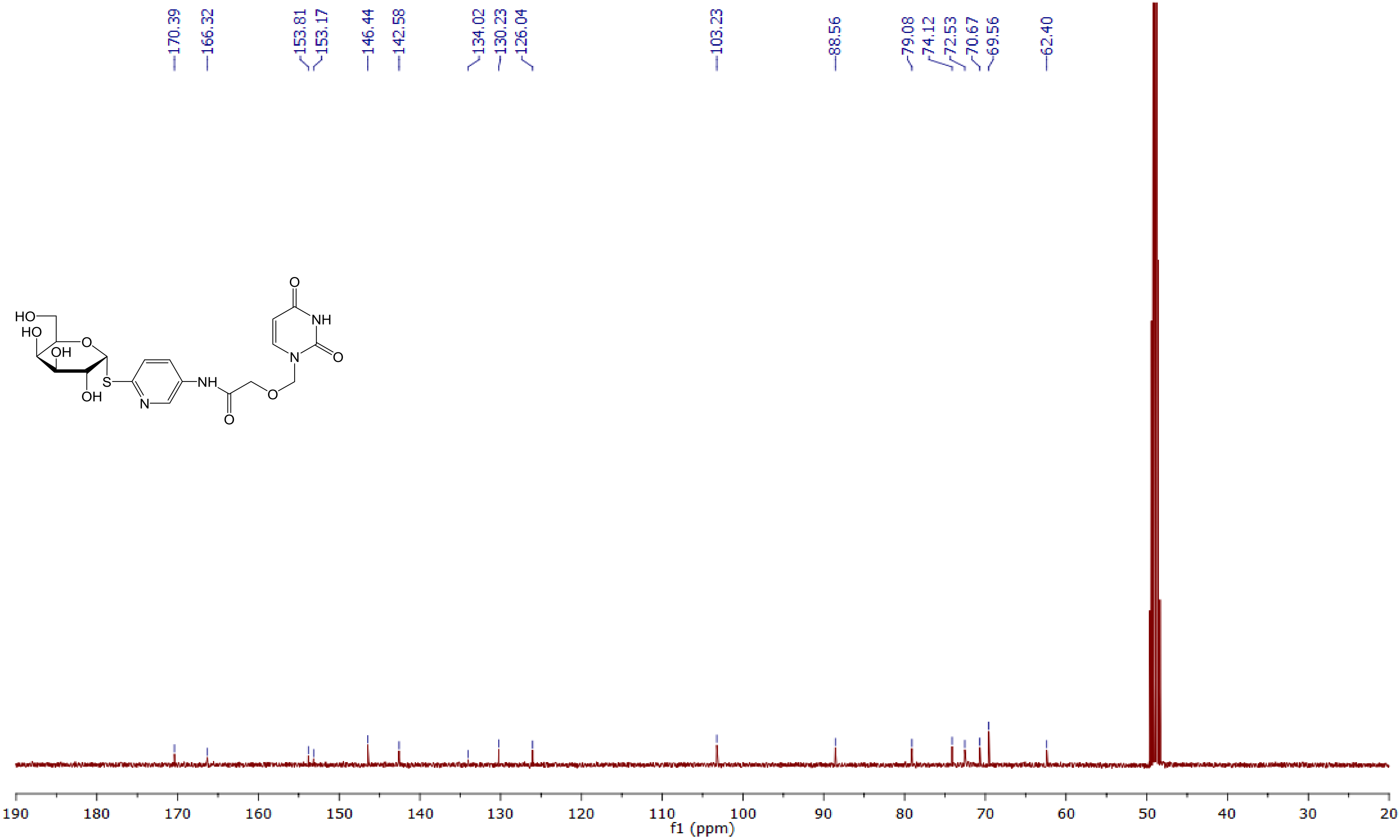

Fig. S72: ^{13}C NMR spectrum of glycoconjugate **58**

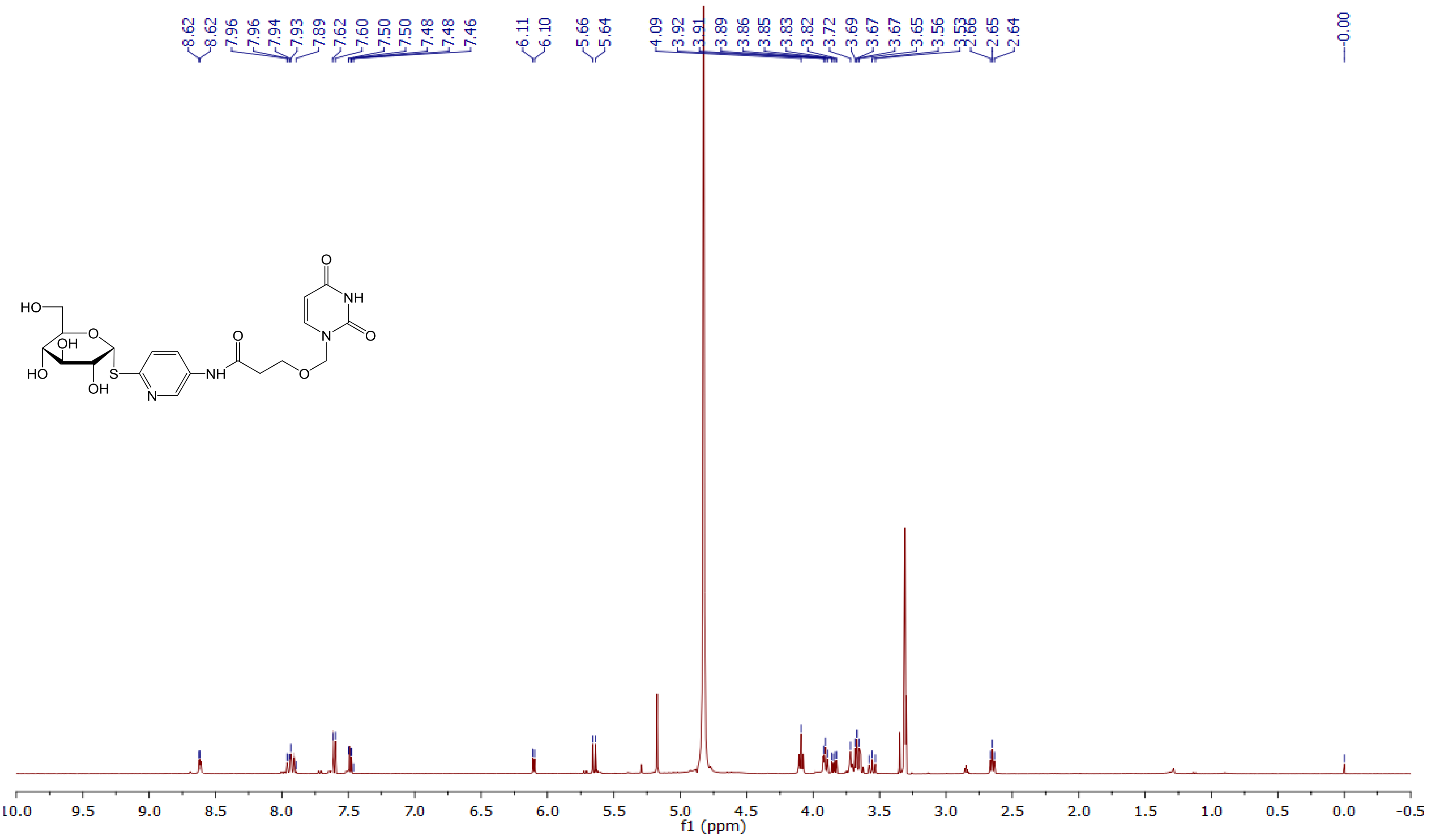

Fig. S73: ^{1}H NMR spectrum of glycoconjugate **59**

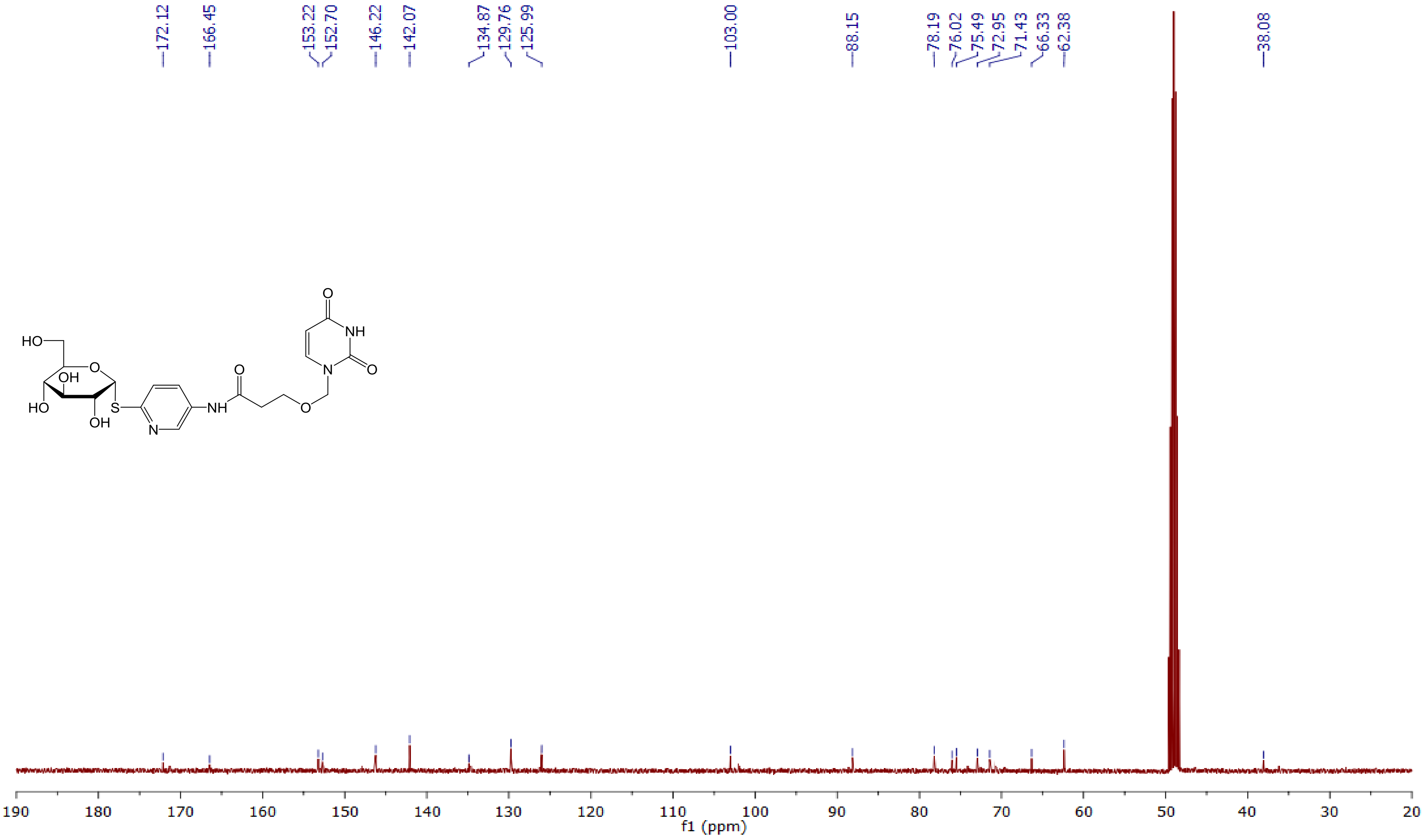

Fig. S74: ^{13}C NMR spectrum of glycoconjugate **59**

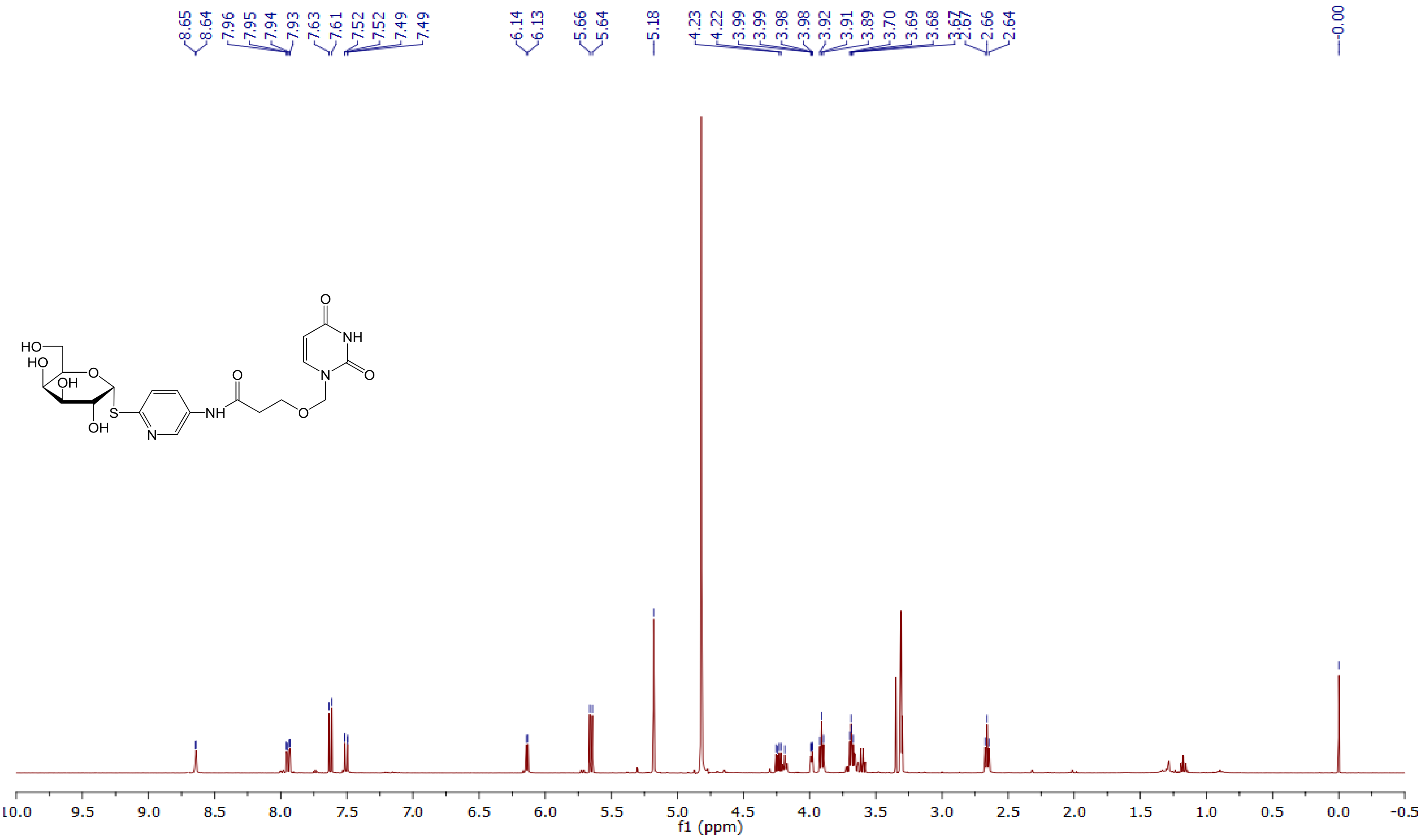

Fig. S75: ^{1}H NMR spectrum of glycoconjugate **60**

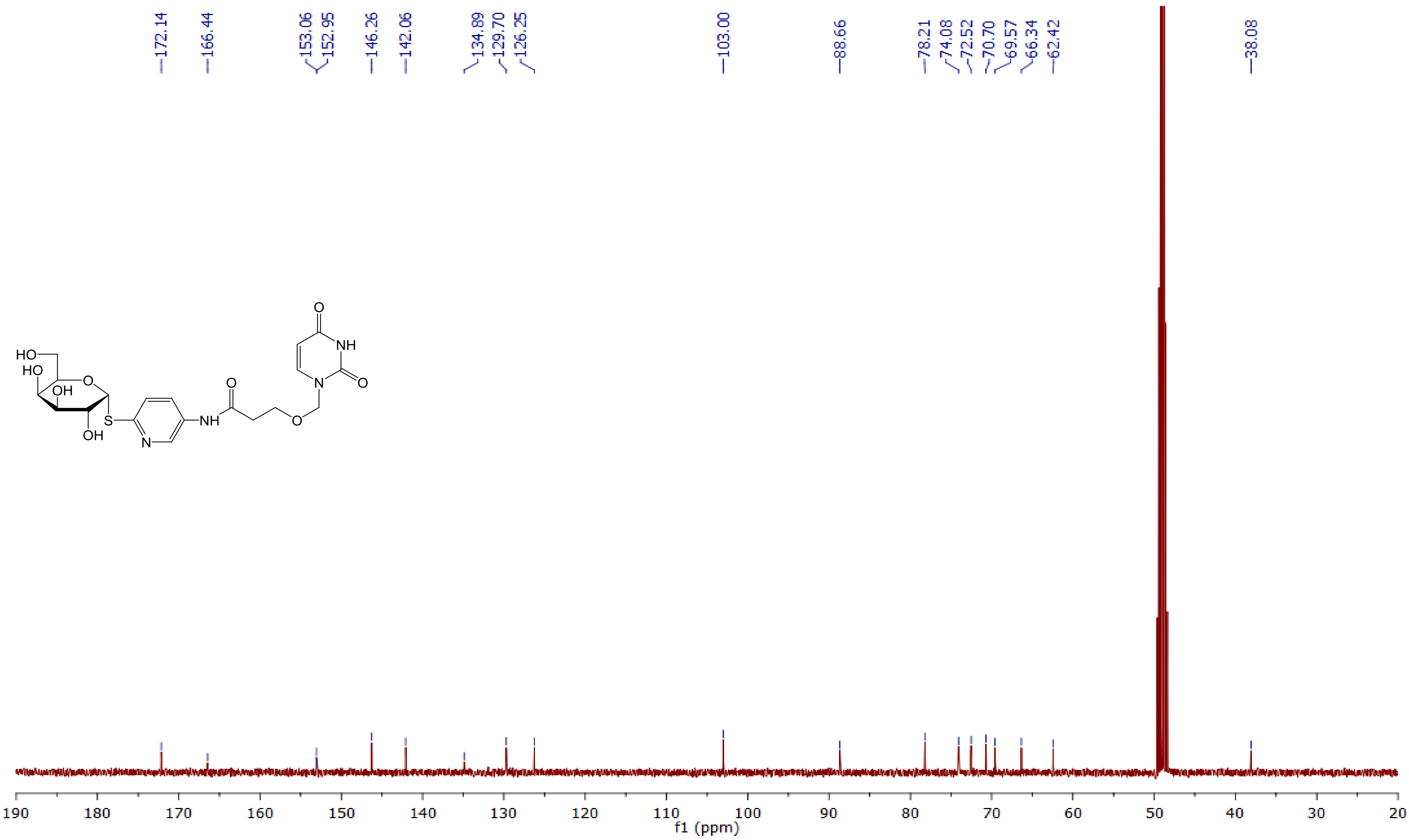

Fig. S76: ^{13}C NMR spectrum of glycoconjugate **60**

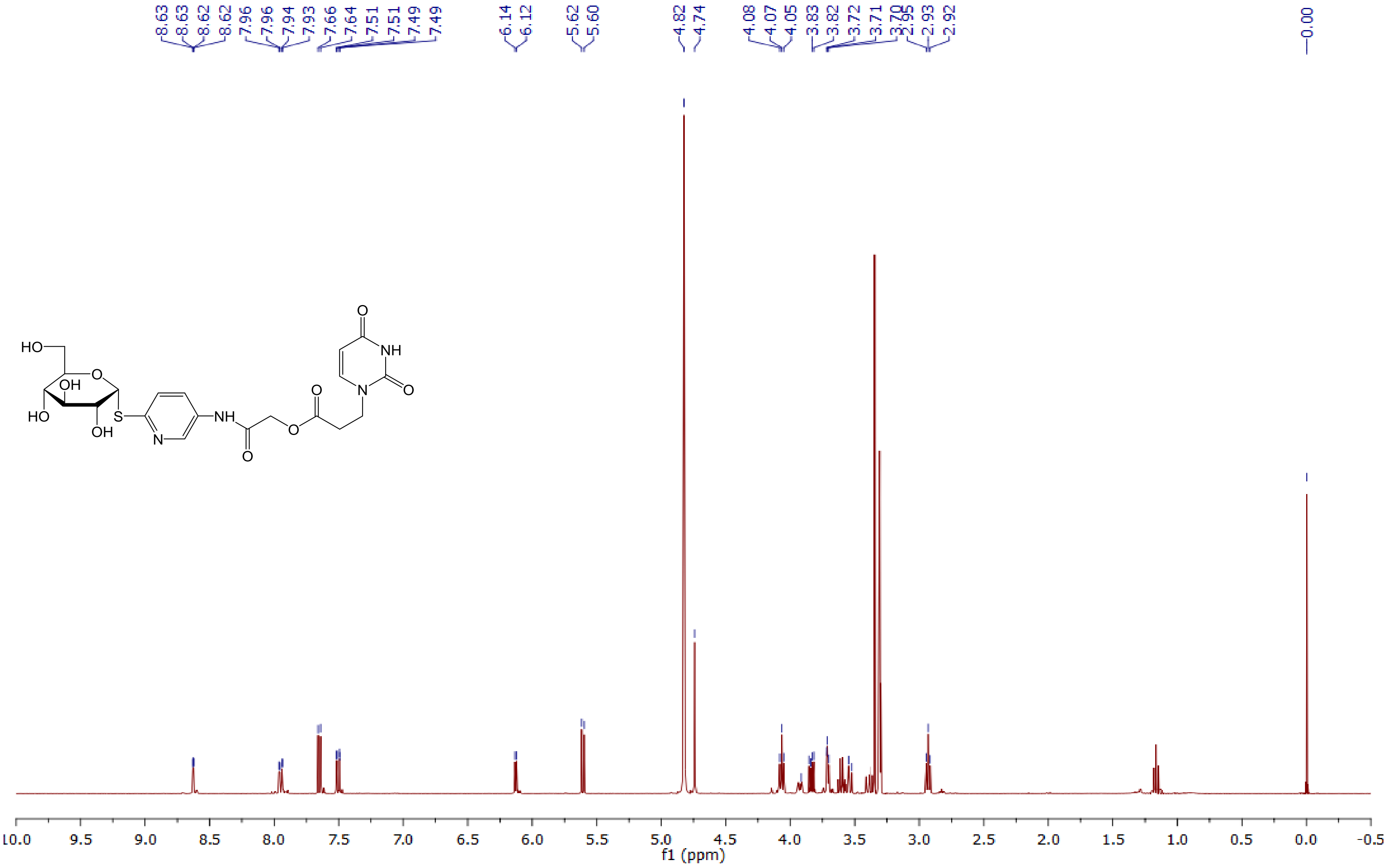

Fig. S77: ^{1}H NMR spectrum of glycoconjugate **61**

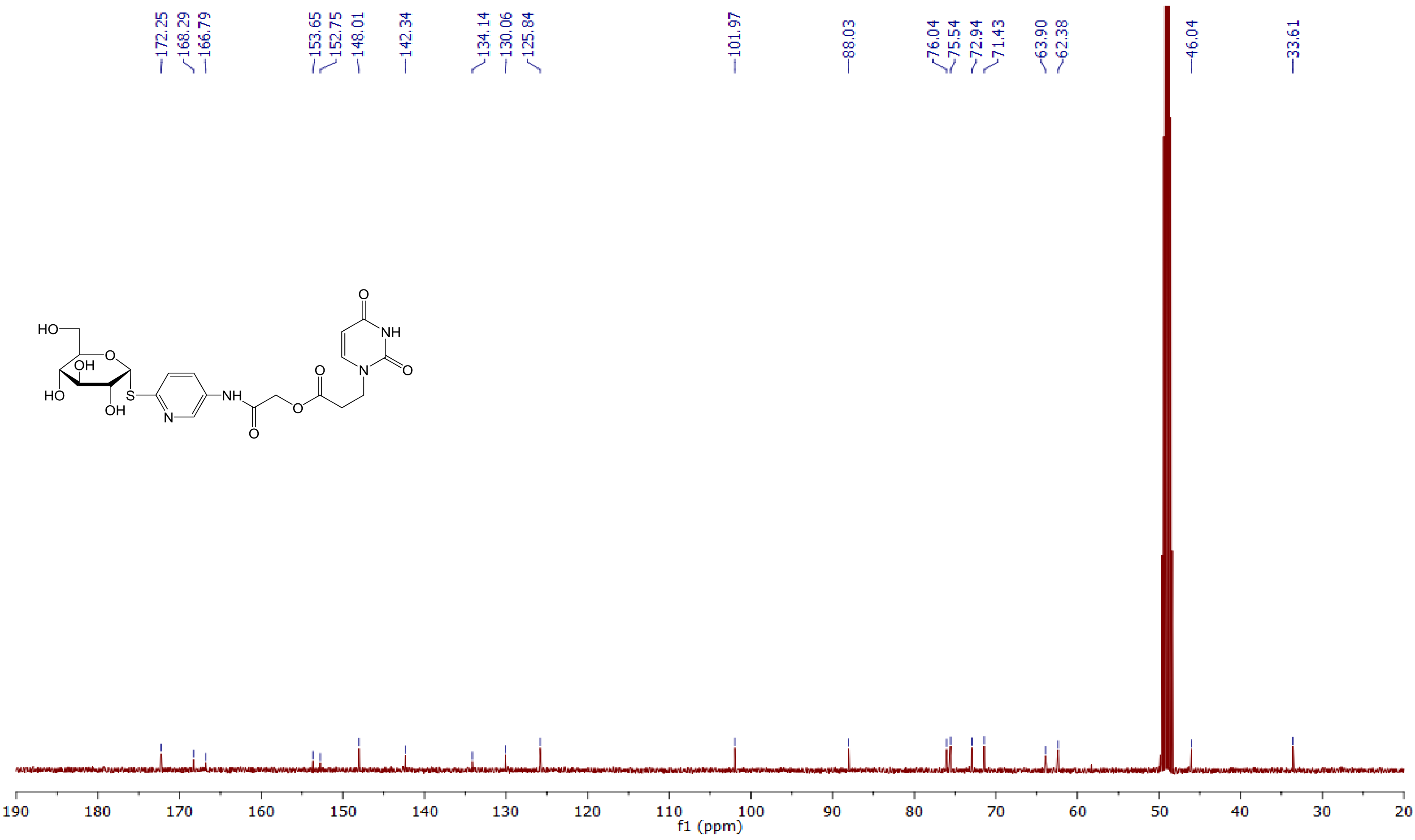

Fig. S78: ^{13}C NMR spectrum of glycoconjugate **61**

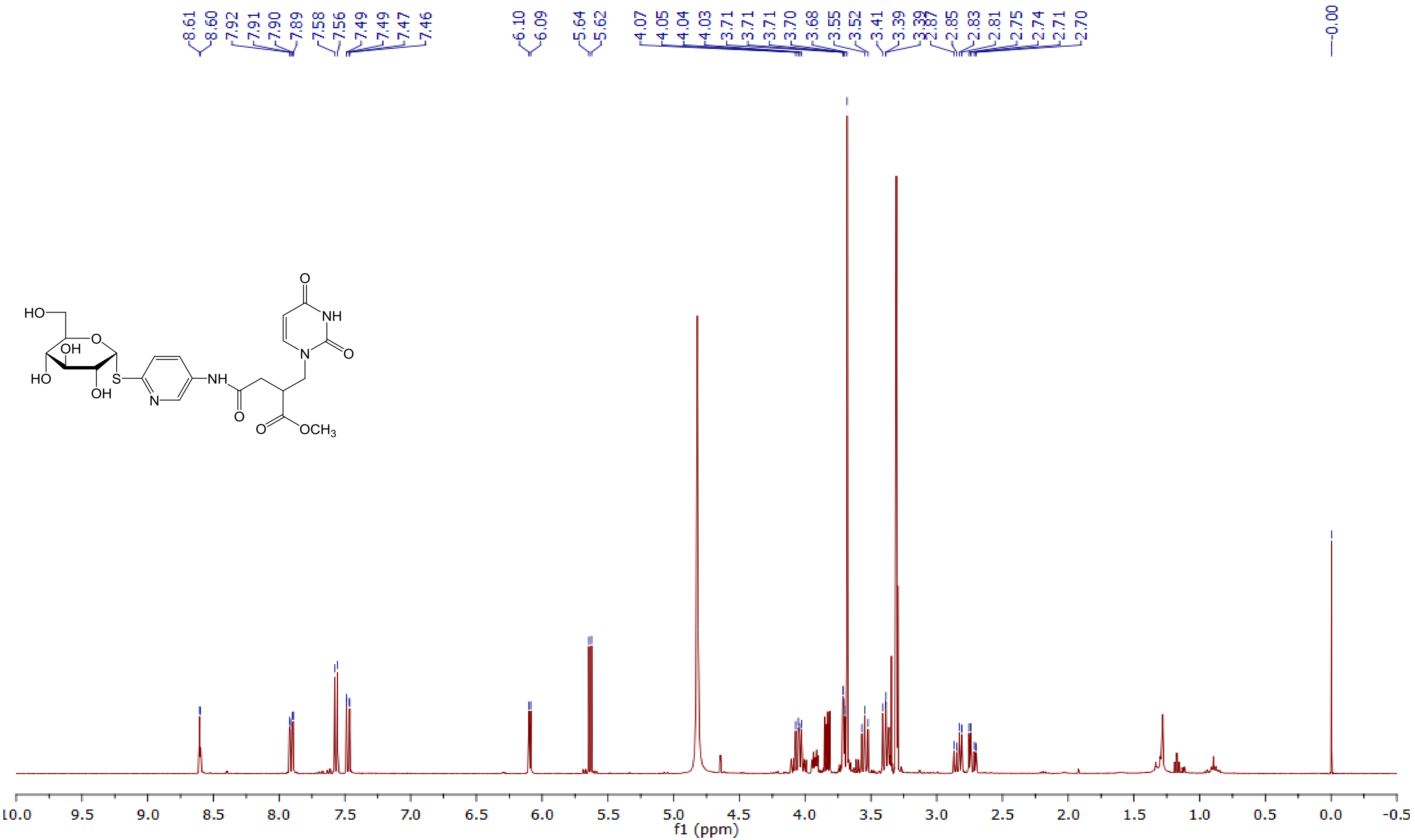

Fig. S79: ^{1}H NMR spectrum of glycoconjugate **62**

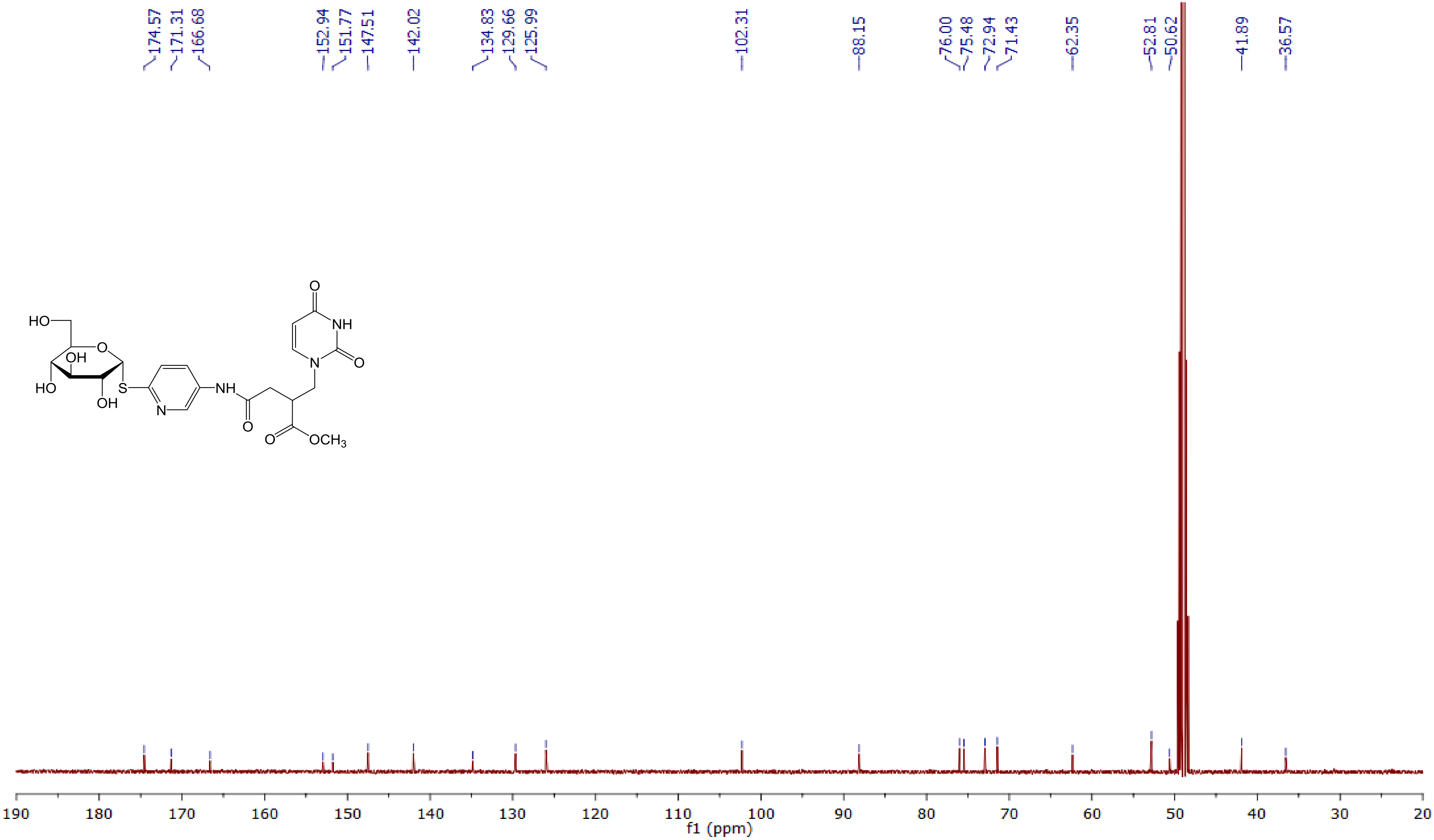

Fig. S80: ^{13}C NMR spectrum of glycoconjugate **62**

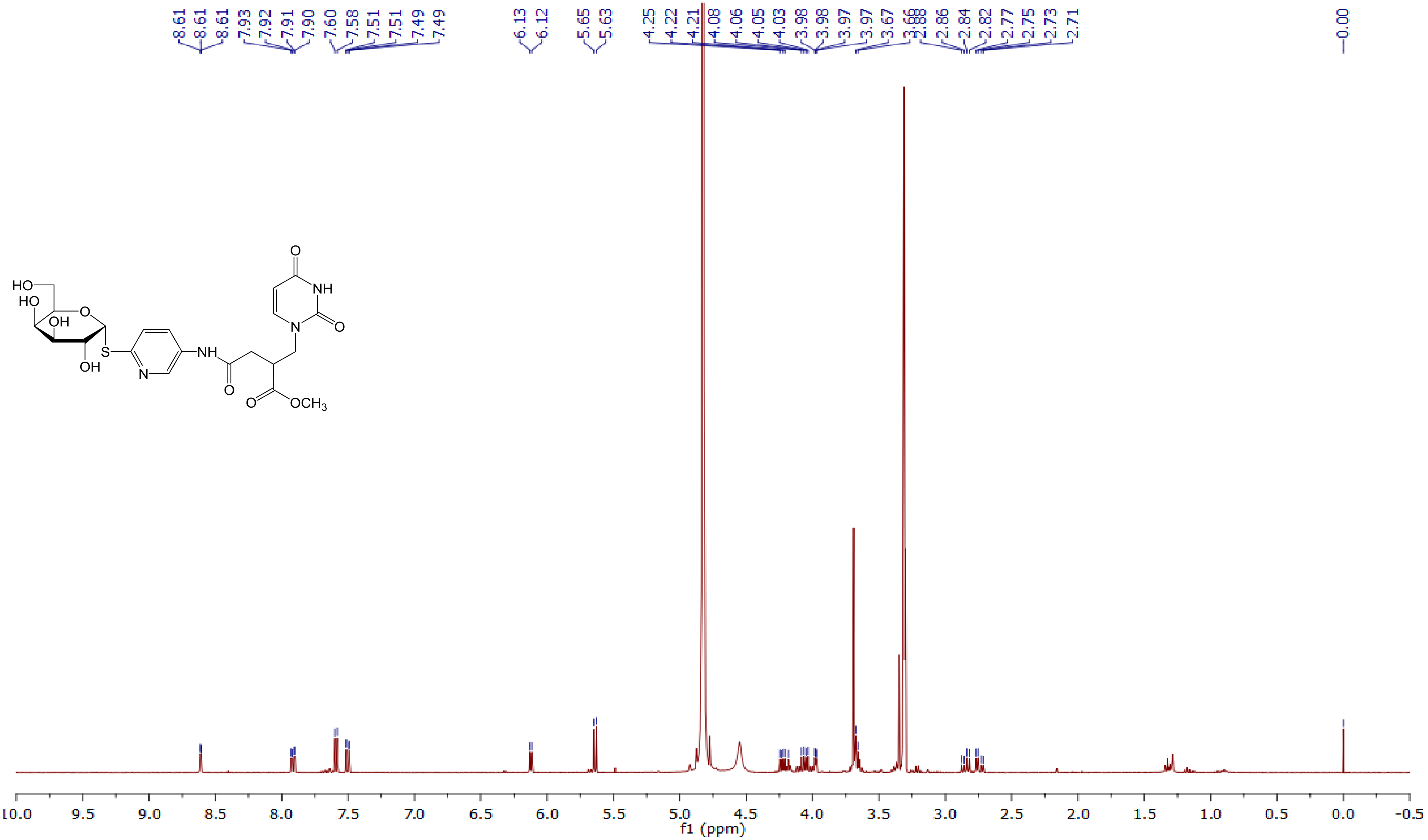

Fig. S81: ^{1}H NMR spectrum of glycoconjugate **63**

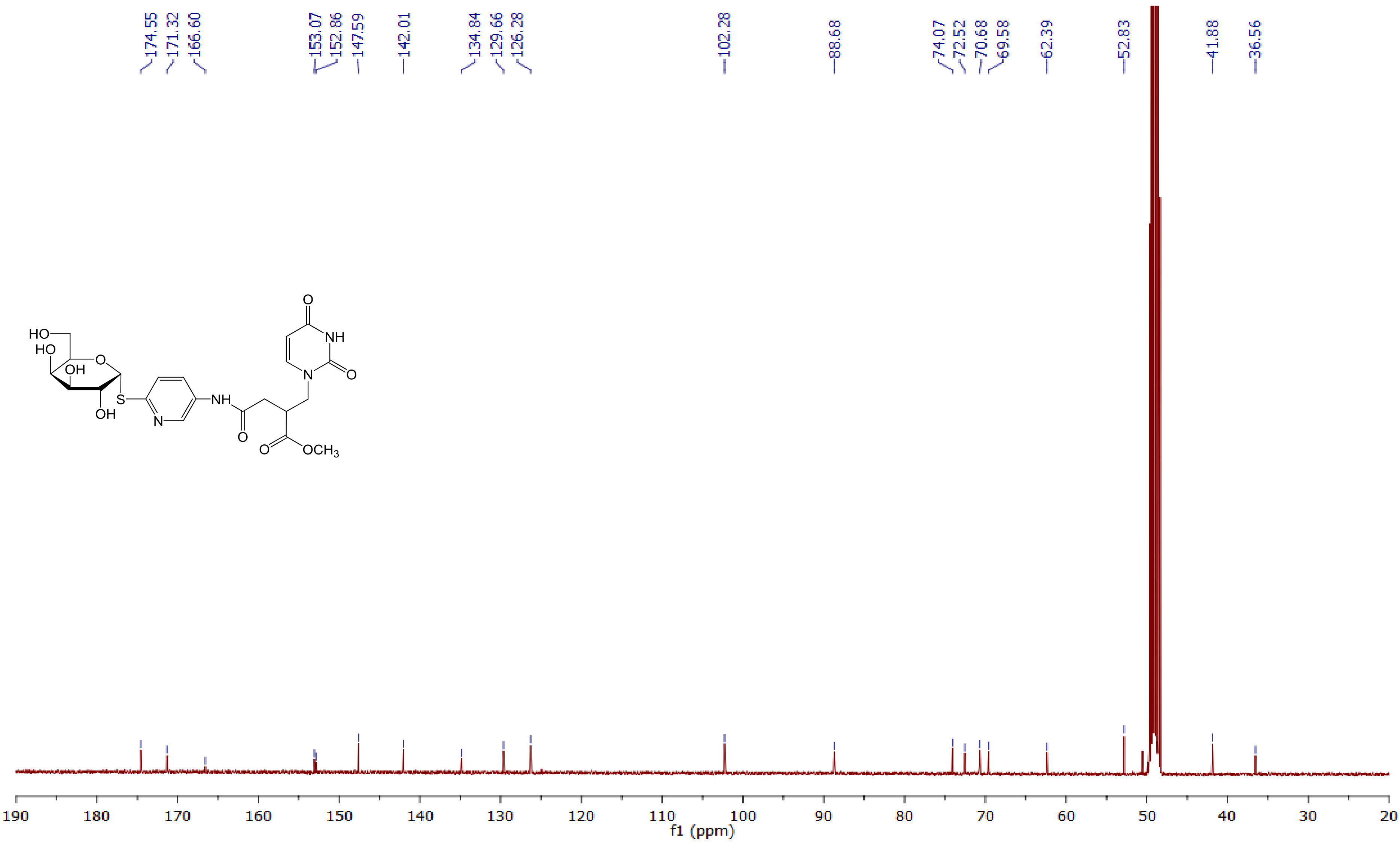

Fig. S82: ^{13}C NMR spectrum of glycoconjugate **63**

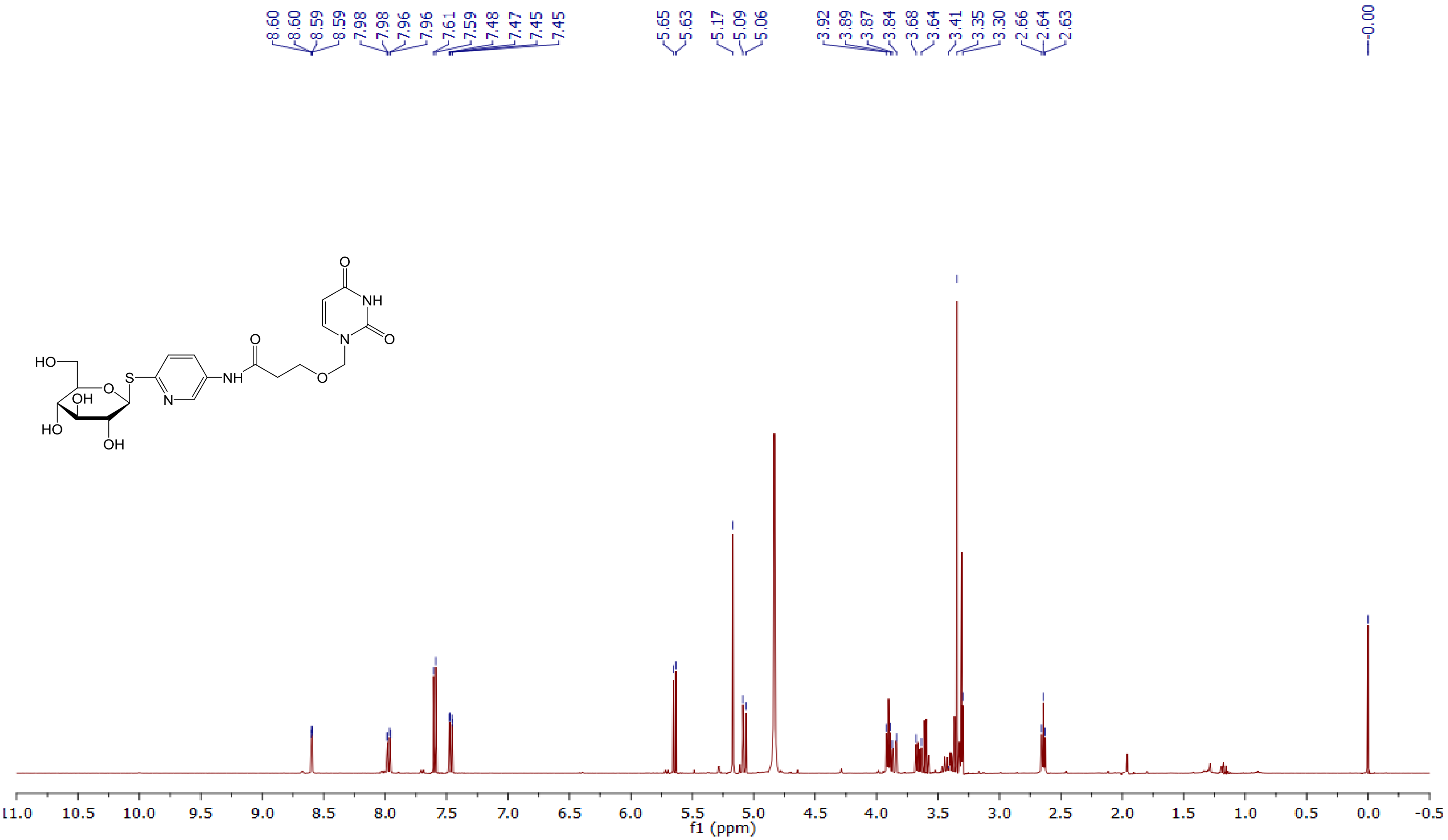

Fig. S83: ^{1}H NMR spectrum of glycoconjugate **64**

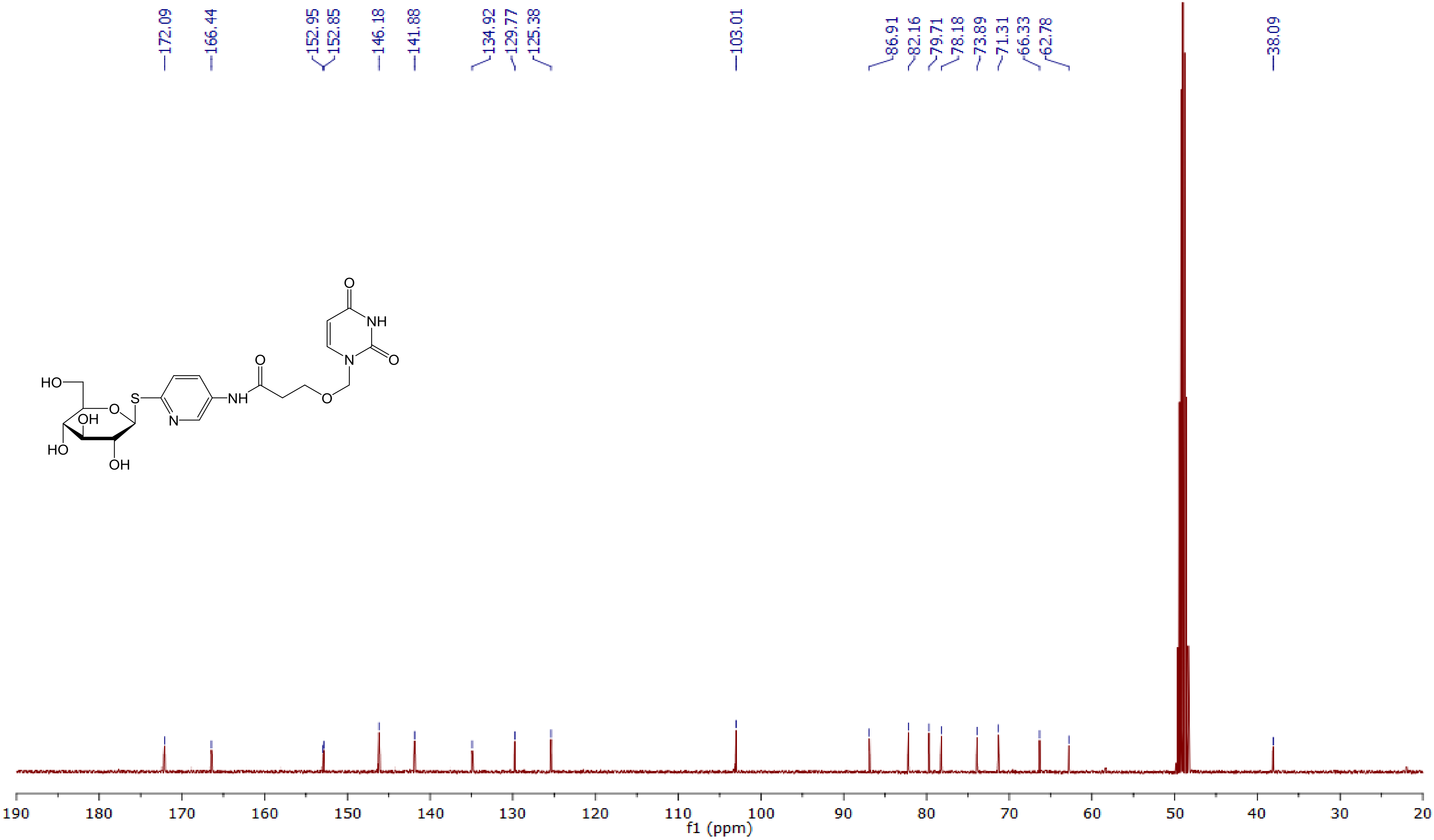

Fig. S84: ^{13}C NMR spectrum of glycoconjugate **64**

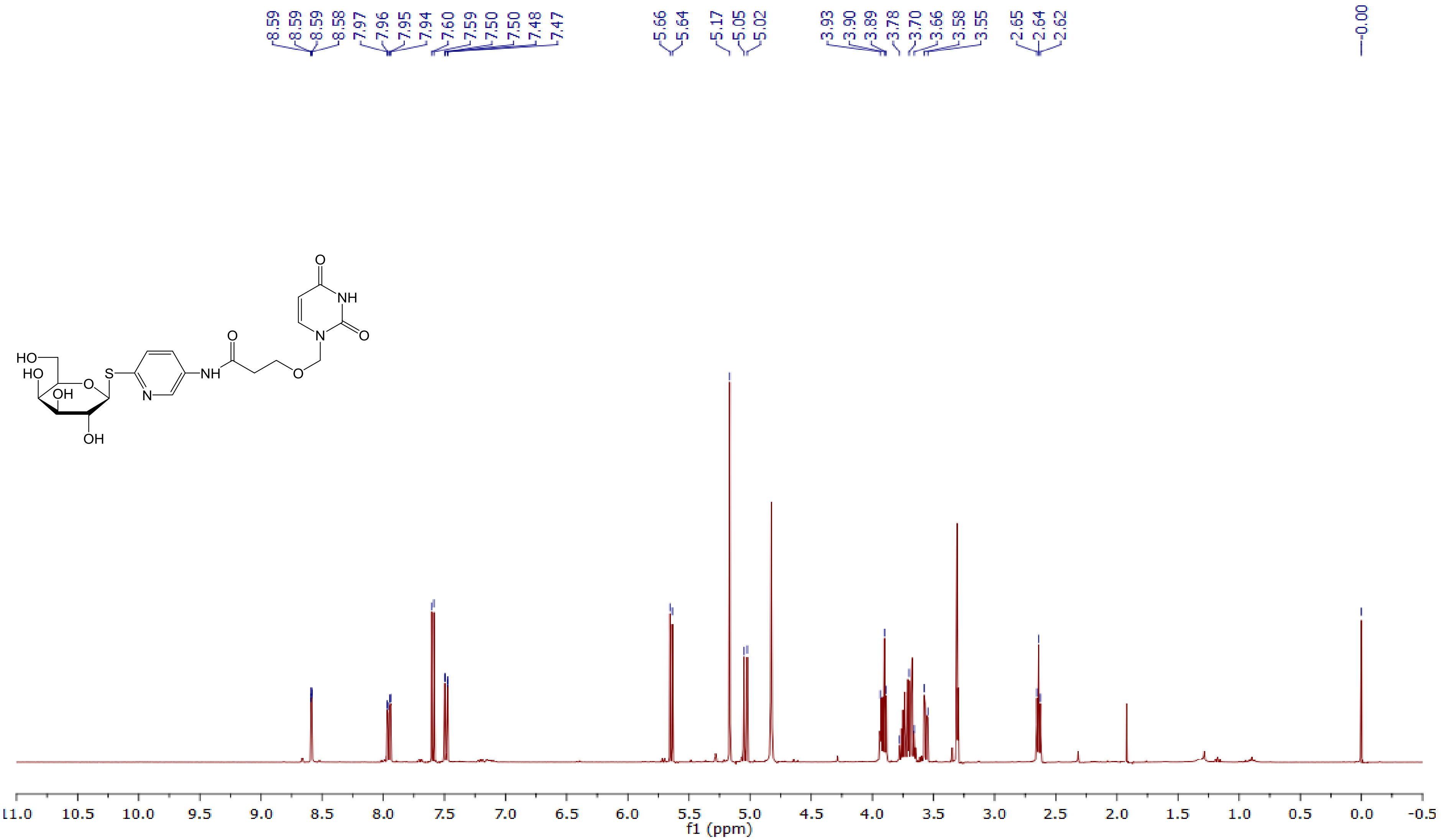

Fig. S85: ^{1}H NMR spectrum of glycoconjugate **65**

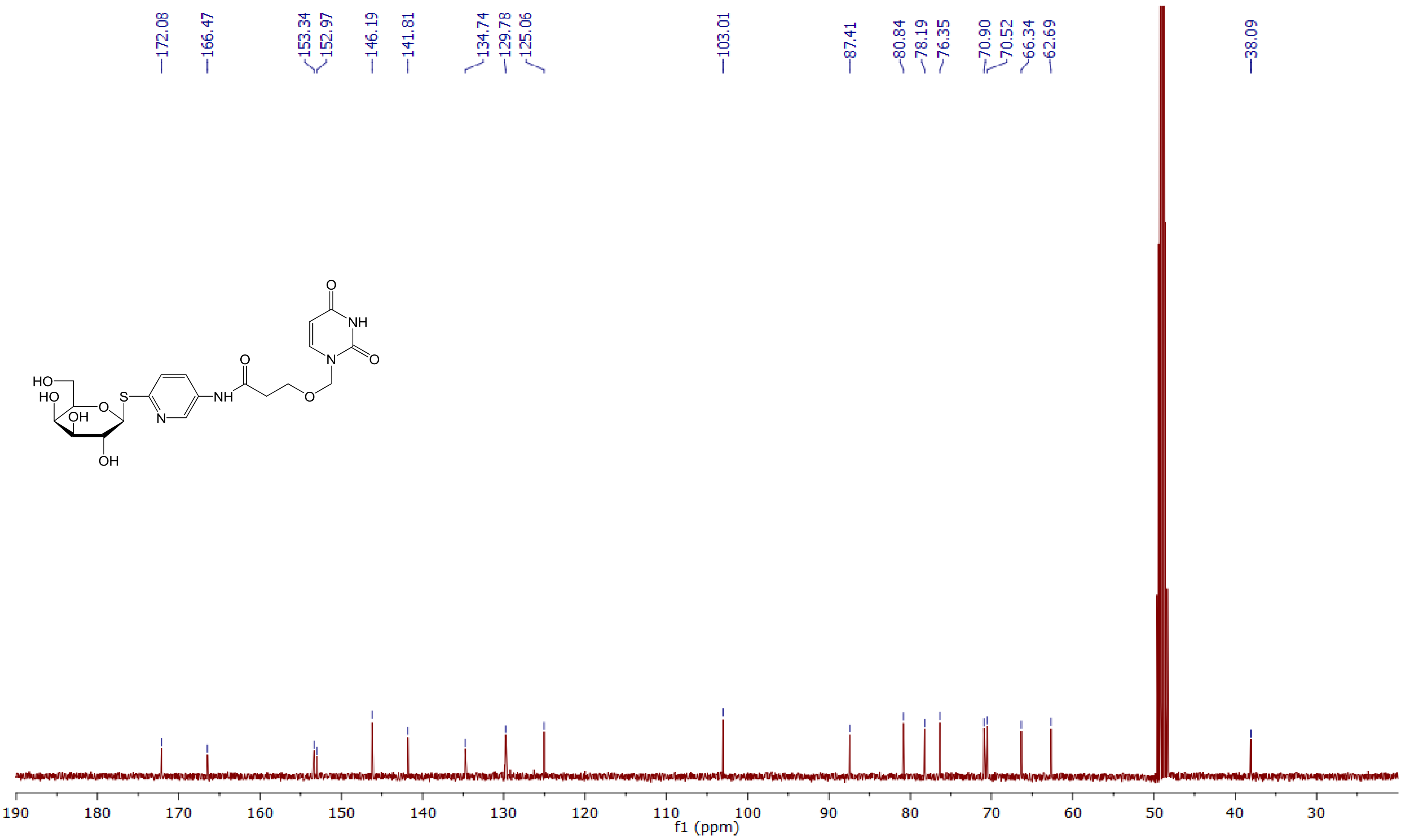

Fig. S86: ^{13}C NMR spectrum of glycoconjugate **65**
